# Synthesis of functionalised β-keto amides by aminoacylation/domino fragmentation of β-enamino amides

**DOI:** 10.3762/bjoc.14.238

**Published:** 2018-10-10

**Authors:** Pavel Yanev, Plamen Angelov

**Affiliations:** 1 Department of Organic Chemistry, University of Plovdiv Paisii Hilendarski, 24 Tsar Asen Str., 4000 Plovdiv, Bulgaria

**Keywords:** amino acids, C-acylation, domino reaction, enamines, enaminones, keto amides, retro-Mannich

## Abstract

Ethylenediamine-derived β-enamino amides are used as equivalents of amide enolate synthons in *C*-acylation reactions with *N*-protected amino acids. Domino fragmentation of the obtained intermediates leads to functionalised β-keto amides, bearing a protected amino group in their side chain.

## Introduction

The acylation of amide enolates is one of the main synthetic approaches towards β-keto amides and has found numerous applications [1–9]. However, while the generation of tertiary amide enolates is straightforward, this is not the case with secondary and primary amides. The NH acidity of the latter compounds necessitates either a double deprotonation to ambident 1,3-dianions [10] or proper masking of the amide functionality prior to deprotonation [11–12]. Consequently, there are very few published reagents that are synthetically equivalent to primary or secondary amide enolate synthons. With regard to acylation reactions, maybe most important among these are some nitriles, which can be successfully acylated at the alpha position [13–15] and then hydrated [13,16–20] to reveal a primary amide functionality. The dianion of silylated acetamide is another such example, although with very limited scope [21]. A contribution of ours in this area is the application of ethylenediamine-derived β-enamino amides as synthetic equivalents of primary and secondary amide enolate synthons in reactions with acid chlorides [22]. To extend the scope of the methodology, we now have explored the acylation of these reagents with suitably activated amino acids, in order to prepare functionalised β-keto amides, bearing a protected amino group in their side chain. Keto amides of this type are excellent precursors to various heterocyclic systems and are also interesting as building blocks for many biologically active substances [5–9,11,23–33].

## Results and Discussion

**Scheme 1:**
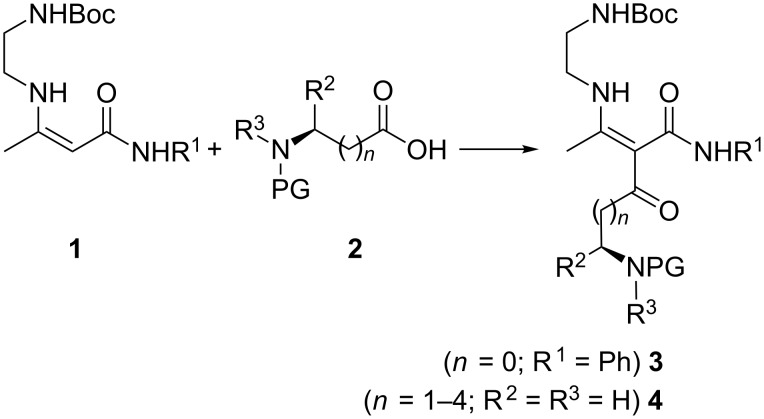
Preparation of α-C-acylated β-enamino amides **3** and **4**. Reagents and conditions: **2**, NMM, EtOCOCl, CH_2_Cl_2_, 0 °C, 5 min; then **1** and DMAP (0.2 equiv) in CH_2_Cl_2_, 0 °C to rt, 1 h.

For the purposes of this study we used two β-enamino amides **1** (R^1^ = H, Ph), that are easily prepared in quantitative yields by condensation of Boc-monoprotected ethylenediamine with commercially available acetoacetamides. As a first step we acylated the starting compounds **1** with mixed carbonic anhydrides of *N*-protected amino acids, applying a procedure previously developed by us for closely related analogues [34] (Scheme 1). Thus, the α-C-acylated intermediates **3a–j** (Table 1) and **4** (Table 2) were obtained in good yields and excellent chiral integrity (er **3c**–**f** ≥ 99:1). The phthaloyl-protected compounds **3k** and **3l** were obtained in low yields by the mixed carbonic anhydride method and after screening various alternative coupling reagents (mixed pivalic anhydrides, T3P, DCC, DIC, CDI) we were able to improve the yields of **3k**,**l** to 77% and 60%, respectively, only by using the corresponding *N*-phthaloyl amino acid chlorides [35–36] as acylating reagents. The increased yield however came at the expense of chiral integrity, with nearly full racemisation.

**Table 1:** Yields of compounds **3** and **5** (R^1^ = Ph, *n* = 0), obtained according to Scheme 1 and Scheme 2.

Compound **3**/**5**	R^2^	R^3^	PG	Yield **3** (%)	Yield **5** (%)

**a**	H	H	COOEt	88	57
**b**	H	H	Troc	75	52
**c**	CH_3_	H	COOEt	86	45
**d**	CH_3_	H	Troc	65	62
**e**	CH_2_Ph	H	COOEt	85	70
**f**	CH_2_Ph	H	Troc	60	54
**g**	H	CH_3_	COOEt	80	95
**h**	H	CH_3_	Troc	80	90
**i**	H	Ph	COOEt	70	85
**j**	H	COC_6_H_4_CO		94	90
**k**	CH_3_	COC_6_H_4_CO		45 (77)^a^	87
**l**	CH_2_Ph	COC_6_H_4_CO		18 (60)^a^	85

^a^Yields from acylation reaction with the corresponding acid chlorides.

**Table 2:** Yields of compounds **4** and **11** (R^2^ = R^3^ = H), obtained according to Scheme 1 and Scheme 6.

compound **4**/**11**	R^1^	*n*	PG	Yield **4** (%)	Yield **11** (%)

**a**	Ph	1	COOEt	90	95
**b**	Ph	2	COOEt	75	93
**c**	Ph	2	Cbz	88	74
**d**	Ph	3	Cbz	85	71
**e**	Ph	4	Cbz	75	68
**f**	Ph	2	Troc	57	90
**g**	Ph	3	Troc	77	60
**h**	Ph	4	Troc	65	73
**i**	H	1	Cbz	95	60
**j**	H	2	Cbz	82	58
**k**	H	3	Cbz	70	60
**l**	H	4	Cbz	73	55

After the series of α-aminoacyl (**3**) and ω-aminoacyl (**4**) intermediates were successfully prepared, we proceeded with the removal of the Boc protection group from the ethylenediamine moiety in order to trigger the domino fragmentation to the targeted β-keto amides. In the course of these experiments the two series of compounds showed markedly different behaviour.

### Domino fragmentation of α-aminoacyl intermediates **3** to *N*-protected γ-amino-β-ketoamides **5**

**Scheme 2:**
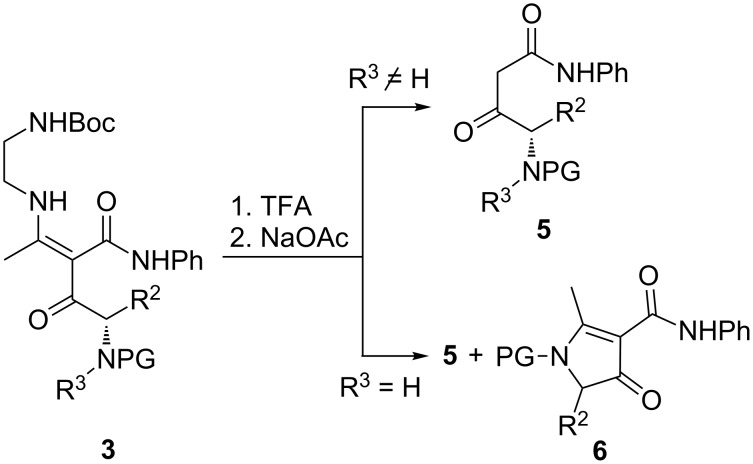
Domino fragmentation of **3** to β-keto amides **5** with competing cyclisation to pyrrolin-4-ones **6**. Reagents and conditions: TFA (neat, 5 min, rt), then 3 M aqueous NaOAc.

Initially we tried a procedure developed earlier by us for simpler β-keto amides (30 min in neat TFA, then aqueous NaOAc) [22]. In contrast to the preparation of non-functionalised β-keto amides, here these conditions led to satisfactory yields of keto amides **5** only for substrates **3** with R^3^ ≠ H (**3g**–**l**), while in all other cases (**3a**–**f**, R^3^ = H) gave significant amounts of pyrrolin-4-ones **6** as competing side products (Scheme 2).

**Scheme 3:**
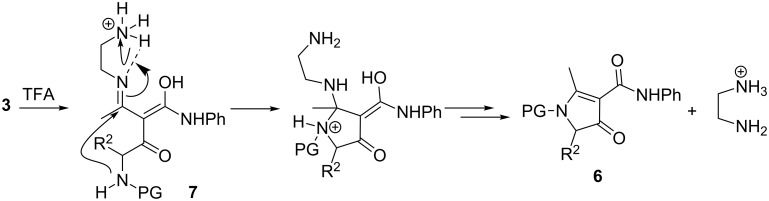
Proposed mechanism for the formation of side products **6**.

**Scheme 4:**
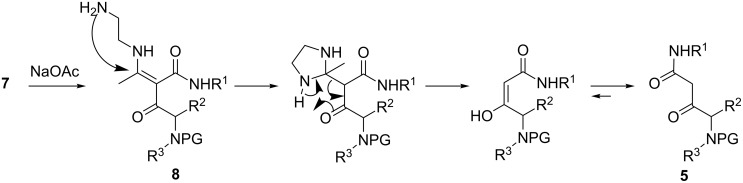
Proposed mechanism for the formation of the target β-keto amides **5**.

The relative amount of the side products **6** depended on the deprotection conditions and mainly on the duration of the exposure of **3** to acid. Given enough time, the treatment in neat or diluted TFA led to a complete transformation of **3a**–**f** into **6** without formation of the desired β-keto amides **5a–f** at all. On the contrary – when the deprotection was carried out for 5 min in neat TFA, the pyrrolinones **6** were the minor product and the β-keto amides **5a–f** were obtained in higher, although still moderate, yields (Table 1). These results indicated that the acidic conditions required for the Boc deprotection favoured the unwanted side process (Scheme 3) and the aza-Michael/retro-Mannich sequence only takes place after the addition of NaOAc (Scheme 4).

**Scheme 5:**
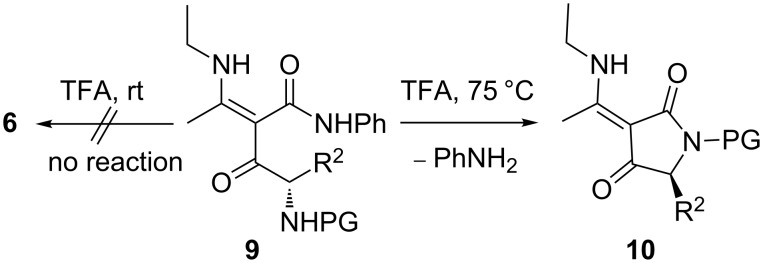
Reactivity of analogues **9**, lacking the auxiliary amino group [34].

It can be hypothesized that in acidic media the ammonium species **7**, resulting from the deprotection of **3** followed by protonation, exists predominantly as an imino tautomer, stabilised by intramolecular H-bonding. Such a tautomer would be susceptible to favourable *5-exo-trig* process, leading eventually to the pyrrolin-4-ones **6**. This hypothesis is strongly supported by the behaviour of analogues **9** in which the auxiliary amino group is absent (Scheme 5). Under identical conditions (neat TFA, rt) compounds **9** do not react even after 12 hours. A reaction occurs only upon heating in TFA solution, but then the cyclisation mode is completely different and leads to enaminotetramic derivatives **10** instead of pyrrolin-4-ones **6** [34]. Another drawback caused by the acidic deprotection conditions was the loss of chiral integrity. When the deprotection of **3** was performed in neat TFA for 5 min, the keto amides **5c–f** were obtained in poor enantiomeric excess and the concomitantly formed pyrrolinones **6c–f** were fully racemic. This again is in sharp contrast to analogues **9**, which cyclise to **10** with excellent retention of configuration [34].

Higher enantiomeric ratios for compounds **5** (87:13–80:20) were obtained with TFA/CH_2_Cl_2_ (1:4) and 15 min deprotection time, without any change in the yields indicated in Table 1. Experiments with more dilute TFA required longer deprotection time, which in turn gave advantage to the unwanted cyclisation to pyrrolinones **6** and lowered the yields of compounds **5**. Only the phthaloyl-protected derivative **3k** gave the corresponding keto amide **5k** in both, high yield and good er (95:5) when the Boc-deprotection was performed in 1:9 TFA/CH_2_Cl_2_ for 30 min.

### Domino fragmentation of ω-aminoacyl intermediates **4** to *N*-protected ω-amino-β-ketoamides **11**

**Scheme 6:**
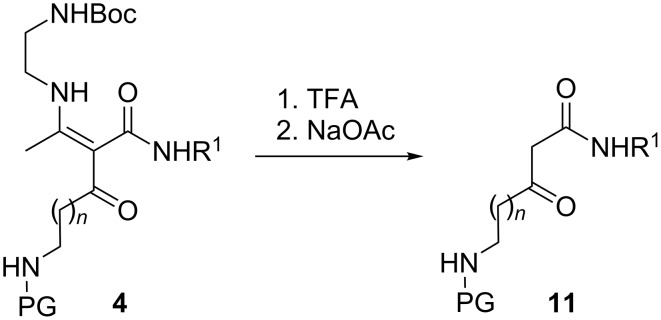
Domino fragmentation of compounds **4** (R^2^ = R^3^ = H) to *N*-protected ω-amino-β-keto amides **11**.

The longer chain in compounds **4** precludes the favourable *5-exo-trig* process discussed earlier (Scheme 3) and accordingly there was no competing formation of cyclic side products. A different type of unwanted cyclisation occurred with the GABA derivatives **4b**,**c**,**f**,**j** (*n* = 2) but here the competing process was slower and could be effectively avoided by limiting the deprotection time to 5 min in neat TFA. In this way a series of ω-amino-β-keto amides **11** with variable chain lengths and *N*-protection groups was successfully obtained (Scheme 6, Table 2).

**Scheme 7:**
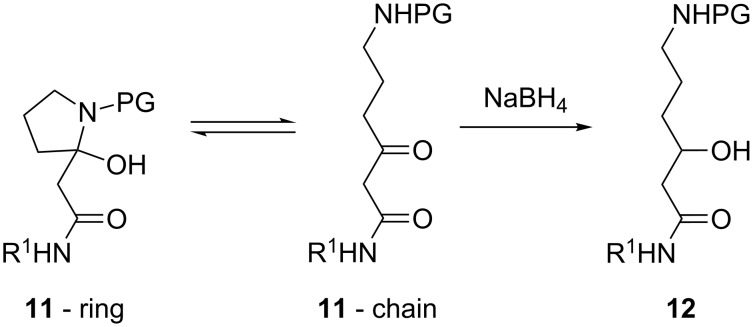
Ring–chain tautomerism in compounds **11b**,**c**,**f**,**j** (*n* = 2) and reduction to the corresponding β-hydroxy amides **12**.

Minor amounts of the enol tautomer (1–10%) were present in the CDCl_3_ solutions of all β-keto amides **5** and **11**. Along with that, the ^1^H NMR spectra of the GABA-derivatives **11b**,**c**,**f**,**j** (*n* = 2) in CDCl_3_ indicated a ring–chain tautomeric equilibrium with 55–75% share of the open chain form. Although an estimation of the tautomeric ratio was possible through a comparison of selected peak areas, the partial overlap and broadening of other signals prevented a full and unequivocal assignment. To circumvent this issue, we chose to lock the chain form by reducing the keto group in **11** with NaBH_4_ (Scheme 7). The alcohols **12** obtained in this way gave clean and well resolved NMR spectra. Similar tautomerism was observed in **11d**,**b**,**g**,**k** (*n* = 3), but with negligible proportion of the ring tautomer.

## Conclusion

In conclusion, we have demonstrated that *N*-protected γ-amino-β-keto amides and ω-amino-β-keto amides can be obtained from amino acids and β-enamino amides through the aza-Michael/retro-Mannich domino approach. A competing cyclisation to pyrrolin-4-ones limits the range of accessible γ-amino-derivatives and imposes the requirement for tertiary-substituted nitrogen in the aminoacyl moiety. There is no such limitation for the ω-amino derivatives.

## Supporting Information

File 1 Full experimental details and analytical data.

File 2 HPLC data and processed NMR spectra.
