# Supplementary material for: Synthesis of functionalised β-keto amides by aminoacylation/domino fragmentation of β-enamino amides
Source: Beilstein J Org Chem. 2018 Oct 10;14:2602–6. doi: 10.3762/bjoc.14.238 (PMC6204755; doi:10.3762/bjoc.14.238)
Supplement: File 1 — Full experimental details and analytical data. [file Beilstein_J_Org_Chem-14-2602-s001.pdf]

Supporting Information File 1

for

Synthesis of functionalised β-keto amides by aminoacylation/domino fragmentation of β-enamino amides

Pavel Yanev and Plamen Angelov*

Address: Department of Organic Chemistry, University of Plovdiv Paisii Hilendarski, 24 Tsar Asen Str., 4000 Plovdiv, Bulgaria

Email: Plamen Angelov - angelov@uni-plovdiv.bg

*Corresponding author

Full experimental details and analytical data

General

All reagents and solvents were purchased from commercial suppliers (Sigma-Aldrich or Merck) and were used without further purification. Boc-monoprotected ethylenediamine [1] and N-protected amino acids [2] were prepared according to published literature procedures. NMR spectra were run on Bruker Avance AV600 (600/150 MHz $^{1}H/^{13}C$) or DRX250 (250/62.5 MHz $^{1}H/^{13}C$) spectrometers and chemical shifts (δ, ppm) are reported downfield from TMS. Mass spectral measurements were performed on a Thermo Scientific Q Exactive hybrid quadrupole-orbitrap mass spectrometer. The enantiomeric ratios of all chiral products were determined using Phenomenex Lux 5 μm Amylose-1 or Amylose-2 chiral 250 × 4.6 mm columns on a Knauer Smartline or Hewlett-Packard 1090 HPLC systems, at a flow rate of 1 mL/min and detection at 310 nm (compounds **3**) or 244 nm (compounds **5**). Eluents for the chiral separations are specified in Supporting Information File 2, along with the chromatograms. IR spectra were recorded on Perkin-Elmer 1750 FTIR spectrometer and only characteristic peaks are listed; TLC was done on aluminium-backed Silica gel 60 sheets (Merck) with $KMnO_4$ staining; Melting points were measured on Boetius hot stage apparatus and are not corrected.

β-Enamino amides 1; general procedure

A magnetically stirred mixture of Boc-monoprotected ethylenediamine [1] (1.60 g, 10 mmol), the corresponding acetoacetamide (10 mmol) and anhydrous Na_2SO_4 (1.56 g, 11 mmol) in CH_2Cl_2 (50 mL) was heated for 1 h at boiling temperature under reflux. The warm reaction mixture was then immediately filtered through a sintered glass filter and the solvent was distilled off. The residue was triturated with a small amount of diethyl ether to give the corresponding β-enamino amide **1** as a white crystalline solid. Compounds **1** are sufficiently stable in air and can be stored at rt, but easily decompose on silica gel and cannot be monitored by TLC.

[2-(2-Carbamoyl-1-methyl-vinylamino)-ethyl]-carbamic acid tert-butyl ester (1, R^1 = H)

Yield: 2.30 g (95%); White solid; mp 120 – 122 °C.

IR (KBr): 3484, 3341, 3158, 1674, 1637, 1615 cm^{-1}.

^1H NMR ($CDCl_3$, 250 MHz): δ = 9.19 (br s, 1H), 5.03 (br s, 1H), 4.85 (br s, 2H), 4.38 (s, 1H), 3.20 – 3.40 (m, 4H), 1.90 (s, 3H), 1.45 (s, 9H).

^{13}C NMR ($CDCl_3$, 62.5 MHz): δ = 172.8, 160.3, 156.0, 84.3, 79.5, 42.7, 41.5, 28.4, 19.3.

HRMS (ES+): *m/z* $[M+Na]^+$ calcd for $C_{11}H_{21}N_3NaO_3^+$: 266.1475; found: 266.1480.

[2-(1-Methyl-2-phenylcarbamoyl-vinylamino)-ethyl]-carbamic acid tert-butyl ester (1, R^1 = Ph)

Yield: 3.05 g (95%); White solid; mp 161 – 162 °C.

IR (KBr): 3327, 1696, 1637, 1611 cm^{-1}.

^1H NMR ($CDCl_3$, 250 MHz): δ = 9.28 (br s, 1H), 7.41 – 7.44 (m, 2H), 7.24 – 7.30 (m, 2H), 6.98 – 7.05 (m, 1H), 6.71 (br s, 1H), 4.86 (br s, 1H), 4.45 (s, 1H), 3.20 – 3.40 (m, 4H), 1.92 (s, 3H), 1.44 (s, 9H).

^{13}C NMR ($CDCl_3$, 62.5 MHz): δ = 169.1, 160.5, 156.0, 139.1, 128.9, 123.0, 119.9, 85.7, 79.6, 42.8, 41.4, 28.4, 19.5.

HRMS (ES+): *m/z* $[M+Na]^+$ calcd for $C_{17}H_{25}N_3NaO_3^+$: 342.1788; found: 342.1786.

Acylation of β-enamino amides 1 with amino acids; general procedure for the preparation of compounds 3 and 4

To a magnetically stirred solution of the corresponding N-protected amino acid **2** (1 mmol) in CH_2Cl_2 (5 mL) was added *N*-methylmorpholine (1 mmol, 0.11 mL). The solution was then placed in an ice bath and ethyl chloroformate (1 mmol, 0.1 mL) was added. The mixture was left to stir for 5 min [3] and after that a suspension of enamino amide **1** (1 mmol) and DMAP (0.2 mmol) in CH_2Cl_2 (10 mL) was added in one portion. The ice bath was removed and the reaction mixture left to stir for one more hour at rt. Then the reaction mixture was transferred to a separatory funnel with additional 30 mL of CH_2Cl_2 and washed with aqueous (10:1) HCl. The aqueous layer was extracted with additional 30 mL of CH_2Cl_2, the combined organic layers were dried with anhydrous sodium sulfate, the drying agent was removed by filtration and the solvent was evaporated under reduced pressure. The crude α-C-acylated products **3** and **4** obtained in this way usually crystallize upon trituration with diethyl ether. Additional amounts of the products were obtained from the ethereal washings after flash column chromatography on silica gel with diethyl ether or diethyl ether/methanol (20:1) as the eluent.

Compounds **3g–i** were registered as mixtures of rotamers, with some of the signals coalescent or doubled, as indicated below.

Acylation with *N*-phthaloyl amino acid chlorides

A solution of the corresponding acid chloride [4] (1 mmol) in CH_2Cl_2 (2 mL) was added to a cooled (0 °C) and magnetically stirred suspension of enamino amide **1** (1 mmol, 320 mg), *N*-methylmorpholine (1 mmol, 0.11 mL) and DMAP (0.2 mmol, 25 mg) in CH_2Cl_2 (3 mL). The mixture was stirred for 30 min at 0 °C and for additional 1 h at rt. Then it was diluted with 30 mL CH_2Cl_2 and worked up as described above.

[4-(2-*tert*-Butoxycarbonylamino-ethylamino)-2-oxo-3-phenylcarbamoyl-pent-3-enyl]-carbamic acid ethyl ester (3a)

Yield: 395 mg (88%); White solid; R_f = 0.65 (Et_2O/CH_3OH, 20:1); mp 133 – 134 ^oC.

IR (KBr): 3351, 2982, 1701, 1644, 1601, 1535 cm^{-1}.

^1H NMR (DMSO-d_6, 600 MHz): δ = 11.36 (t, *J* = 5.9, 1H, *NH*), 10.17 (s, 1H, *NH*), 7.65 (d, *J* = 7.6, 2H), 7.30 (t, *J* = 7.6, 2H), 7.05 (m, 2H, *NH+ArH*), 6.94 (t, *J* = 5.9, 1H, *NH*), 3.95 (q, *J* = 7.0, 2H), 3.83 (d, *J* = 5.9, 2H), 3.38 (m, 2H), 3.11 (m, 2H), 2.02 (s, 3H), 1.38 (s, 9H), 1.14 (t, *J* = 7.0, 3H).

^{13}C NMR (DMSO-d_6, 150 MHz): δ = 189.1, 167.9, 163.8, 157.1, 156.2, 140.0, 129.2, 123.8, 119.6, 107.0, 78.4, 60.1, 46.8, 42.5, 40.7, 28.7, 16.5, 15.1.

HRMS (ES+): *m/z* $[M+Na]^+$ calcd for $C_{22}H_{32}N_4NaO_6^+$: 471.2214; found: 471.2214.

[4-(2-*tert*-Butoxycarbonylamino-ethylamino)-2-oxo-3-phenylcarbamoyl-pent-3-enyl]-carbamic acid 2,2,2-trichloro-ethyl ester (3b)

Yield: 414 mg (75%); White solid; R_f = 0.30 (Et_2O); mp 123 – 125 ^oC.

IR (KBr): 3350, 2980, 1720, 1660, 1610, 1540cm^{-1}.

^1H NMR (DMSO-d_6, 600 MHz): δ = 11.35 (t, *J* = 5.9, 1H, *NH*), 10.19 (s, 1H, *NH*), 7.65 (d, *J* = 7.6, 2H), 7.63 (t, *J* = 5.9, 1H, *NH*), 7.31 (t, *J* = 7.6, 2H), 7.05 (m, 2H, *NH+ArH*), 4.77 (s, 2H), 3.89 (d, *J* = 5.9, 2H), 3.40 (m, 2H), 3.11 (m, 2H), 2.03 (s, 3H), 1.38 (s, 9H).

^{13}C NMR (DMSO-d_6, 150 MHz): δ = 188.3, 167.8, 164.0, 155.3, 139.9, 129.2, 123.8, 119.6, 106.9, 96.6, 78.4, 73.9, 46.9, 42.5, 40.7, 28.7, 16.6.

HRMS (ES+): *m/z* $[M+Na]^+$ calcd for $C_{22}H_{29}Cl_3N_4NaO_6^+$: 573.1045; found: 573.1049.

[4-(2-*tert*-Butoxycarbonylamino-ethylamino)-1-methyl-2-oxo-3-phenylcarbamoyl-pent-3-enyl]-carbamic acid ethyl ester (3c)

Yield: 397 mg (86%); White solid; R_f = 0.70 (Et_2O/CH_3OH, 20:1); mp 126 – 129 ^oC (L), 134 – 136 ^oC (DL).

IR (KBr): 3345, 2983, 1704, 1657, 1603, 1535 cm^{-1}.

^1H NMR (DMSO-d_6, 600 MHz): δ = 11.63 (br s, 1H, *NH*), 10.09 (s, 1H, *NH*), 7.65 (d, *J* = 7.6, 2H), 7.30 (t, *J* = 7.6, 2H), 7.05 (m, 2H, *NH+ArH*), 6.93 (br s, 1H, *NH*), 4.39 (m, 1H), 3.92 (m, 2H), 3.39 (m, 2H), 3.13 (m, 2H), 2.03 (s, 3H), 1.39 (s, 9H), 1.16 (d, *J* = 7.0, 3H), 1.11 (t, *J* = 7.0, 3H).

^{13}C NMR (DMSO-d_6, 150 MHz): δ = 193.3, 168.1, 165.0, 156.2, 156.0, 140.1, 129.1, 123,7, 119.7, 107.0, 78.4, 60.1, 51.7, 42.7, 40.5, 28.7, 19.5, 16.8, 15.1.

HRMS (ES+): *m/z* $[M+Na]^+$ calcd for $C_{23}H_{34}N_4NaO_6^+$: 485.2371; found: 485.2375.

[4-(2-*tert*-Butoxycarbonylamino-ethylamino)-1-methyl-2-oxo-3-phenylcarbamoyl-pent-3-enyl]-carbamic acid 2,2,2-trichloro-ethyl ester (3d)

Yield: 368 mg (65%); White solid; R_f = 0.33 (Et_2O); mp 89 – 92 ^oC (L), 150 – 152 ^oC (DL).

IR (KBr): 3347, 2982, 1719, 1657, 1603, 1539 cm^{-1}.

^{1}H NMR (DMSO-d_6, 600 MHz): δ = 11.61 (br s, 1H, *NH*), 10.10 (s, 1H, *NH*), 7.65 (d, *J* = 7.6, 2H), 7.58 (br s, 1H, *NH*), 7.30 (t, *J* = 7.6, 2H), 7.05 (m, 2H, *NH+ArH*), 4.77 (d, ^{2}J = 12.3, 1H), 4.70 (d, ^{2}J = 12.3, 1H), 4.45 (m, 1H), 3.40 (m, 2H), 3.13 (m, 2H), 2.04 (s, 3H), 1.39 (s, 9H), 1.22 (d, *J* = 7.0, 3H).

^{13}C NMR (DMSO-d_6, 150 MHz): δ = 185.5, 168.1, 165.1, 156.2, 154.1, 140.1, 129.1, 123.7, 119.7, 106.9, 96.6, 78.4, 73.8, 52.1, 42.7, 40.5, 28.7, 19.3, 16.9.

HRMS (ES+): *m/z* $[M+Na]^+$ calcd for $C_{23}H_{31}Cl_3N_4NaO_6^+$: 587.1201; found: 587.1207.

[1-Benzyl-4-(2-*tert*-butoxycarbonylamino-ethylamino)-2-oxo-3-phenylcarbamoyl-pent-3-enyl]-carbamic acid ethyl ester (3e)

Yield: 458 mg (85%); White solid; R_f = 0.55 (Et_2O/CH_3OH, 20:1); mp 84 – 86 °C (L), 152 – 155 °C (DL).

IR (KBr): 3327, 2982, 2935, 2872, 1695, 1611, 1540 cm^{-1}.

^{1}H NMR (DMSO-d_6, 600 MHz): δ = 11.64 (br s, 1H, *NH*), 10.17 (s, 1H, *NH*), 7.72 (d, *J* = 7.6, 2H), 7.33 (d, *J* = 7.6, 2H), 7.22 – 7.05 (m, 8H, *ArH+NH*), 4.53 (m, 1H,), 3.81 (<u>AB</u>X_3 m, 2H), 3.42 (m, 2H), 3.16 – 3.11 (m, 3H), 2.59 (m, 1H), 2.07 (s, 3H), 1.39 (s, 9H), 1.05 (t, *J* = 7.0, 3H).

^{13}C NMR (DMSO-d_6, 150 MHz): δ = 192.9, 168.3, 165.0, 156.3, 140.2, 139.6, 129.5, 129.2, 128.4, 126.5, 123.7, 119.7, 107.1, 78.4, 60.0, 58.6, 42.7, 40.6, 38.3, 28.7, 16.9, 15.1.

HRMS (ES+): *m/z* $[M+Na]^+$ calcd for $C_{29}H_{38}N_4NaO_6^+$: 561.2684; found: 561.2685.

[1-Benzyl-4-(2-*tert*-butoxycarbonylamino-ethylamino)-2-oxo-3-phenylcarbamoyl-pent-3-enyl]-carbamic acid 2,2,2-trichloro-ethyl ester (3f)

Yield: 385 mg (60%); White solid; R_f = 0.60 (Et_2O); mp 86 – 88 °C (L), 93 – 95 °C (DL).

IR (KBr): 3340, 2985, 2892, 1708, 1661, 1604, 1535 cm^{-1}.

^{1}H NMR (DMSO-d_6, 600 MHz): δ = 11.62 (br s, 1H, *NH*), 10.21 (s, 1H, *NH*), 7.80 (d, *J* = 8.2, 1H, *NH*), 7.72 (d, *J* = 7.6, 2H), 7.33 (d, *J* = 7.6, 2H), 7.23 – 7.05 (m, 7H, *ArH+NH*), 4.62 (AB quartet, ^{2}J = 12.3, 2H + m, 1H), 3.42 (m, 2H), 3.17 – 3.11 (m, 3H), 2.68 (m, 1H), 2.08 (s, 3H), 1.39 (s, 9H).

^{13}C NMR (DMSO-d_6, 150 MHz): δ = 192.1, 168.2, 165.1, 156.3, 154.4, 140.1, 139.4, 129.5, 129.2, 128.4, 126.5, 123.8, 119.7, 107.0, 96.6, 78.4, 73.7, 58.8, 42.7, 40.6, 38.2, 28.7, 16.7.

HRMS (ES+): *m/z* $[M+Na]^+$ calcd for $C_{29}H_{35}Cl_3N_4NaO_6^+$: 663.1514; found: 663.1516.

[4-(2-*tert*-Butoxycarbonylamino-ethylamino)-2-oxo-3-phenylcarbamoyl-pent-3-enyl]-methyl-carbamic acid ethyl ester (3g)

Yield: 370 mg (80%); White solid; R_f = 0.35 (Et_2O/CH_3OH, 20:1); mp 146 – 148 °C.

IR (KBr): 3280, 1710, 1647, 1620, 1540 cm^{-1}.

^{1}H NMR (DMSO-d_6, 600 MHz): δ = 11.38, 11.37 (t, *J* = 5.9, 1H, *NH*), 10.18, 10.17 (s, 1H, *NH*), 7.65 (d, *J* = 7.6, 2H), 7.30 (t, *J* = 7.6, 2H), 7.06 (m, 2H, *NH+ArH*), 4.07, 4.06 (s, 2H), 3.98, 3.93 (q, *J* = 7.0, 2H), 3.39 (m, 2H), 3.12 (m, 2H), 2.80, 2.76 (s, 3H), 2.03, 2.02 (s, 3H), 1.38 (s, 9H), 1.16, 1.08 (t, *J* = 7.0, 3H).

^{13}C NMR (DMSO-d_6, 150 MHz): δ = 188.5, 167.8, 164.1, 156.7, 156.2, 140.0, 129.2, 123.8, 119.6, 107.2, 78.4, 61.1/61.0, 54.9/54.5, 42.6, 40.6, 36.0/35.7, 28.7, 16.6, 15.1.

HRMS (ES+): *m/z* $[M+Na]^+$ calcd for $C_{23}H_{34}N_4NaO_6^+$: 485.2371; found: 485.2373.

[4-(2-*tert*-Butoxycarbonylamino-ethylamino)-2-oxo-3-phenylcarbamoyl-pent-3-enyl]-methyl-carbamic acid 2,2,2-trichloro-ethyl ester (3h)

Yield: 453 mg (80%); White solid; R_f = 0.60 (Et_2O/CH_3OH, 20:1); mp 141 – 143 °C.

IR (KBr): 3279, 1718, 1645, 1617, 1543 cm^{-1}.

^{1}H NMR (DMSO-d$_6$, 600 MHz): δ = 11.39, 11.38 (t, *J* = 5.9, 1H, *NH*), 10.20, 10.12 (s, 1H, *NH*), 7.66 (m, 2H), 7.31 (m, 2H), 7.07 (m, 2H), 4.80, 4.74 (s, 2H), 4.21, 4.14 (s, 2H), 3.39 (m, 2H), 3.12 (m, 2H), 2.92, 2.86 (s, 3H), 2.05, 2.04 (s, 3H), 1.38 (s, 9H).

^{13}C NMR (DMSO-d$_6$, 150 MHz): δ = 187.8, 167.7, 156.2, 154.9, 154.8, 140.0, 129.2, 123.8, 119.7, 107.1, 96.5, 78.4, 74.7/74.4, 55.2/54.6, 42.6, 40.5, 36.6/35.7, 28.7, 16.6.

HRMS (ES+): *m/z* [M+Na]$^+$ calcd for $C_{23}H_{31}Cl_3N_4NaO_6^+$: 587.1201; found: 587.1205.

[4-(2-*tert*-Butoxycarbonylamino-ethylamino)-2-oxo-3-phenylcarbamoyl-pent-3-enyl]-phenyl-carbamic acid ethyl ester (3i)

Yield: 367 mg (70%); White solid; R_f = 0.60 (Et_2O/CH_3OH, 20:1); mp 157 – 159 °C.

IR (KBr): 3335, 2985, 1713, 1566, 1531 cm^{-1}.

^{1}H NMR (DMSO-d$_6$, 600 MHz): δ = 11.36 (t, *J* = 5.9, 1H, *NH*), 10.19 (s, 1H, *NH*), 7.64 (d, *J* = 7.6, 2H), 7.32 – 7.28 (m, 6H), 7.18 (m, 1H), 7.08 (t, *J* = 5.3, 1H, *NH*), 7.05 (t, *J* = 7.6, 1H), 4.44 (s, 2H), 4.02 (m, 2H), 3.40 (m, 2H), 3.13 (m, 2H), 2.03 (s, 3H), 1.38 (s, 9H), 1.11 (br s, 3H).

^{13}C NMR (DMSO-d$_6$, 150 MHz): δ = 187.9, 167.8, 164.1, 156.2, 139.9, 129.2, 128.9, 123.8, 119.7, 78.4, 61.5, 42.6, 40.6, 28.7, 16.6, 14.9.

HRMS (ES+): *m/z* [M+Na]$^+$ calcd for $C_{28}H_{36}N_4NaO_6^+$: 547.2527; found: 547.2527.

{2-[4-(1,3-Dioxo-1,3-dihydroisoindol-2-yl)-1-methyl-3-oxo-2-phenylcarbamoyl-but-1-enylamino]-ethyl}-carbamic acid *tert*-butyl ester (3j)

Yield: 476 mg (94%); White solid; R_f = 0.50 (Et_2O/CH_3OH, 20:1); mp 174 – 176 °C.

IR (KBr): 3299, 2983, 1775, 1728, 1611, 1542 cm^{-1}.

^{1}H NMR (DMSO-d$_6$, 600 MHz): δ = 11.27 (t, *J* = 5.9, 1H, *NH*), 10.34 (s, 1H, *NH*), 7.90 – 7.85 (m, 4H), 7.71 (d, *J* = 7.6, 2H), 7.33 (t, *J* = 7.6, 2H), 7.07(t, *J* = 7.6, 1H), 7.01 (t, *J* = 5.3, 1H, *NH*), 4.49 (s, 2H), 3.38 (m, 2H), 3.10 (m, 2H), 2.07 (s, 3H), 1.27 (s, 9H),

^{13}C NMR (DMSO-d$_6$, 150 MHz): δ = 184.7, 168.2, 167.6, 165.0, 156.1, 139.9, 135.0, 132.3, 129.2, 123.9, 123.6, 119.7, 106.3, 78.3, 44.0, 42.7, 28.6, 16.9.

HRMS (ES+): *m/z* [M+Na]$^+$ calcd for $C_{27}H_{30}N_4NaO_6^+$: 529.2058; found: 529.2060.

{2-[4-(1,3-Dioxo-1,3-dihydro-isoindol-2-yl)-1-methyl-3-oxo-2-phenylcarbamoyl-pent-1-enylamino]-ethyl}-carbamic acid *tert*-butyl ester (3k)

Yield: 400 mg (77%); White solid; R_f = 0.43 (Et_2O/CH_3OH, 20:1); mp 160 – 162 °C.

IR (KBr): 3354, 2985, 2944, 1781, 1723, 1604, 1533 cm^{-1}.

^{1}H NMR (DMSO-d$_6$, 600 MHz): δ = 11.44 (t, *J* = 5.9, 1H, *NH*), 10.04 (s, 1H, *NH*), 7.74 – 7.69 (m, 4H), 7.42 (d, *J* = 7.6, 2H), 7.16 (t, *J* = 7.6, 2H), 7.06 (t, *J* = 5.3, 1H, *NH*), 6.96 (t, *J* = 7.6, 1H), 5.22 (q, *J* = 7.0, 1H), 3.39 (m, 2H), 3.12 (m, 2H), 1.99 (s, 3H), 1.48 (d, *J* = 7.0, 3H), 1.34 (s, 9H).

^{13}C NMR (DMSO-d$_6$, 150 MHz): δ = 188.8, 167.9, 167.8, 164.6, 156.2, 139.9, 134.7, 132.0, 128.8, 123.4, 123.2, 119.4, 106.5, 78.4, 51.4, 42.6, 28.6, 16.7, 15.4.

HRMS (ES+): *m/z* [M+Na]$^+$ calcd for $C_{28}H_{32}N_4NaO_6^+$: 543.2214; found: 543.2211.

{2-[4-(1,3-Dioxo-1,3-dihydroisoindol-2-yl)-1-methyl-3-oxo-5-phenyl-2-phenylcarbamoyl-pent-1-enylamino]-ethyl}-carbamic acid *tert*-butyl ester (3l)

Yield: 358 mg (60%); White solid; R_f = 0.20 (Et_2O); mp 151 – 153 °C.

IR (KBr): 3350, 2981, 2950, 1770, 1720, 1604 cm^{-1}.

^{1}H NMR (DMSO-d$_6$, 600 MHz): δ = 11.50 (br s, 1H, *NH*), 10.15 (s, 1H, *NH*), 7.72 – 7.65 (m, 4H), 7.49 (d, *J* = 7.6, 2H), 7.19 (t, *J* = 7.6, 2H), 7.13 – 7.04 (m, 6H, *ArH+NH*), 6.98 (t, *J* = 7.6, 1H), 5.47 (m, 1H), 3.49 – 3.35 (m, 4H), 3.14 (m, 2H), 2.03 (s, 3H), 1.33 (s, 9H).

^{13}C NMR (DMSO-d$_6$, 150 MHz): δ = 187.6, 168.0, 167.9, 165.2, 156.2, 139.9, 138.8, 134.8, 131.5, 129.1, 128.9, 128.7, 126.8, 123.6, 123.3, 119.4, 106.5, 78.4, 58.2, 42.7, 34.1, 28.6, 16.9.

HRMS (ES+): *m/z* [M+Na]$^+$ calcd for $C_{34}H_{36}N_4NaO_6^+$: 619.2527; found: 619.2525.

[5-(2-*tert*-Butoxycarbonylamino-ethylamino)-3-oxo-4-phenylcarbamoyl-hex-4-enyl]-carbamic acid ethyl ester (4a)

Yield: 416 mg (90%); White solid; R$_f$ = 0.50 (Et_2O/CH_3OH, 20:1); mp 84 – 86 °C.

IR (KBr): 3330, 3115, 1699, 1656, 1604 cm^{-1}.

^{1}H NMR (DMSO-d$_6$, 600 MHz): δ =11.57 (t, *J* = 5.9, 1H, *NH*), 10.13 (s, 1H, *NH*), 7.65 (d, *J* = 7.6, 2H), 7.30 (t, *J* = 7.6, 2H), 7.04 (m, 2H, *NH+ArH*), 6.90 (t, *J* = 5.9, 1H, *NH*), 3.90 (q, *J* = 7.0, 2H), 3.38 (m, 2H), 3.18 (m, 2H), 3.11 (m, 2H), 2.52 (m, 2H, dmso overlap), 2.00 (s, 3H), 1.39 (s, 9H), 1.10 (t, *J* = 7.0, 3H).

^{13}C NMR (DMSO-d$_6$, 150 MHz): δ = 191.8, 168.6, 163.4, 156.5, 156.2, 140.1, 129.1, 123.7, 119.7, 109.4, 78.4, 59.9, 42.5, 40.7, 39.3, 37.2, 28.7, 16.5, 15.1.

HRMS (ES+): *m/z* [M+Na]$^+$ calcd for $C_{23}H_{34}N_4NaO_6^+$: 485.2371; found: 485.2376.

[6-(2-*tert*-Butoxycarbonylamino-ethylamino)-4-oxo-5-phenylcarbamoyl-hept-5-enyl]-carbamic acid ethyl ester (4b)

Yield: 357 mg (75%); White solid; R$_f$ = 0.50 (Et_2O/CH_3OH, 20:1); mp 104 – 105 °C.

IR (KBr): 3372, 3271, 3129, 1727, 1702, 1651, 1599 cm^{-1}.

^{1}H NMR (DMSO-d$_6$, 600 MHz): δ =11.57 (t, *J* = 5.9, 1H, *NH*), 10.12 (s, 1H, *NH*), 7.64 (d, *J* = 7.6, 2H), 7.29 (t, *J* = 7.6, 2H), 7.04 (m, 3H, *NH+ArH*), 3.90 (q, *J* = 7.0, 2H), 3.38 (m, 2H), 3.10 (m, 2H), 2.89 (m, 2H), 2.31 (t, *J* = 7.6, 2H), 1.99 (s, 3H), 1.62 (p, *J* = 7.6, 2H), 1.38 (s, 9H), 1.10 (t, *J* = 7.0, 3H).

^{13}C NMR (DMSO-d$_6$, 150 MHz): δ = 193.5, 168.8, 163.0, 156.6, 156.2, 140.1, 129.1, 123.7, 119.7, 109.4, 78.4, 59.8, 42.4, 40.7, 40.6, 36.6, 28.7, 25.7, 16.4, 15.1.

HRMS (ES+): *m/z* [M+Na]$^+$ calcd for $C_{24}H_{36}N_4NaO_6^+$: 499.2527; found: 499.2530.

[6-(2-*tert*-Butoxycarbonylamino-ethylamino)-4-oxo-5-phenylcarbamoyl-hept-5-enyl]-carbamic acid benzyl ester (4c)

Yield: 474 mg (88%); White solid; R$_f$ = 0.45 (Et_2O/CH_3OH, 20:1); mp 120 – 122 °C.

IR (KBr): 3360, 3277, 2980, 2950, 1703, 1655, 1604, 1570, 1542 cm^{-1}.

^{1}H NMR (DMSO-d$_6$, 600 MHz): δ = 11.58 (t, *J* = 5.9, 1H, *NH*), 10.13 (s, 1H, *NH*), 7.65 (d, *J* = 7.6, 2H), 7.35 – 7.27 (m, 7H), 7.22 (t, *J* = 5.9, 1H, *NH*), 7.06 – 7.02 (m, 2H, *ArH+NH*), 4.95 (s, 2H), 3.36 (m, 2H), 3.10 (m, 2H), 2.94 (m, 2H), 2.33 (t, *J* = 7.0, 2H), 1.99 (s, 3H), 1.64 (p, *J* = 7.0, 2H), 1.38 (s, 9H).

^{13}C NMR (DMSO-d$_6$, 150 MHz): δ = 193.5, 168.8, 163.0, 156.5, 156.2, 140.1, 137.7, 131.7, 129.1, 128.8, 128.2, 123.7, 119.7, 109.4, 78.4, 65.5, 42.4, 40.7, 36.5, 28.7, 25.7, 16.4.

HRMS (ES+): *m/z* [M+Na]$^+$ calcd for $C_{29}H_{38}N_4NaO_6^+$: 561.2684; found: 561.2689.

[7-(2-*tert*-Butoxycarbonylamino-ethylamino)-5-oxo-6-phenylcarbamoyl-oct-6-enyl]-carbamic acid benzyl ester (4d)

Yield: 470 mg (85%); White solid; R$_f$ = 0.50 (Et_2O/CH_3OH, 20:1); mp 114 – 116 °C.

IR (KBr): 3375, 3302, 2863, 1644, 1596, 1533, 1488 cm^{-1}.

^{1}H NMR (DMSO-d_6, 600 MHz): δ = 11.61 (t, *J* = 5.9, 1H, *NH*), 10.12 (s, 1H, *NH*), 7.65 (d, *J* = 7.6, 2H), 7.36 – 7.27 (m, 7H), 7.19 (t, *J* = 5.9, 1H, *NH*), 7.06 – 7.02 (m, 2H, *ArH+NH*), 4.98 (s, 2H), 3.37 (m, 2H), 3.10 (m, 2H), 2.93 (m, 2H), 2.31 (t, *J* = 7.0, 2H), 1.99 (s, 3H), 1.49 (p, *J* = 7.0, 2H), 1.38 (s, 9H), 1.33 (p, *J* = 7.0, 2H).

^{13}C NMR (DMSO-d_6, 150 MHz): δ = 193.8, 168.8, 163.0, 156.5, 156.2, 140.1, 137.8, 129.1, 128.8, 128.2, 123.7, 119.6, 109.5, 78.4, 65.5, 42.4, 40.7, 38.9, 29.8, 28.7, 22.7, 16.4.

HRMS (ES+): *m/z* $[M+Na]^+$ calcd for $C_{30}H_{40}N_4NaO_6^+$: 575.2840; found: 575.2841.

[8-(2-*tert*-Butoxycarbonylamino-ethylamino)-6-oxo-7-phenylcarbamoyl-non-7-enyl]-carbamic acid benzyl ester (4e)

Yield: 425 mg (75%); White solid; R_f = 0.52 (Et_2O/CH_3OH, 20:1); mp 107 – 109 °C.

IR (KBr): 3355, 3280, 2980, 2950, 1710, 1655, 1605 cm^{-1}.

^{1}H NMR (DMSO-d_6, 600 MHz): δ = 11.61 (t, *J* = 5.9, 1H, *NH*), 10.12 (s, 1H, *NH*), 7.65 (d, *J* = 7.6, 2H), 7.36 – 7.27 (m, 7H), 7.19 (t, *J* = 5.9, 1H, *NH*), 7.06 – 7.02 (m, 2H, *ArH+NH*), 4.98 (s, 2H), 3.36 (m, 2H), 3.10 (m, 2H), 2.93 (m, 2H), 2.30 (t, *J* = 7.0, 2H), 1.99 (s, 3H), 1.49 (p, *J* = 7.0, 2H), 1.39 (s, 9H), 1.33 (p, *J* = 7.0, 2H), 1.17 (p, *J* = 7.0, 2H).

^{13}C NMR (DMSO-d_6, 150 MHz): δ = 194.0, 168.8, 162.9, 156.5, 156.2, 140.1, 137.8, 129.1, 128.8, 128.2, 128.1, 123.6, 119.6, 109.5, 78.4, 65.5, 42.4, 40.7, 40.6, 39.2, 29.9, 28.7, 26.7, 25.1, 16.4.

HRMS (ES+): *m/z* $[M+Na]^+$ calcd for $C_{31}H_{42}N_4NaO_6^+$: 589.2997; found: 589.2993.

[6-(2-*tert*-Butoxycarbonylamino-ethylamino)-4-oxo-5-phenylcarbamoyl-hept-5-enyl]-carbamic acid 2,2,2-trichloro-ethyl ester (4f)

Yield: 330 mg (57%); White solid; R_f = 0.60 (Et_2O/CH_3OH, 20:1); mp 90 – 92 °C.

IR (KBr): 3355, 3300, 3084, 2984, 1705, 1652, 1601, 1558 cm^{-1}.

^{1}H NMR (DMSO-d_6, 600 MHz): δ = 11.57 (t, *J* = 5.9, 1H, *NH*), 10.12 (s, 1H, *NH*), 7.66 (m, 3H, *ArH+NH*), 7.29 (t, *J* = 7.6, 2H), 7.04 (m, 2H, *ArH+NH*), 4.71 (s, 2H), 3.36 (m, 2H), 3.10 (m, 2H), 2.97 (m, 2H), 2.34 (t, *J* = 7.0, 2H), 1.99 (s, 3H), 1.66 (p, *J* = 7.0, 2H), 1.39 (s, 9H).

^{13}C NMR (DMSO-d_6, 150 MHz): δ = 193.4, 168.7, 163.0, 156.2, 154.8, 140.1, 129.1, 123.7, 119.7, 109.4, 96.8, 78.4, 73.7, 65.4, 42.4, 40.9, 40.7, 36.4, 28.7, 25.5, 16.4.

HRMS (ES+): *m/z* $[M+Na]^+$ calcd for $C_{24}H_{33}Cl_3N_4NaO_6^+$: 601.1358; found: 601.1361.

[7-(2-*tert*-Butoxycarbonylamino-ethylamino)-5-oxo-6-phenylcarbamoyl-oct-6-enyl]-carbamic acid 2,2,2-trichloro-ethyl ester (4g)

Yield: 457 mg (77%); White solid; R_f = 0.60 (Et_2O/CH_3OH, 20:1); mp 113 – 115 °C.

IR (KBr): 3339, 3074, 2953, 2892, 1730, 1657, 1604, 1540 cm^{-1}.

^{1}H NMR (DMSO-d_6, 600 MHz): δ = 11.60 (t, *J* = 5.9, 1H, *NH*), 10.12 (s, 1H, *NH*), 7.64 (m, 3H, *ArH+NH*), 7.29 (t, *J* = 7.6, 2H), 7.04 (m, 2H, *ArH+NH*), 4.76 (s, 2H), 3.37 (m, 2H), 3.10 (m, 2H), 2.95 (m, 2H), 2.31 (t, *J* = 7.0, 2H), 1.99 (s, 3H), 1.49 (p, *J* = 7.0, 2H), 1.39 (s, 9H), 1.36 (p, *J* = 7.0, 2H).

^{13}C NMR (DMSO-d_6, 150 MHz): δ = 193.8, 168.8, 163.0, 156.2, 154.8, 140.1, 123.7, 119.6, 109.5, 96.8, 78.4, 73.7, 42.4, 40.9, 40.7, 38.8, 29.6, 28.7, 22.6, 16.4.

HRMS (ES+): *m/z* $[M+Na]^+$ calcd for $C_{25}H_{35}Cl_3N_4NaO_6^+$: 615.1514; found: 615.1510.

[8-(2-*tert*-Butoxycarbonylamino-ethylamino)-6-oxo-7-phenylcarbamoyl-non-7-enyl]-carbamic acid 2,2,2-trichloro-ethyl ester (4h)

Yield: 395 mg (65%); Oil; R_f = 0.63 (Et_2O/CH_3OH, 20:1).

IR (KBr): 3345, 3080, 2983, 2950, 2883, 1729, 1662, 1605 cm^{-1}.

^{1}H NMR (DMSO-d_6, 600 MHz): δ = 11.60 (t, *J* = 5.9, 1H, *NH*), 10.11 (s, 1H, *NH*), 7.64 (m, 3H, *ArH+NH*), 7.29 (t, *J* = 7.6, 2H), 7.04 (m, 2H, *ArH+NH*), 4.76 (s, 2H), 3.36 (m, 2H), 3.09 (m, 2H), 2.95 (m, 2H), 2.30 (t, *J* = 7.0, 2H), 1.99 (s, 3H), 1.49 (m, 2H), 1.39 (s, 9H), 1.35 (m, 2H), 1.18 (m, 2H).

^{13}C NMR (DMSO-d_6, 150 MHz): δ = 193.9, 168.8, 162.9, 154.8, 140.1, 129.1, 119.6, 109.4, 96.8, 78.4, 73.7, 55.4, 42.4, 40.8, 40.7, 39.2, 29.6, 28.7, 26.5, 25.0, 16.4.

HRMS (ES+): *m/z* $[M+Na]^+$ calcd for $C_{26}H_{37}Cl_3N_4NaO_6^+$: 629.1671; found: 629.1672.

[5-(2-*tert*-Butoxycarbonylamino-ethylamino)-4-carbamoyl-3-oxo-hex-4-enyl]-carbamic acid benzyl ester (4i)

Yield: 426 mg (95%); Oil; R_f = 0.25 (Et_2O/CH_3OH, 10:1).

IR (KBr): 3349, 3033, 2985, 2961, 1702, 1646, 1602, 1583, 1533cm^{-1}.

^{1}H NMR (DMSO-d_6, 600 MHz): δ = 11.52 (t, *J* = 5.9, 1H, *NH*), 7.47 (br s, 1H, *NH*), 7.37 – 7.29 (m, 5H), 7.15 (br s, 2H, *NH*), 7.02 (t, *J* = 5.3, 1H, *NH*), 4.98 (s, 2H), 3.32 (m, 2H), 3.19 (m, 2H), 3.06 (m, 2H), 2.53 (t, *J* = 7.0, 2H), 1.99 (s, 3H), 1.37 (s, 9H).

^{13}C NMR (DMSO-d_6, 150 MHz): δ = 191.7, 171.9, 162.8, 156.4, 156.2, 137.7, 128.8, 128.2, 108.8, 78.4, 65.6, 42.4, 40.7, 39.2, 37.5, 28.7, 16.4.

HRMS (ES+): *m/z* $[M+Na]^+$ calcd for $C_{22}H_{32}N_4NaO_6^+$: 471.2214; found: 471.2214.

[6-(2-*tert*-Butoxycarbonylamino-ethylamino)-5-carbamoyl-4-oxo-hept-5-enyl]-carbamic acid benzyl ester (4j)

Yield: 379 mg (82%); White solid; R_f = 0.40 (Et_2O/CH_3OH, 10:1); mp 141 – 143 °C.

IR (KBr): 3392, 3300, 3206, 2989, 2950, 2917, 1690, 1681, 1605 cm^{-1}.

^{1}H NMR (DMSO-d_6, 600 MHz): δ = 11.52 (t, *J* = 5.9, 1H, *NH*), 7.45 (br s, 1H, *NH*), 7.37 – 7.29 (m, 5H), 7.25 (t, *J* = 5.9, 1H, *NH*), 7.12 (br s, 1H, *NH*), 7.02 (t, *J* = 5.9, 1H, *NH*), 4.99 (s, 2H), 3.30 (m, 2H), 3.06 (m, 2H), 2.95 (m, 2H), 2.33 (t, *J* = 7.0, 2H), 1.98 (s, 3H), 1.62 (p, *J* = 7.0, 2H), 1.38 (s, 9H).

^{13}C NMR (DMSO-d_6, 150 MHz): δ = 193.4, 172.2, 162.3, 156.5, 156.2, 137.7, 128.8, 128.2, 108.8, 78.4, 65.6, 42.3, 40.7, 36.4, 28.7, 25.9, 16.4.

HRMS (ES+): *m/z* $[M+Na]^+$ calcd for $C_{23}H_{34}N_4NaO_6^+$: 485.2371; found: 485.2376.

[7-(2-*tert*-Butoxycarbonylamino-ethylamino)-6-carbamoyl-5-oxo-oct-6-enyl]-carbamic acid benzyl ester (4k)

Yield: 334 mg (70%); White solid; R_f = 0.27 (Et_2O/CH_3OH, 10:1); mp 108 – 110 °C.

IR (KBr): 3342, 3207, 3033, 2982, 2966, 2874, 1696, 1614, 1560 cm^{-1}.

^{1}H NMR (DMSO-d_6, 600 MHz): δ = 11.53 (t, *J* = 5.9, 1H, *NH*), 7.43 (br s, 1H, *NH*), 7.37 – 7.29 (m, 5H), 7.22 (t, *J* = 5.9, 1H, *NH*), 7.10 (br s, 1H, *NH*), 7.02 (t, *J* = 5.3, 1H, *NH*), 4.99 (s, 2H), 3.30 (m, 2H), 3.06 (m, 2H), 2.95 (m, 2H), 2.31 (t, *J* = 7.6, 2H), 1.97 (s, 3H), 1.47 (p, *J* = 7.6, 2H), 1.38 (s, 9H), 1.36 (p, *J* = 7.6, 2H).

^{13}C NMR (DMSO-d_6, 150 MHz): δ = 193.8, 172.2, 162.2, 156.5, 156.2, 137.8, 128.8, 128.2, 108.8, 78.4, 65.6, 42.3, 40.8, 40.7, 38.7, 29.8, 28.7, 22.8, 16.3.

HRMS (ES+): *m/z* $[M+Na]^+$ calcd for $C_{24}H_{36}N_4NaO_6^+$: 499.2527; found: 499.2531.

[8-(2-*tert*-Butoxycarbonylamino-ethylamino)-7-carbamoyl-6-oxo-non-7-enyl]-carbamic acid benzyl ester (4l)

Yield: 358 mg (73%); White solid; R_f = 0.33 (Et_2O/CH_3OH, 10:1); mp 88 – 90 °C.

IR (KBr): 3373, 3205, 2971, 2897, 1699, 1675, 1597, 1574, 1557 cm^{-1}.

^{1}H NMR (DMSO-d_6, 600 MHz): δ = 11.53 (t, *J* = 5.9, 1H, *NH*), 7.43 (br s, 1H, *NH*), 7.37 – 7.29 (m, 5H), 7.23 (t, *J* = 5.3, 1H, *NH*), 7.09 (br s, 1H, *NH*), 7.02 (t, *J* = 5.3, 1H, *NH*), 4.99 (s, 2H), 3.30 (m, 2H), 3.06 (m, 2H), 2.96 (m, 2H), 2.31 (t, *J* = 7.6, 2H), 1.97 (s, 3H), 1.47 (p, *J* = 7.6, 2H), 1.38 (s, 9H), 1.36 (p, *J* = 7.6, 2H), 1.20 (p, *J* = 7.6, 2H).

^{13}C NMR (DMSO-d_6, 150 MHz): δ = 193.9, 172.2, 162.1, 156.5, 156.2, 137.8, 128.8, 128.2, 108.8, 78.4, 65.6, 42.3, 40.7, 39.1, 29.9, 28.7, 26.7, 25.2, 16.3.

HRMS (ES+): *m/z* $[M+Na]^+$ calcd for $C_{25}H_{38}N_4NaO_6^+$: 513.2684; found: 513.2685.

Functionalised β-keto amides 5 and 11; general procedure

To the corresponding precursor **3** or **4** (1 mmol) was added TFA (1 mL per 100 mg of substrate) and the mixture was stirred for 5 min at rt. Then, 3 mol/L aqueous solution of NaOAc (10 mL for each mL of TFA) was added, followed by CH_2Cl_2 (30–50 mL) and the mixture was left to stir intensely overnight (**4i–l**), for 6h (**3j–l**) [5] or for 2 h in all other examples. The layers were then separated and the aqueous layer was extracted two more times with CH_2Cl_2. The organic layers were combined, washed with a small amount of saturated aqueous $NaHCO_3$ (20 mL) and then dried over Na_2SO_4. The solvent was removed on a rotary evaporator and the residue was either purified by silica gel column chromatography (**5a–f**) or triturated and rinsed with diethyl ether or ether/petroleum in most other cases. The filtration of the ethereal washings through a short plug of silica gel affords additional amounts of the keto amides.

Variable proportions of the enol tautomer (1–10%) were registered in the $CDCl_3$ solutions of all β-keto amides, but only signals for the major keto tautomer are given in the textual NMR description. In addition to that, compounds **5g,h** were registered as mixtures of rotamers, with some of the signals doubled, as indicated below.

(2-Oxo-3-phenylcarbamoyl-propyl)-carbamic acid ethyl ester (5a)

Yield: 151 mg (57%); White solid; R_f = 0.35 (Et_2O); mp 116 – 117 °C.

IR (KBr): 3335, 3305, 3200, 3138, 1728, 1692, 1664, 1602 cm^{-1}.

^{1}H NMR ($CDCl_3$, 600 MHz): δ = 8.77 (s, 1H, *NH*), 7.44 (d, *J* = 7.6, 2H), 7.24 (t, *J* = 7.6, 2H), 7.05 (t, *J* = 7.6, 1H), 5.37 (br s, 1H, *NH*), 4.09 (d, *J* = 5.3, 2H), 4.06 (q, *J* = 7.0, 2H), 3.52 (s, 2H), 1.18 (t, *J* = 7.0, 3H).

^{13}C NMR ($CDCl_3$, 150 MHz): δ = 200.3, 163.1, 156.8, 137.4, 129.1, 124.8, 120.2, 61.6, 51.2, 47.5, 14.6.

HRMS (ES+): *m/z* $[M+Na]^+$ calcd for $C_{13}H_{16}N_2NaO_4^+$: 287.1002; found: 287.1002.

(2-Oxo-3-phenylcarbamoyl-propyl)-carbamic acid 2,2,2-trichloro-ethyl ester (5b)

Yield: 190 mg (52%); White solid; R_f = 0.30 (Et_2O/Petrol, 2:1); mp 105 – 107 °C.

IR (KBr): 3347, 2929, 1717, 1669, 1602, 1548 cm^{-1}.

^{1}H NMR ($CDCl_3$, 600 MHz): δ = 8.67 (s, 1H, *NH*), 7.52 (d, *J* = 7.6, 2H), 7.35 (t, *J* = 7.6, 2H), 7.16 (t, *J* = 7.6, 1H), 5.80 (br s, 1H, *NH*), 4.77 (s, 2H), 4.29 (d, *J* = 5.3, 2H), 3.64 (s, 2H).

^{13}C NMR ($CDCl_3$, 150 MHz): δ = 201.2, 162.7, 154.7, 137.2, 129.1, 125.0, 120.3, 95.2, 74.8, 51.3, 47.6.

HRMS (ES+): *m/z* $[M+Na]^+$ calcd for $C_{13}H_{13}Cl_3N_2NaO_4^+$: 388.9833; found: 388.9835.

(1-Methyl-2-oxo-3-phenylcarbamoyl-propyl)-carbamic acid ethyl ester (5c)

Yield: 125 mg (45%); White solid; R_f = 0.60 (Et_2O); mp 100 – 102 °C.

IR (KBr): 3305, 1692, 1651, 1602, 1546 cm^{-1}.

^{1}H NMR ($CDCl_3$, 600 MHz): δ = 8.97 (s, 1H, *NH*), 7.55 (d, *J* = 7.6, 2H), 7.33 (t, *J* = 7.6, 2H), 7.14 (t, *J* = 7.6, 1H), 5.43 (br d, *J* = 5.9, 1H, *NH*), 4.41 (m, 1H), 4.15 (m, 2H), 3.70 (AB quartet, ^{2}J = 17.0, 2H), 1.42 (d, *J* = 7.0, 3H), 1.27 (t, *J* = 7.0, 3H).

^{13}C NMR ($CDCl_3$, 150 MHz): δ = 206.0, 163.4, 156.3, 137.5, 129.0, 124.7, 120.2, 61.6, 56.4, 46.5, 16.6, 14.6.

HRMS (ES+): *m/z* $[M+Na]^+$ calcd for $C_{14}H_{18}N_2NaO_4^+$: 301.1159; found: 301.1160.

(1-Methyl-2-oxo-3-phenylcarbamoyl-propyl)-carbamic acid 2,2,2-trichloro-ethyl ester (5d)

Yield: 237 mg (62%); White solid; R_f = 0.30 (Et_2O/Petrol, 2:1); mp 99 – 101 °C.

IR (KBr): 3332, 1711, 1664, 1603, 1556 cm^{-1}.

^1H NMR ($CDCl_3$, 600 MHz): δ = 8.77 (s, 1H, *NH*), 7.54 (d, *J* = 7.6, 2H), 7.35 (t, *J* = 7.6, 2H), 7.15 (t, *J* = 7.6, 1H), 5.81 (d, *J* = 6.5, 1H, *NH*), 4.76 (AB quartet, ^2J = 11.7, 2H), 4.50 (m, 1H), 3.71 (AB quartet, ^2J = 17.0, 2H), 1.49 (d, *J* = 7.6, 3H).

^{13}C NMR ($CDCl_3$, 150 MHz): δ = 205.1, 162.9, 154.2, 137.3, 129.1, 124.9, 120.2, 95.2, 74.7, 56.7, 46.6, 16.7.

HRMS (ES+): *m/z* $[M+Na]^+$ calcd for $C_{14}H_{15}Cl_3N_2NaO_4^+$: 402.9990; found: 402.9997.

(1-Benzyl-2-oxo-3-phenylcarbamoyl-propyl)-carbamic acid ethyl ester (5e)

Yield: 248 mg (70%); White solid; R_f = 0.65 (Et_2O); mp 139 – 141 °C.

IR (KBr): 3323, 2995, 1727, 1684, 1604, 1543 cm^{-1}.

^1H NMR ($CDCl_3$, 600 MHz): δ = 8.86 (s, 1H, *NH*), 7.54 (d, *J* = 7.6, 2H), 7.29 – 7.36 (m, 5H), 7.19 (d, *J* = 7.0, 2H), 7.14 (t, *J* = 7.6, 1H), 5.32 (d, *J* = 6.5, 1H, *NH*), 4.58 (m, 1H), 4.12 (m, 2H), 3.70 (d, ^2J = 17.0, 1H), 3.56 (d, ^2J = 17.0, 1H), 3.18 (dd, ^2J = 14.0, ^3J = 6.5, 1H), 3.00 (dd, ^2J = 14.0, ^3J = 7.6, 1H), 1.24 (t, *J* = 7.0, 3H).

^{13}C NMR ($CDCl_3$, 150 MHz): δ = 205.5, 163.2, 156.4, 137.6, 135.4, 129.4, 129.1, 129.0, 127.5, 124.6, 120.2, 61.7, 47.5, 36.7, 14.5.

HRMS (ES+): *m/z* $[M+Na]^+$ calcd for $C_{20}H_{22}N_2NaO_4^+$: 377.1472; found: 377.1474.

(1-Benzyl-2-oxo-3-phenylcarbamoyl-propyl)-carbamic acid 2,2,2-trichloro-ethyl ester (5f)

Yield: 247 mg (54%); White solid; R_f = 0.50 (Et_2O/Petrol, 2:1); mp 51 – 53 °C.

IR (KBr): 3335, 3033, 2965, 1744, 1604, 1554 cm^{-1}.

^1H NMR ($CDCl_3$, 600 MHz): δ = 8.65 (s, 1H, *NH*), 7.52 (d, *J* = 7.6, 2H), 7.29 – 7.37 (m, 5H), 7.19 (d, *J* = 7.0, 2H), 7.16 (t, *J* = 7.6, 1H), 5.75 (d, *J* = 7.0, 1H, *NH*), 4.73 (AB quartet, ^2J = 11.7, 2H), 4.70 (m, 1H), 3.70 (d, ^2J = 17.0, 1H), 3.58 (d, ^2J = 17.0, 1H), 3.26 (dd, ^2J = 14.1, ^3J = 5.9, 1H), 3.07 (dd, ^2J = 14.1, ^3J = 7.6, 1H).

^{13}C NMR ($CDCl_3$, 150 MHz): δ = 204.6, 162.8, 154.3, 137.3, 135.0, 129.2, 129.1, 127.6, 124.8, 120.2, 95.2, 74.7, 61.9, 47.7, 36.8.

HRMS (ES+): *m/z* $[M+Na]^+$ calcd for $C_{20}H_{19}Cl_3N_2NaO4^+$: 479.0303; found: 479.0309.

Methyl-(2-oxo-3-phenylcarbamoyl-propyl)-carbamic acid ethyl ester (5g)

Yield: 264 mg (95%); Oil; R_f = 0.40 (Et_2O).

IR (KBr): 3300, 3215, 3145, 3105, 1715, 1660, 1608 cm^{-1}.

^1H NMR ($CDCl_3$, 600 MHz): δ = 8.99, 8.91 (s, 1H, *NH*), 7.55 (m, 2H), 7.33 (m, 2H), 7.13 (m, 1H), 4.20 (s, 2H), 4.18, 4.12 (q, *J* = 7.0, 2H), 3.60, 3.57 (s, 2H), 3.01, 2.99 (s, 3H), 1.31, 1.19 (t, *J* = 7.0, 3H).

^{13}C NMR ($CDCl_3$, 150 MHz): δ = 202.5, 163.2, 157.2, 137.6, 129.0, 124.6, 120.2, 62.2/62.0, 59.1/58.8, 47.2/46.9, 36.1/35.8, 14.6.

HRMS (ES+): *m/z* $[M+Na]^+$ calcd for $C_{14}H_{18}N_2NaO_4^+$: 301.1159; found: 301.1155.

Methyl-(2-oxo-3-phenylcarbamoyl-propyl)-carbamic acid 2,2,2-trichloro-ethyl ester (5h)

Yield: 343 mg (90%); Oil; R_f = 0.60 (Et_2O).

IR (KBr): 3380, 1720, 1605, 1554 cm^{-1}.

^{1}H NMR ($CDCl_3$, 600 MHz): δ = 8.78, 8.76 (s, 1H, *NH*), 7.54 (m, 2H), 7.35 (m, 2H), 7.16 (m, 1H), 4.79, 4.72 (s, 2H), 4.31, 4.29 (s, 2H), 3.61, 3.60 (s, 2H), 3.11, 3.08 (s, 3H).

^{13}C NMR ($CDCl_3$, 150 MHz): δ = 201.4, 155.3, 137.2, 129.1, 124.8, 120.2, 95.3, 75.5/75.2, 59.3/58.8, 47.3/47.1, 36.7/36.0.

HRMS (ES+): *m/z* $[M+Na]^+$ calcd for $C_{14}H_{15}Cl_3N_2NaO_4^+$: 402.9990; found: 402.9993.

(2-Oxo-3-phenylcarbamoyl-propyl)-phenyl-carbamic acid ethyl ester (5i)

Yield: 290 mg (85%); White solid; R_f = 0.60 (Et_2O); mp 89 – 91 °C.

IR (KBr): 3304, 3209, 3151, 3103, 1711, 1663, 1600, 1560 cm^{-1}.

^{1}H NMR (DMSO-d_6, 600 MHz): δ = 10.15 (s, 1H, *NH*), 7.55 (d, *J* = 7.6, 2H), 7.36 – 7.20 (m, 7H), 7.06 (t, *J* = 7.6, 1H), 4.65 (s, 2H), 4.04 (q, *J* = 7.0, 2H), 3.62 (s, 2H), 1.10 (t, *J* = 7.0, 3H).

^{13}C NMR (DMSO-d_6, 150 MHz): δ = 200.7, 164.8, 155.1, 139.2, 129.3, 129.2, 126.6, 124.0, 119.6, 61.9, 59.9, 49.3, 14.8.

HRMS (ES+): *m/z* $[M+Na]^+$ calcd for $C_{19}H_{20}N_2NaO_4^+$: 363.1315; found: 363.1315.

4-(1,3-Dioxo-1,3-dihydroisoindol-2-yl)-3-oxo-*N*-phenyl-butyramide (5j)

Yield: 290 mg (90%); White solid; R_f = 0.60 (Et_2O); mp 185 – 187 °C.

IR (KBr): 3320, 1770, 1710, 1700, 1605, 1540 cm^{-1}.

^{1}H NMR (DMSO-d_6, 600 MHz): δ = 10.21 (s, 1H, *NH*), 7.93 – 7.87 (m, 4H) 7.56 (m, 2H), 7.31 (m, 2H), 7.07 (m, 1H), 4.74 (s, 2H), 3.77 (s, 2H).

^{13}C NMR (DMSO-d_6, 150 MHz): δ = 198.6, 167.7, 164.5, 139.2, 135.3, 131.9, 129.3, 124.1, 123.8, 119.6, 49.5, 47.4.

HRMS (ES+): *m/z* $[M+Na]^+$ calcd for $C_{18}H_{14}N_2NaO_4^+$: 345.0846; found: 345.0851.

4-(1,3-Dioxo-1,3-dihydroisoindol-2-yl)-3-oxo-pentanoic acid phenylamide (5k)

Yield: 293 mg (87%); White solid; R_f = 0.65 (Et_2O); mp 125 – 127 °C.

IR (KBr): 3321, 1779, 1711, 1700, 1603, 1542 cm^{-1}.

^{1}H NMR ($CDCl_3$, 600 MHz): δ = 8.94 (s, 1H, *NH*), 7.89 (m, 2H), 7.78 (m, 2H), 7.52 (m, 2H), 7.33 (m, 2H), 7.13 (m, 1H), 4.98 (q, *J* = 7.0, 1H), 3.66 (s, 2H), 1.69 (d, *J* = 7.0, 3H).

^{13}C NMR ($CDCl_3$, 150 MHz): δ = 202.5, 167.5, 162.7, 137.4, 134.6, 131.7, 129.0, 124.7, 123.8, 120.3, 54.8, 45.9, 14.2.

HRMS (ES+): *m/z* $[M+Na]^+$ calcd for $C_{19}H_{16}N_2NaO_4^+$: 359.1002; found: 359.1004.

4-(1,3-Dioxo-1,3-dihydroisoindol-2-yl)-3-oxo-5-phenyl-pentanoic acid phenylamide (5l)

Yield: 350 mg (85%); White solid; R_f = 0.35 (Et_2O/Petrol, 2:1); mp 154 – 156 °C.

IR (KBr): 3318, 1775, 1710, 1698, 1600, 1545 cm^{-1}.

^{1}H NMR ($CDCl_3$, 600 MHz): δ = 8.89 (s, 1H, *NH*), 7.82 – 7.79 (m, 2H), 7.75 – 7.73 (m, 2H), 7.53 (d, *J* = 7.6, 2H), 7.34 (t, *J* = 7.6, 2H), 7.21 – 7.13 (m, 6H), 5.18 (dd, *J* = 5.3, *J* = 10.6, 1H), 3.67 (AB quartet, ^{2}J = 17.6, 2H), 3.63 (dd, ^{2}J = 14.1, ^{3}J = 5.3, 1H), 3.39 (dd, ^{2}J = 14.1, ^{3}J = 10.6, 1H).

^{13}C NMR ($CDCl_3$, 150 MHz): δ = 201.8, 167.5, 162.6, 137.3, 136.1, 134.6, 131.3, 129.0, 128.9, 128.7, 127.1, 124.7, 123.77, 120.3, 60.6, 46.5, 33.7.

HRMS (ES+): *m/z* $[M+Na]^+$ calcd for $C_{25}H_{20}N_2NaO_4^+$: 435.1315; found: 435.1310.

(3-Oxo-4-phenylcarbamoyl-butyl)-carbamic acid ethyl ester (11a)

Yield: 264 mg (95%); White solid; R_f = 0.35 (Et_2O); mp 85 – 86 °C.

IR (KBr): 3330, 3304, 3204, 3146, 1718, 1695, 1662; 1621, 1601 cm^{-1}.

^{1}H NMR ($CDCl_3$, 600 MHz): δ = 8.84 (s, 1H, *NH*), 7.46 (d, *J* = 7.6, 2H), 7.25 (t, *J* = 7.6, 2H), 7.05 (t, *J* = 7.6, 1H), 5.07 (br s, 1H, *NH*), 4.03 (q, *J* = 7.0, 2H), 3.51 (s, 2H), 3.39 (m, 2H), 2.74 (t, *J* = 5.9, 2H), 1.15 (t, *J* = 7.0, 3H).

^{13}C NMR ($CDCl_3$, 150 MHz): δ = 205.9, 163.4, 156.8, 137.6, 129.0, 124.6, 120.1, 61.1, 49.9, 44.0, 35.6, 14.6.

HRMS (ES+): *m/z* $[M+Na]^+$ calcd for $C_{14}H_{18}N_2NaO_4^+$: 301.1159; found: 301.1161.

(4-Oxo-5-phenylcarbamoyl-pentyl)-carbamic acid ethyl ester (11b)

Yield: 272 mg (93%); White solid; R_f = 0.50 (Et_2O); mp 104 – 105 °C.

IR (KBr): 3291, 3247, 3194, 1717, 1686, 1664, 1599 cm^{-1}.

HRMS (ES+): *m/z* $[M+Na]^+$ calcd for $C_{15}H_{20}N_2NaO_4^+$: 315.1315; found: 315.1315.

(4-Oxo-5-phenylcarbamoyl-pentyl)-carbamic acid benzyl ester (11c)

Yield: 262 mg (74%); White solid; R_f = 0.30 (Et_2O); mp 87 – 89 °C.

IR (KBr): 3338, 3152, 3072, 2962, 1729, 1714, 1690, 1663, 1603 cm^{-1}.

HRMS (ES+): *m/z* $[M+Na]^+$ calcd for $C_{20}H_{22}N_2NaO_4^+$: 377.1472; found: 377.1475.

(5-Oxo-6-phenylcarbamoyl-hexyl)-carbamic acid benzyl ester (11d)

Yield: 260 mg (71%); White solid; R_f = 0.40 (Et_2O); mp 110 – 112 °C.

IR (KBr): 3340, 3150, 2980, 1725, 1715, 1693, 1660, 1604 cm^{-1}.

^{1}H NMR ($CDCl_3$, 600 MHz): δ = 9.01 (s, 1H, *NH*), 7.46 (d, *J* = 7.6, 2H), 7.28 – 7.23 (m, 7H), 7.04 (t, *J* = 7.6, 1H), 5.02 (s, 2H), 4.82 (br s, 1H, *NH*), 3.46 (s, 2H), 3.12 (m, 2H), 2.54 (t, *J* = 7.0, 2H), 1.57 (p, *J* = 7.0, 2H), 1.45 (p, *J* = 7.0, 2H).

^{13}C NMR ($CDCl_3$, 150 MHz): δ = 207.0, 163.5, 156.5, 137.6, 129.0, 128.6, 128.2, 128.1, 124.6, 120.1, 66.7, 49.4, 43.3, 40.5, 29.2, 20.2.

HRMS (ES+): *m/z* $[M+Na]^+$ calcd for $C_{21}H_{24}N_2NaO_4^+$: 391.1628; found: 391.1629.

(6-Oxo-7-phenylcarbamoyl-heptyl)-carbamic acid benzyl ester (11e)

Yield: 260 mg (68%); White solid; R_f = 0.47 (Et_2O); mp 83 – 84 °C.

IR (KBr): 3320, 3305, 3178, 2950, 2893, 1714, 1689, 1662, 1607 cm^{-1}.

^{1}H NMR ($CDCl_3$, 600 MHz): δ = 9.14 (s, 1H, *NH*), 7.56 (d, *J* = 7.6, 2H), 7.38 – 7.33 (m, 7H), 7.14 (t, *J* = 7.6, 1H), 5.11 (s, 2H), 4.85 (br s, 1H, *NH*), 3.56 (s, 2H), 3.21 (m, 2H), 2.60 (t, *J* = 7.0, 2H), 1.65 (p, *J* = 7.0, 2H), 1.54 (p, *J* = 7.0, 2H), 1.35 (p, *J* = 7.0, 2H).

^{13}C NMR ($CDCl_3$, 150 MHz): δ = 207.3, 163.5, 156.5, 137.6, 136.6, 129.0, 128.6, 128.2, 128.1, 124.6, 120.1, 66.7, 49.2, 43.8, 40.7, 29.8, 26.0, 22.8.

HRMS (ES+): *m/z* $[M+Na]^+$ calcd for $C_{22}H_{26}N_2NaO_4^+$: 405.1785; found: 405.1788.

(4-Oxo-5-phenylcarbamoyl-pentyl)-carbamic acid 2,2,2-trichloro-ethyl ester (11f)

Yield: 356 mg (90%); White solid; R_f = 0.57 (Et_2O); mp 114 – 116 °C.

IR (KBr): 3331, 3237, 3150, 3098, 2952, 1727, 1700, 1663, 1607 cm^{-1}.

HRMS (ES+): *m/z* $[M+Na]^+$ calcd for $C_{15}H_{17}Cl_3N_2NaO_4^+$: 417.0146; found: 417.0150.

(5-Oxo-6-phenylcarbamoyl-hexyl)-carbamic acid 2,2,2-trichloro-ethyl ester (11g)

Yield: 246 mg (60%); White solid; R_f = 0.60 (Et_2O); mp 67 – 69 °C.

IR (KBr): 3330, 3232, 3143, 3088, 2944, 1727, 1709, 1669, 1606 cm^{-1}.

^1H NMR ($CDCl_3$, 600 MHz): δ = 9.05 (s, 1H, *NH*), 7.55 (d, *J* = 7.6, 2H), 7.35 (t, *J* = 7.6, 2H), 7.14 (t, *J* = 7.6, 1H), 5.16 (br s, 1H, *NH*), 4.74 (s, 2H), 3.58 (s, 2H), 3.26 (m, 2H), 2.66 (t, *J* = 7.0, 2H), 1.69 (p, *J* = 7.0, 2H), 1.58 (p, *J* = 7.0, 2H).

^{13}C NMR ($CDCl_3$, 150 MHz): δ = 207.0, 163.5, 154.7, 137.5, 129.1, 124.7, 120.1, 95.6, 74.5, 49.4, 43.3, 40.7, 29.0, 20.1.

HRMS (ES+): *m/z* $[M+Na]^+$ calcd for $C_{16}H_{19}Cl_3N_2NaO_4^+$: 431.0303; found: 431.0304.

(6-Oxo-7-phenylcarbamoyl-heptyl)-carbamic acid 2,2,2-trichloro-ethyl ester (11h)

Yield: 309 mg (73%); White solid; R_f = 0.65 (Et_2O); mp 82 – 84 °C.

IR (KBr): 3333, 3230, 3140, 3091, 2880, 1723, 1712, 1670, 1604 cm^{-1}.

^1H NMR ($CDCl_3$, 600 MHz): δ = 9.11 (s, 1H, *NH*), 7.56 (d, *J* = 7.6, 2H), 7.35 (t, *J* = 7.6, 2H), 7.14 (t, *J* = 7.6, 1H), 5.09 (br s, 1H, *NH*), 4.74 (s, 2H), 3.58 (s, 2H), 3.26 (m, 2H), 2.62 (t, *J* = 7.0, 2H), 1.67 (p, *J* = 7.0, 2H), 1.58 (p, *J* = 7.0, 2H), 1.38 (p, *J* = 7.0, 2H).

^{13}C NMR ($CDCl_3$, 150 MHz): δ = 207.3, 163.5, 154.7, 137.5, 129.1, 124.6, 120.1, 95.7, 74.5, 49.2, 43.8, 40.9, 29.6, 25.9, 22.8.

HRMS (ES+): *m/z* $[M+Na]^+$ calcd for $C_{17}H_{21}Cl_3N_2NaO_4^+$: 445.0459; found: 445.0463.

(4-Carbamoyl-3-oxo-butyl)-carbamic acid benzyl ester (11i)

Yield: 160 mg (60%); White solid; R_f = 0.50 (Et_2O/CH_3OH, 10:1); mp 105 – 107 °C.

IR (KBr): 3400, 3342, 3218, 2955, 1709, 1692, 1658, 1547 cm^{-1}.

^1H NMR (DMSO-d_6, 600 MHz): δ = 7.49 (br s, 1H, *NH*), 7.37 – 7.29 (m, 5H), 7.23 (br t, *J* = 5.3, 1H, *NH*), 7.08 (br s, 1H, *NH*), 5.00 (s, 2H), 3.28 (s, 2H), 3.18 (m, 2H), 2.69 (t, *J* = 7.0, 2H).

^{13}C NMR (DMSO-d_6, 150 MHz): δ = 204.3, 168.6, 156.5, 137.6, 128.8, 128.3, 128.2, 65.7, 50.9, 42.8, 35.8.

HRMS (ES+): *m/z* $[M+Na]^+$ calcd for $C_{13}H_{16}N_2NaO_4^+$: 287.1002; found: 287.1002.

(5-Carbamoyl-4-oxo-pentyl)-carbamic acid benzyl ester (11j)

Yield: 162 mg (58%); White solid; R_f = 0.27 (Et_2O/CH_3OH, 20:1); mp 96 – 98 °C.

IR (KBr): 3358, 3246, 3183, 3033, 2923, 1778, 1718, 1693, 1604 cm^{-1}.

HRMS (ES+): *m/z* $[M+Na]^+$ calcd for $C_{14}H_{18}N_2NaO_4^+$: 301.1159; found: 301.1162.

(6-Carbamoyl-5-oxo-hexyl)-carbamic acid benzyl ester (11k)

Yield: 175 mg (60%); White solid; R_f = 0.43 (Et_2O/CH_3OH, 10:1); mp 117 – 119 °C.

IR (KBr): 3396, 3351, 3206, 3033, 2950, 2885, 1712, 1690, 1651 cm^{-1}.

^1H NMR (DMSO-d_6, 600 MHz): δ = 7.48 (br s, 1H, *NH*), 7.37 – 7.25 (m, 5H), 7.26 (br t, *J* = 5.9, 1H, *NH*), 7.05 (br s, 1H, *NH*), 5.00 (s, 2H), 3.24 (s, 2H), 2.97 (m, 2H), 2.51 (m, 2H, dmso overlap), 1.43 (m, 2H), 1.35 (m, 2H).

^{13}C NMR (DMSO-d_6, 150 MHz): δ = 205.4, 168.7, 156.6, 137.8, 128.8, 128.2, 65.6, 50.8, 42.2, 40.5, 29.3, 20.7.

HRMS (ES+): *m/z* $[M+Na]^+$ calcd for $C_{15}H_{20}N_2NaO_4^+$: 315.1315; found: 315.1315.

(7-Carbamoyl-6-oxo-heptyl)-carbamic acid benzyl ester (11l)

Yield: 168 mg (55%); White solid; R_f = 0.40 (Et_2O/CH_3OH, 10:1); mp 89 – 90 °C.

IR (KBr): 3390, 3340, 3212, 3030, 2948, 2889, 1715, 1690, 1645 cm^{-1}.

^{1}H NMR (DMSO-d_6, 600 MHz): δ = 7.48 (br s, 1H, *NH*), 7.38 – 7.30 (m, 5H), 7.24 (br t, *J* = 5.9, 1H, *NH*), 7.05 (br s, 1H, *NH*), 5.00 (s, 2H), 3.25 (s, 2H), 2.97 (m, 2H), 2.49 (m, 2H, dmso overlap), 1.42 (m, 2H), 1.39 (m, 2H), 1.20 (m, 2H).

^{13}C NMR (DMSO-d_6, 150 MHz): δ = 205.5, 168.7, 156.6, 137.8, 128.8, 128.2, 65.6, 50.8, 42.5, 40.6, 29.7, 26.2, 23.1.

HRMS (ES+): *m/z* $[M+Na]^+$ calcd for $C_{16}H_{22}N_2NaO_4^+$: 329.1472; found: 329.1476.

Derivatisation of β-keto amides 11b,c,f,j to β-hydroxy amides 12

To the corresponding β-keto amide **11** (100 mg) in methanol (10 mL) was added $NaBH_4$ in small portions (5–7 mg every 10 min) until TLC indicated absence of starting material. The mixture was then diluted with water (50 mL) and extracted with CH_2Cl_2 (3 × 20 mL). The organic layers were combined, dried over Na_2SO_4 and the solvent was removed on a rotary evaporator to afford practically pure hydroxy amides **12**.

(4-Hydroxy-5-phenylcarbamoyl-pentyl)-carbamic acid ethyl ester (12b)

Yield: 91 mg (90%); White solid; R_f = 0.25 (Et_2O); mp 116 – 117 °C.

IR (KBr): 3325, 3301, 1689, 1664, 1600 cm^{-1}.

^{1}H NMR (DMSO-d_6, 600 MHz): δ = 9.85 (s, 1H, *NH*), 7.59 (d, *J* = 7.6, 2H), 7.28, (t, *J* = 7.6, 2H), 7.07 (t, *J* = 5.3, 1H, *NH*), 7.01 (t, *J* = 7.6, 1H), 4.74 (d, *J* = 5.9, 1H, *OH*), 3.95 (q, *J* = 7.0, 2H), 3.89 (m, 1H), 2.95 (m, 2H), 2.36 (m, 2H), 1.56 – 1.32 (m, 4H), 1.13 (t, *J* = 7.0, 3H).

^{13}C NMR (DMSO-d_6, 150 MHz): δ = 170.3, 156.7, 139.8, 129.1, 123.4, 119.5, 67.7, 59.9, 45.5, 40.8, 34.8, 26.4, 15.2.

HRMS (ES+): *m/z* $[M+Na]^+$ calcd for $C_{15}H_{22}N_2NaO_4^+$: 317.1472; found: 317.1472.

(4-Hydroxy-5-phenylcarbamoyl-pentyl)-carbamic acid benzyl ester (12c)

Yield: 88 mg (88%); White solid; R_f = 0.70 (Et_2O/CH_3OH, 20:1); mp 163 – 165 °C.

^{1}H NMR (DMSO-d_6, 600 MHz): δ = 9.86 (s, 1H, *NH*), 7.60 (d, *J* = 7.6, 2H), 7.37 – 7.26 (m, 7H), 7.01 (t, *J* = 7.6, 1H), 5.00 (s, 2H), 4.77 (d, *J* = 5.3, 1H, *OH*), 3.91 (m, 1H), 3.00 (m, 2H), 2.37 (m, 2H), 1.56 – 1.34 (m, 4H).

^{13}C NMR (DMSO-d_6, 150 MHz): δ = 170.3, 156.6, 139.8, 137.8, 129.1, 128.8, 128.2, 123.5, 119.5, 67.7, 65.6, 45.5, 40.9, 34.8, 26.3.

HRMS (ES+): *m/z* $[M+Na]^+$ calcd for $C_{20}H_{24}N_2NaO_4^+$: 379.1628; found: 379.1632.

(4-Hydroxy-5-phenylcarbamoyl-pentyl)-carbamic acid 2,2,2-trichloro-ethyl ester (12f)

Yield: 92 mg (92%); White solid; R_f = 0.23 (Et_2O); mp 89 – 90 °C.

^{1}H NMR (DMSO-d_6, 600 MHz): δ = 9.86 (s, 1H, *NH*), 7.70 (t, *J* = 5.9, 1H, *NH*), 7.59 (d, *J* = 7.6, 2H), 7.28, (t, *J* = 7.6, 2H), 7.01 (t, *J* = 7.6, 1H), 4.77 (s, 2H), 4.76 (d, *J* = 5.3, 1H, *OH*), 3.90 (m, 1H), 3.02 (m, 2H), 2.36 (m, 2H), 1.62 – 1.32 (m, 4H).

^{13}C NMR (DMSO-d_6, 150 MHz): δ = 170.3, 154.9, 139.8, 129.1, 123.4, 119.5, 96.8, 73.7, 67.7, 45.5, 41.1, 34.7, 26.1.

HRMS (ES+): *m/z* $[M+Na]^+$ calcd for $C_{15}H_{19}Cl_3N_2NaO_4^+$: 419.0303; found: 419.0301.

(5-Carbamoyl-4-hydroxy-pentyl)-carbamic acid benzyl ester (12j)

Yield: 85 mg (84%); White solid; R_f = 0.25 (Et_2O/CH_3OH, 10:1); mp 105 – 107 °C.

^{1}H NMR (DMSO-d_6, 600 MHz): δ = 7.37 – 7.30 (m, 5H), 7.27 (br s, 1H, *NH*), 7.25 (t, *J* = 5.9, 1H, *NH*), 6.81 (br s, 1H, *NH*), 5.00 (s, 2H), 4.65 (d, *J* = 4.7, 1H, *OH*), 3.76 (m, 1H), 2.97 (m, 2H), 2.11 (m, 2H), 1.55 – 1.25 (m, 4H).

^{13}C NMR (DMSO-d_6, 150 MHz): δ = 173.6, 156.6, 137.8, 128.8, 128.2, 128.1, 67.6, 65.5, 43.8, 40.9, 34.6, 26.3.

HRMS (ES+): *m/z* $[M+Na]^+$ calcd for $C_{14}H_{20}N_2NaO_4^+$: 303.1315; found: 303.1318.

Pyrrolin-4-ones (6)

These compounds were obtained as byproducts in the synthesis of β-keto amides **5a–f**, following the general procedure described above.

5-Methyl-3-oxo-4-phenylcarbamoyl-2,3-dihydropyrrole-1-carboxylic acid ethyl ester (6a)

Yield: 73 mg (25%); White solid; mp 178 – 179 °C.

IR (KBr): 3245, 3196, 1744, 1683, 1659, 1596, 1563 cm^{-1}.

^{1}H NMR ($CDCl_3$, 600 MHz): δ = 10.19 (s, 1H, *NH*), 7.55 (d, *J* = 7.6, 2H), 7.25 (t, *J* = 7.6, 2H), 7.02 (t, *J* = 7.6, 1H), 4.24 (q, *J* = 7.0, 2H), 4.20 (s, 2H), 3.12 (s, 3H), 1.30 (t, *J* = 7.0, 3H).

^{13}C NMR ($CDCl_3$, 150 MHz): δ = 195.2, 180.5, 160.6, 150.1, 138.2, 128.9, 124.0, 120.2, 113.2, 63.7, 56.3, 16.7, 14.3.

HRMS (ES+): *m/z* $[M+Na]^+$ calcd for $C_{15}H_{16}N_2NaO_4^+$: 311.1002; found: 311.1002.

5-Methyl-3-oxo-4-phenylcarbamoyl-2,3-dihydropyrrole-1-carboxylic acid 2,2,2-trichloro-ethyl ester (6b)

Yield: 82 mg (21%); White solid; R_f = 0.70 (Et_2O/Petrol, 2:1); mp 167 – 169 °C.

IR (KBr): 3251, 3200, 1745, 1690, 1660, 1602, 1560 cm^{-1}.

^{1}H NMR ($CDCl_3$, 600 MHz): δ = 10.21 (s, 1H, *NH*), 7.65 (d, *J* = 7.6, 2H), 7.36 (t, *J* = 7.6, 2H), 7.13 (t, *J* = 7.6, 1H), 4.89 (s, 2H), 4.41 (s, 2H), 3.25 (s, 3H).

^{13}C NMR ($CDCl_3$, 150 MHz): δ = 194.9, 179.8, 160.1, 148.4, 137.9, 129.0, 124.3, 120.3, 114.5, 94.1, 75.7, 56.0, 16.7.

HRMS (ES+): *m/z* $[M+Na]^+$ calcd for $C_{15}H_{13}Cl_3N_2NaO_4^+$: 412.9833; found: 412.9836.

2,5-Dimethyl-3-oxo-4-phenylcarbamoyl-2,3-dihydropyrrole-1-carboxylic acid ethyl ester (6c)

Yield: 157 mg (52%); White solid; R_f = 0.5 (Et_2O/Petrol, 2:1); mp 134 – 135 °C.

IR (KBr): 3335, 3286, 3232, 3118, 2991, 1729, 1696, 1662, 1601 cm^{-1}.

^{1}H NMR ($CDCl_3$, 600 MHz): δ = 10.22 (s, 1H, *NH*), 7.55 (d, *J* = 7.6, 2H), 7.25 (t, *J* = 7.6, 2H), 7.02 (t, *J* = 7.6, 1H), 4.33 – 4.23 ($\underline{AB}X_3$ m, 2H), 4.19 (q, *J* = 7.0, 1H), 3.12 (s, 3H), 1.49 (d, *J* = 7.0, 3H), 1.32 (t, *J* = 7.0, 3H).

^{13}C NMR ($CDCl_3$, 150 MHz): δ = 199.1, 179.6, 160.9, 150.3, 138.2, 128.9, 124.0, 120.2, 111.2, 63.6, 62.6, 17.3, 16.9, 14.3.

HRMS (ES+): *m/z* $[M+Na]^+$ calcd for $C_{16}H_{18}N_2NaO_4^+$: 325.1159; found: 325.1160.

2,5-Dimethyl-3-oxo-4-phenylcarbamoyl-2,3-dihydropyrrole-1-carboxylic acid 2,2,2-trichloro-ethyl ester (6d)

Yield: 81 mg (20%); White solid; R_f = 0.70 (Et_2O/Petrol, 2:1); mp 144 – 146 °C.

IR (KBr): 3330, 3290, 3228, 3120, 2985, 1732, 1698, 1660, 1604 cm^{-1}.

^{1}H NMR ($CDCl_3$, 600 MHz): δ = 10.24 (s, 1H, *NH*), 7.65 (d, *J* = 7.6, 2H), 7.36 (t, *J* = 7.6, 2H), 7.13 (t, *J* = 7.6, 1H), 5.01 (d, ^{2}J = 12.0, 1H), 4.85 (d, ^{2}J = 12.0, 1H), 4.40 (q, *J* = 7.0, 1H), 3.26 (s, 3H), 1.69 (d, *J* = 7.0, 3H).

^{13}C NMR ($CDCl_3$, 150 MHz): δ = 198.9, 179.1, 160.4, 148.8, 138.0, 129.0, 124.2, 120.3, 112.5, 94.0, 75.8, 62.7, 17.4, 17.0.

HRMS (ES+): *m/z* $[M+Na]^+$ calcd for $C_{16}H_{15}Cl_3N_2NaO_4^+$: 426.9990; found: 426.9995.

2-Benzyl-5-methyl-3-oxo-4-phenylcarbamoyl-2,3-dihydropyrrole-1-carboxylic acid ethyl ester (6e)

Yield: 75 mg (20%); White solid; R_f = 0.60 (Et_2O/Petrol, 2:1); mp 93 – 95 °C.

IR (KBr): 3308, 3168, 3088, 3033, 2982, 1750, 1687, 1654, 1600 cm^{-1}.

^1H NMR ($CDCl_3$, 600 MHz): δ = 10.31 (s, 1H, *NH*), 7.64 (d, *J* = 7.6, 2H), 7.35 (t, *J* = 7.6, 2H), 7.23 (m, 3H), 7.11 (t, *J* = 7.6, 1H), 7.02 (m, 2H), 4.54 (m, 1H), 4.42 (q, *J* = 7.0, 2H), 3.51 (dd, ^2J = 14.1, ^3J = 6.5, 1H), 3.40 (dd, ^2J = 14.1, ^3J = 2.9, 1H), 2.95 (s, 3H), 1.46 (t, *J* = 7.0, 3H).

^{13}C NMR ($CDCl_3$, 150 MHz): δ = 198.3, 180.6, 160.5, 150.3, 133.5, 129.2, 128.9, 128.5, 127.6, 124.0, 120.2, 112.8, 66.7, 63.7, 37.0, 16.5, 14.4.

HRMS (ES+): *m/z* $[M+Na]^+$ calcd for $C_{22}H_{22}N_2NaO_4^+$: 401.1472; found: 401.1472.

2-Benzyl-5-methyl-3-oxo-4-phenylcarbamoyl-2,3-dihydropyrrole-1-carboxylic acid 2,2,2-trichloro-ethyl ester (6f)

Yield: 96 mg (20%); White solid; R_f = 0.50 (Et_2O/Petrol, 2:1); mp 150 – 152 °C.

IR (KBr): 3300, 3260, 3242, 3030, 2978, 1745, 1690, 1650, 1605 cm^{-1}.

^1H NMR ($CDCl_3$, 600 MHz): δ = 10.12 (s, 1H, *NH*), 7.54 (d, *J* = 7.6, 2H), 7.26 (t, *J* = 7.6, 2H), 7.14 (m, 3H), 7.03 (t, *J* = 7.6, 1H), 6.96 (m, 2H), 4.88 (AB quartet, ^2J = 11.7, 2H), 4.55 (m, 1H), 3.56 (dd, ^2J = 14.1, ^3J = 6.5, 1H), 3.36 (dd, ^2J = 14.1, ^3J = 2.9, 1H), 2.88 (s, 3H).

^{13}C NMR ($CDCl_3$, 150 MHz): δ = 198.1, 180.0, 159.9, 148.8, 138.0, 133.1, 129.3, 129.0, 128.6, 127.4, 124.2, 120.3, 114.2, 94.0, 76.0, 66.7, 36.8, 29.7, 16.6.

HRMS (ES+): *m/z* $[M+Na]^+$ calcd for $C_{22}H_{19}Cl_3N_2NaO_4^+$: 503.0303; found: 503.0307.

References

1. Kofoed, T.; Hansen, H. F.; Ørum, H.; Koch, T. *J. Peptide Sci.* **2001**, *7*, 402 – 412.

2. (a) Carter, H. E.; Frank, R. L.; Johnston, H. W. *Organic Syntheses*, **1955**, Coll. Vol. 3, p.167 (b) Carson, J. F. *Synthesis* **1981**, 268-270. (c) Nefkens, G. H. L. *Nature* **1960**, 309. (d) Benoiton, N. L.; Chen, F. M. F. *Can. J. Chem.* **1987**, *65*, 1224-1227.

3. Not exceeding 5 min is particularly important for the phthaloyl-protected amino acids.

4. Gruzdev, D. A.; Levit, G. L.; Krasnov, V. P.; Chulakov, E. N.; Sadretdinova, L. Sh.; Grishakov, A. N.; Ezhikova, M. A.; Kodess, M. I.; Charushin, V. N. *Tetrahedron: Asymmetry* **2010**, *21*, 936–942.

5. An immediate filtration of the obtained mixture in case of **3j** led to the isolation of the unstable intermediate **8j**, which has low solubility in both CH_2Cl_2 and water. Its crude NMR is provided in Supporting Information File 2.
