# Supplementary material for: Synthesis of functionalised β-keto amides by aminoacylation/domino fragmentation of β-enamino amides
Source: Beilstein J Org Chem. 2018 Oct 10;14:2602–6. doi: 10.3762/bjoc.14.238 (PMC6204755; doi:10.3762/bjoc.14.238)
Supplement: File 2 — HPLC data and processed NMR spectra. [file Beilstein_J_Org_Chem-14-2602-s002.pdf]

Supporting Information File 2

for

Synthesis of functionalised β-keto amides by aminoacylation/domino fragmentation of β-enamino amides

Pavel Yanev and Plamen Angelov*

Address: Department of Organic Chemistry, University of Plovdiv Paisii Hilendarski, 24 Tsar Asen Str., 4000 Plovdiv, Bulgaria

Email: Plamen Angelov - angelov@uni-plovdiv.bg

*Corresponding author

HPLC data and processed NMR spectra

Table of contents

Chiral phase HPLC data....S2
Processed NMR spectra ($^1H/^{13}C$)
- Keto amides **5**....S8
- Keto amides **11**....S20
- Hydroxy amides **12**....S28
- Pyrrolinones **6**....S32
- Intermediate **8j**....S38
- Intermediates **3**....S40
- Intermediates **4**....S52
- Enamino amides **1**....S64

(Primary NMR data available at http://doi.org/10.5281/zenodo.1419645)

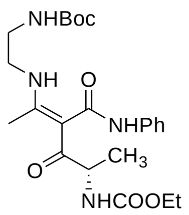

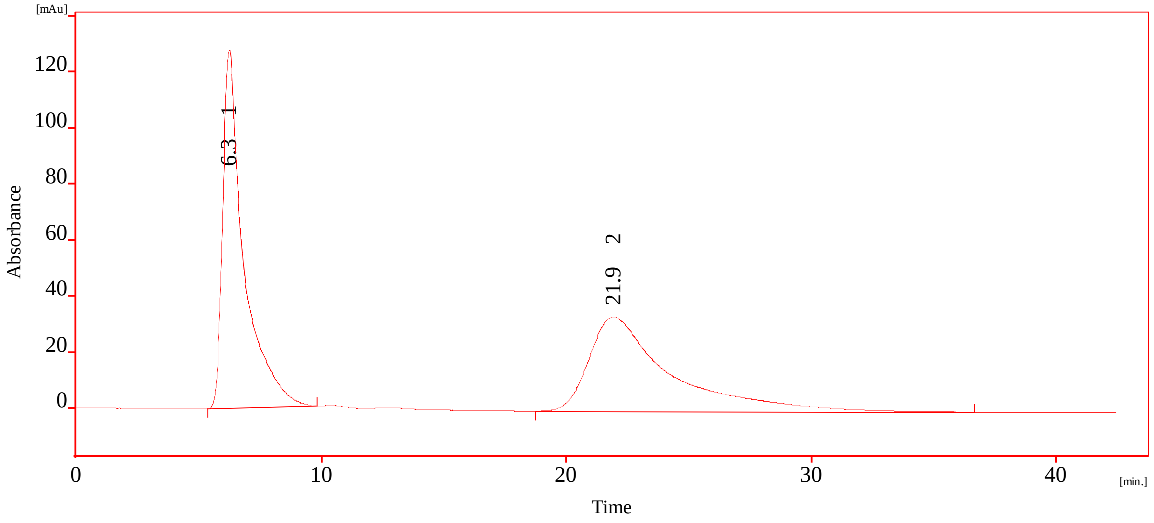

Column: Lux Amylose-1 (250×4.6)
Eluent: Hexane/Isopropanol 4:1
Flow: 1 mL/min
Detection: 310 nm

3c, racemic

Reten. Time [min]	Area [%]
6.25	49.7
21.93	50.3

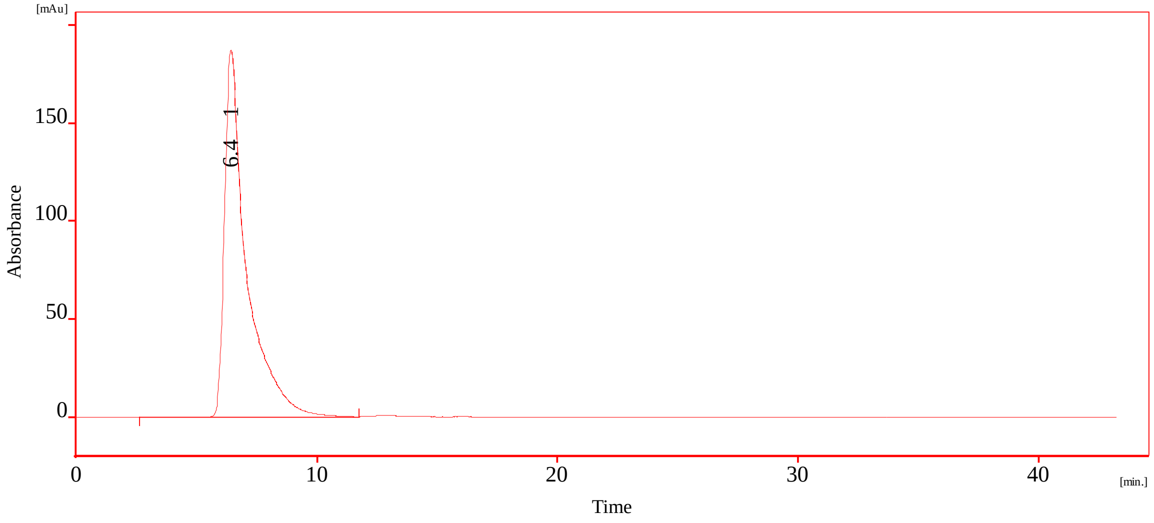

Column: Lux Amylose-1 (250×4.6)
Eluent: Hexane/Isopropanol 4:1
Flow: 1 mL/min
Detection: 310 nm

3c (L)

Reten. Time [min]	Area [%]
6.43	100

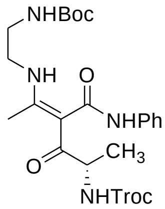

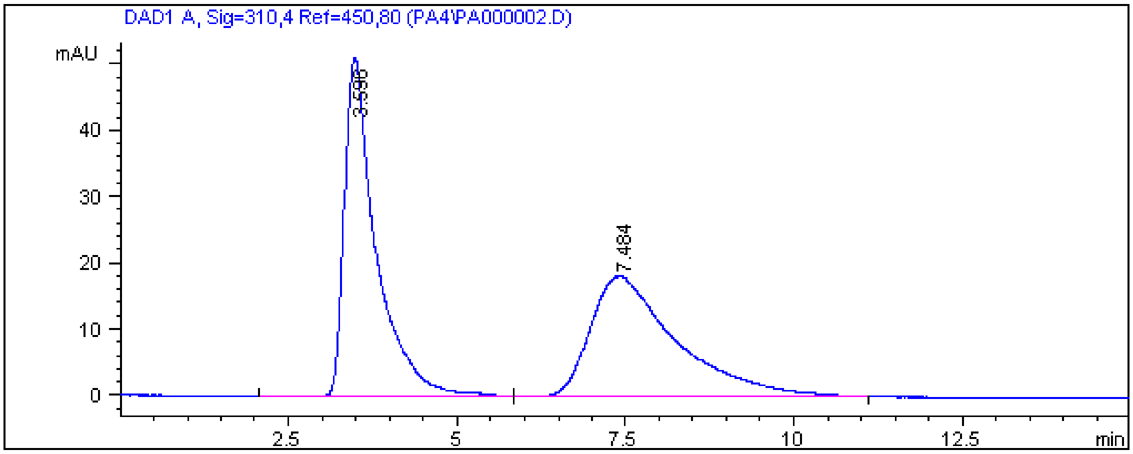

Column: Lux Amylose-1 (250×4.6)
Eluent: Hexane/Ethanol 1:1
Flow: 1 mL/min
Detection: 310 nm

3d, racemic

Reten. Time [min]	Area [%]
3.60	50.8
7.48	49.2

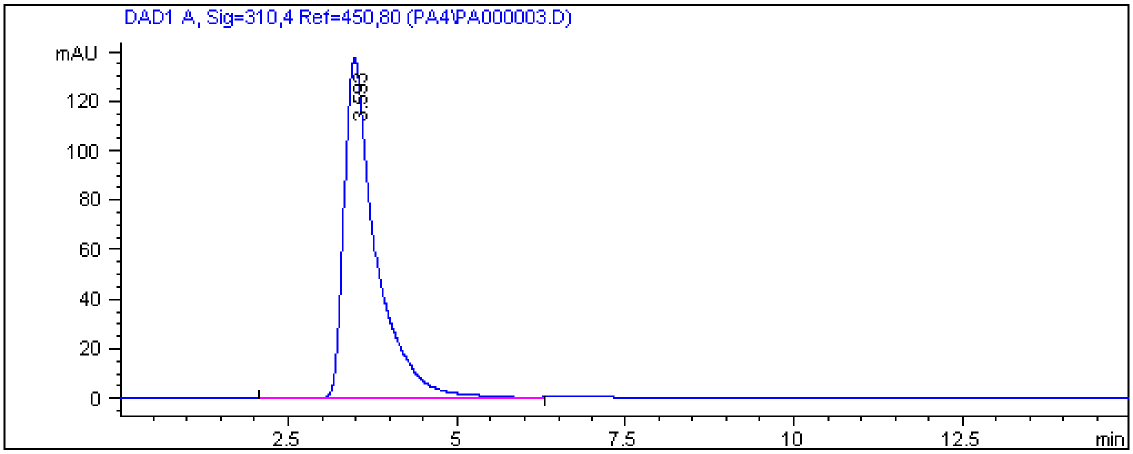

Column: Lux Amylose-1 (250×4.6)
Eluent: Hexane/Ethanol 1:1
Flow: 1 mL/min
Detection: 310 nm

3d (L)

Reten. Time [min]	Area [%]
3.59	100

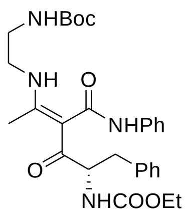

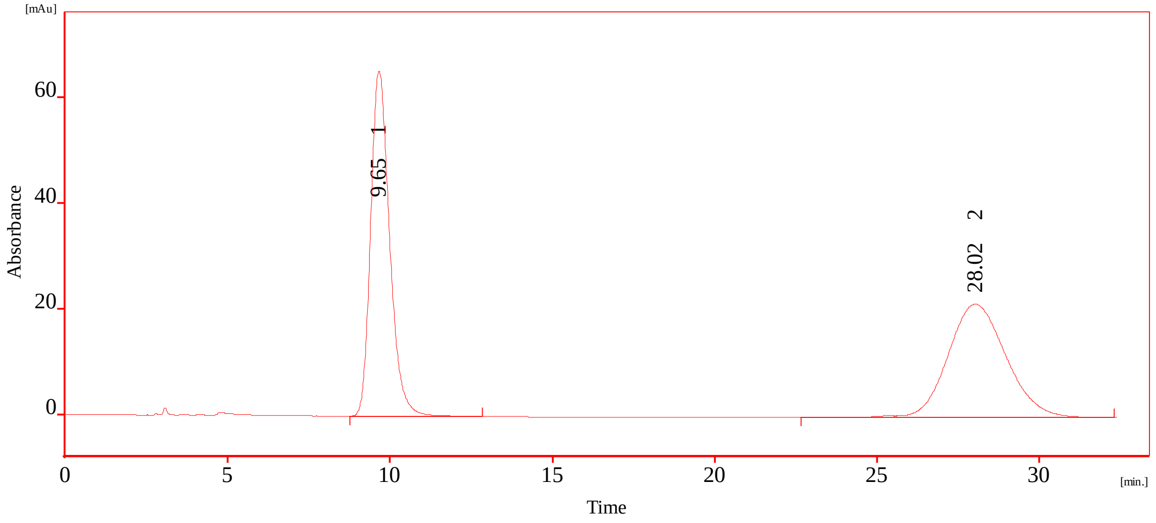

Column: Lux Amylose-1 (250×4.6)
Eluent: Hexane/Isopropanol 4:1
Flow: 1 mL/min
Detection: 310 nm

3e, racemic

Reten. Time [min]	Area [%]
9.65	49.9
28.02	50.1

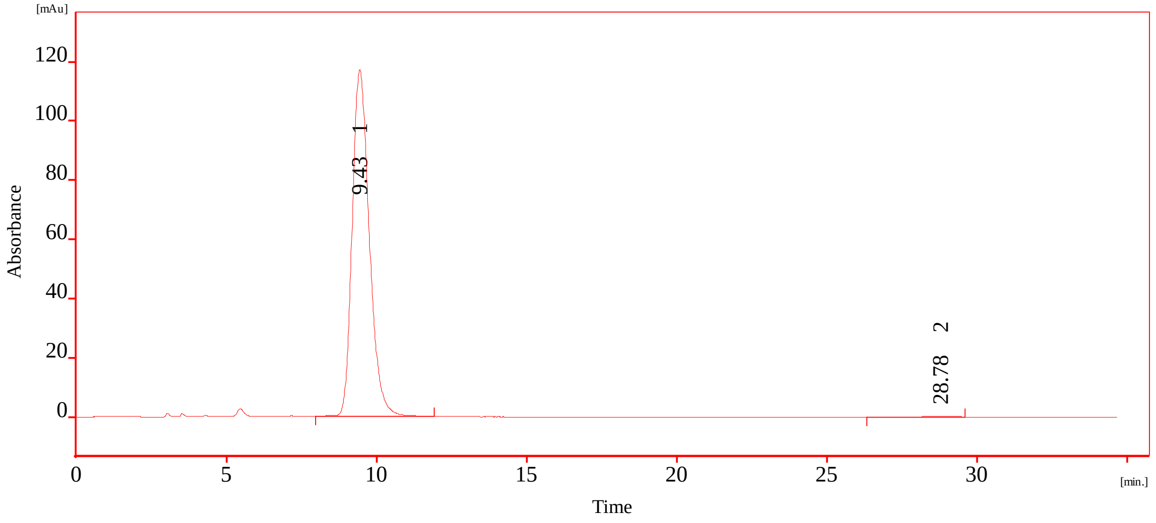

Column: Lux Amylose-1 (250×4.6)
Eluent: Hexane/Isopropanol 4:1
Flow: 1 mL/min
Detection: 310 nm

3e (L)

Reten. Time [min]	Area [%]
9.43	99.7
28.78	0.3

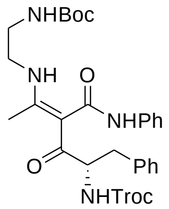

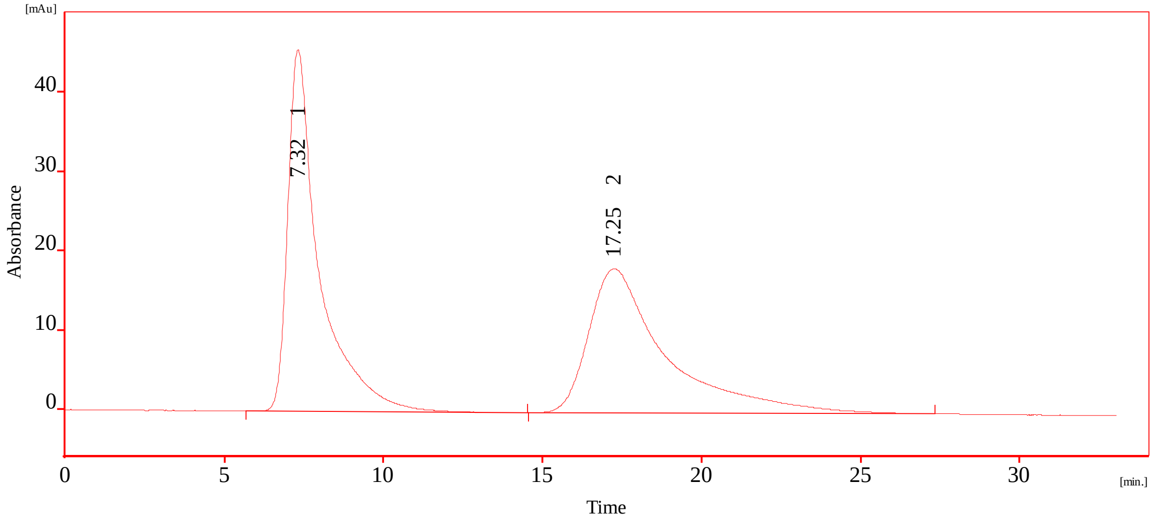

Column: Lux Amylose-1 (250×4.6)
Eluent: Hexane/Isopropanol 4:1
Flow: 1 mL/min
Detection: 310 nm

3f, racemic

Reten. Time [min]	Area [%]
7.32	50.6
17.25	49.4

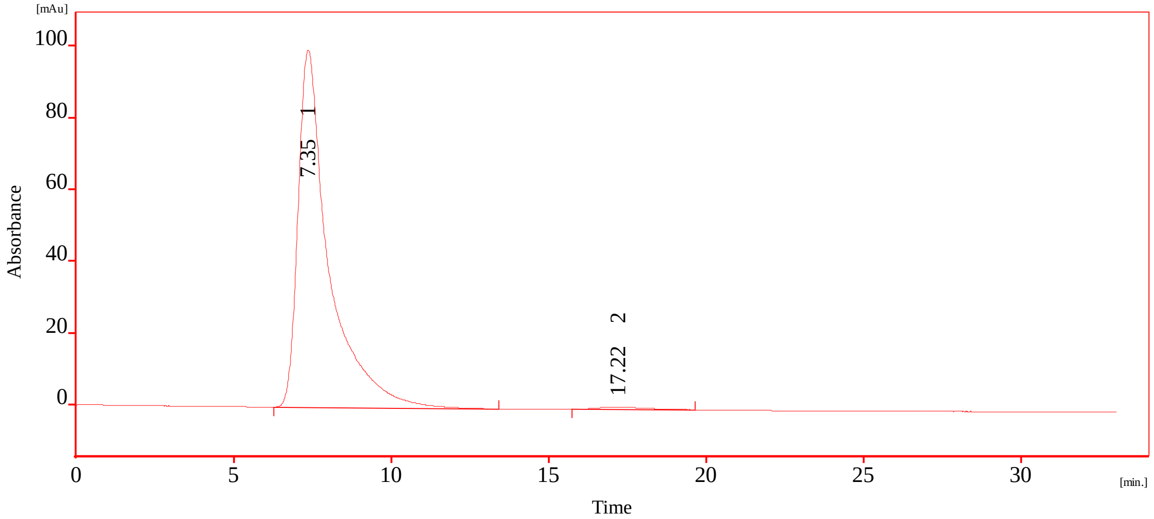

Column: Lux Amylose-1 (250×4.6)
Eluent: Hexane/Isopropanol 4:1
Flow: 1 mL/min
Detection: 310 nm

3f (L)

Reten. Time [min]	Area [%]
7.35	98.9
17.22	1.1

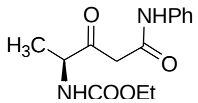

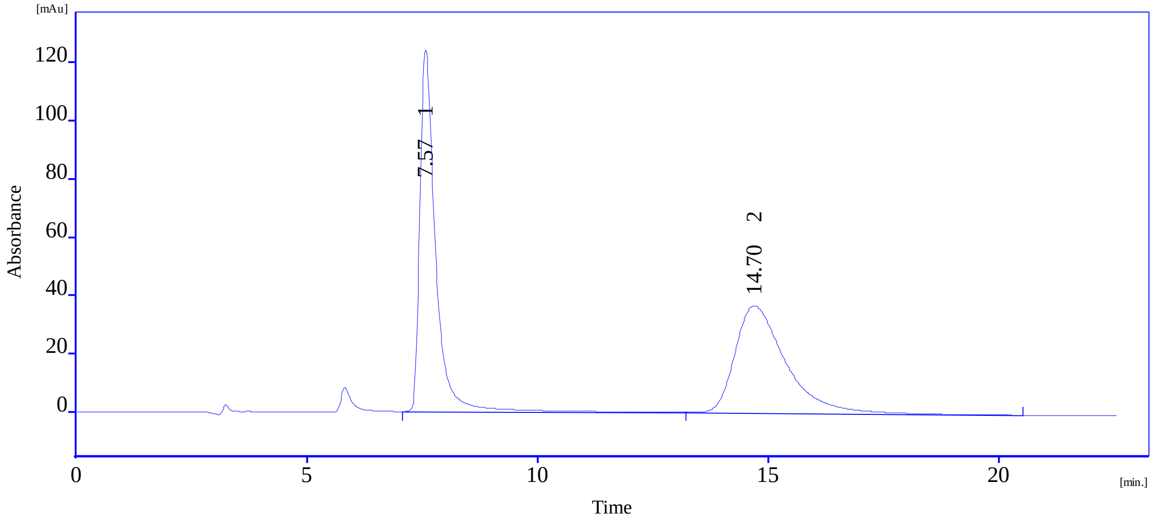

Column: Lux Amylose-2 (250×4.6)
Eluent: Hexane/Isopropanol 1:1
Flow: 1 mL/min
Detection: 244 nm

5c, racemic

Reten. Time [min]	Area [%]
7.57	51.1
14.70	48.9

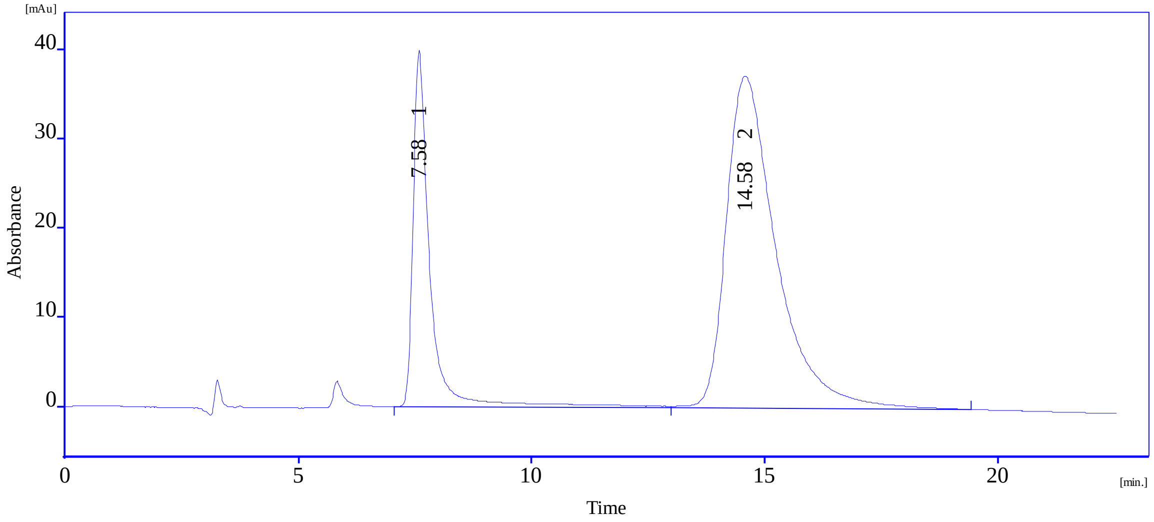

Column: Lux Amylose-2 (250×4.6)
Eluent: Hexane/Isopropanol 1:1
Flow: 1 mL/min
Detection: 244 nm

5c
5 min deprotection of **3c** in neat TFA

Reten. Time [min]	Area [%]
7.58	26.8
14.58	73.2

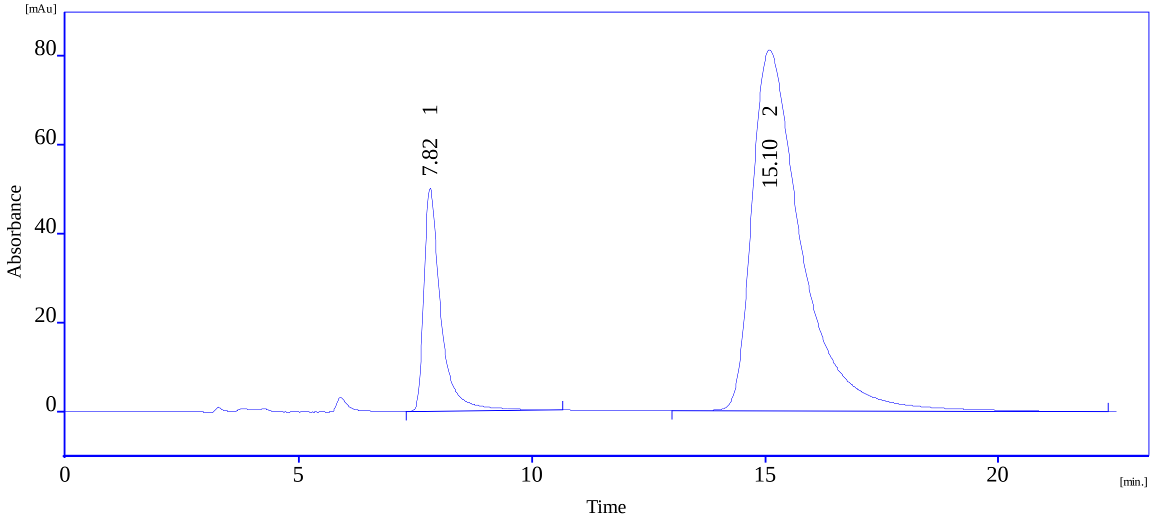

Column: Lux Amylose-2 (250×4.6)
Eluent: Hexane/Isopropanol 1:1
Flow: 1 mL/min
Detection: 244 nm

5c
15 min deprotection of **3c** in TFA/CH_2Cl_2 (1:4)

Reten. Time [min]	Area [%]
7.82	17.6
15.10	82.4

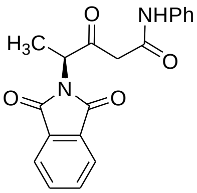

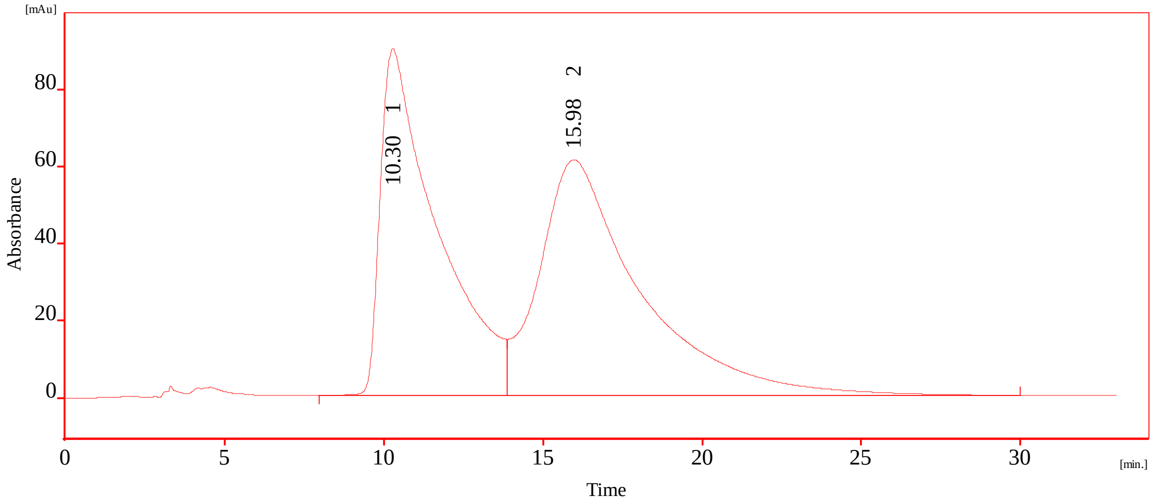

Column: Lux Amylose-1 (250×4.6)
Eluent: Hexane/Ethanol 1:3
Flow: 1 mL/min
Detection: 244 nm

5k, racemic

Reten. Time [min]	Area [%]
10.30	45.3
15.98	54.7

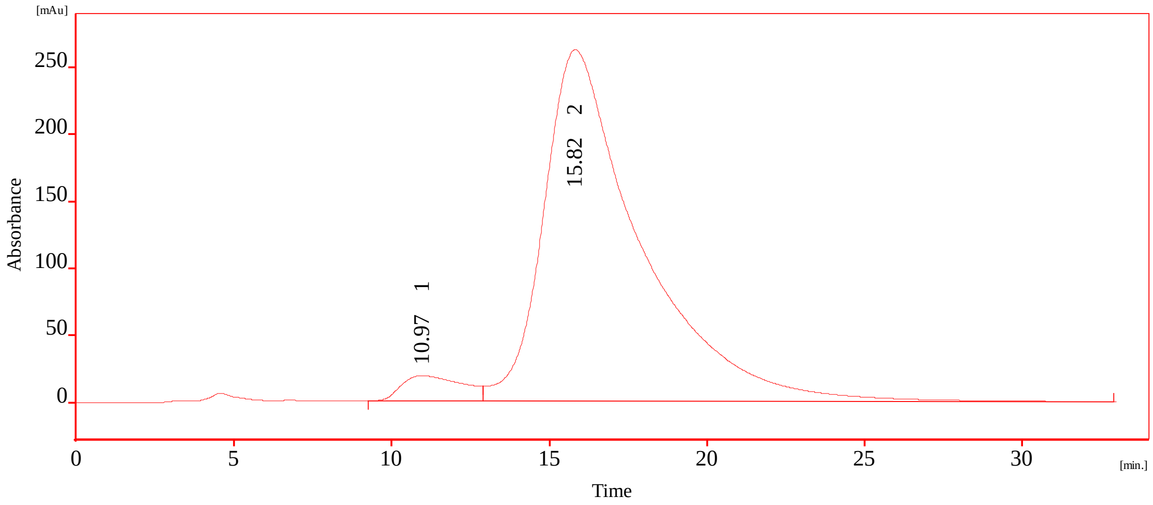

Column: Lux Amylose-1 (250×4.6)
Eluent: Hexane/Ethanol 1:3
Flow: 1 mL/min
Detection: 244 nm

5k (L)
30 min deprotection of **3k** in TFA/CH_2Cl_2 (1:10)

Reten. Time [min]	Area [%]
10.97	4.3
15.82	95.7

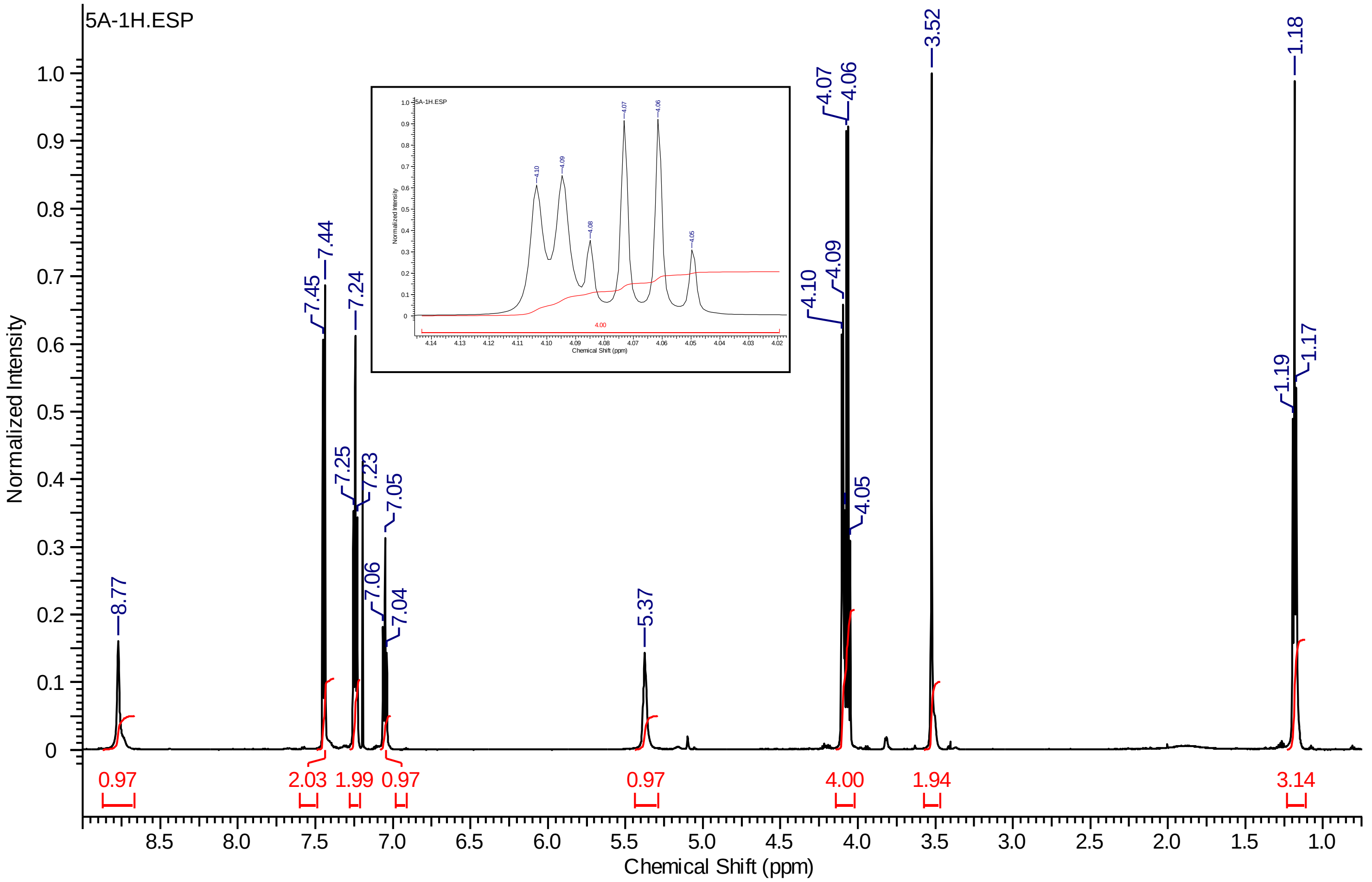
5A-1H.ESP
Normalized Intensity
Chemical Shift (ppm)
8.77
7.45
7.44
7.25
7.24
7.23
7.06
7.05
7.04
5.37
4.10
4.09
4.07
4.06
4.05
3.52
1.19
1.18
1.17
0.97
2.03
1.99
0.97
0.97
4.00
1.94
3.14

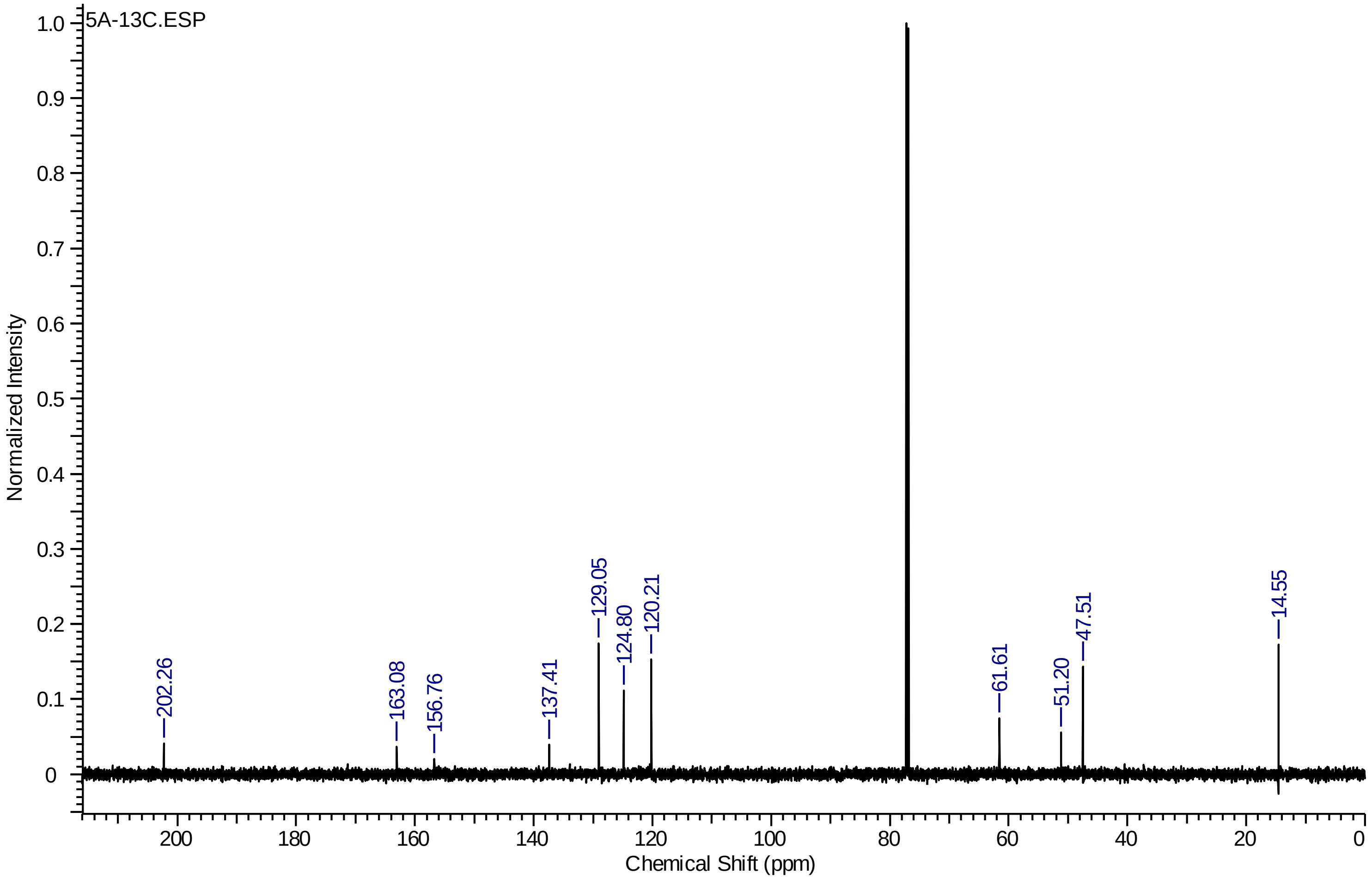
5A-13C.ESP
Normalized Intensity
Chemical Shift (ppm)
202.26
163.08
156.76
137.41
129.05
124.80
120.21
61.61
51.20
47.51
14.55

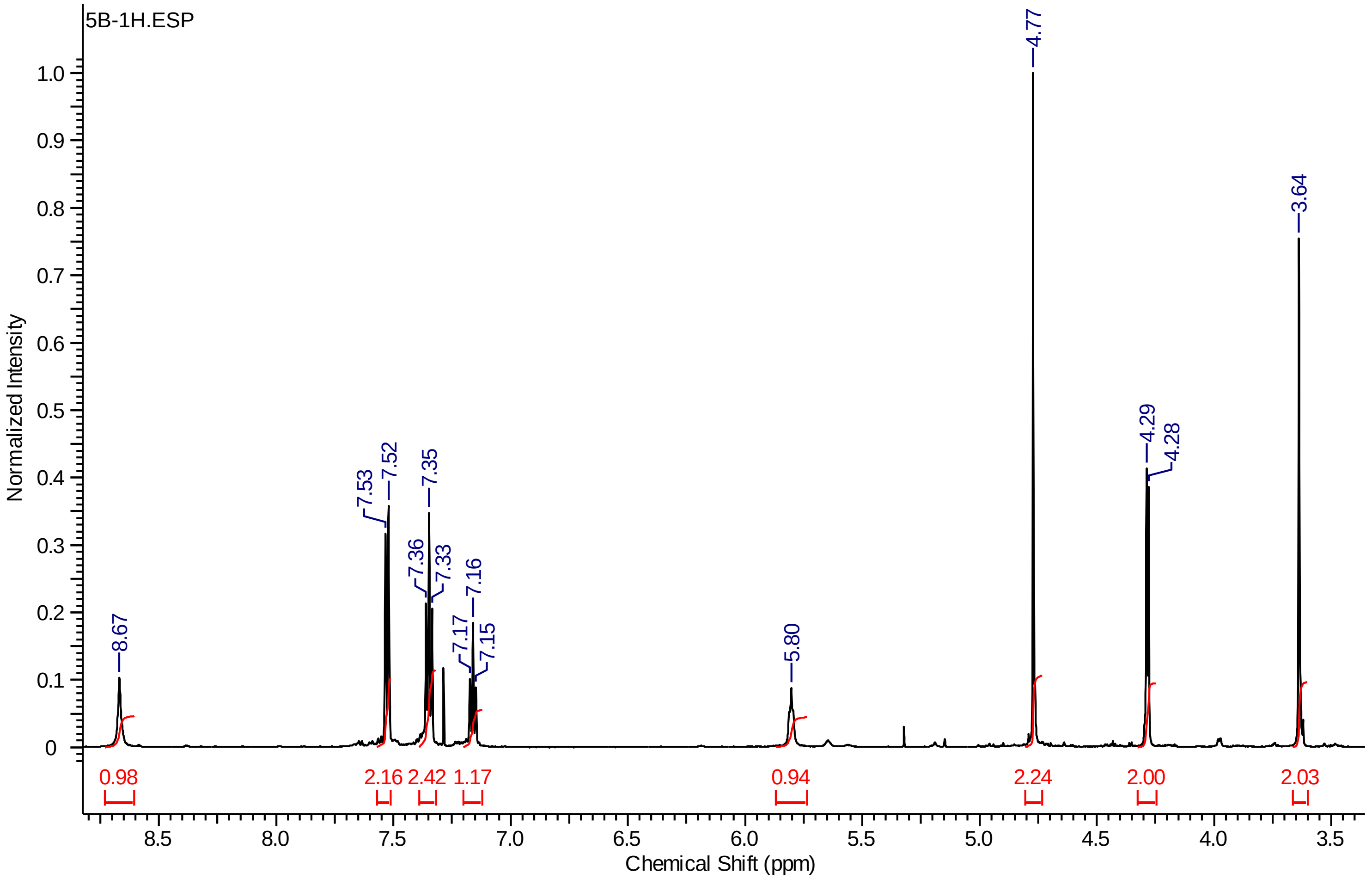
5B-1H.ESP
Normalized Intensity
Chemical Shift (ppm)
8.67
7.53
7.52
7.36
7.35
7.33
7.17
7.16
7.15
5.80
4.77
4.29
4.28
3.64
0.98
2.16
2.42
1.17
0.94
2.24
2.00
2.03

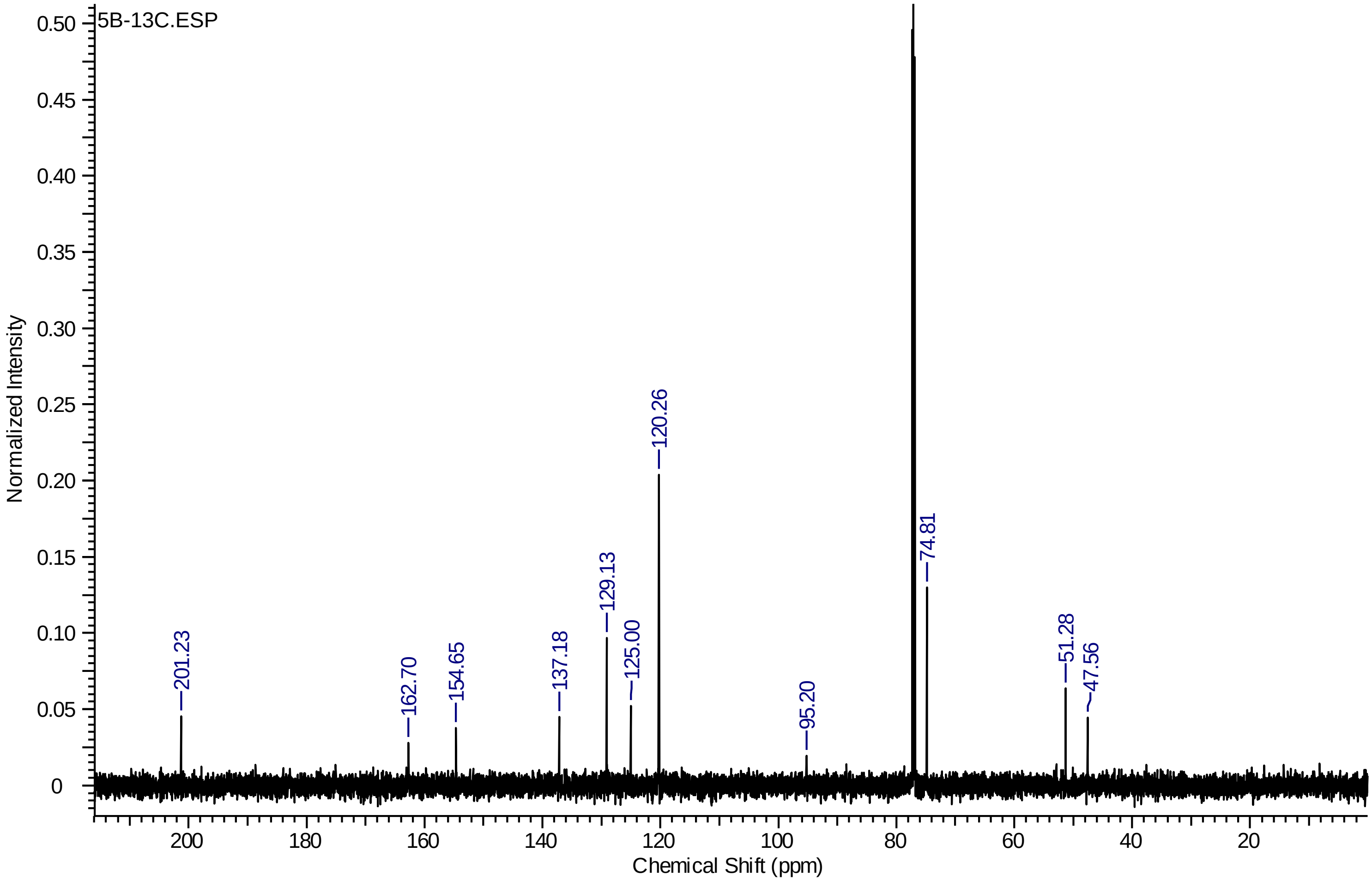
5B-13C.ESP
Normalized Intensity
Chemical Shift (ppm)
201.23
162.70
154.65
137.18
129.13
125.00
120.26
95.20
74.81
51.28
47.56

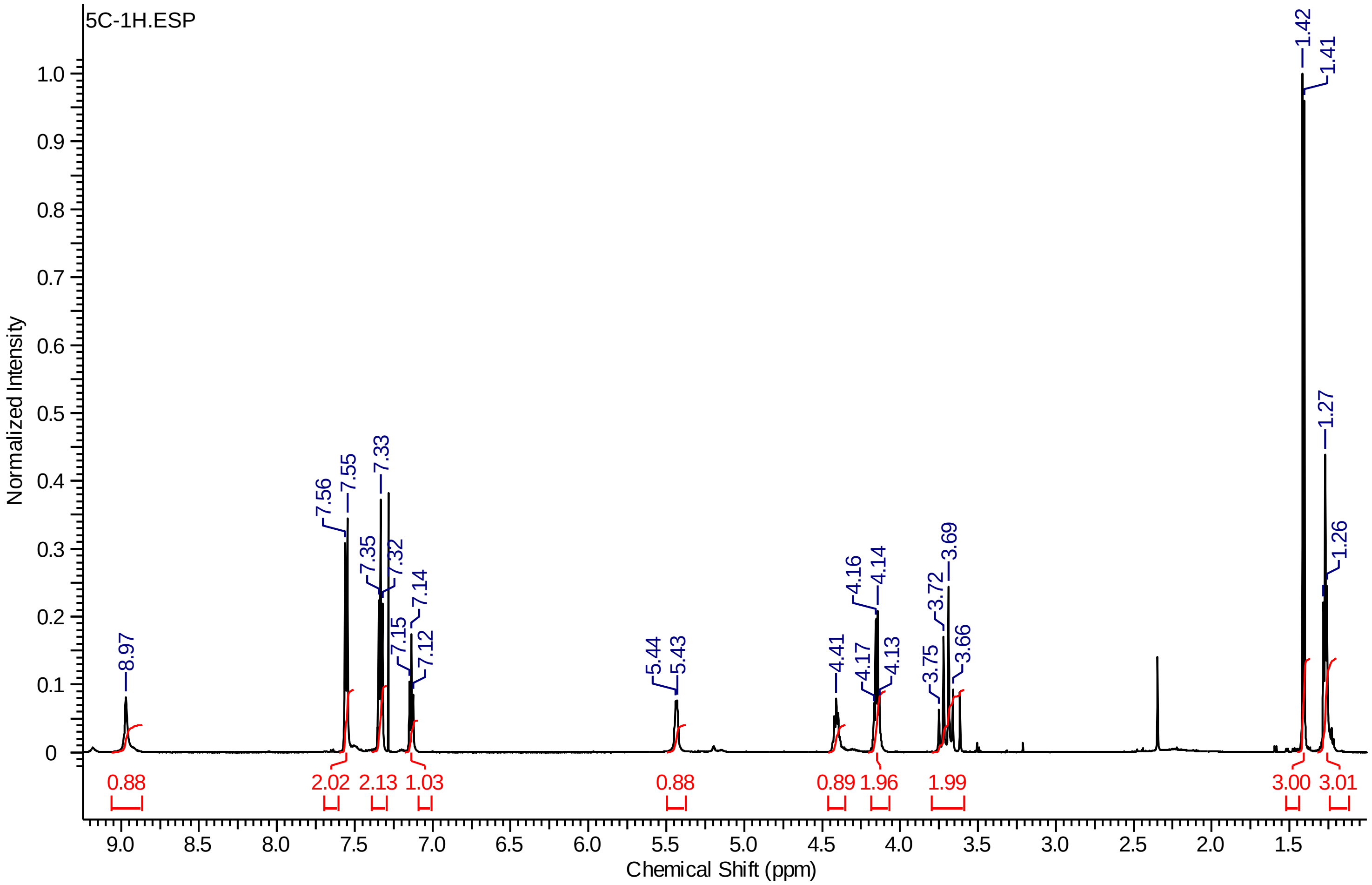
5C-1H.ESP
Normalized Intensity
Chemical Shift (ppm)
8.97
7.56
7.55
7.35
7.33
7.32
7.15
7.14
7.12
5.44
5.43
4.41
4.17
4.16
4.14
4.13
3.75
3.72
3.69
3.66
1.42
1.41
1.27
1.26
0.88
2.02
2.13
1.03
0.88
0.89
1.96
1.99
3.00
3.01

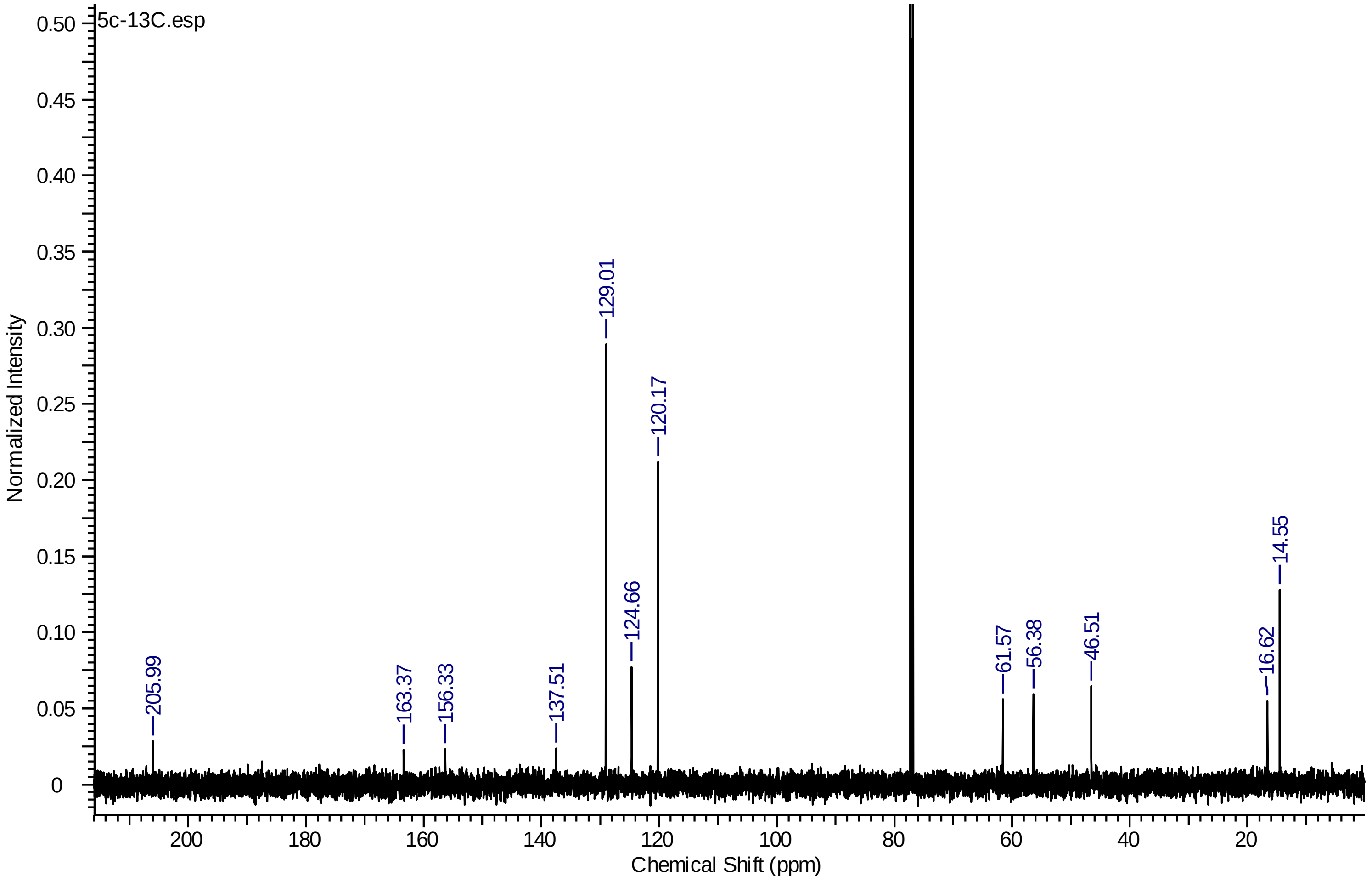
5c-13C.esp
Normalized Intensity
Chemical Shift (ppm)
205.99
163.37
156.33
137.51
129.01
124.66
120.17
61.57
56.38
46.51
16.62
14.55

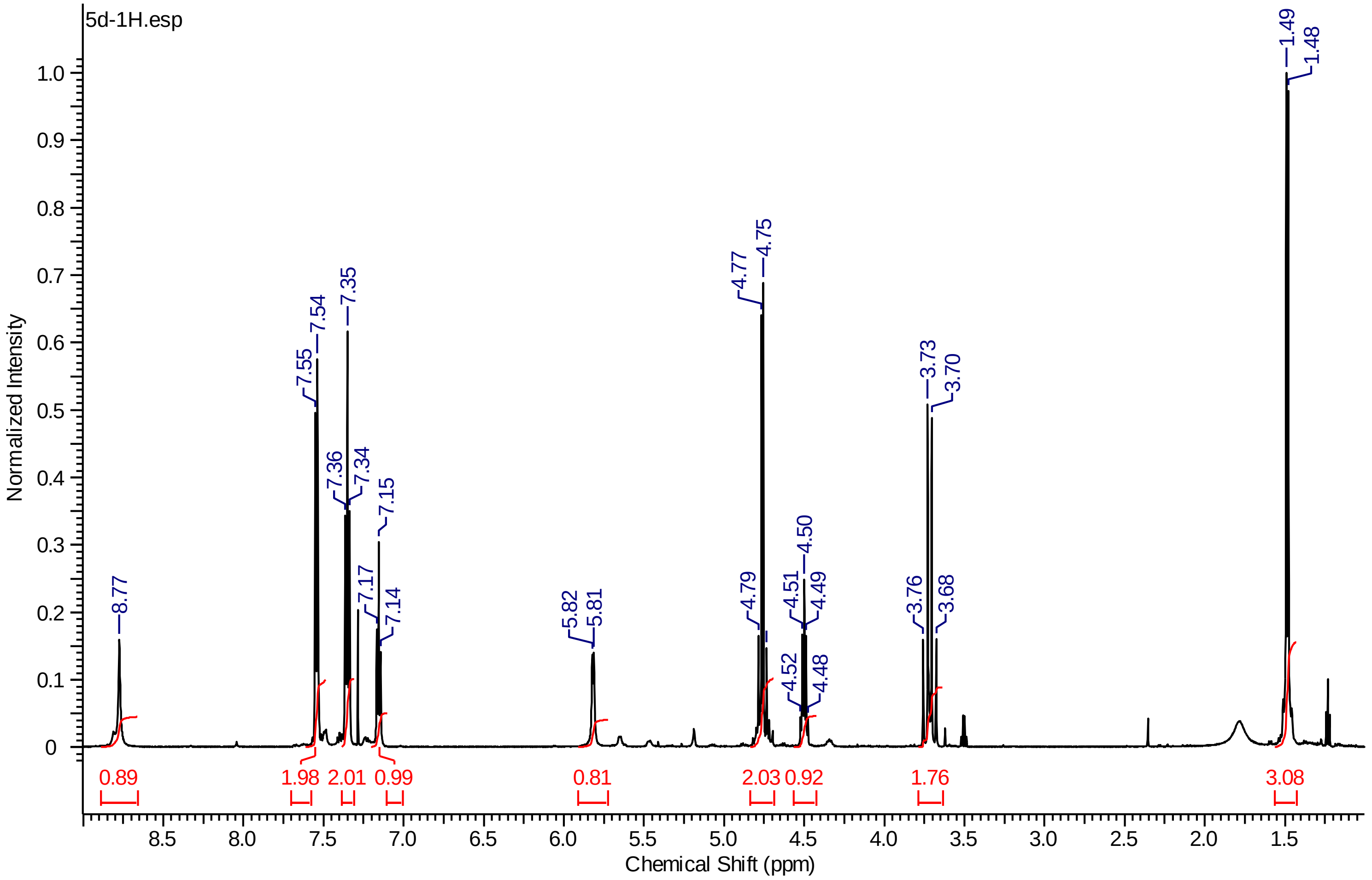
5d-1H.esp
Normalized Intensity
Chemical Shift (ppm)
8.77
7.55
7.54
7.36
7.35
7.34
7.17
7.15
7.14
5.82
5.81
4.79
4.77
4.75
4.52
4.51
4.50
4.49
4.48
3.76
3.73
3.70
3.68
1.49
1.48
0.89
1.98
2.01
0.99
0.81
2.03
0.92
1.76
3.08

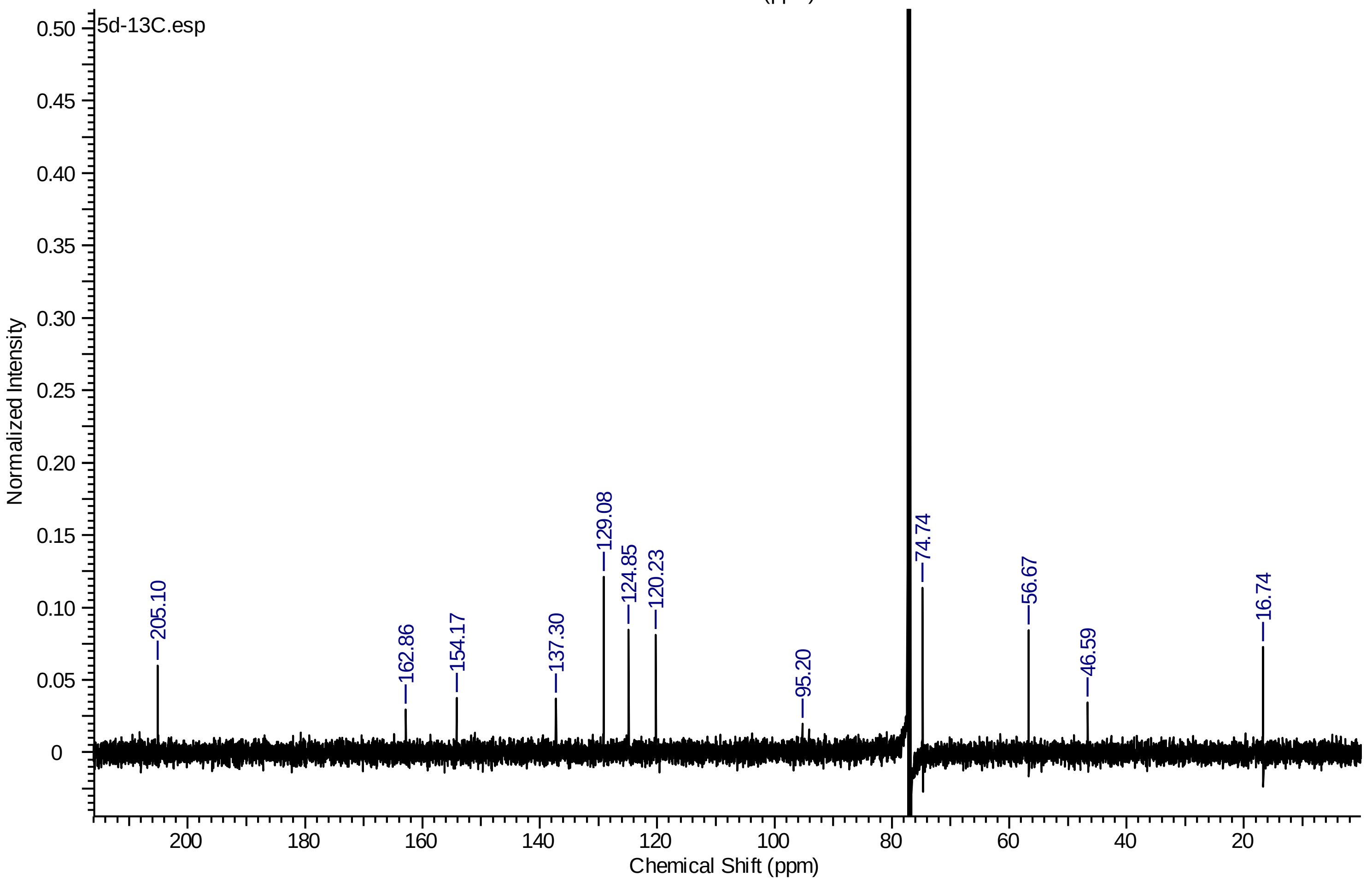
5d-13C.esp
Normalized Intensity
Chemical Shift (ppm)
205.10
162.86
154.17
137.30
129.08
124.85
120.23
95.20
74.74
56.67
46.59
16.74

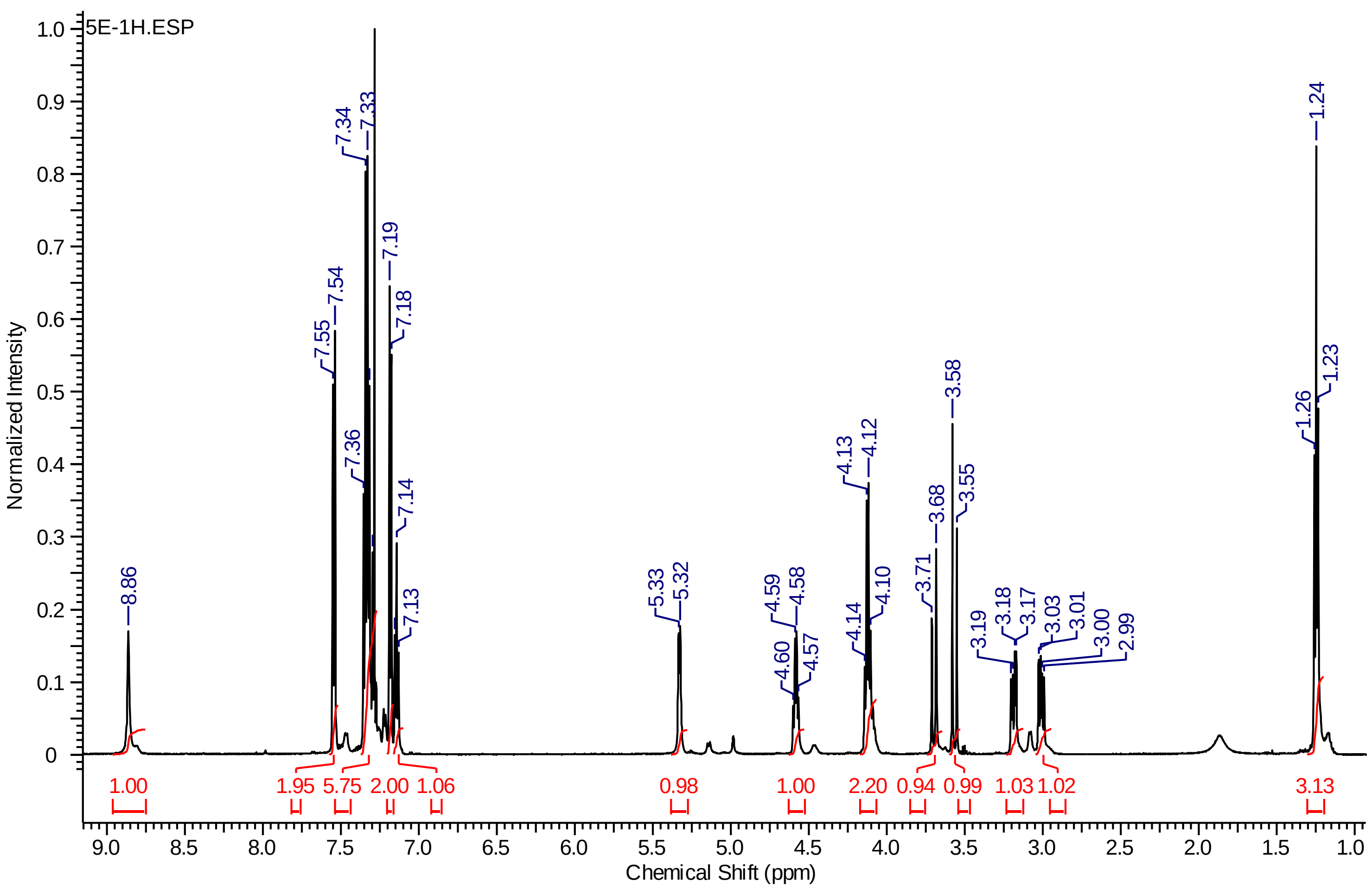
5E-1H.ESP
Normalized Intensity
Chemical Shift (ppm)
8.86
7.55
7.54
7.36
7.34
7.33
7.19
7.18
7.14
7.13
5.33
5.32
4.60
4.59
4.58
4.57
4.14
4.13
4.12
4.10
3.71
3.68
3.58
3.55
3.19
3.18
3.17
3.03
3.01
3.00
2.99
1.26
1.24
1.23
1.00
1.95
5.75
2.00
1.06
0.98
1.00
2.20
0.94
0.99
1.03
1.02
3.13

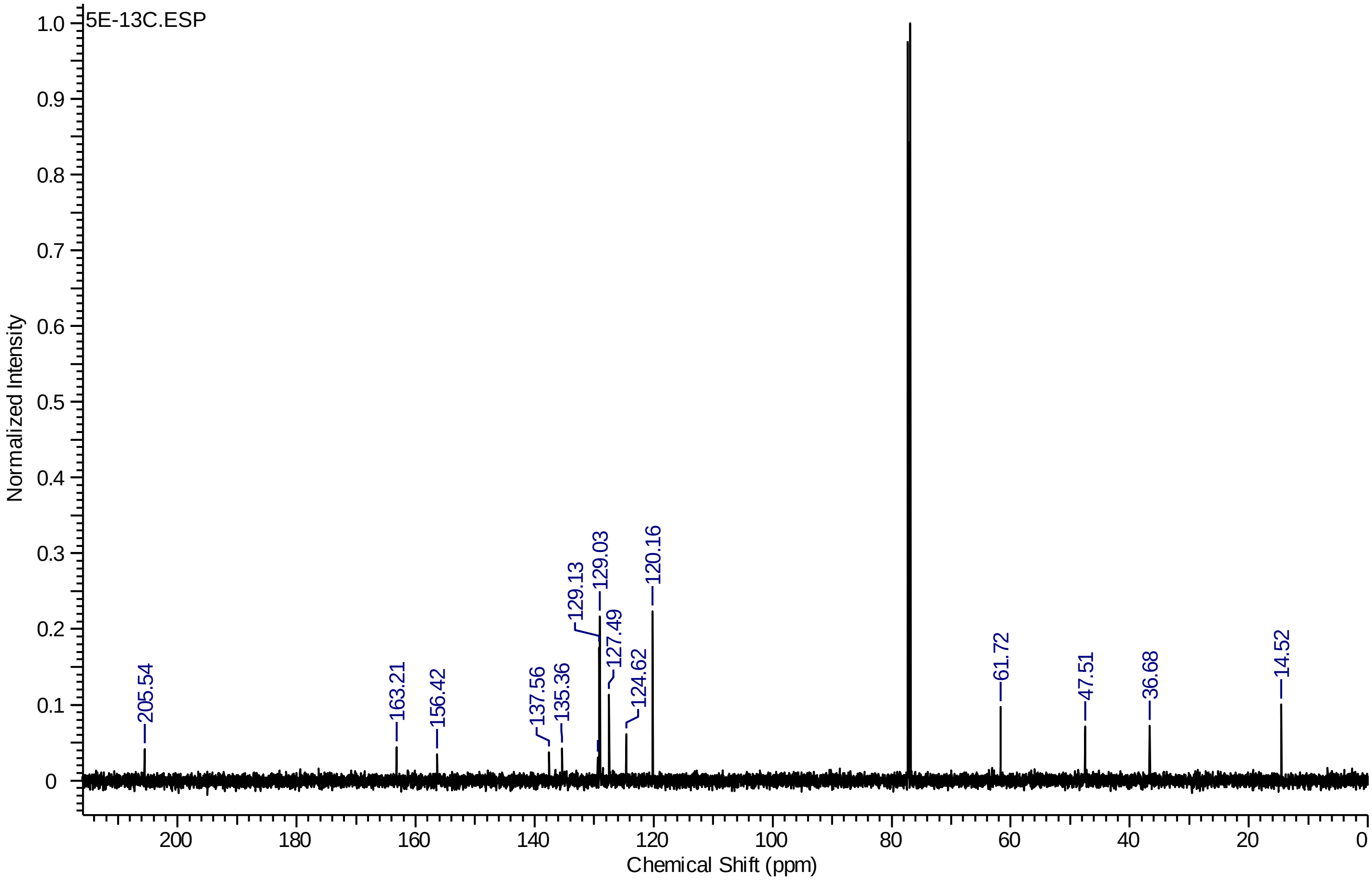
5E-13C.ESP
Normalized Intensity
Chemical Shift (ppm)
205.54
163.21
156.42
137.56
135.36
129.13
129.03
127.49
124.62
120.16
61.72
47.51
36.68
14.52

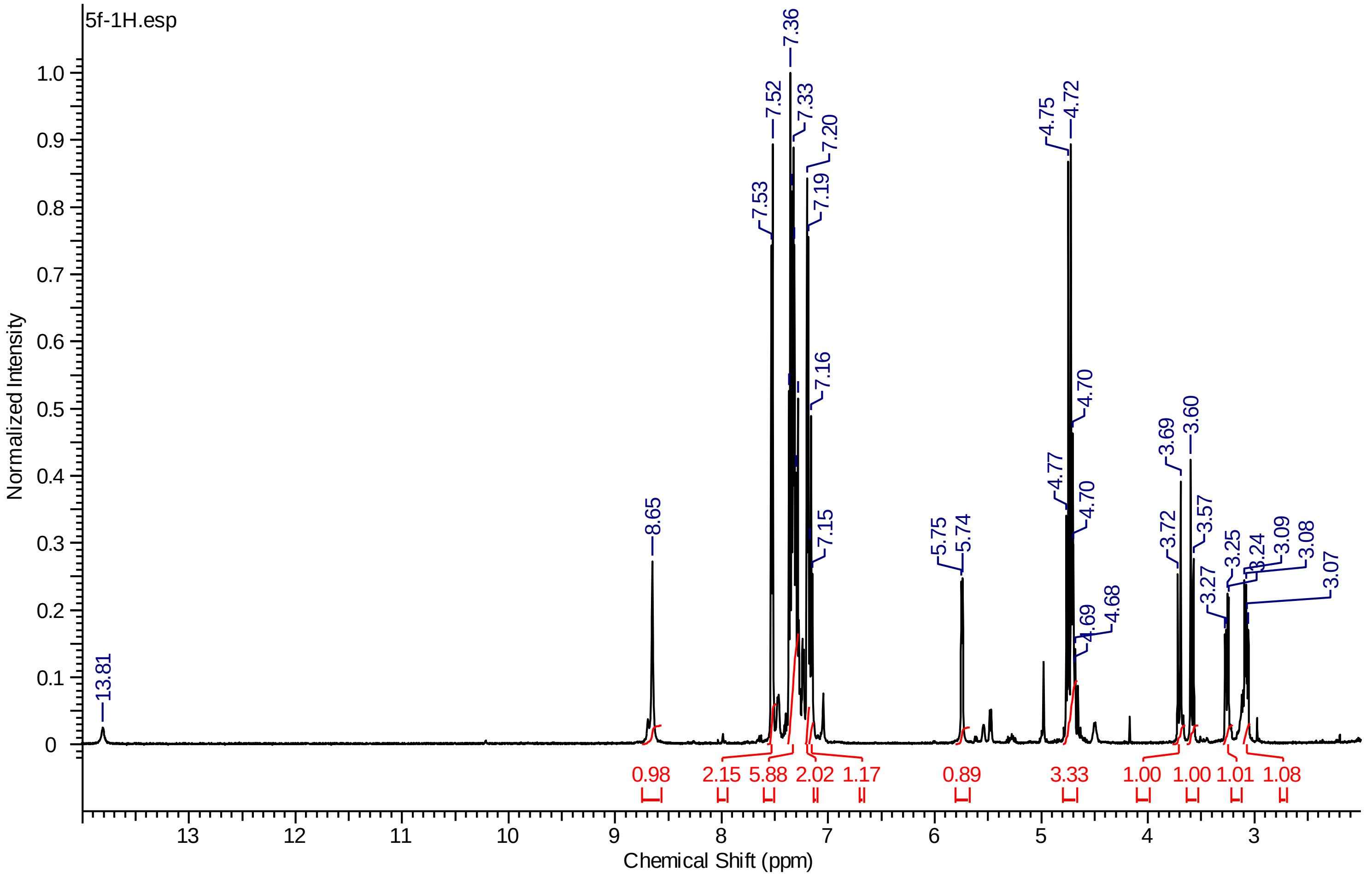
5f-1H.esp
Normalized Intensity
Chemical Shift (ppm)
13.81
8.65
7.53
7.52
7.36
7.33
7.20
7.19
7.16
7.15
5.75
5.74
4.77
4.75
4.72
4.70
4.70
4.69
4.68
3.72
3.69
3.60
3.57
3.27
3.25
3.24
3.09
3.08
3.07
0.98
2.15
5.88
2.02
1.17
0.89
3.33
1.00
1.00
1.01
1.08

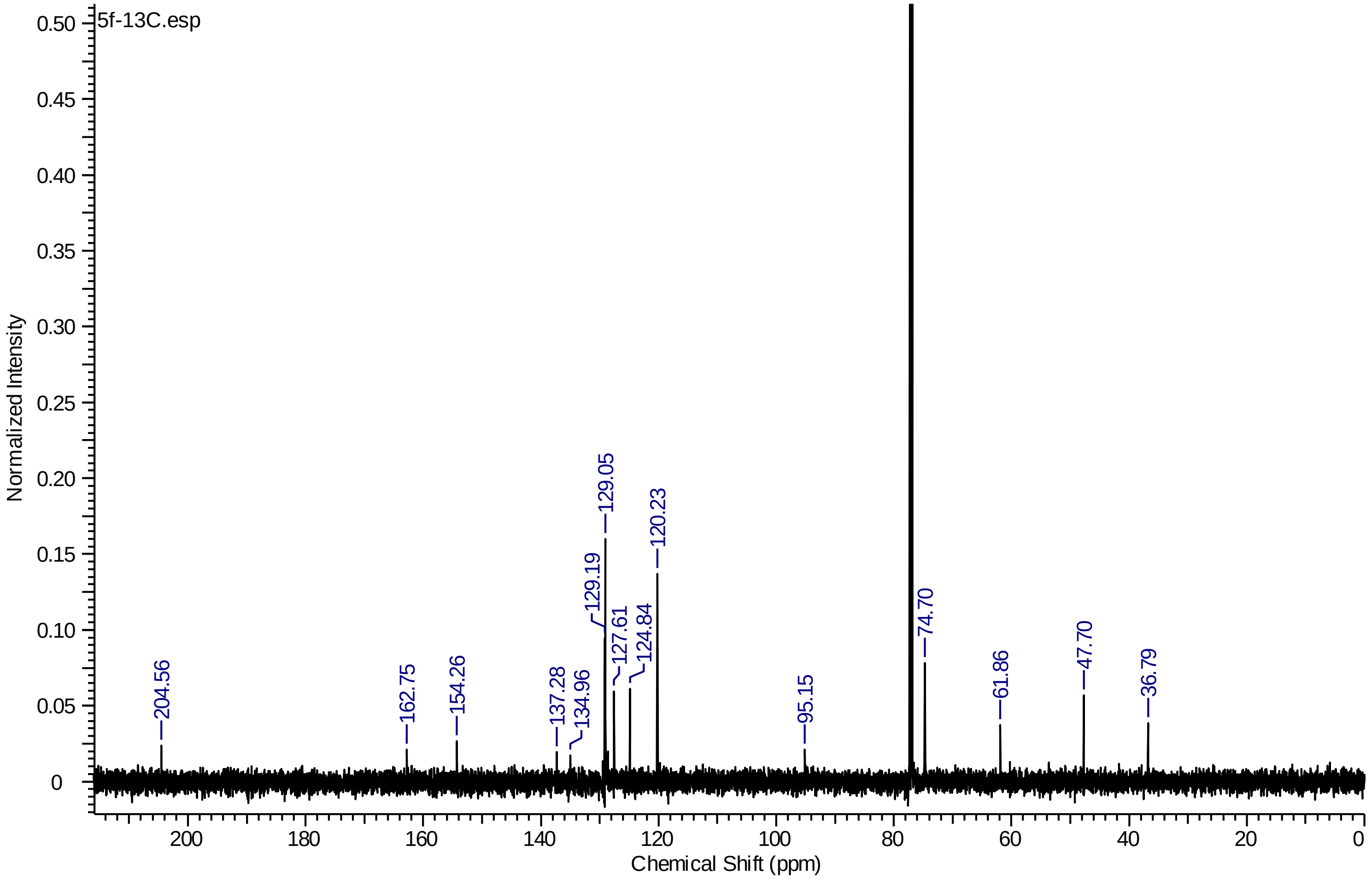
5f-13C.esp
Normalized Intensity
Chemical Shift (ppm)
204.56
162.75
154.26
137.28
134.96
129.19
129.05
127.61
124.84
120.23
95.15
74.70
61.86
47.70
36.79

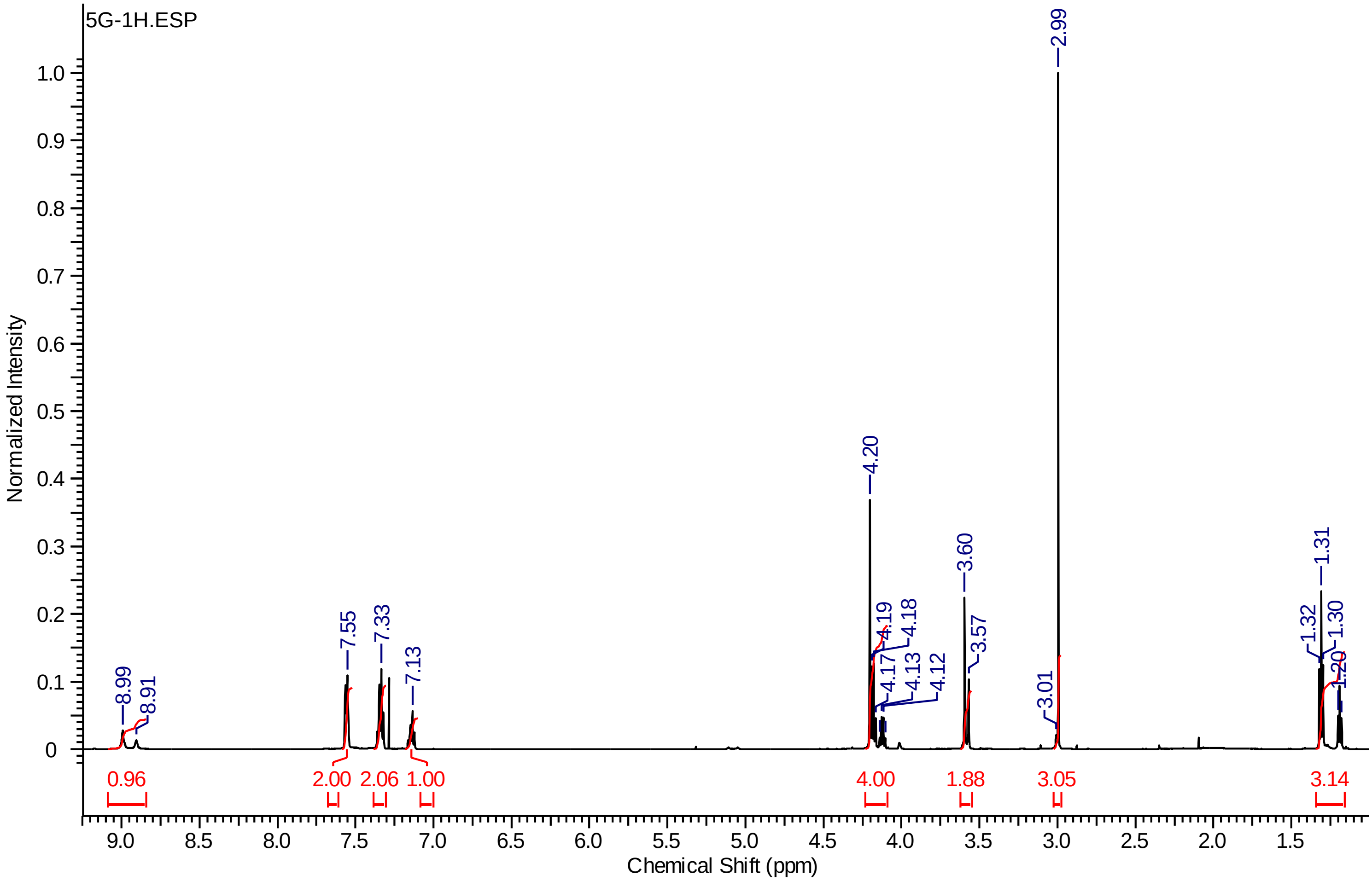
5G-1H.ESP
Normalized Intensity
Chemical Shift (ppm)
8.99
8.91
7.55
7.33
7.13
4.20
4.19
4.18
4.17
4.13
4.12
3.60
3.57
3.01
2.99
1.32
1.31
1.30
1.20
0.96
2.00
2.06
1.00
4.00
1.88
3.05
3.14

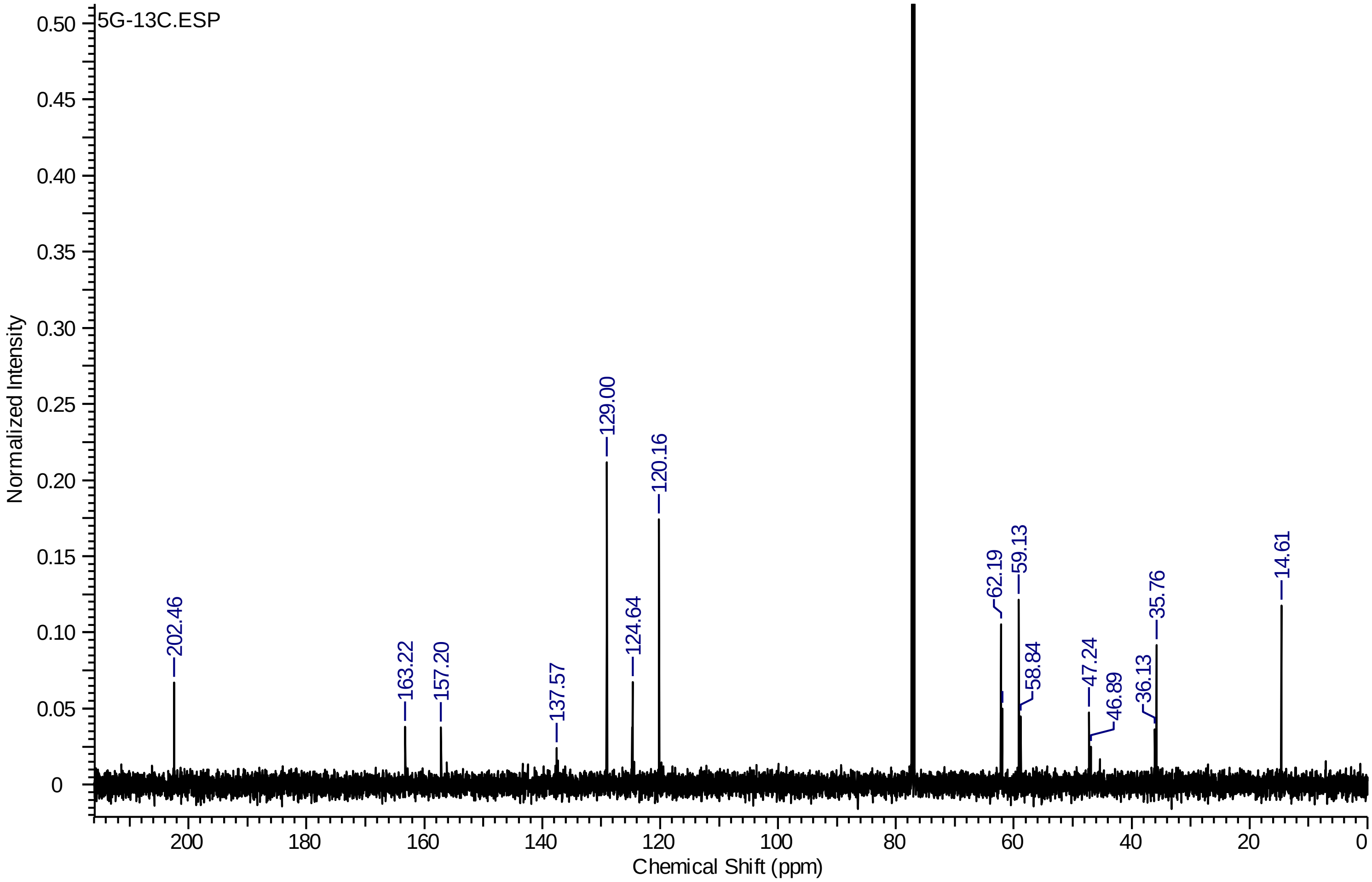
5G-13C.ESP
Normalized Intensity
Chemical Shift (ppm)
202.46
163.22
157.20
137.57
129.00
124.64
120.16
62.19
59.13
58.84
47.24
46.89
36.13
35.76
14.61

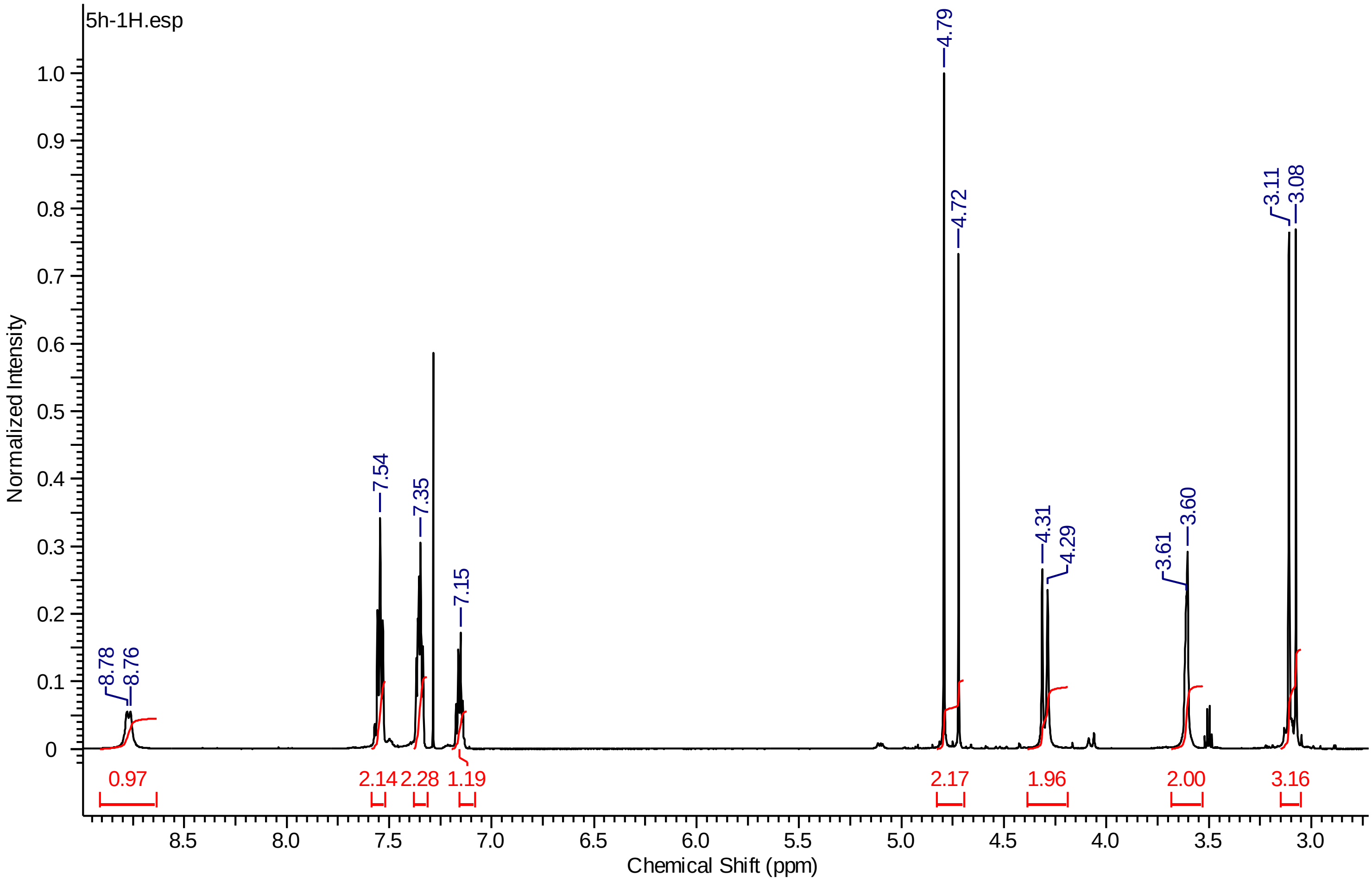
5h-1H.esp
Normalized Intensity
Chemical Shift (ppm)
8.78
8.76
7.54
7.35
7.15
4.79
4.72
4.31
4.29
3.61
3.60
3.11
3.08
0.97
2.14
2.28
1.19
2.17
1.96
2.00
3.16

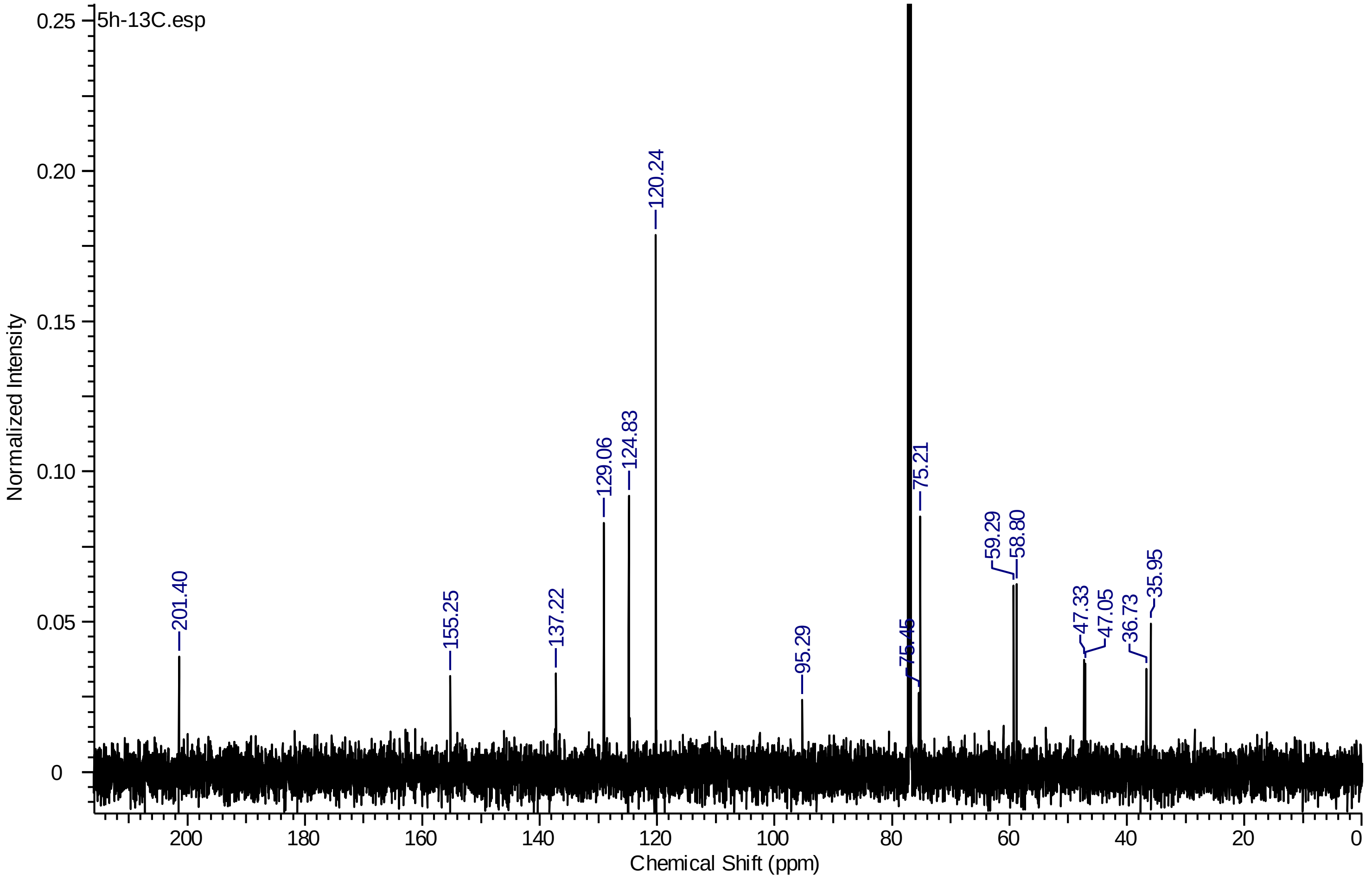
5h-13C.esp
Normalized Intensity
Chemical Shift (ppm)
201.40
155.25
137.22
129.06
124.83
120.24
95.29
75.45
75.21
59.29
58.80
47.33
47.05
36.73
35.95

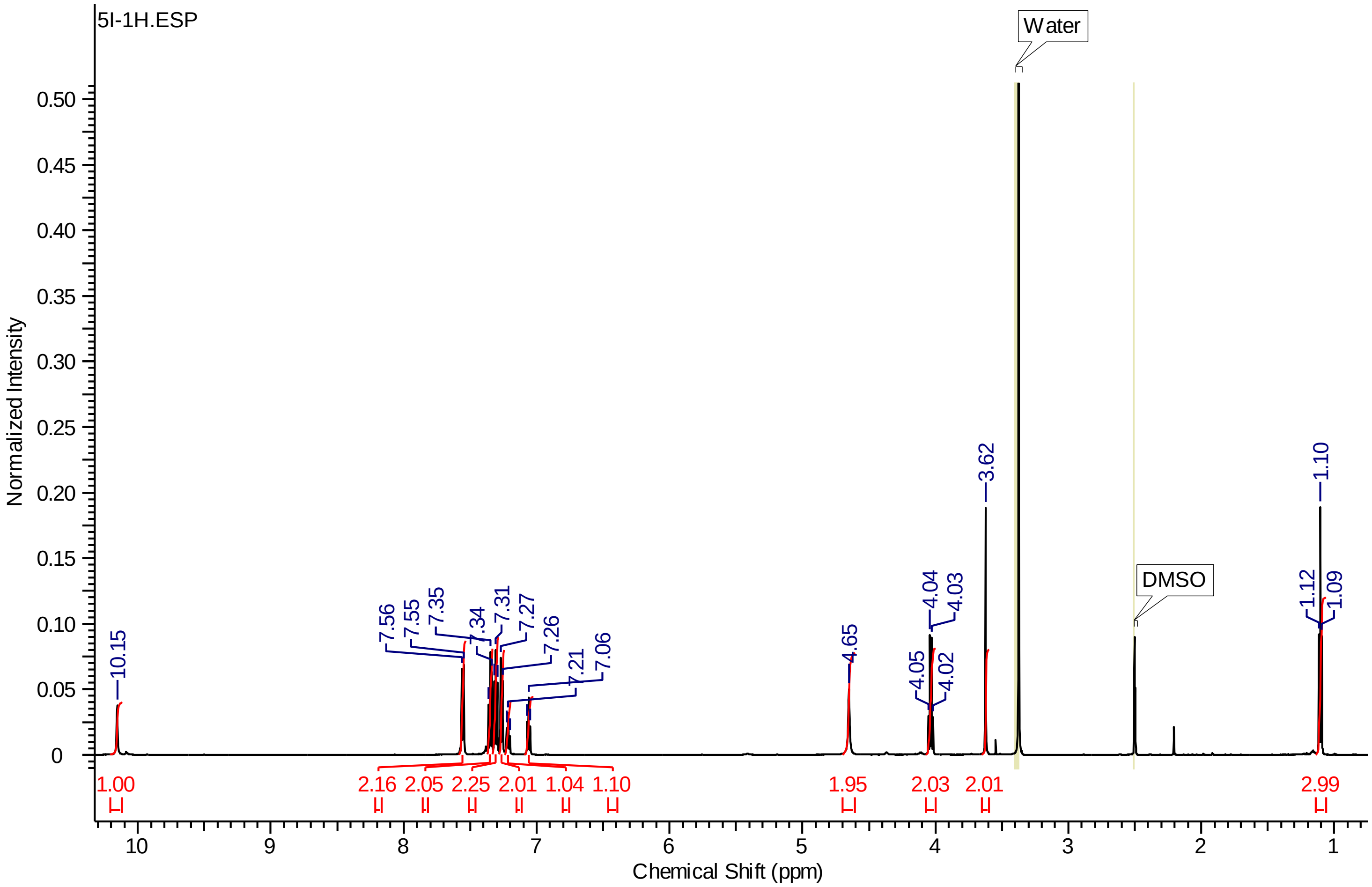
5I-1H.ESP
Water
DMSO
10.15
7.56
7.55
7.35
7.34
7.31
7.27
7.26
7.21
7.06
4.65
4.05
4.04
4.03
4.02
3.62
1.12
1.10
1.09
1.00
2.16
2.05
2.25
2.01
1.04
1.10
1.95
2.03
2.01
2.99
Normalized Intensity
Chemical Shift (ppm)

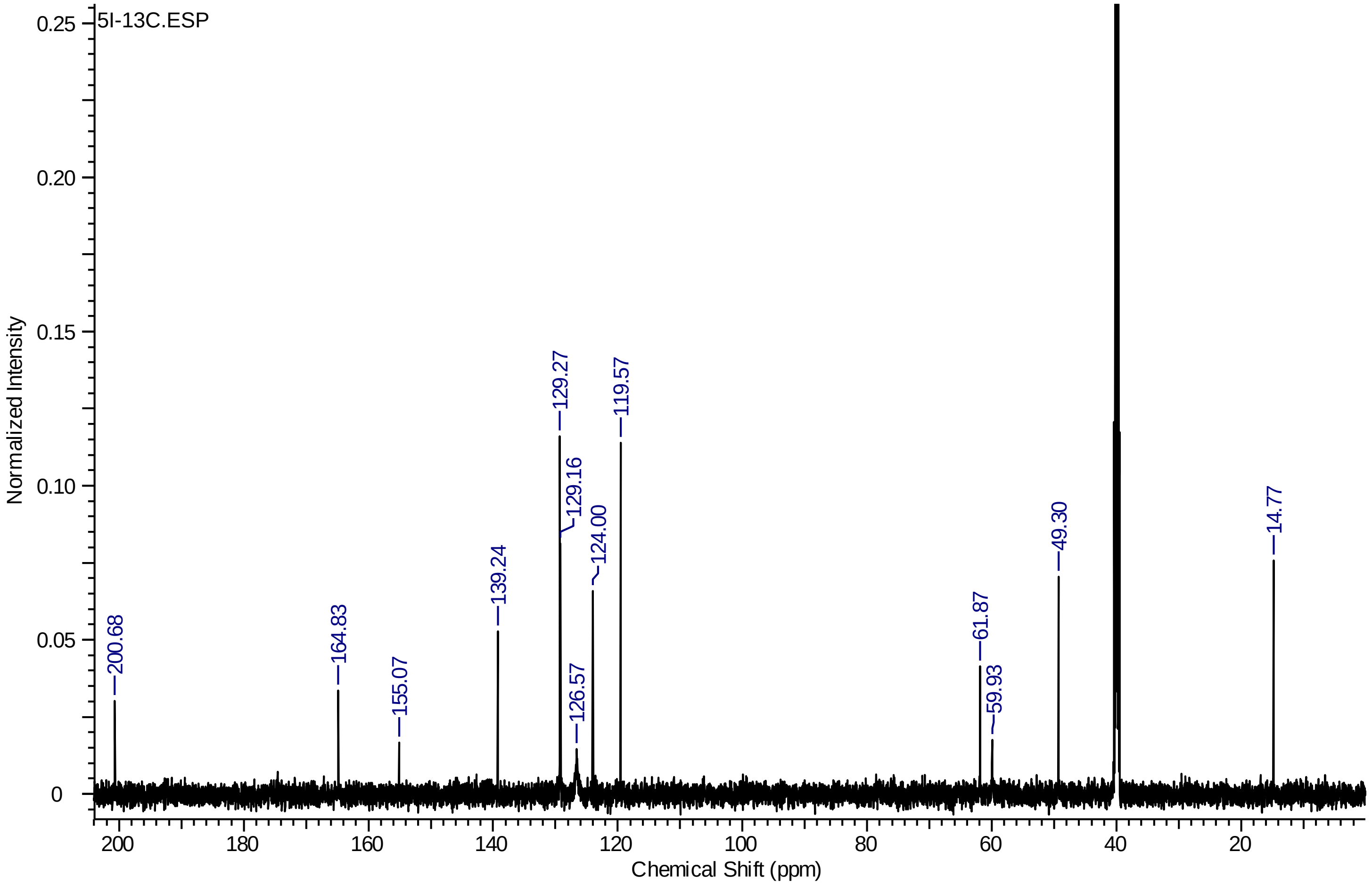
5I-13C.ESP
200.68
164.83
155.07
139.24
129.27
129.16
126.57
124.00
119.57
61.87
59.93
49.30
14.77
Normalized Intensity
Chemical Shift (ppm)

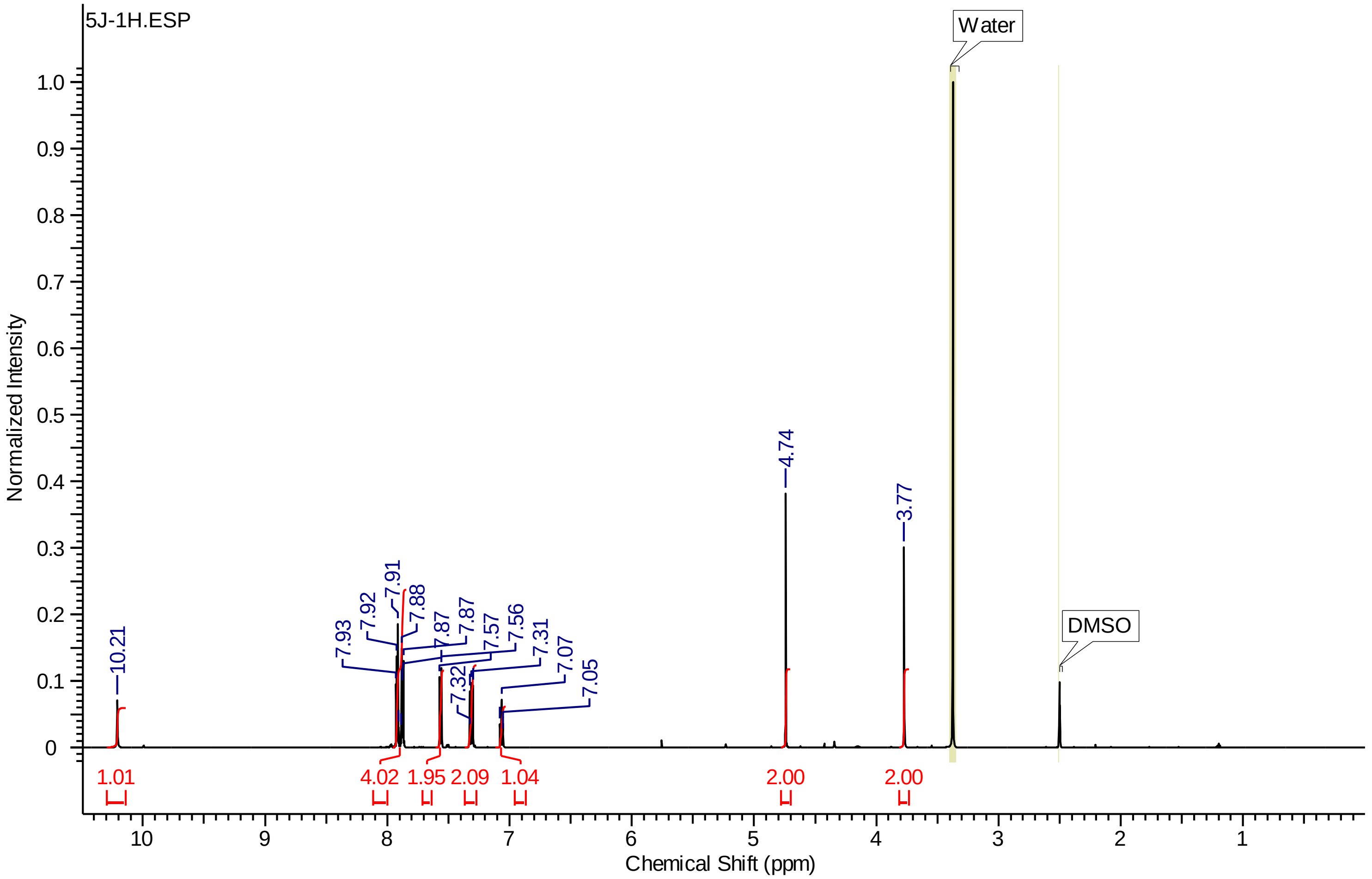
5J-1H.ESP
Water
DMSO
10.21
7.93
7.92
7.91
7.88
7.87
7.87
7.57
7.56
7.32
7.31
7.07
7.05
4.74
3.77
1.01
4.02
1.95
2.09
1.04
2.00
2.00
Normalized Intensity
Chemical Shift (ppm)

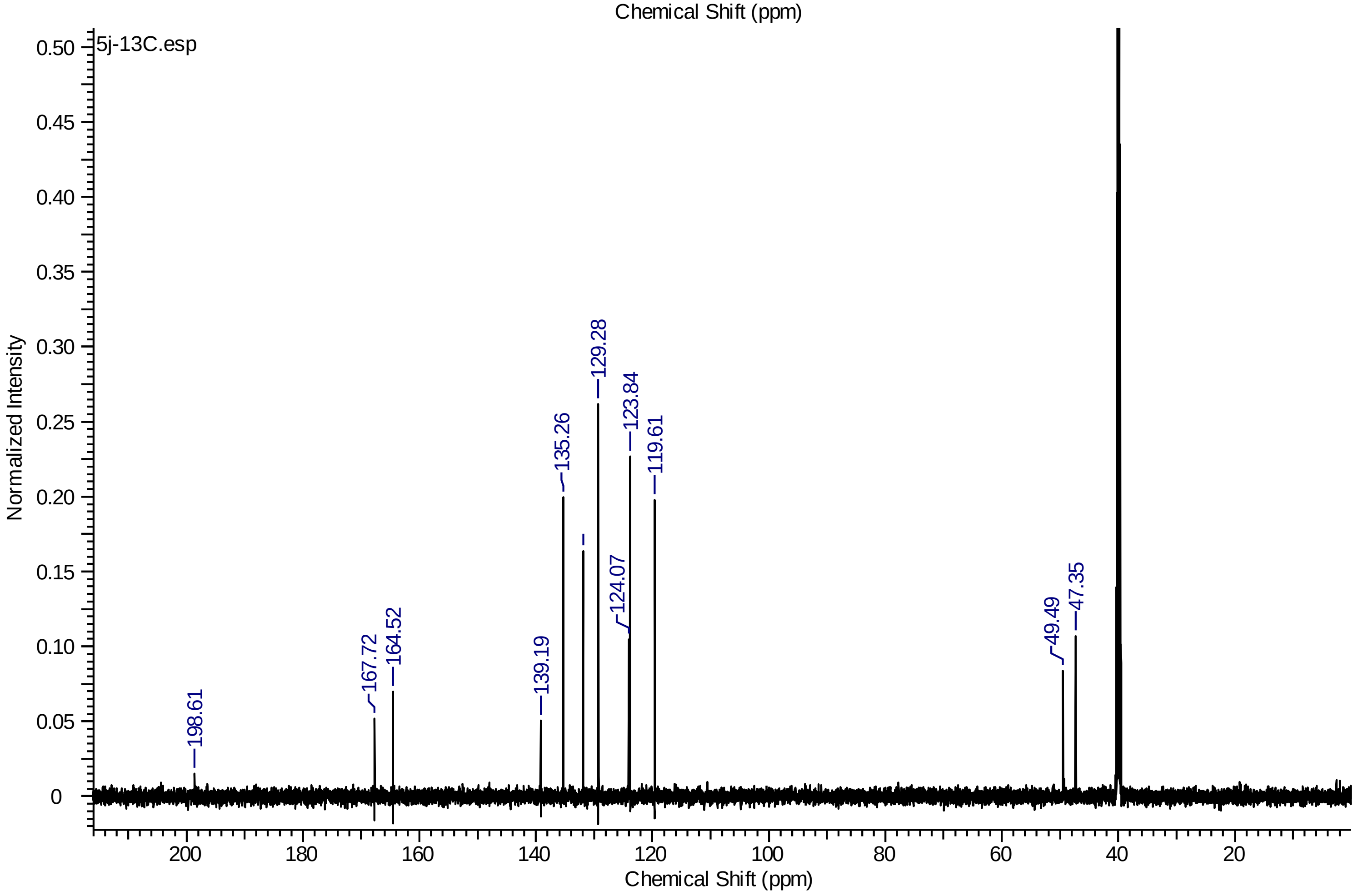
5j-13C.esp
198.61
167.72
164.52
139.19
135.26
129.28
124.07
123.84
119.61
49.49
47.35
Normalized Intensity
Chemical Shift (ppm)

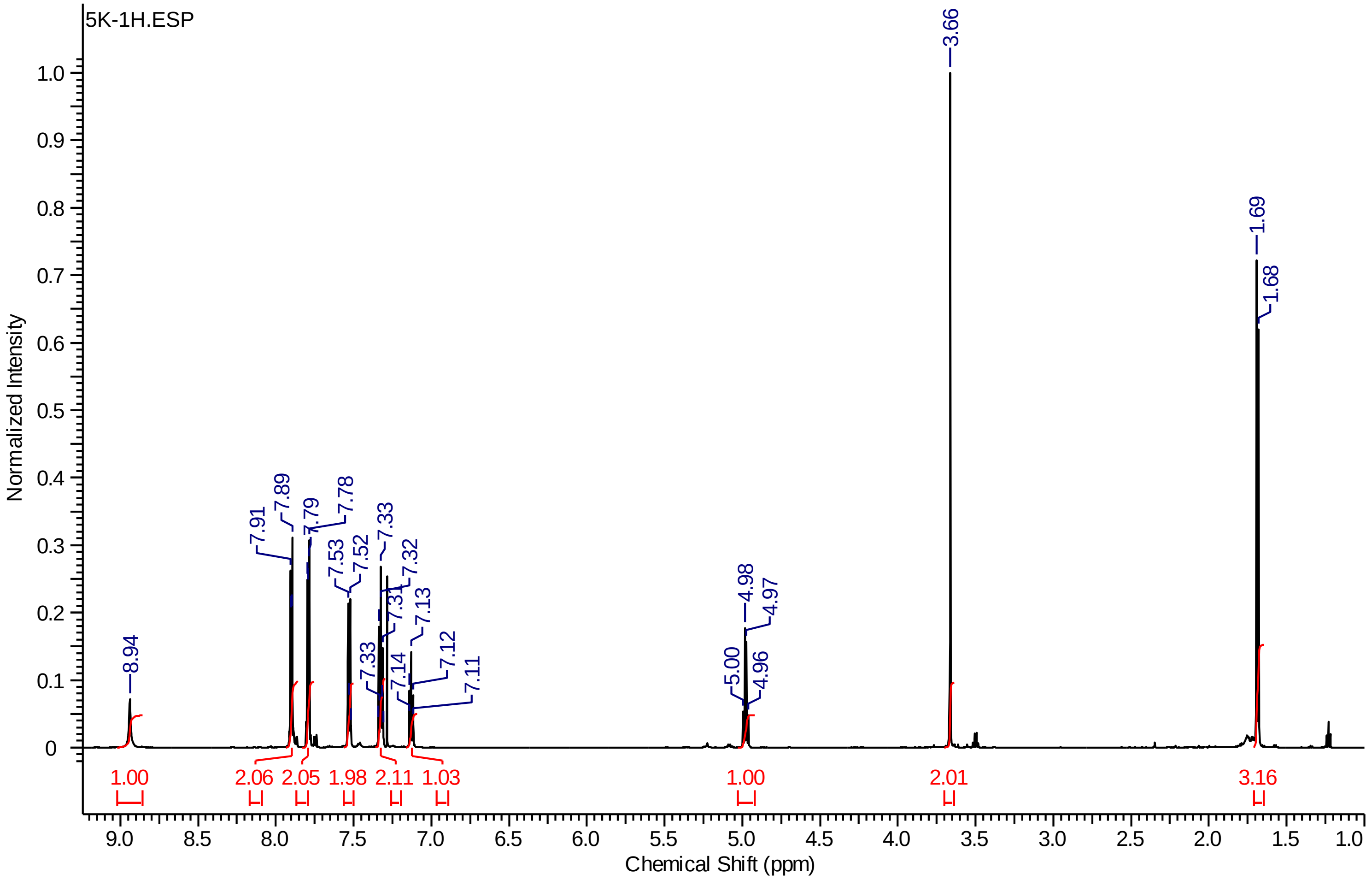
5K-1H.ESP
Normalized Intensity
Chemical Shift (ppm)
8.94
7.91
7.89
7.79
7.78
7.53
7.52
7.33
7.33
7.32
7.31
7.14
7.13
7.12
7.11
5.00
4.98
4.97
4.96
3.66
1.69
1.68
1.00
2.06
2.05
1.98
2.11
1.03
1.00
2.01
3.16

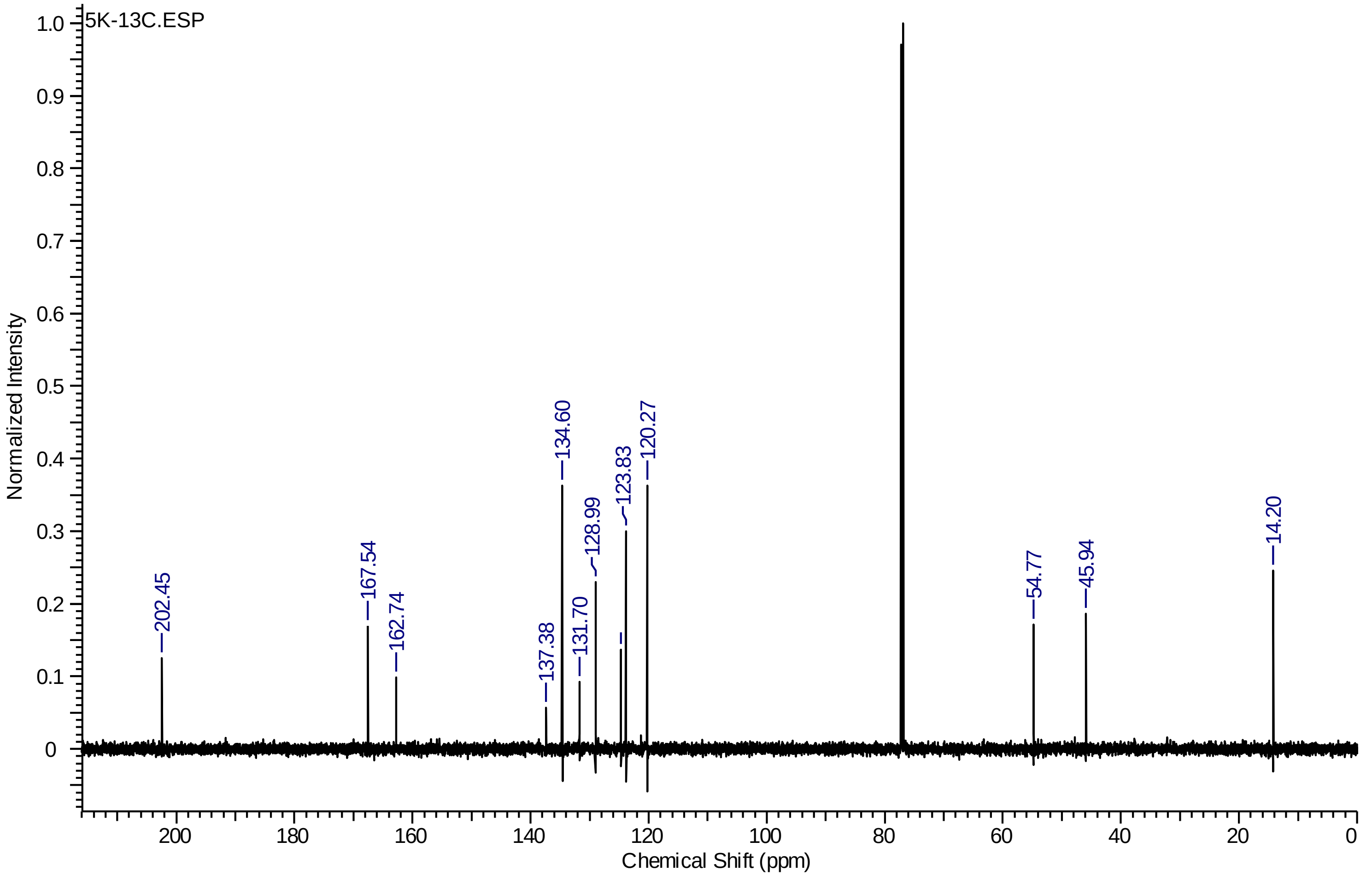
5K-13C.ESP
Normalized Intensity
Chemical Shift (ppm)
202.45
167.54
162.74
137.38
134.60
131.70
128.99
123.83
120.27
54.77
45.94
14.20

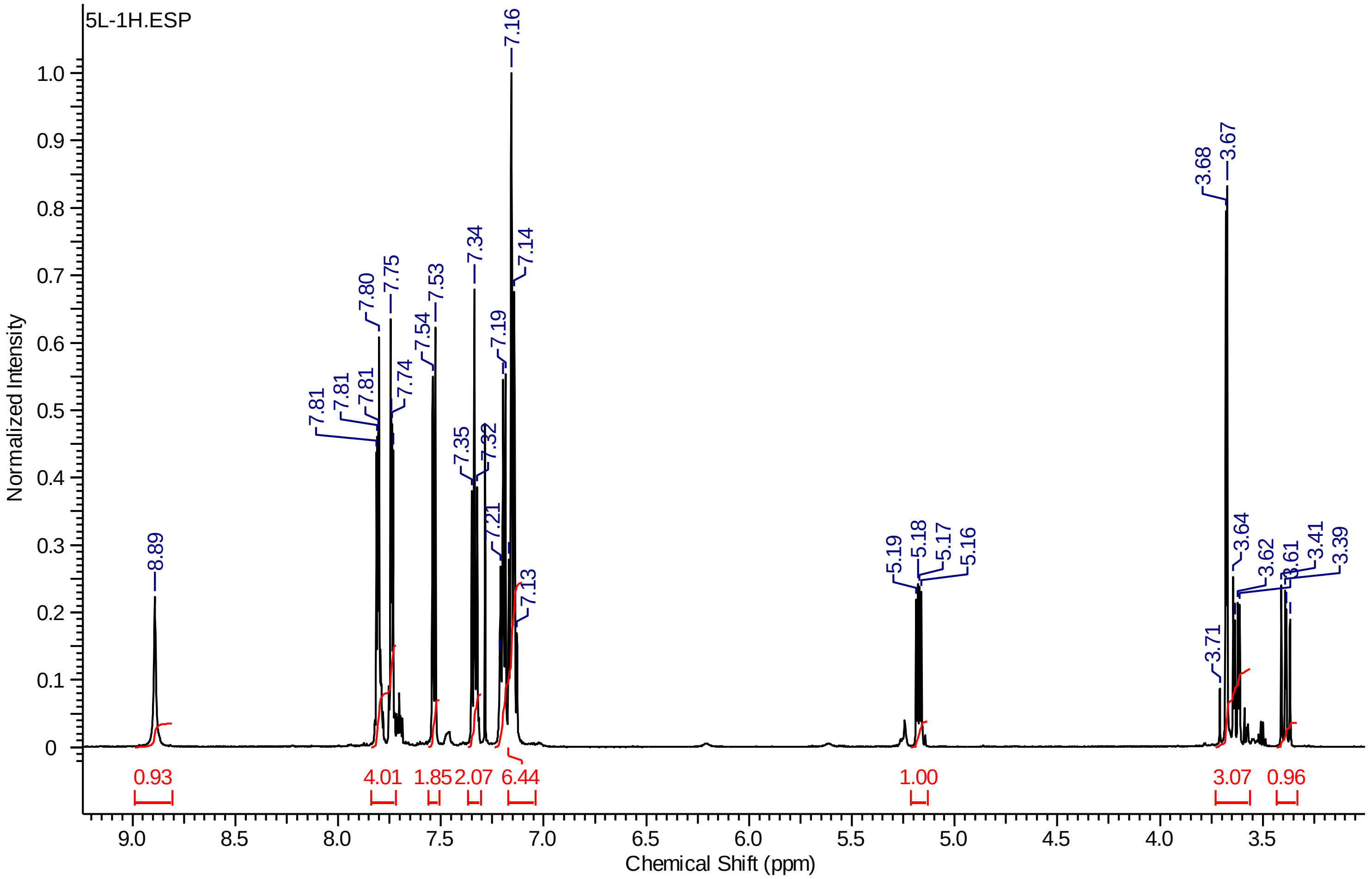

5L-1H.ESP
Normalized Intensity
Chemical Shift (ppm)
8.89
7.81
7.81
7.81
7.80
7.75
7.74
7.54
7.53
7.35
7.34
7.32
7.21
7.19
7.16
7.14
7.13
5.19
5.18
5.17
5.16
3.71
3.68
3.67
3.64
3.62
3.61
3.41
3.39
0.93
4.01
1.85
2.07
6.44
1.00
3.07
0.96

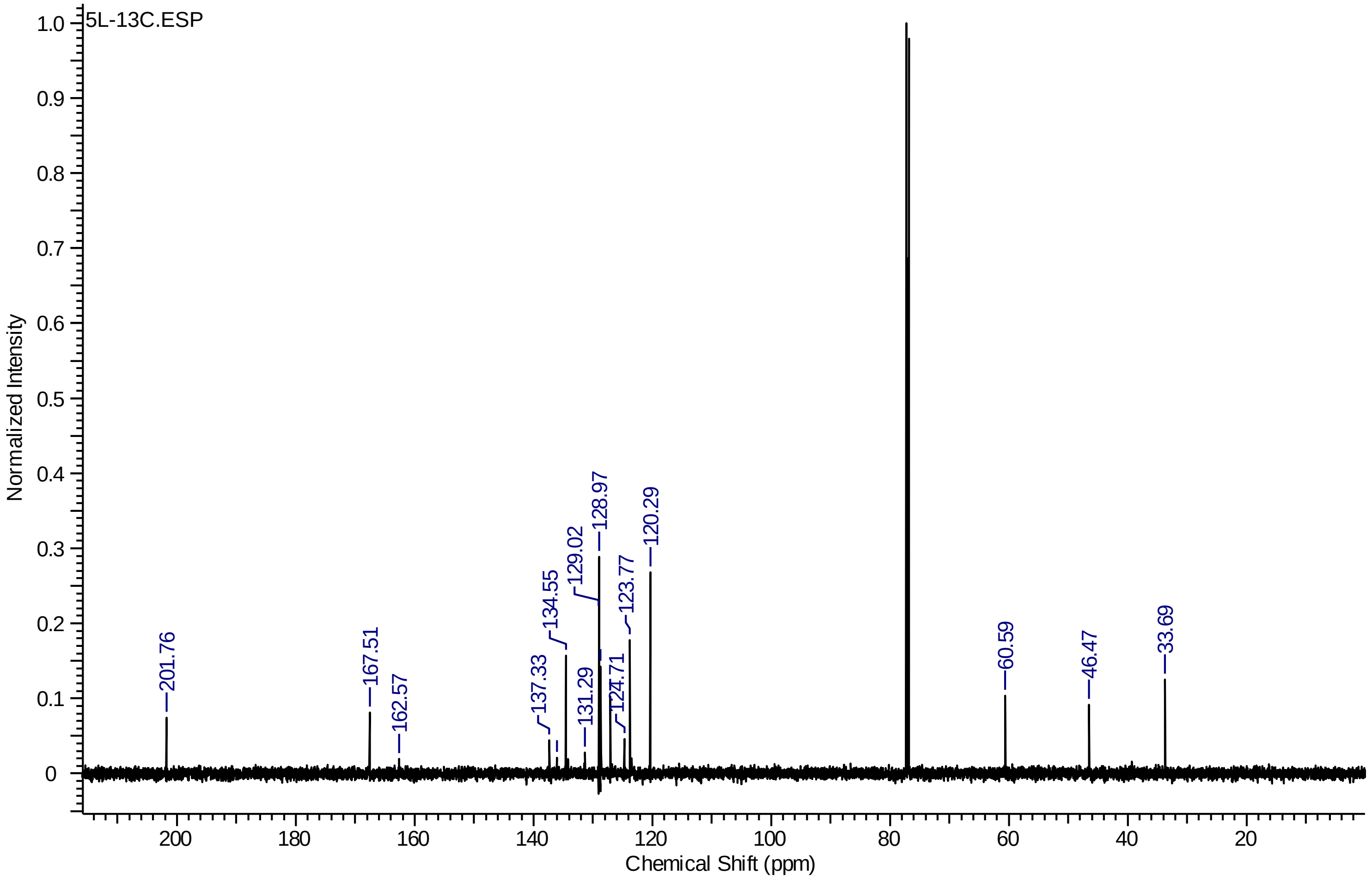

5L-13C.ESP
Normalized Intensity
Chemical Shift (ppm)
201.76
167.51
162.57
137.33
134.55
131.29
129.02
128.97
124.71
123.77
120.29
60.59
46.47
33.69

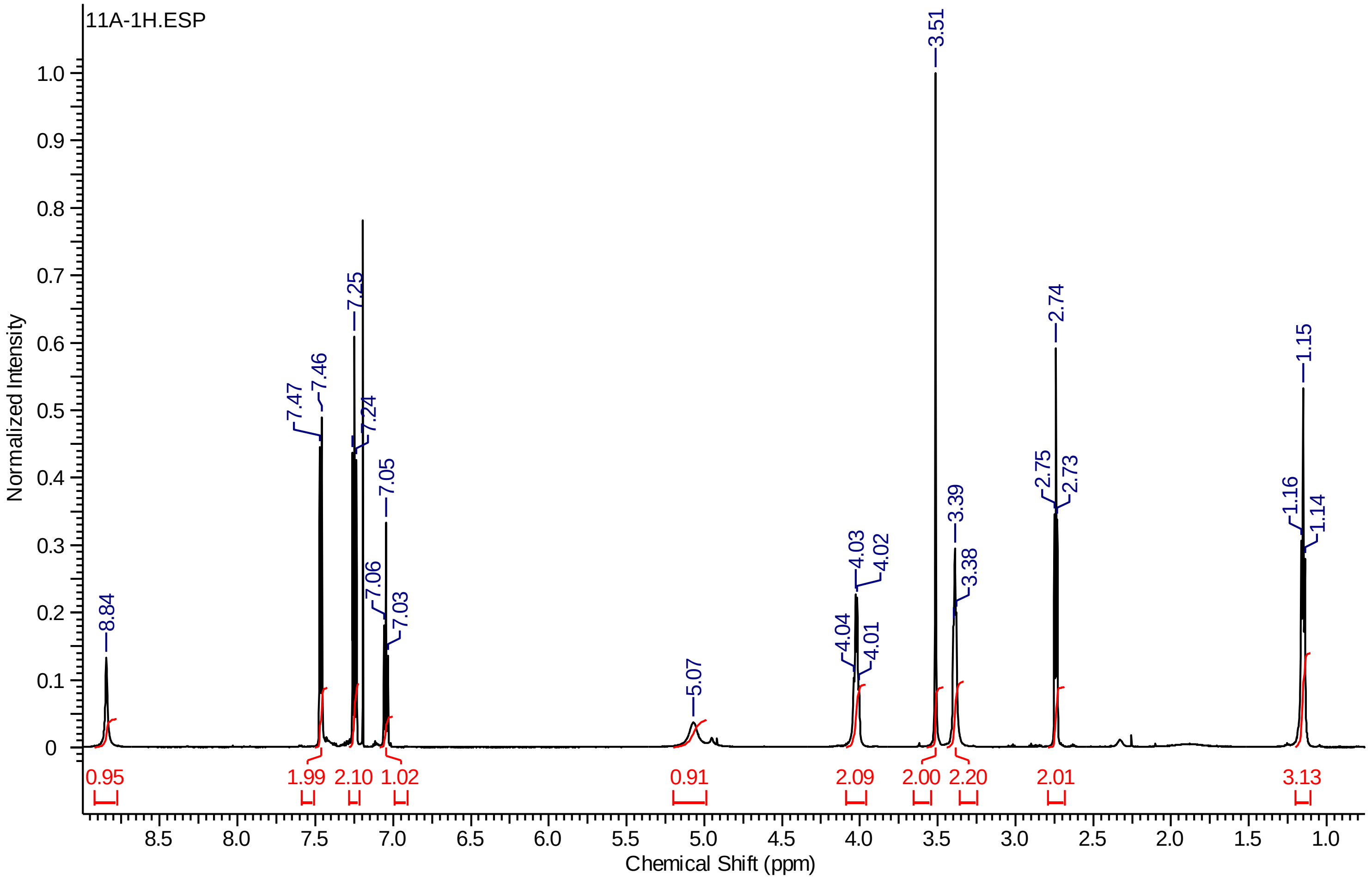

11A-1H.ESP
Normalized Intensity
Chemical Shift (ppm)
8.84
7.47
7.46
7.25
7.24
7.06
7.05
7.03
5.07
4.04
4.03
4.02
4.01
3.51
3.39
3.38
2.75
2.74
2.73
1.16
1.15
1.14
0.95
1.99
2.10
1.02
0.91
2.09
2.00
2.20
2.01
3.13

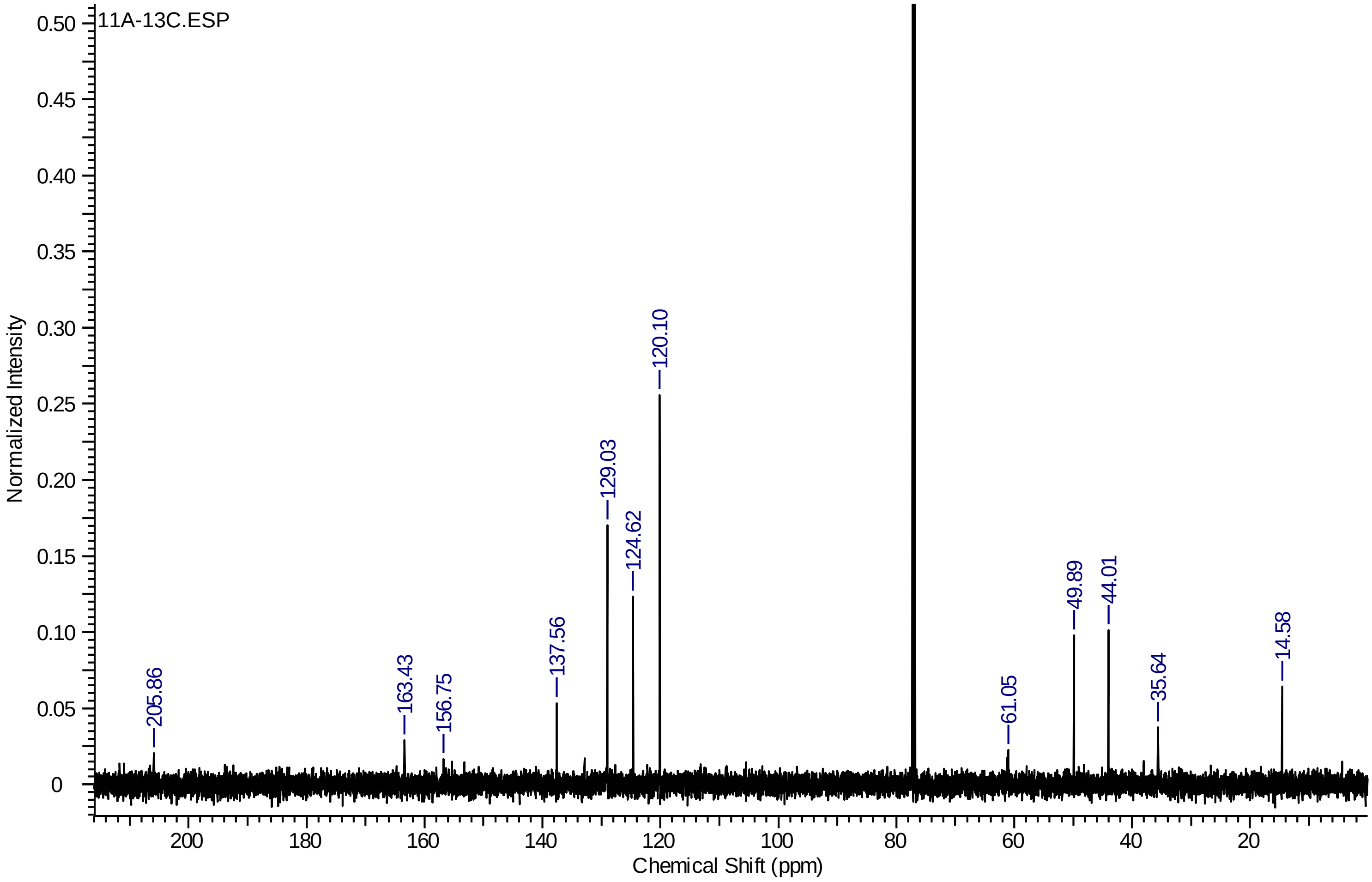

11A-13C.ESP
Normalized Intensity
Chemical Shift (ppm)
205.86
163.43
156.75
137.56
129.03
124.62
120.10
61.05
49.89
44.01
35.64
14.58

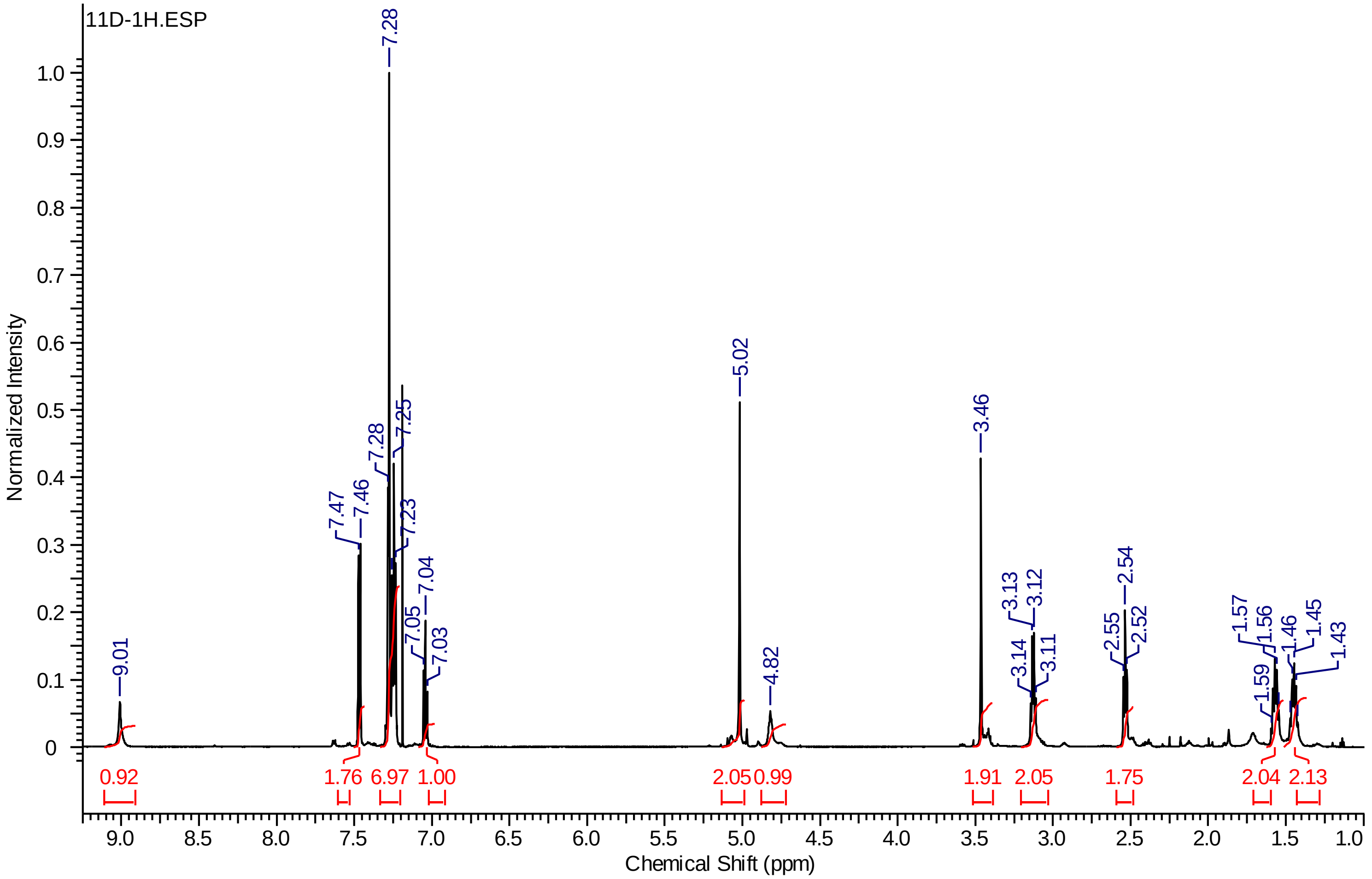
11D-1H.ESP
Normalized Intensity
Chemical Shift (ppm)
9.01
7.47
7.46
7.28
7.28
7.25
7.23
7.05
7.04
7.03
5.02
4.82
3.46
3.14
3.13
3.12
3.11
2.55
2.54
2.52
1.59
1.57
1.56
1.46
1.45
1.43
0.92
1.76
6.97
1.00
2.05
0.99
1.91
2.05
1.75
2.04
2.13

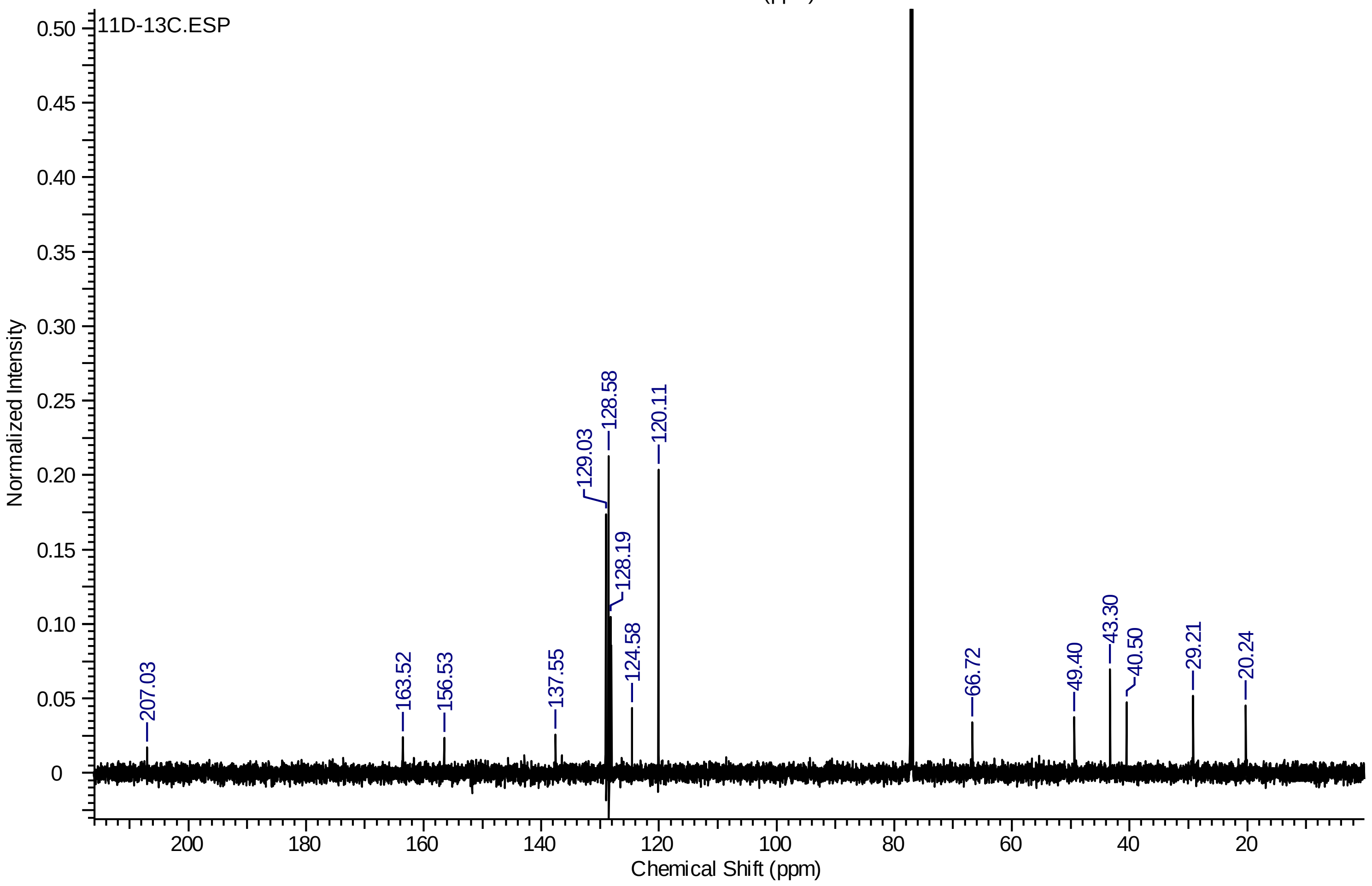
11D-13C.ESP
Normalized Intensity
Chemical Shift (ppm)
207.03
163.52
156.53
137.55
129.03
128.58
128.19
124.58
120.11
66.72
49.40
43.30
40.50
29.21
20.24

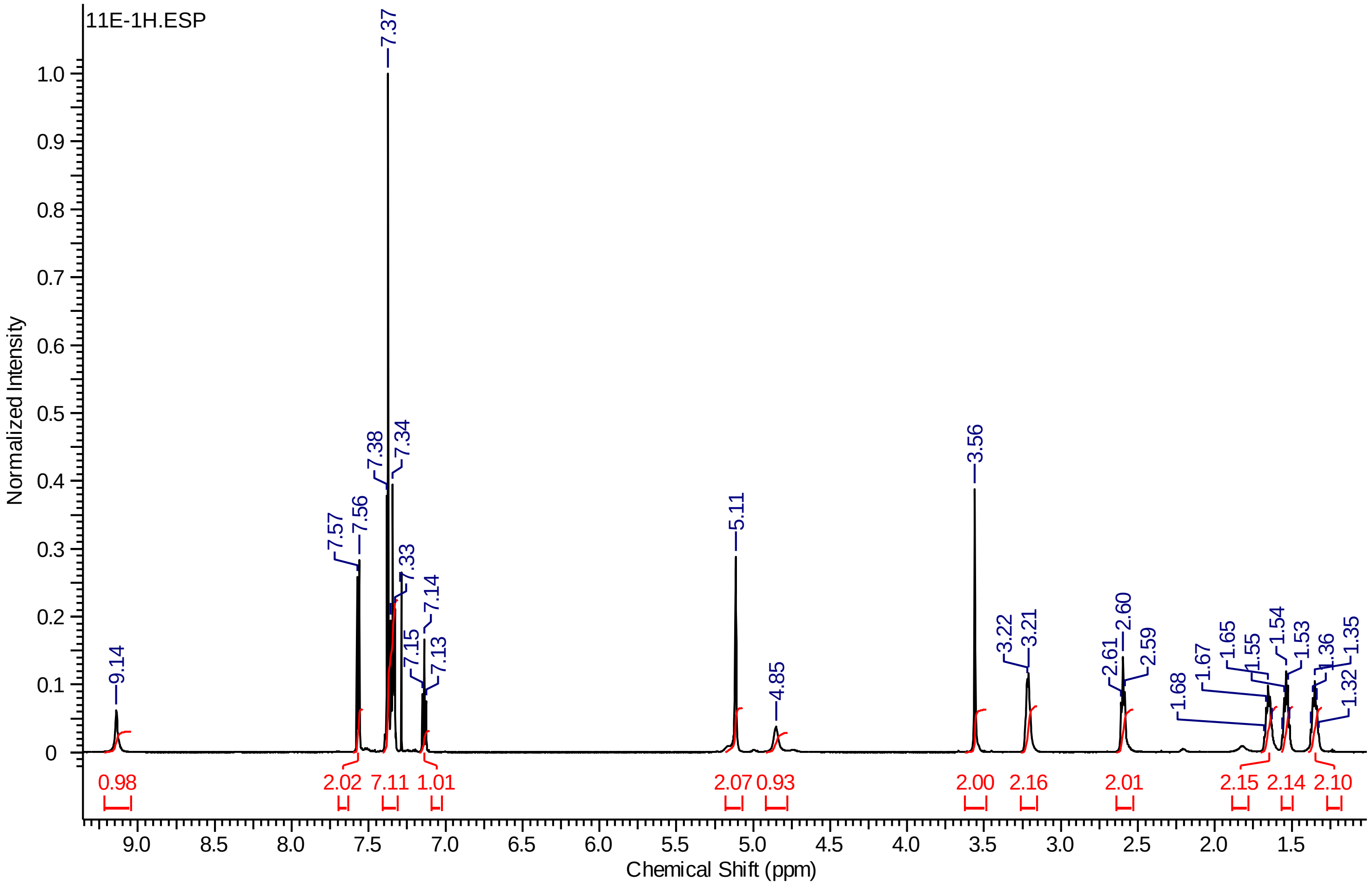
11E-1H.ESP
Normalized Intensity
Chemical Shift (ppm)
9.14
7.57
7.56
7.38
7.37
7.34
7.33
7.15
7.14
7.13
5.11
4.85
3.56
3.22
3.21
2.61
2.60
2.59
1.68
1.67
1.65
1.55
1.54
1.53
1.36
1.35
1.32
0.98
2.02
7.11
1.01
2.07
0.93
2.00
2.16
2.01
2.15
2.14
2.10

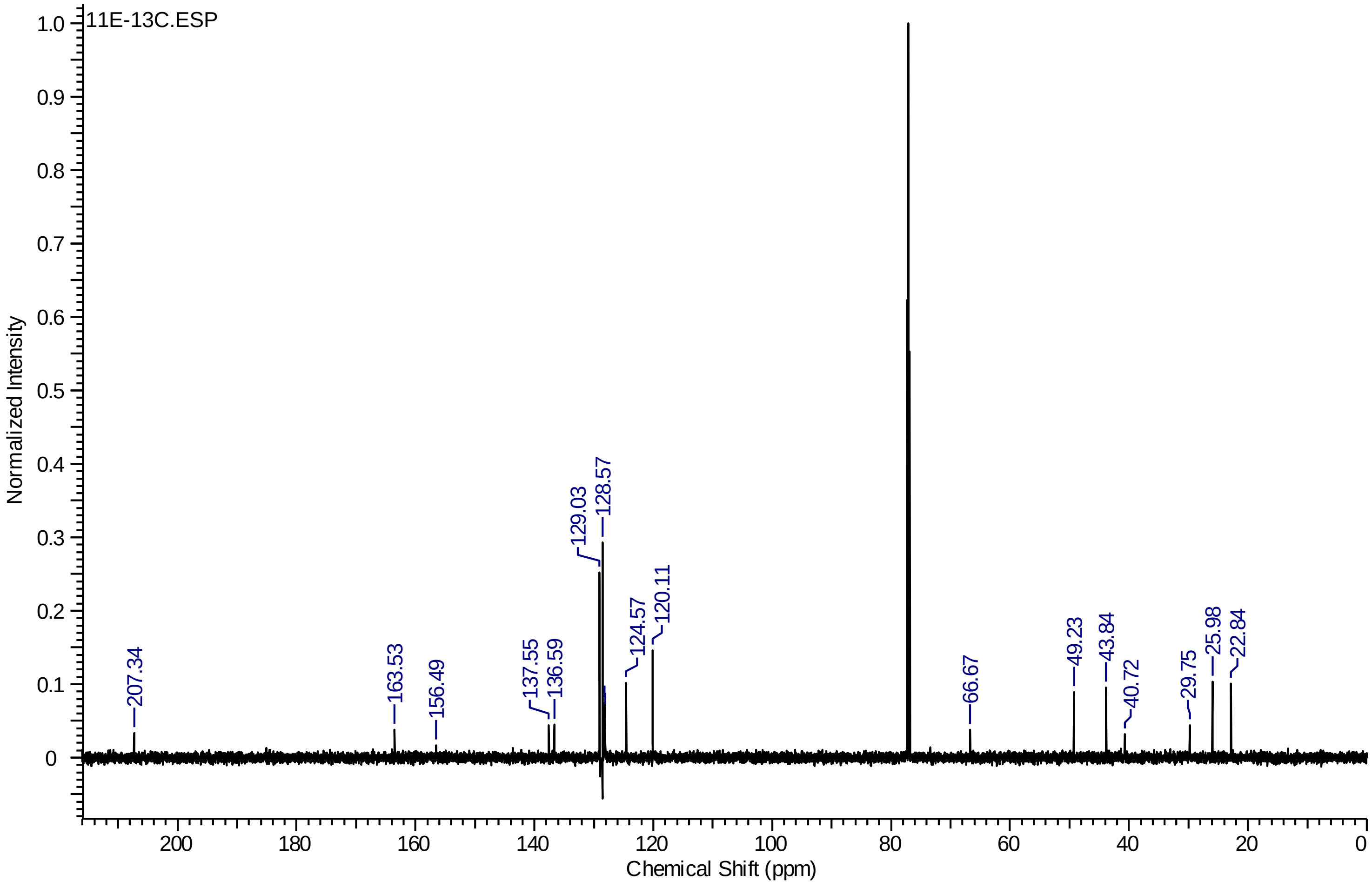
11E-13C.ESP
Normalized Intensity
Chemical Shift (ppm)
207.34
163.53
156.49
137.55
136.59
129.03
128.57
124.57
120.11
66.67
49.23
43.84
40.72
29.75
25.98
22.84

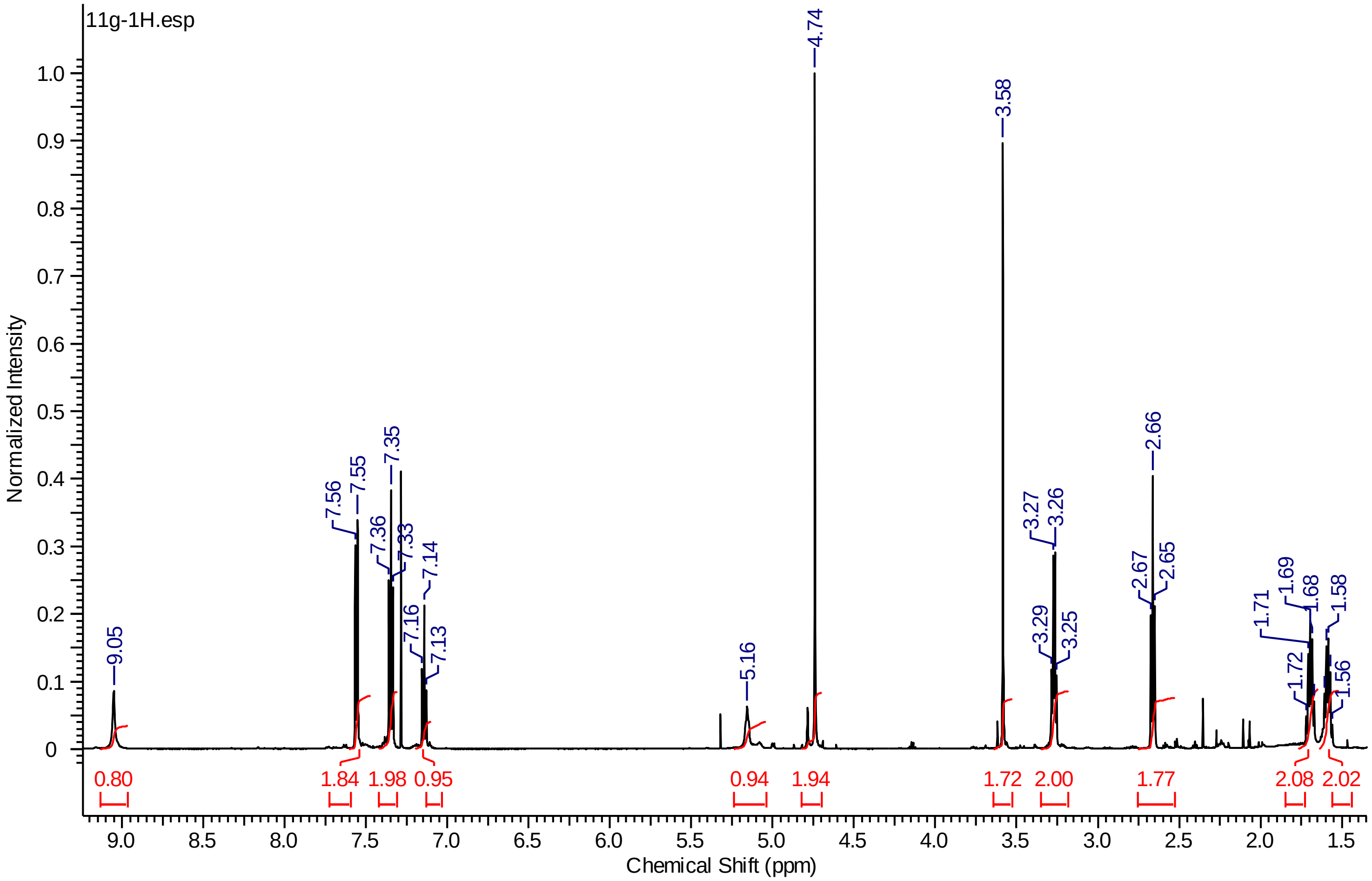
11g-1H.esp
Normalized Intensity
Chemical Shift (ppm)
9.05
7.56
7.55
7.36
7.35
7.33
7.16
7.14
7.13
5.16
4.74
3.58
3.29
3.27
3.26
3.25
2.67
2.66
2.65
1.72
1.71
1.69
1.68
1.58
1.56
0.80
1.84
1.98
0.95
0.94
1.94
1.72
2.00
1.77
2.08
2.02

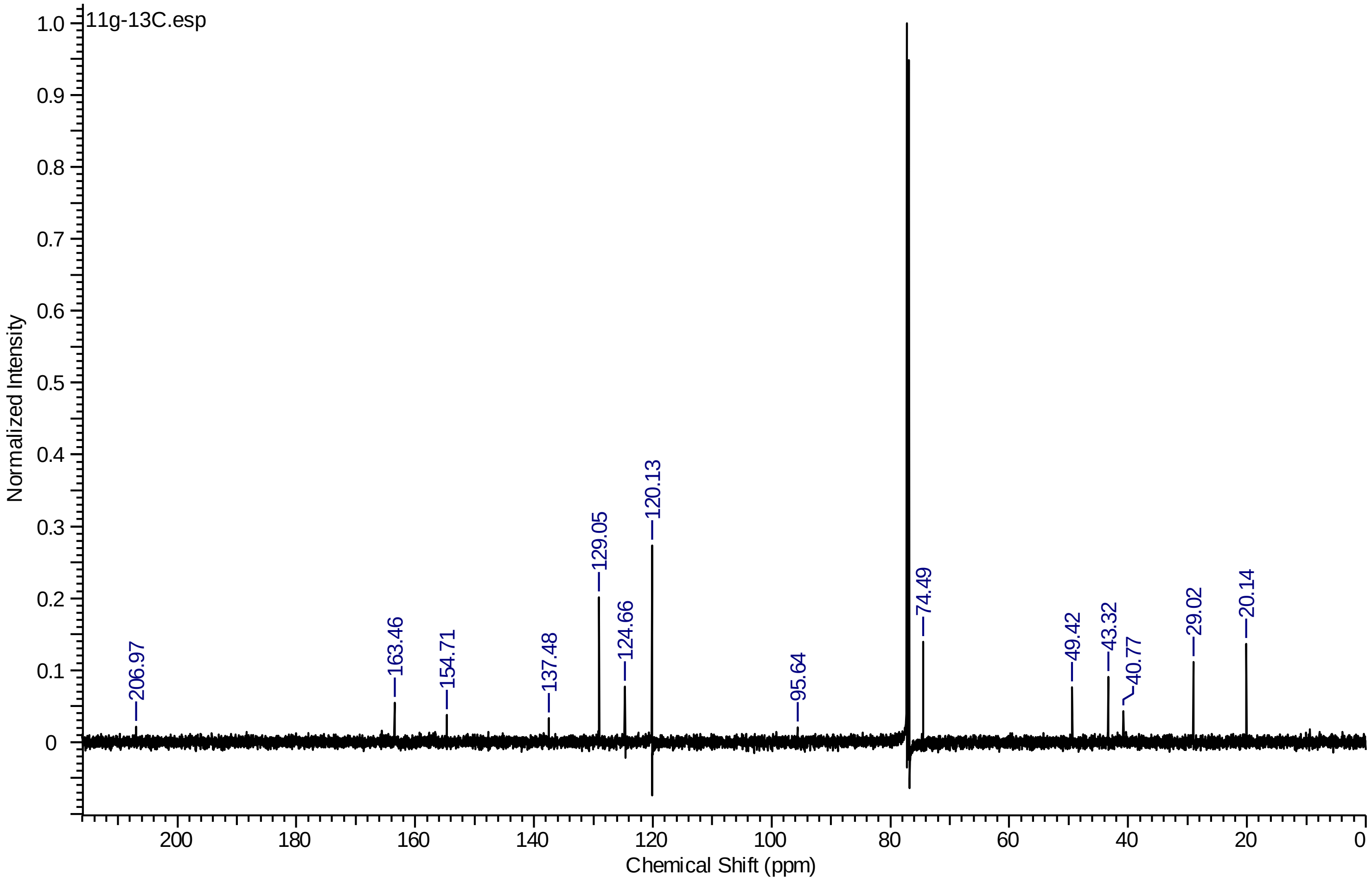
11g-13C.esp
Normalized Intensity
Chemical Shift (ppm)
206.97
163.46
154.71
137.48
129.05
124.66
120.13
95.64
74.49
49.42
43.32
40.77
29.02
20.14

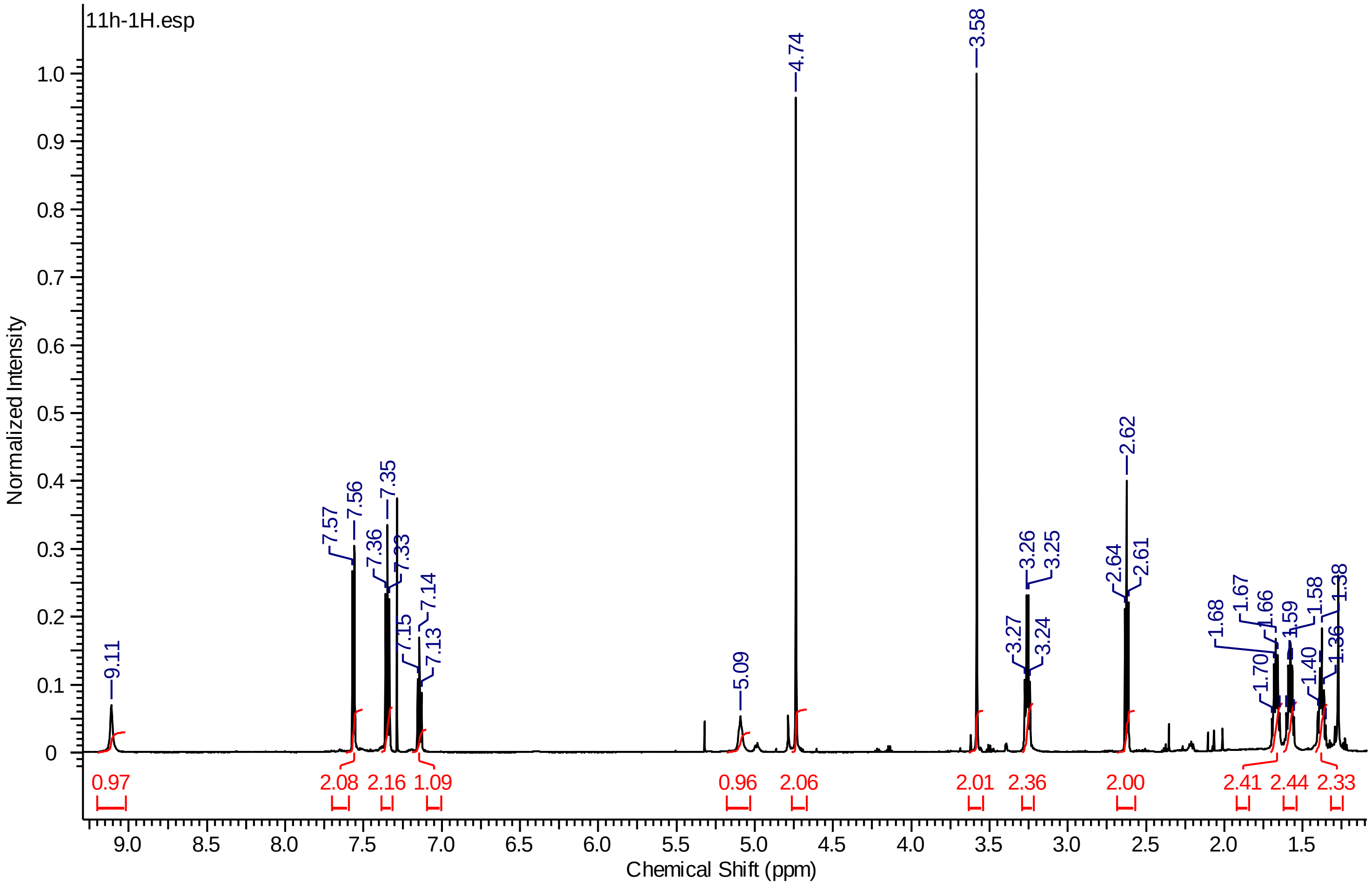
11h-1H.esp
Normalized Intensity
Chemical Shift (ppm)
9.11
7.57
7.56
7.36
7.35
7.33
7.15
7.14
7.13
5.09
4.74
3.58
3.27
3.26
3.25
3.24
2.64
2.62
2.61
1.70
1.68
1.67
1.66
1.59
1.58
1.40
1.38
1.36
0.97
2.08
2.16
1.09
0.96
2.06
2.01
2.36
2.00
2.41
2.44
2.33

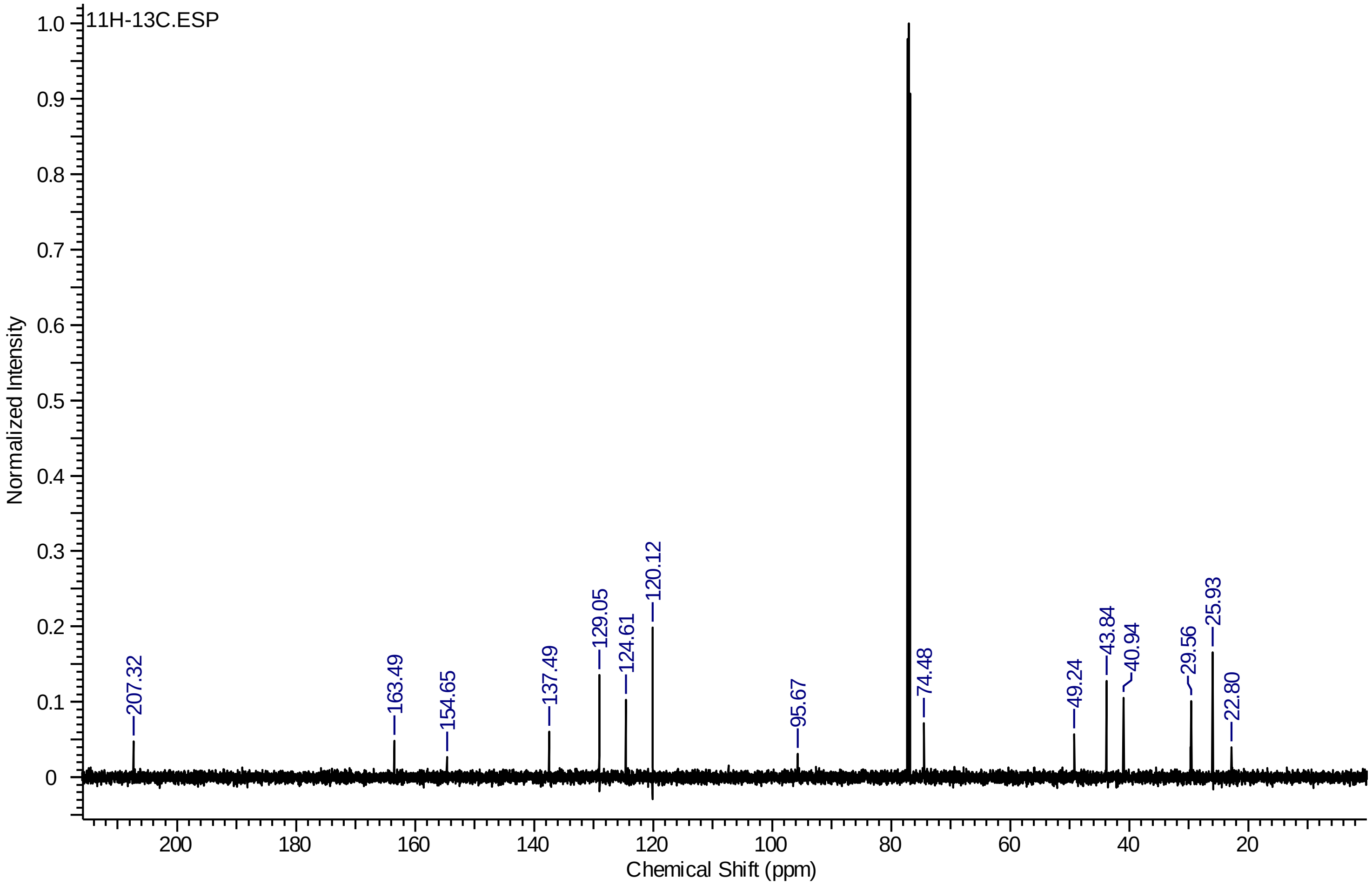
11H-13C.ESP
Normalized Intensity
Chemical Shift (ppm)
207.32
163.49
154.65
137.49
129.05
124.61
120.12
95.67
74.48
49.24
43.84
40.94
29.56
25.93
22.80

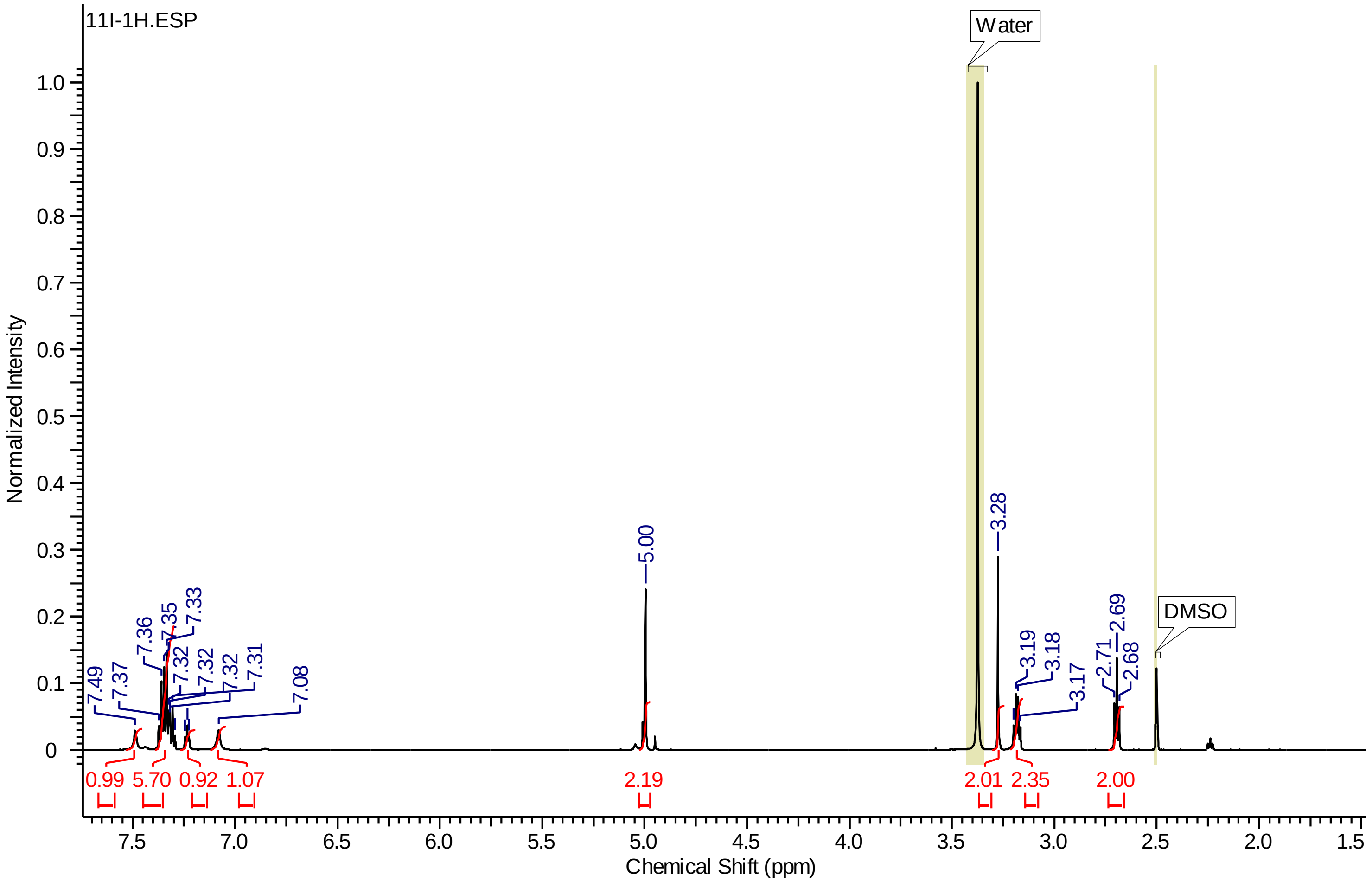
11I-1H.ESP
Water
DMSO
Normalized Intensity
Chemical Shift (ppm)
7.49
7.37
7.36
7.35
7.33
7.32
7.32
7.32
7.31
7.08
5.00
3.28
3.19
3.18
3.17
2.71
2.69
2.68
0.99
5.70
0.92
1.07
2.19
2.01
2.35
2.00

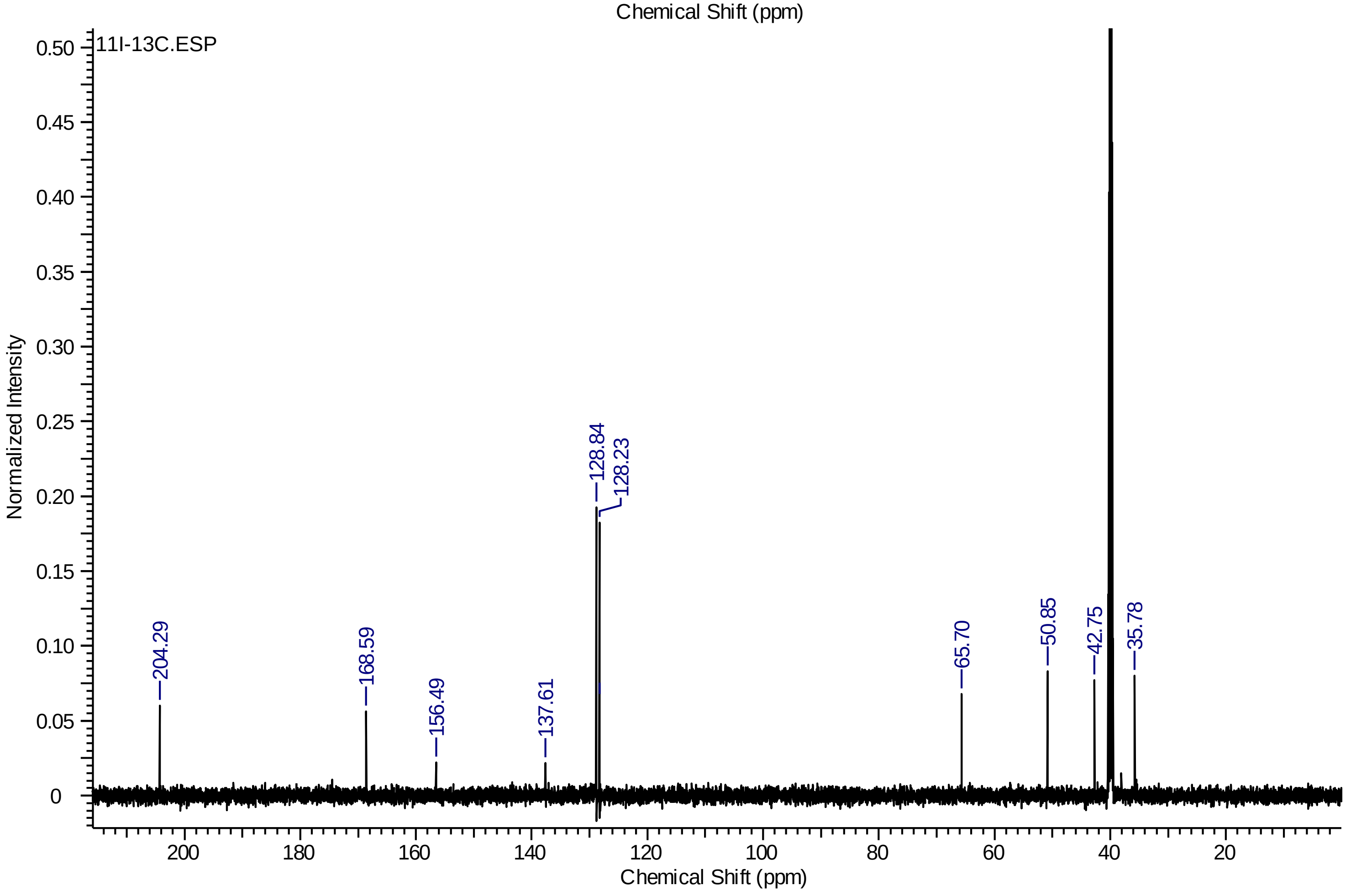
11I-13C.ESP
Normalized Intensity
Chemical Shift (ppm)
204.29
168.59
156.49
137.61
128.84
128.23
65.70
50.85
42.75
35.78

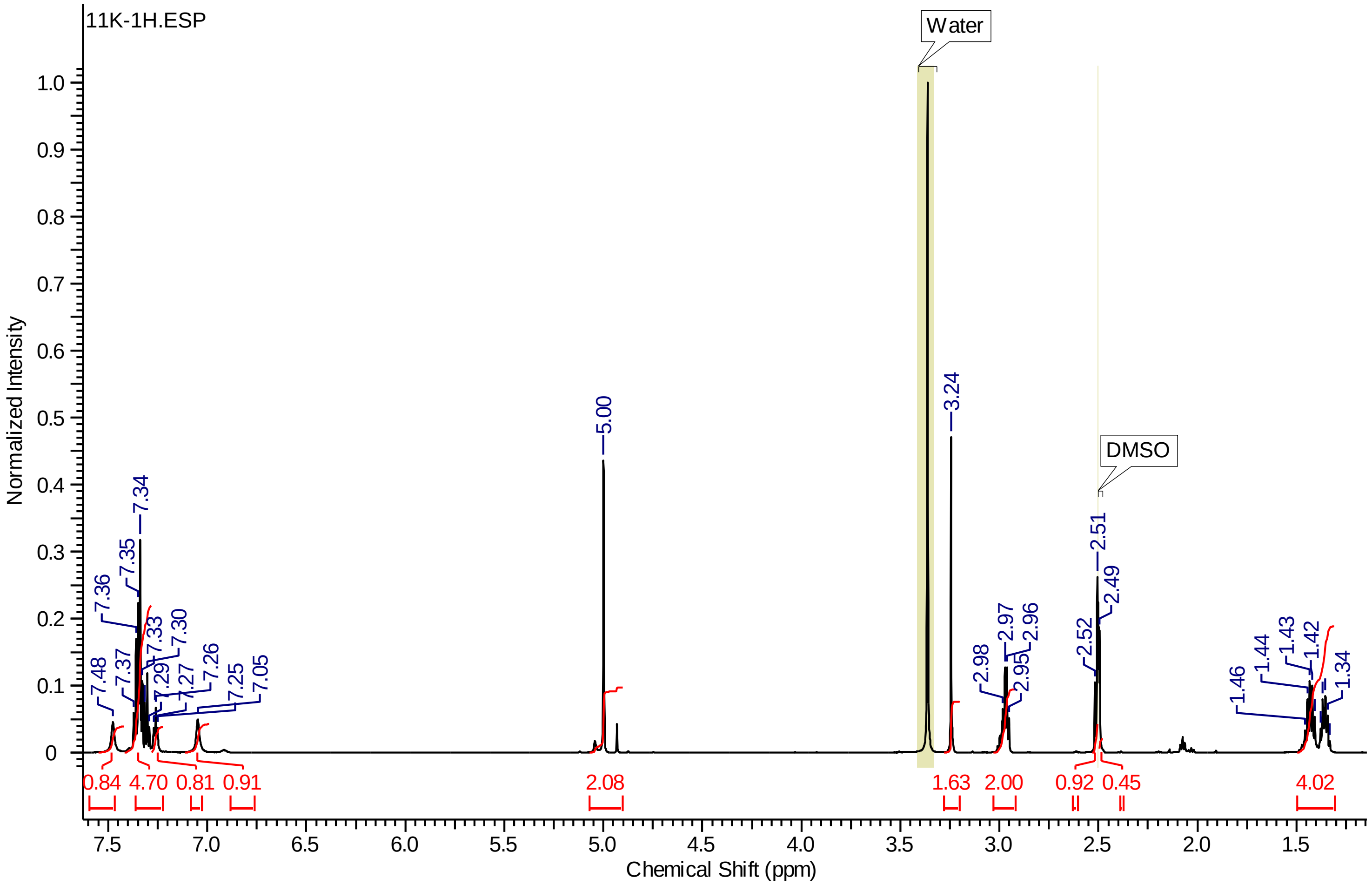
11K-1H.ESP
Water
DMSO
Normalized Intensity
Chemical Shift (ppm)
7.48
7.37
7.36
7.35
7.34
7.33
7.30
7.29
7.27
7.26
7.25
7.05
5.00
3.24
2.98
2.97
2.96
2.95
2.52
2.51
2.49
1.46
1.44
1.43
1.42
1.34
0.84
4.70
0.81
0.91
2.08
1.63
2.00
0.92
0.45
4.02

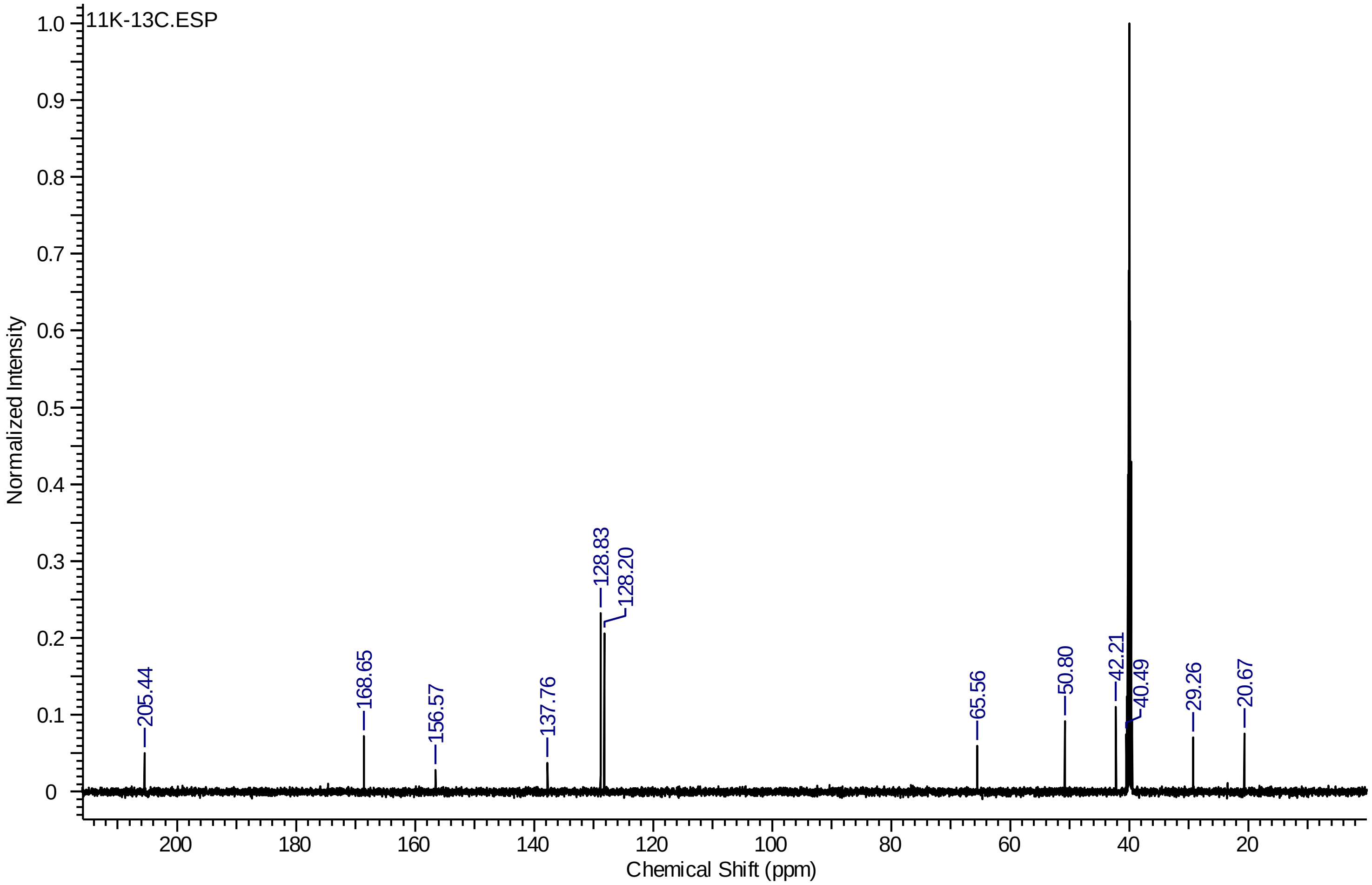
11K-13C.ESP
Normalized Intensity
Chemical Shift (ppm)
205.44
168.65
156.57
137.76
128.83
128.20
65.56
50.80
42.21
40.49
29.26
20.67

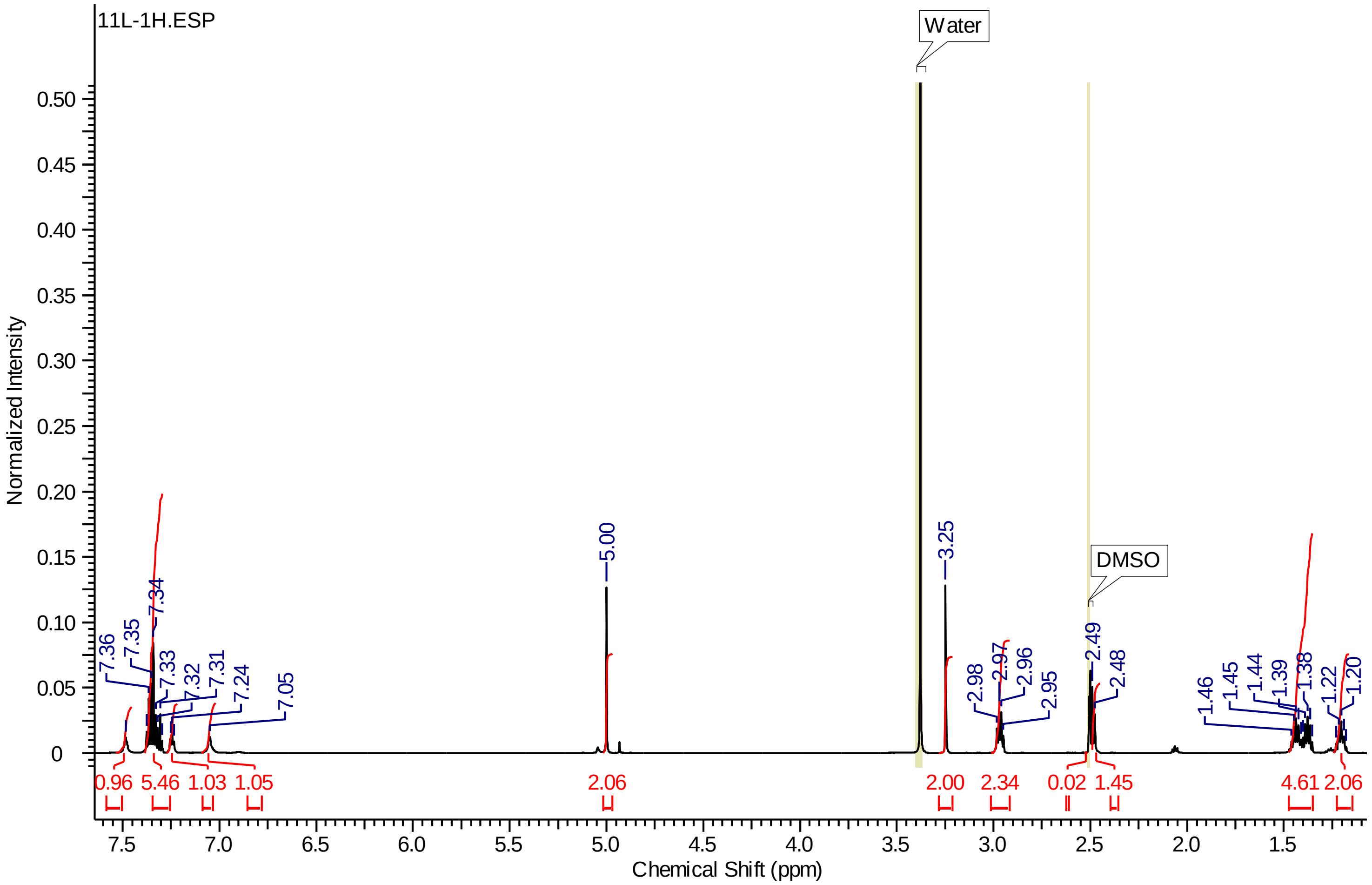
11L-1H.ESP
Water
DMSO
7.36
7.35
7.34
7.33
7.32
7.31
7.24
7.05
5.00
3.25
2.98
2.97
2.96
2.95
2.49
2.48
1.46
1.45
1.44
1.39
1.38
1.22
1.20
0.96
5.46
1.03
1.05
2.06
2.00
2.34
0.02
1.45
4.61
2.06
Normalized Intensity
Chemical Shift (ppm)

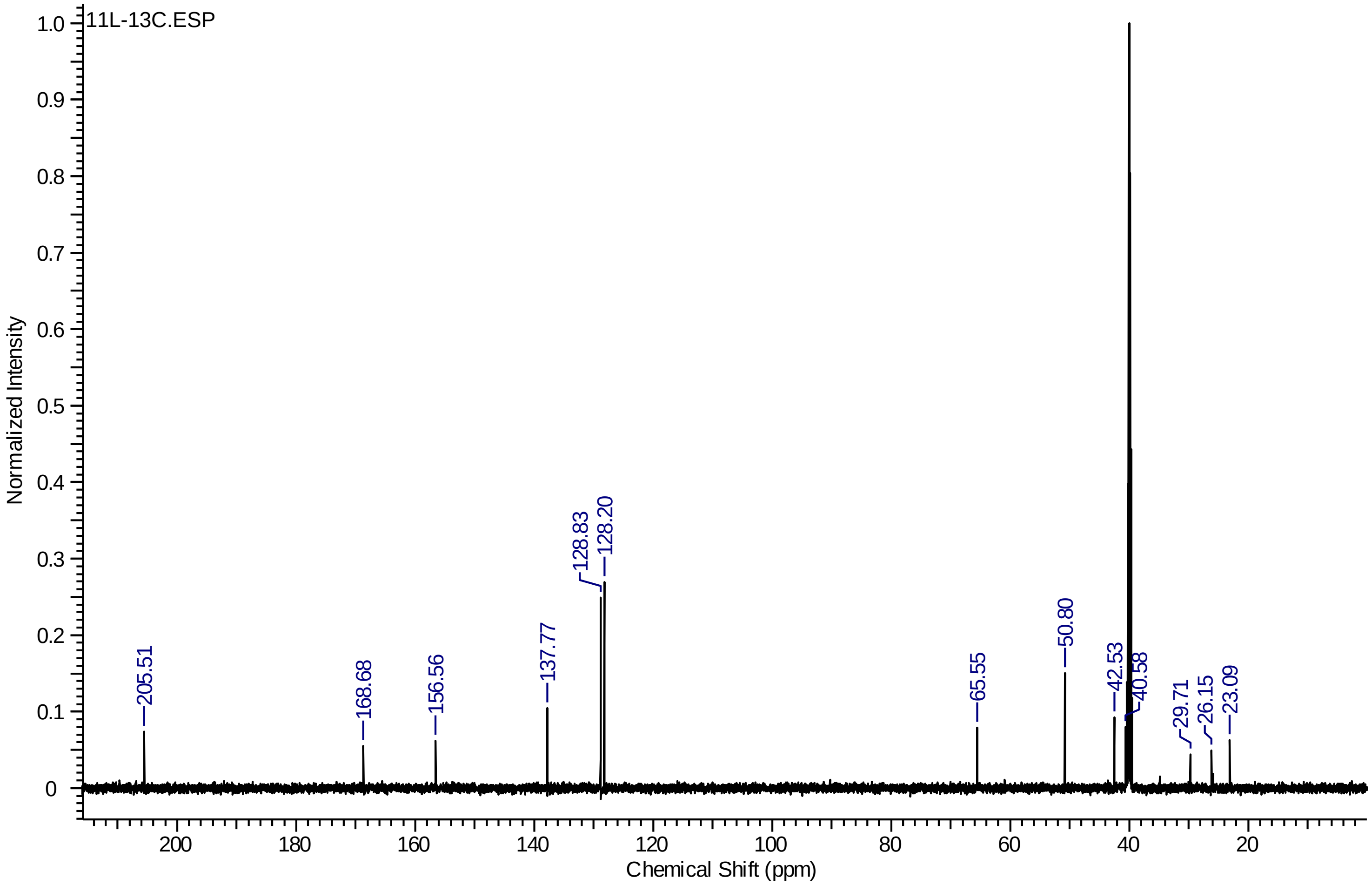
11L-13C.ESP
205.51
168.68
156.56
137.77
128.83
128.20
65.55
50.80
42.53
40.58
29.71
26.15
23.09
Normalized Intensity
Chemical Shift (ppm)

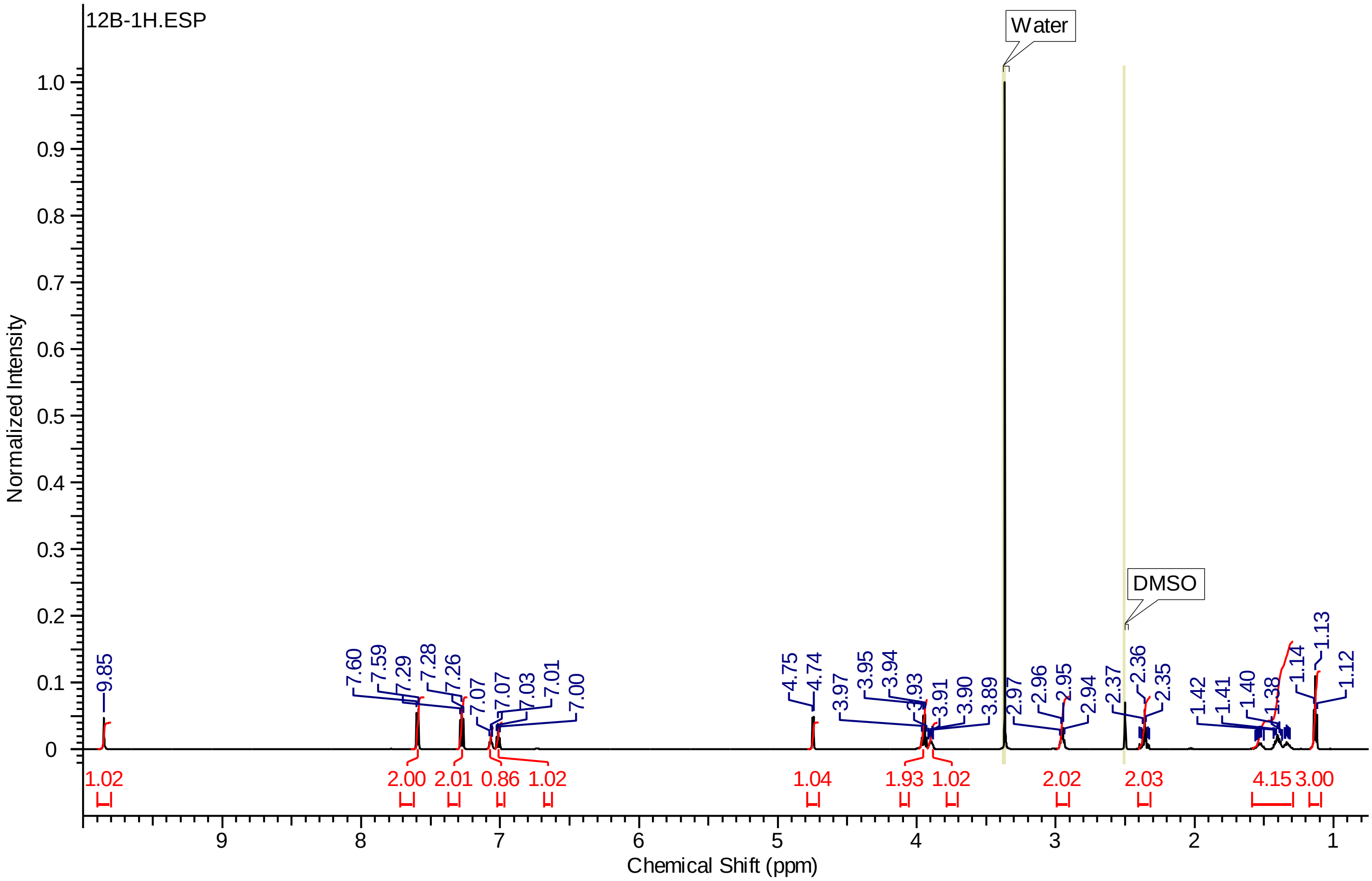
12B-1H.ESP
Water
DMSO
Normalized Intensity
Chemical Shift (ppm)
9.85
7.60
7.59
7.29
7.28
7.26
7.07
7.07
7.03
7.01
7.00
4.75
4.74
3.97
3.95
3.94
3.93
3.91
3.90
3.89
2.97
2.96
2.95
2.94
2.37
2.36
2.35
1.42
1.41
1.40
1.38
1.14
1.13
1.12
1.02
2.00
2.01
0.86
1.02
1.04
1.93
1.02
2.02
2.03
4.15
3.00

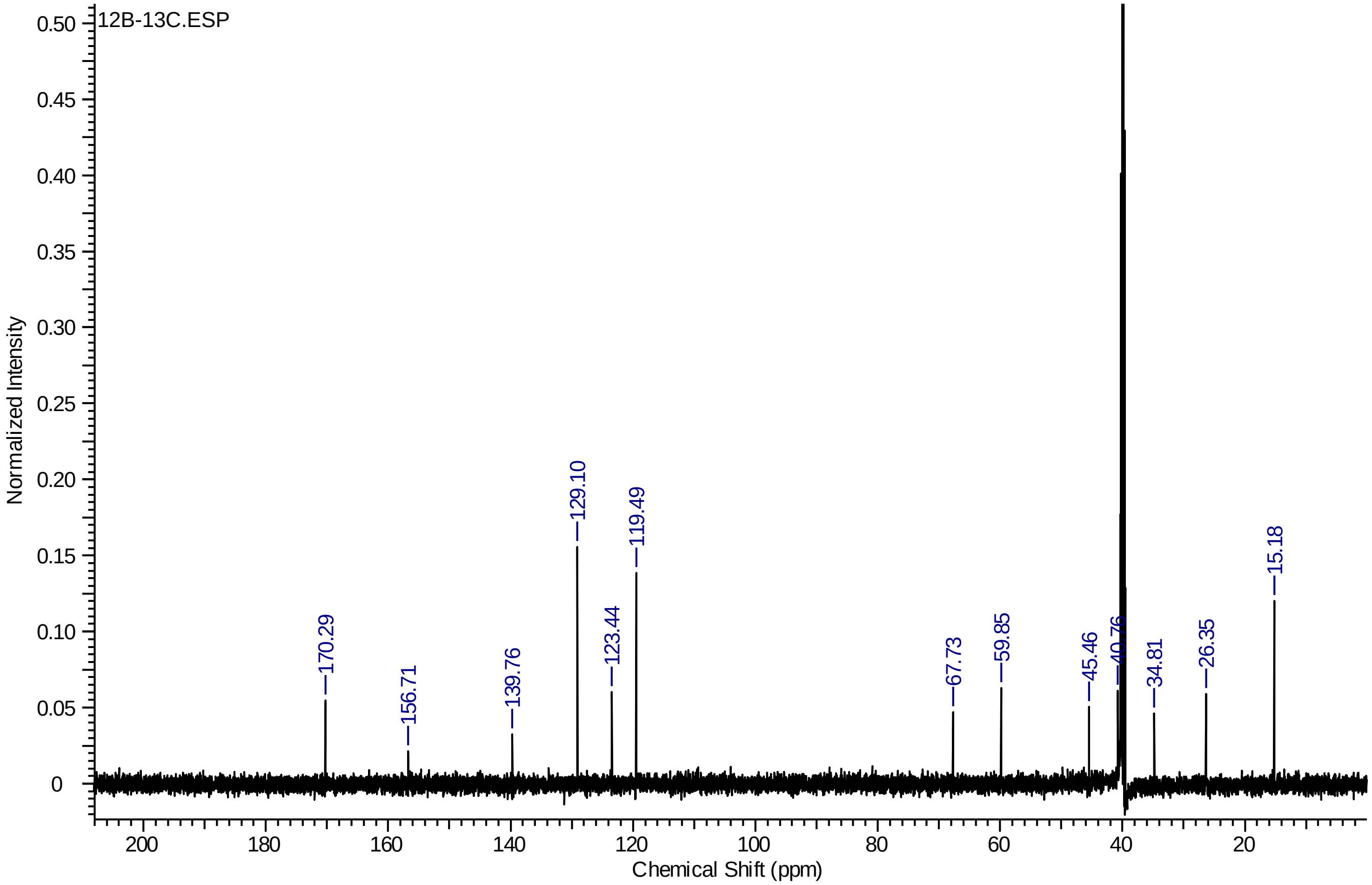
12B-13C.ESP
Normalized Intensity
Chemical Shift (ppm)
170.29
156.71
139.76
129.10
123.44
119.49
67.73
59.85
45.46
40.76
34.81
26.35
15.18

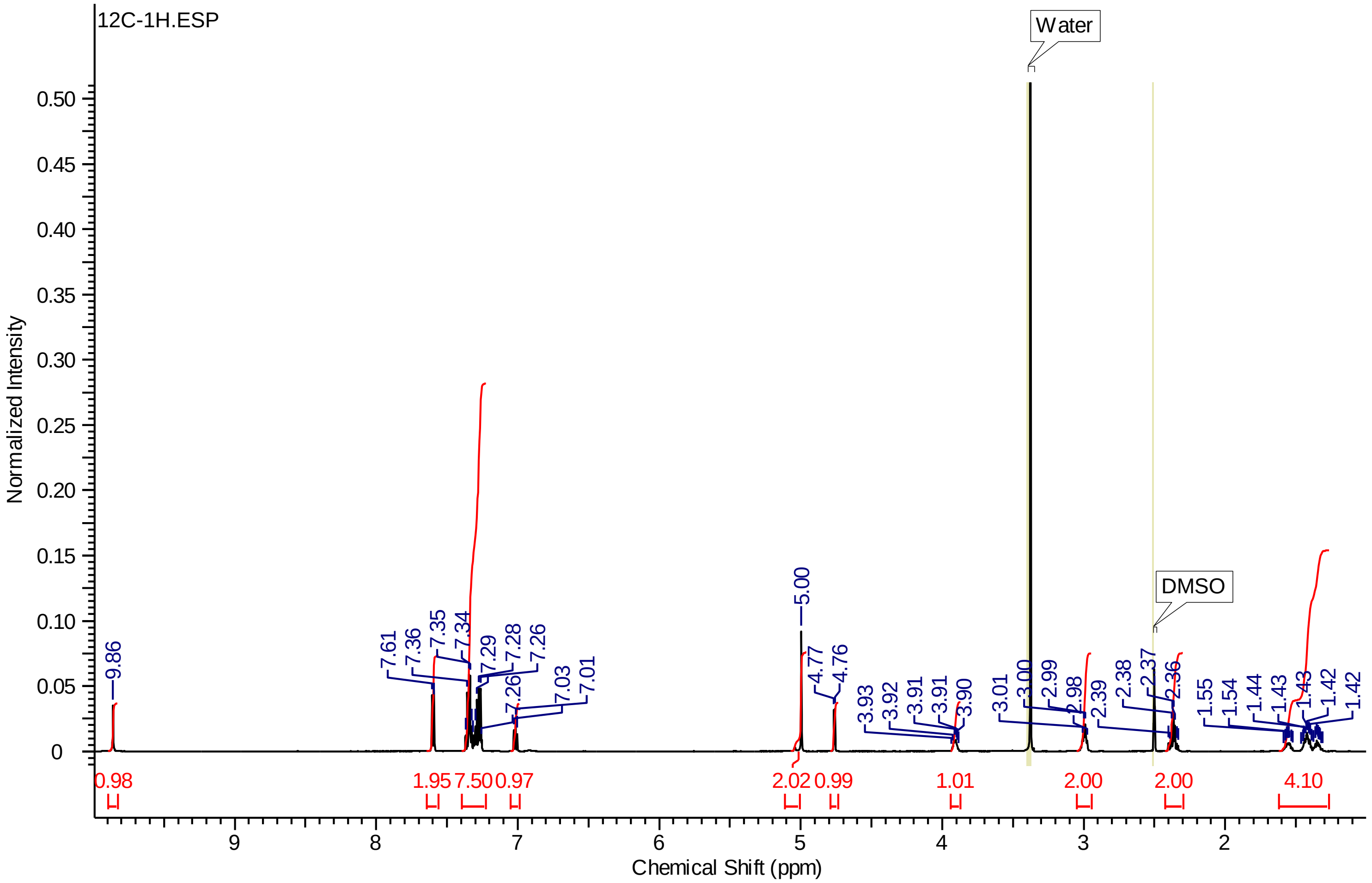
12C-1H.ESP
Water
DMSO
Normalized Intensity
Chemical Shift (ppm)
9.86
7.61
7.36
7.35
7.34
7.29
7.28
7.26
7.26
7.03
7.01
5.00
4.77
4.76
3.93
3.92
3.91
3.91
3.90
3.01
3.00
2.99
2.98
2.39
2.38
2.37
2.36
1.55
1.54
1.44
1.43
1.43
1.42
1.42
0.98
1.95
7.50
0.97
2.02
0.99
1.01
2.00
2.00
4.10

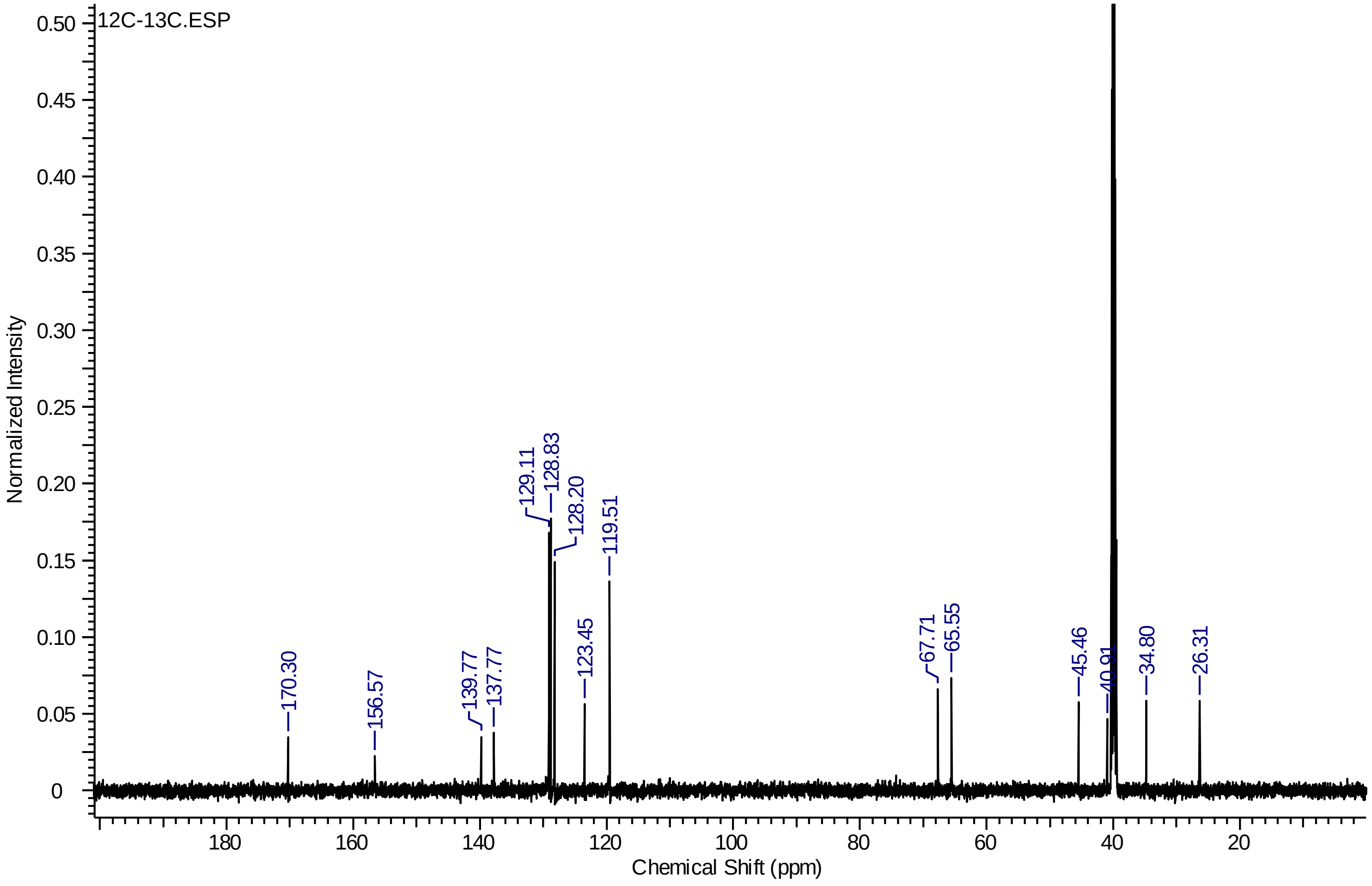
12C-13C.ESP
Normalized Intensity
Chemical Shift (ppm)
170.30
156.57
139.77
137.77
129.11
128.83
128.20
123.45
119.51
67.71
65.55
45.46
40.91
34.80
26.31

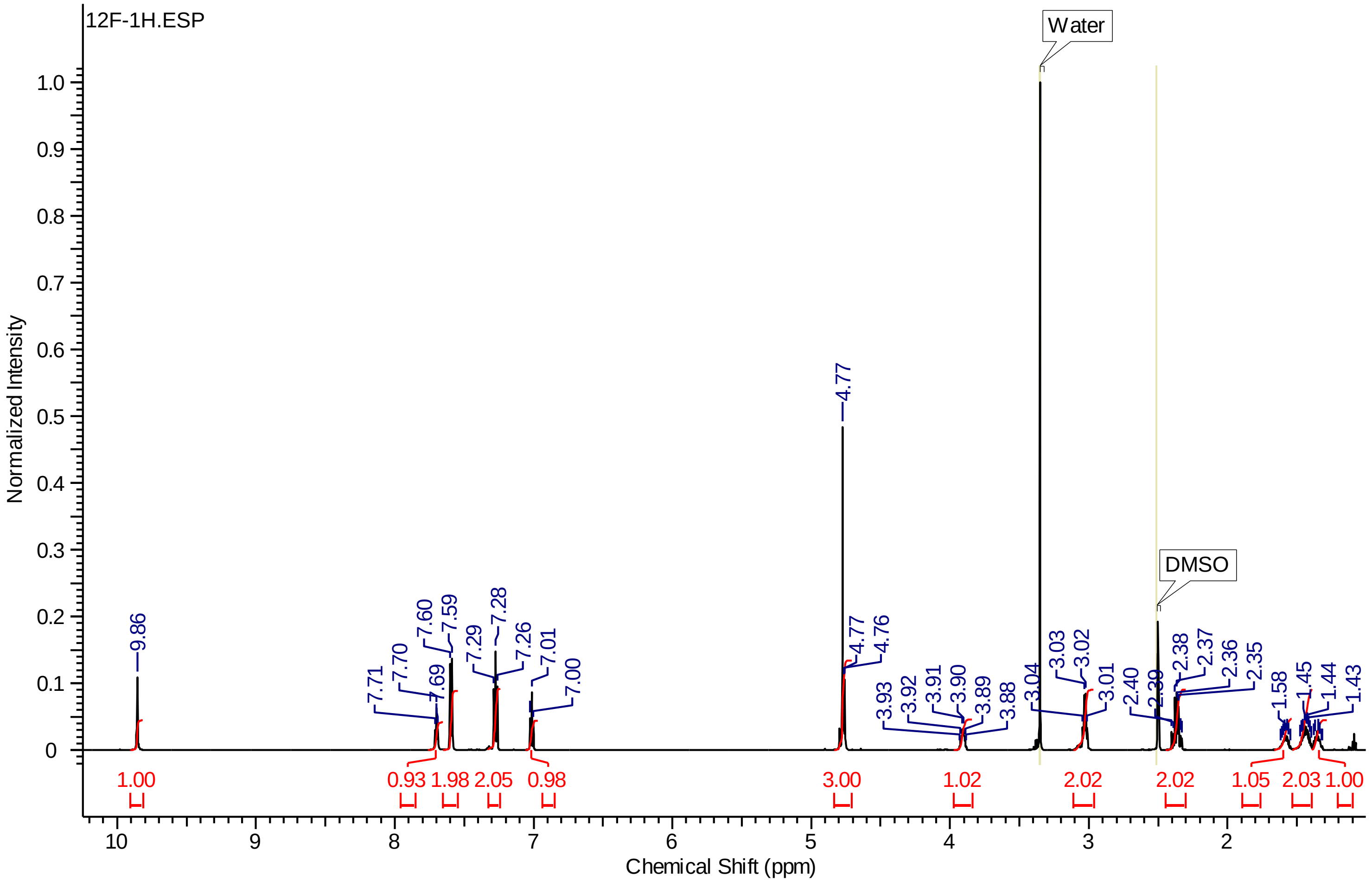
12F-1H.ESP
Water
DMSO
Normalized Intensity
Chemical Shift (ppm)
9.86
7.71 7.70 7.69 7.60 7.59 7.29 7.28 7.26 7.01 7.00
4.77 4.77 4.76
3.93 3.92 3.91 3.90 3.89 3.88
3.04 3.03 3.02 3.01
2.40 2.39 2.38 2.37 2.36 2.35
1.58 1.45 1.44 1.43
1.00 0.93 1.98 2.05 0.98 3.00 1.02 2.02 2.02 1.05 2.03 1.00

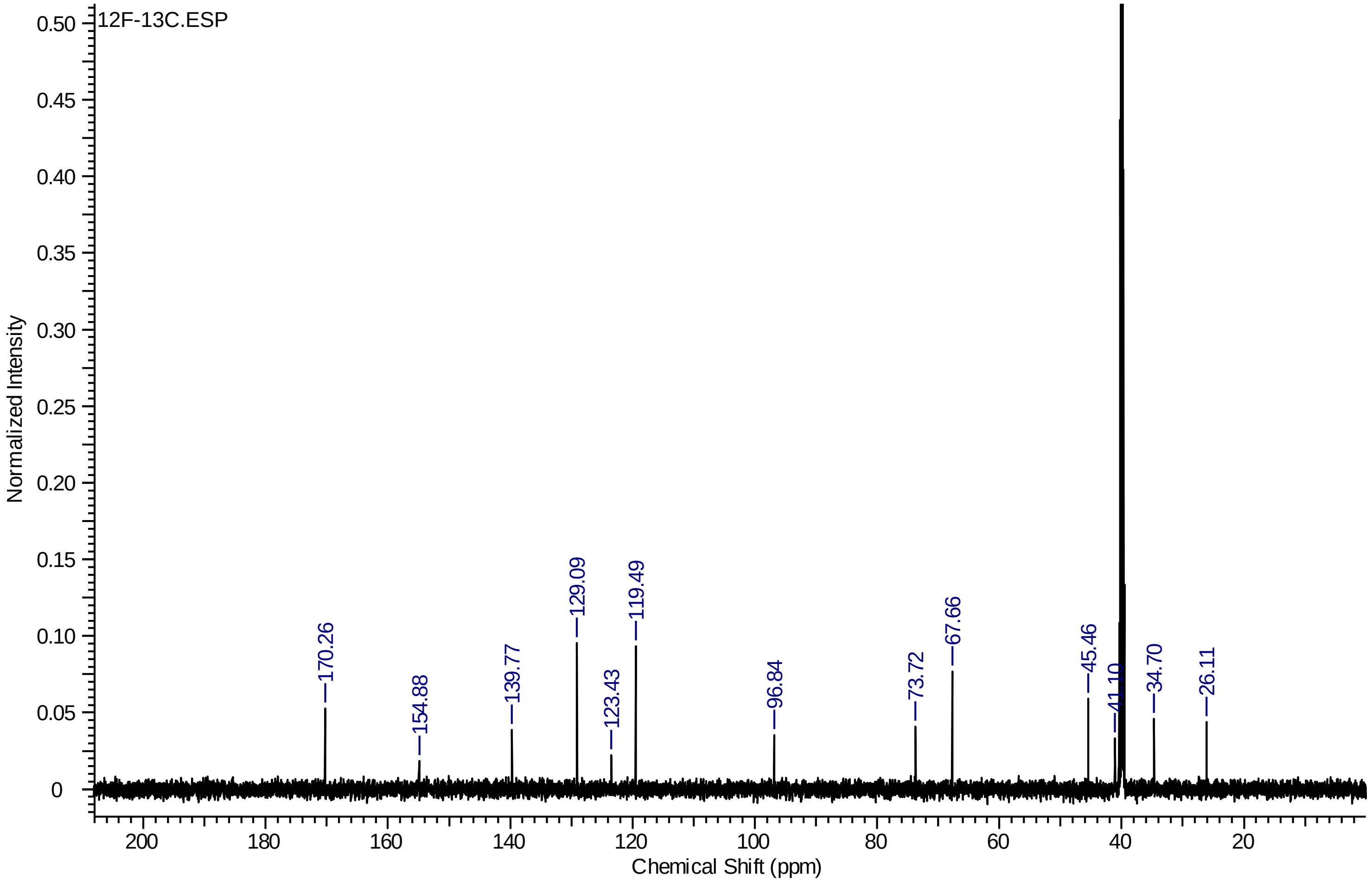
12F-13C.ESP
Normalized Intensity
Chemical Shift (ppm)
170.26
154.88
139.77
129.09
123.43
119.49
96.84
73.72
67.66
45.46
41.10
34.70
26.11

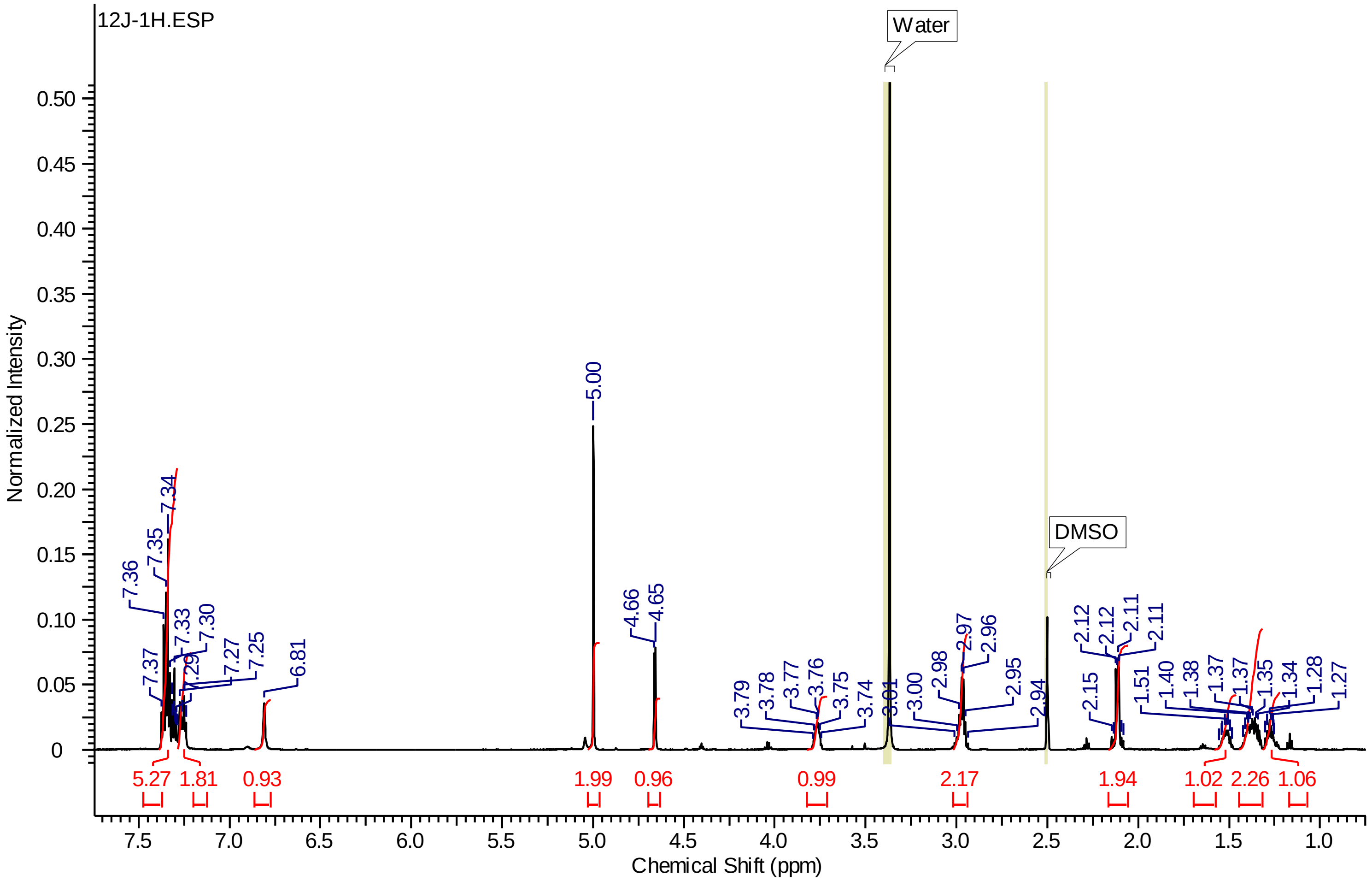
12J-1H.ESP
Water
DMSO
Normalized Intensity
Chemical Shift (ppm)

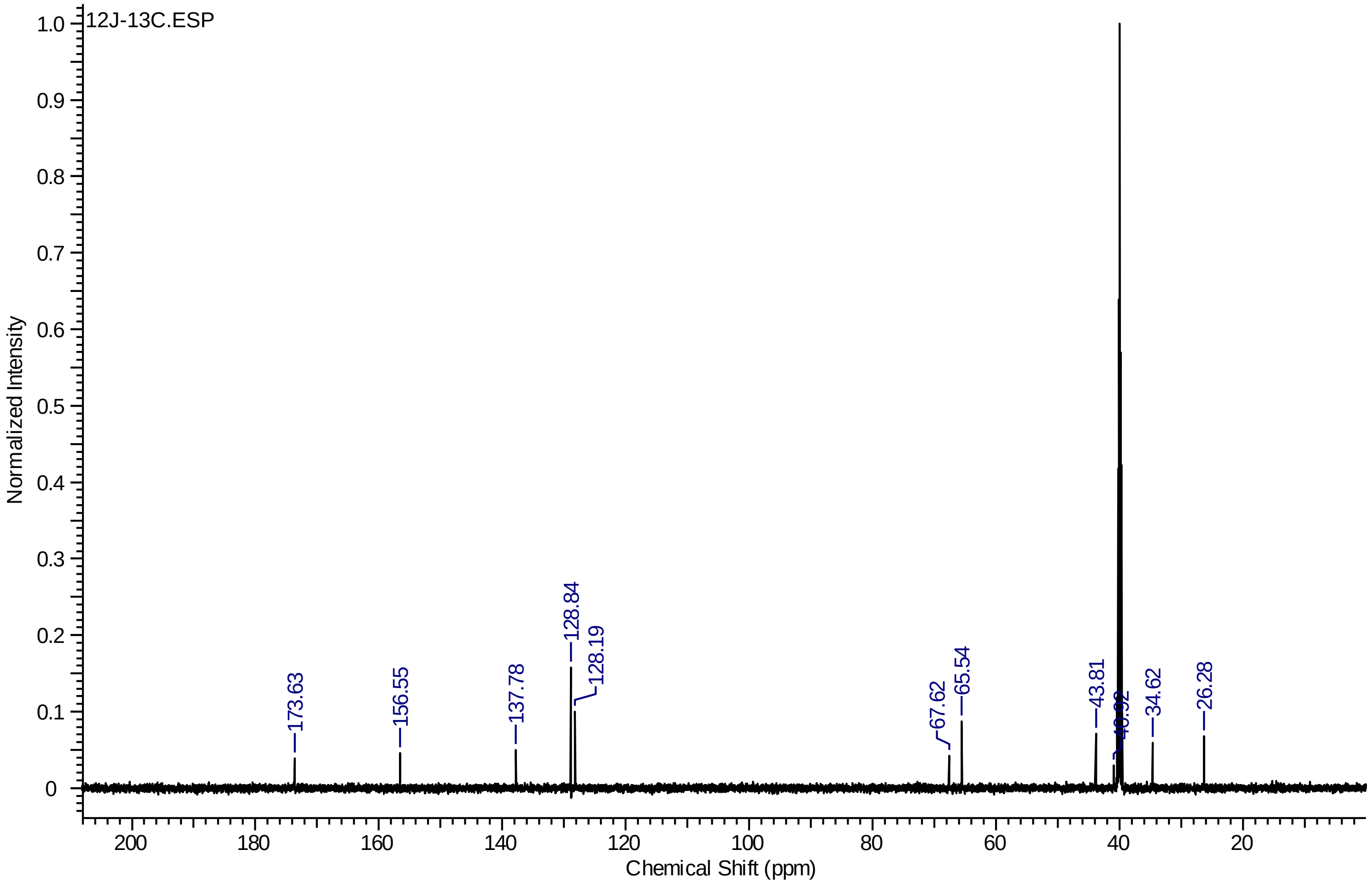
12J-13C.ESP
173.63
156.55
137.78
128.84
128.19
67.62
65.54
43.81
34.62
26.28
Normalized Intensity
Chemical Shift (ppm)

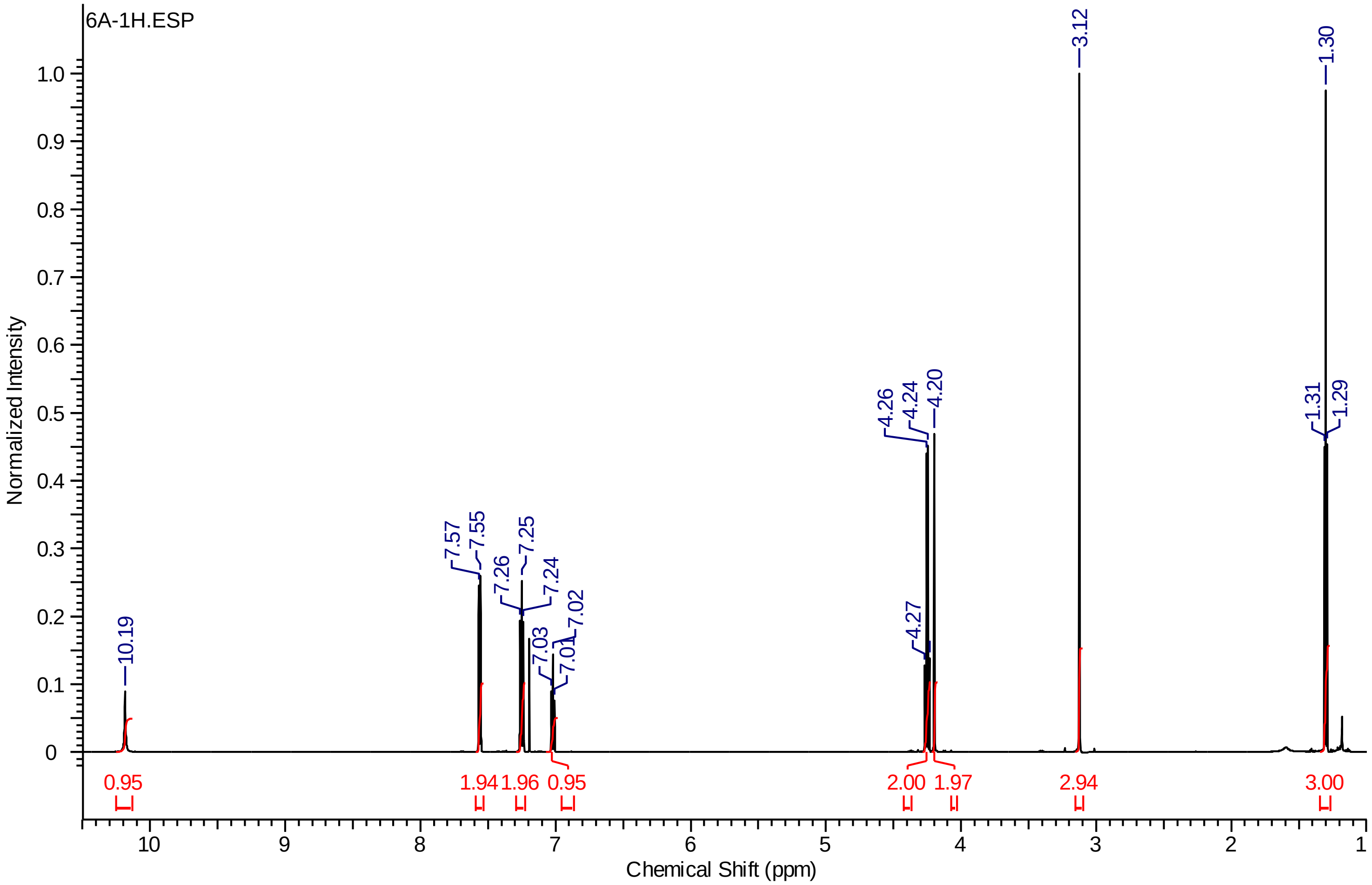
6A-1H.ESP
Normalized Intensity
Chemical Shift (ppm)
10.19
7.57
7.55
7.26
7.25
7.24
7.03
7.02
7.01
4.27
4.26
4.24
4.20
3.12
1.31
1.30
1.29
0.95
1.94
1.96
0.95
2.00
1.97
2.94
3.00

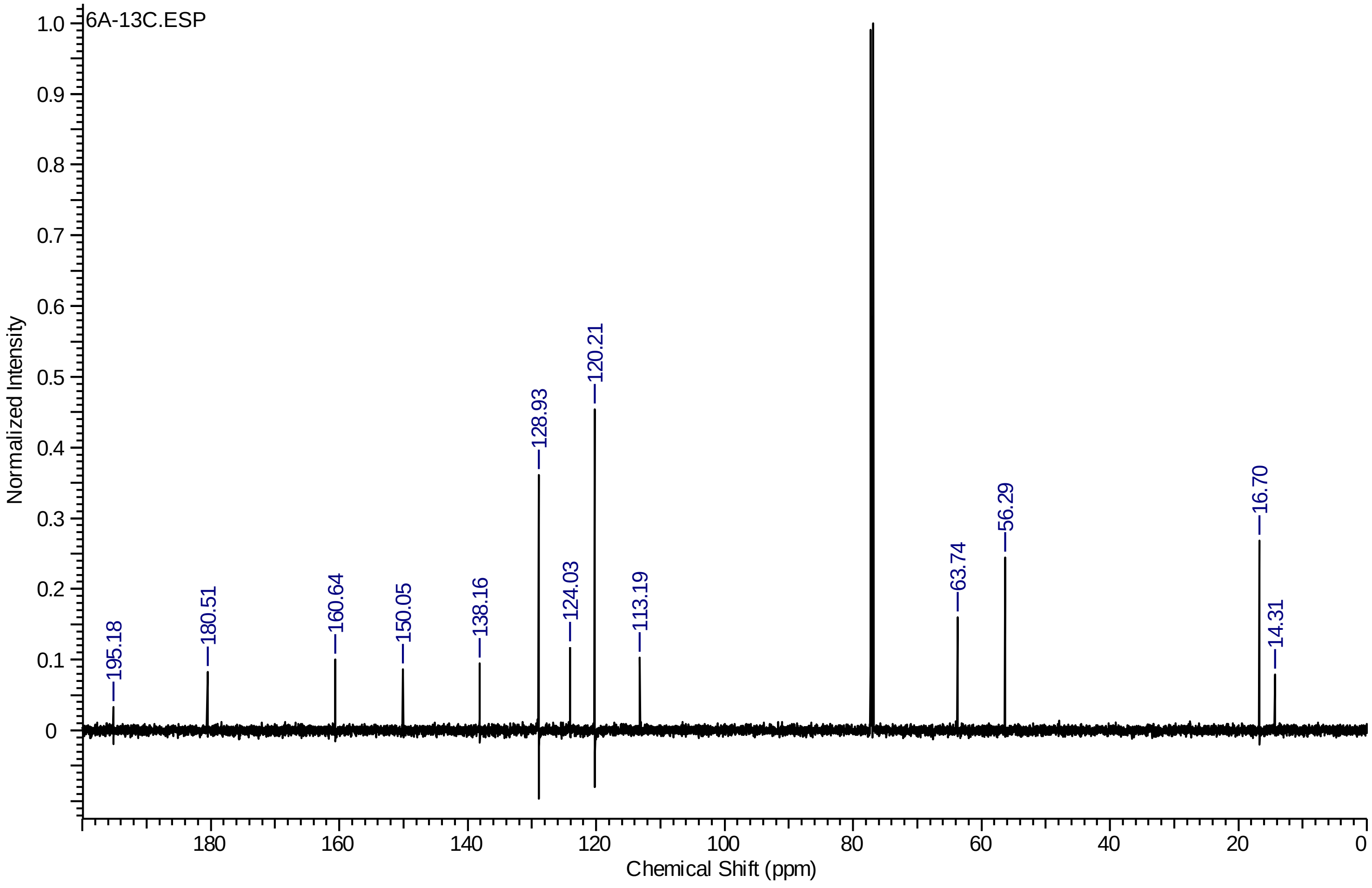
6A-13C.ESP
Normalized Intensity
Chemical Shift (ppm)
195.18
180.51
160.64
150.05
138.16
128.93
124.03
120.21
113.19
63.74
56.29
16.70
14.31

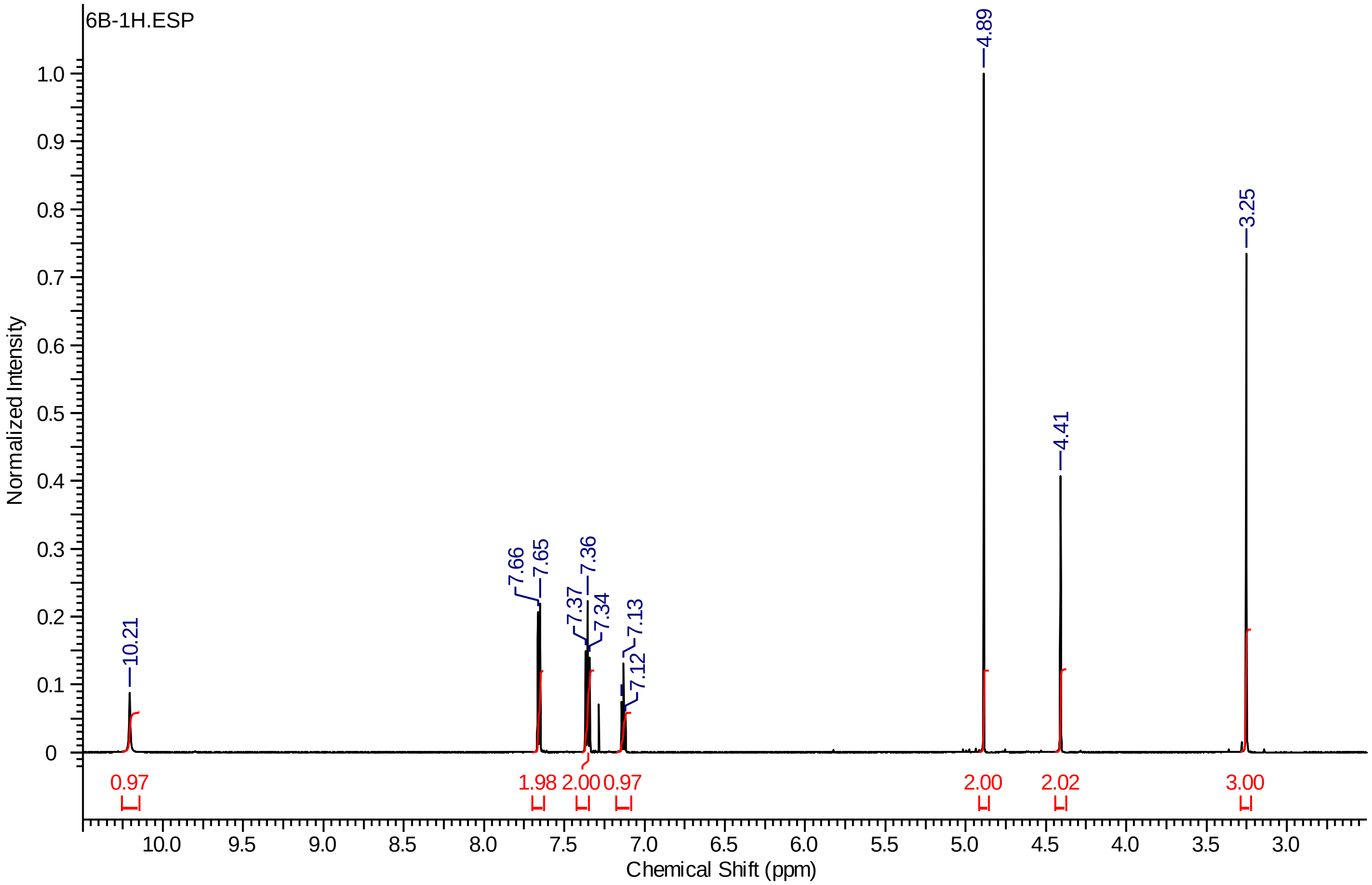
6B-1H.ESP
10.21
7.66
7.65
7.37
7.36
7.34
7.13
7.12
4.89
4.41
3.25
0.97
1.98
2.00
0.97
2.00
2.02
3.00
Normalized Intensity
Chemical Shift (ppm)

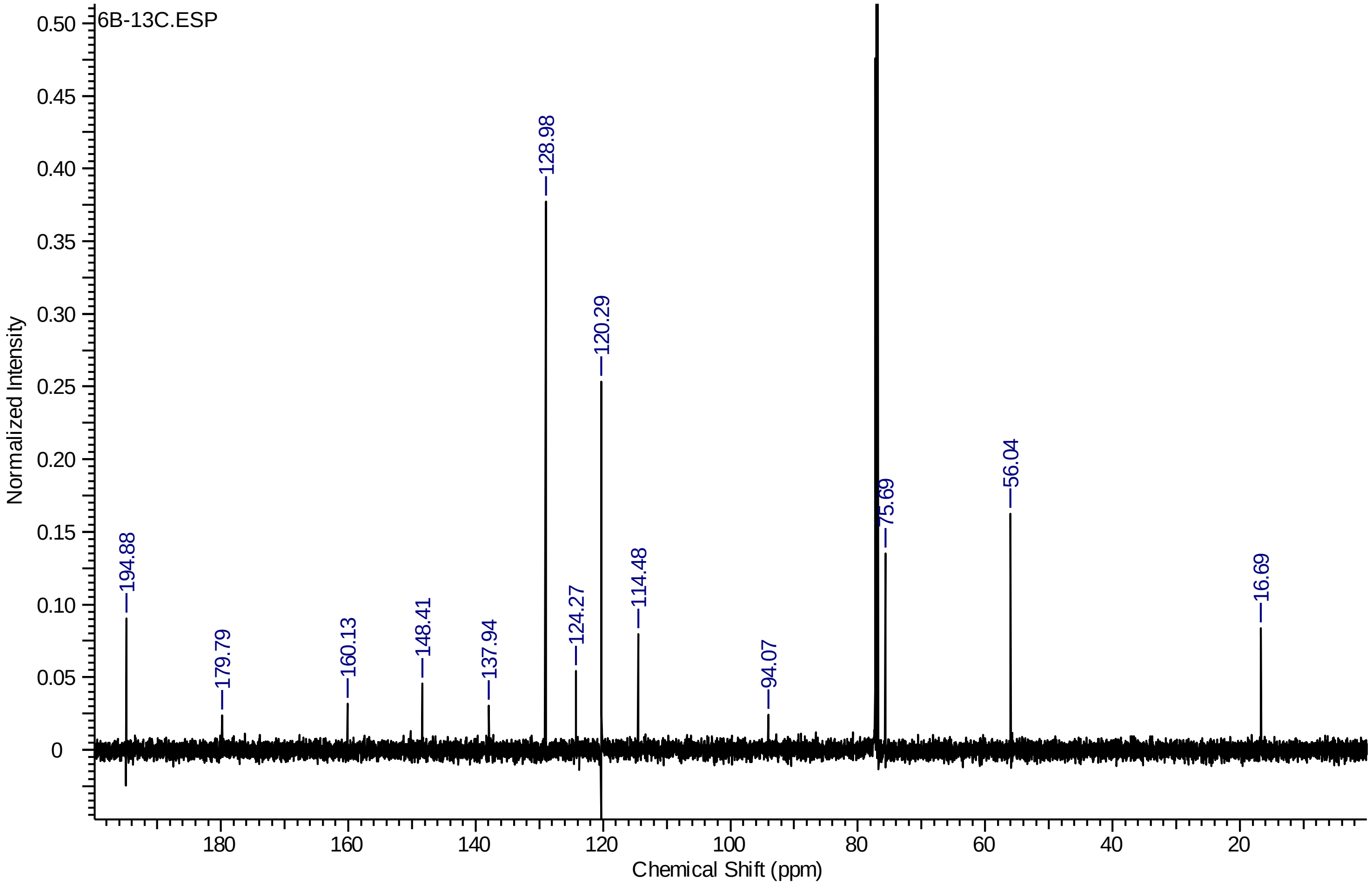
6B-13C.ESP
194.88
179.79
160.13
148.41
137.94
128.98
124.27
120.29
114.48
94.07
75.69
56.04
16.69
Normalized Intensity
Chemical Shift (ppm)

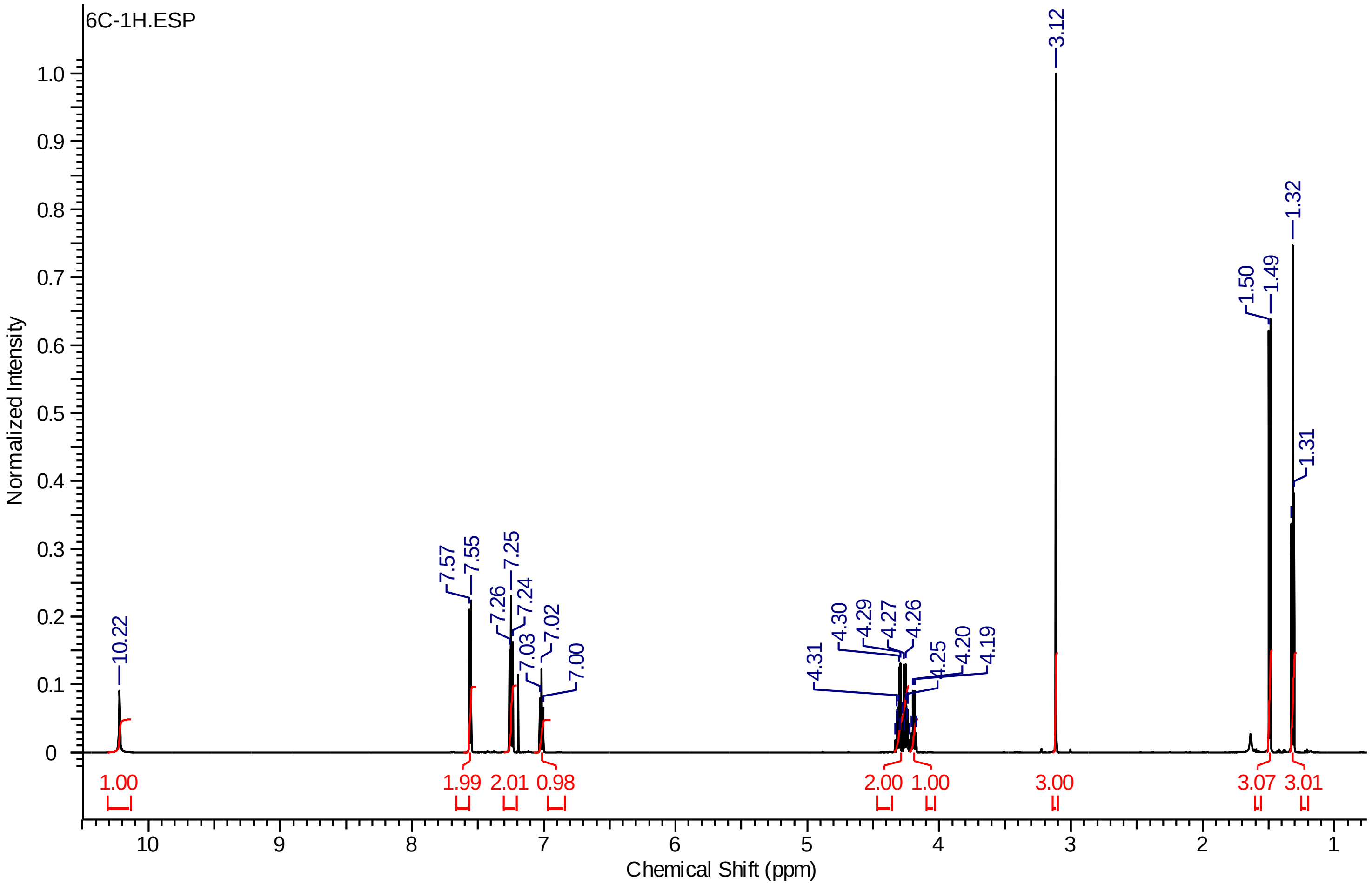
6C-1H.ESP
10.22
7.57
7.55
7.26
7.25
7.24
7.03
7.02
7.00
4.31
4.30
4.29
4.27
4.26
4.25
4.20
4.19
3.12
1.50
1.49
1.32
1.31
1.00
1.99
2.01
0.98
2.00
1.00
3.00
3.07
3.01
Normalized Intensity
Chemical Shift (ppm)

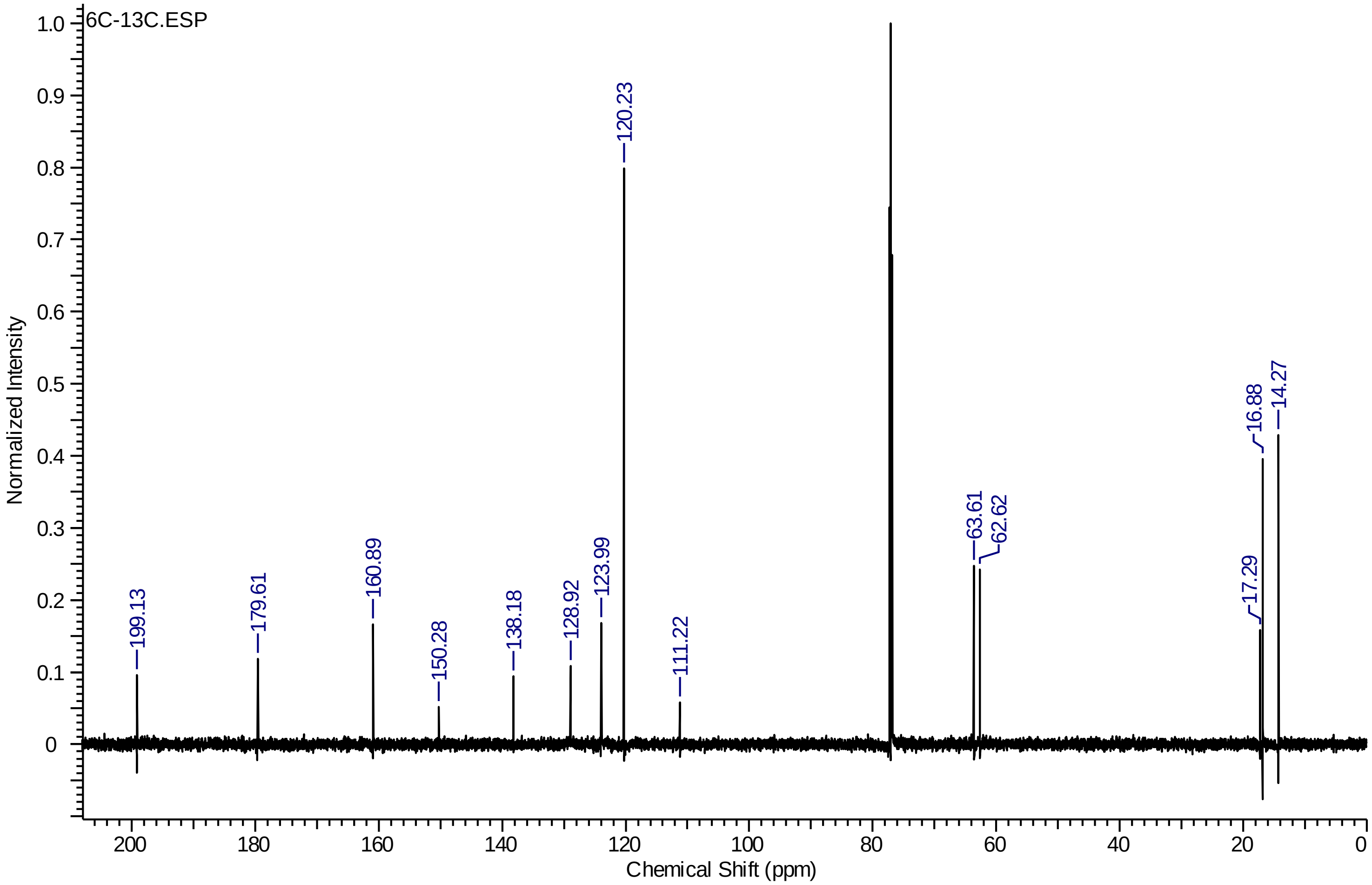
6C-13C.ESP
199.13
179.61
160.89
150.28
138.18
128.92
123.99
120.23
111.22
63.61
62.62
17.29
16.88
14.27
Normalized Intensity
Chemical Shift (ppm)

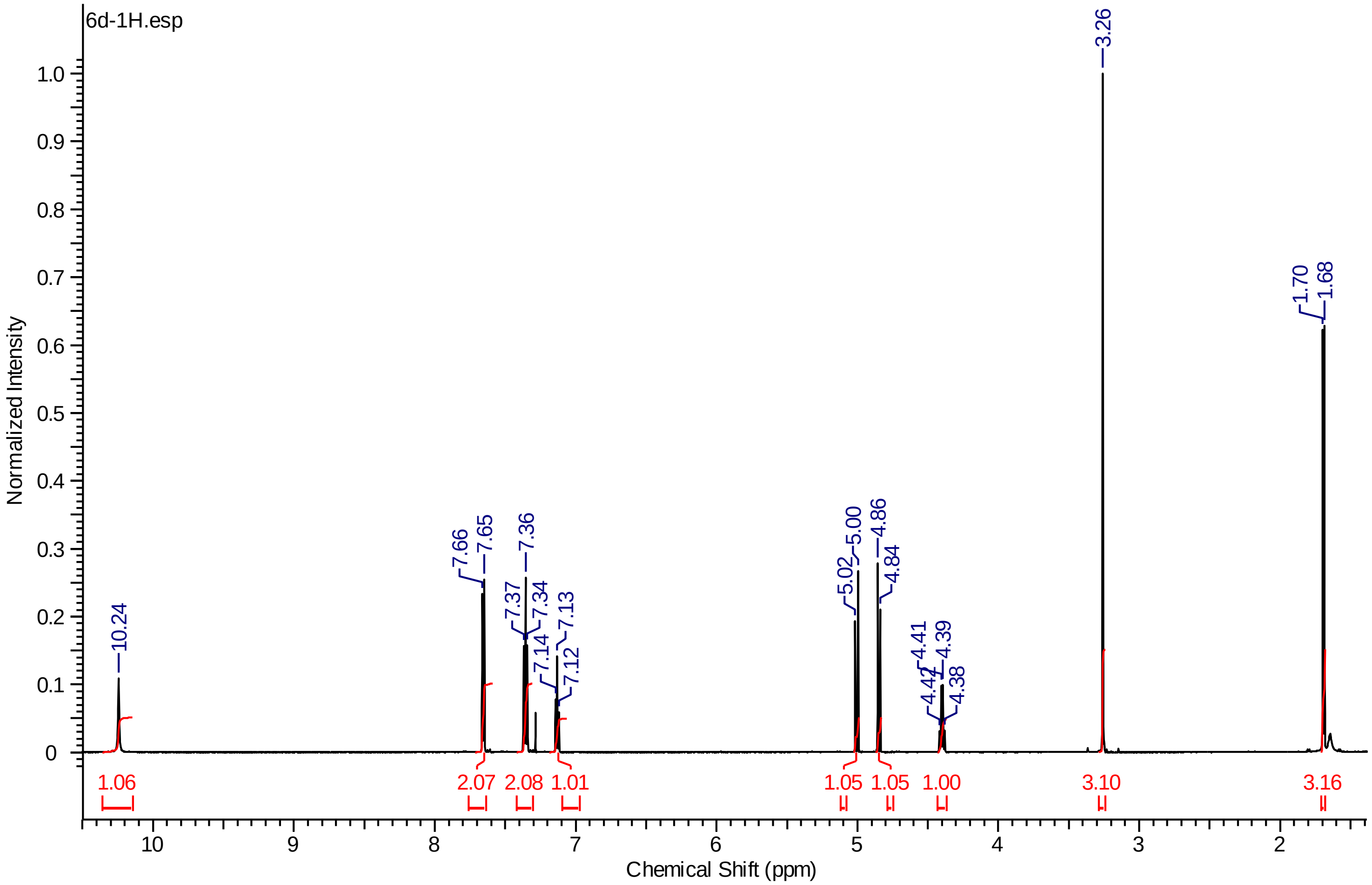
6d-1H.esp
10.24
7.66
7.65
7.37
7.36
7.34
7.14
7.13
7.12
5.02
5.00
4.86
4.84
4.42
4.41
4.39
4.38
3.26
1.70
1.68
1.06
2.07
2.08
1.01
1.05
1.05
1.00
3.10
3.16
Normalized Intensity
Chemical Shift (ppm)

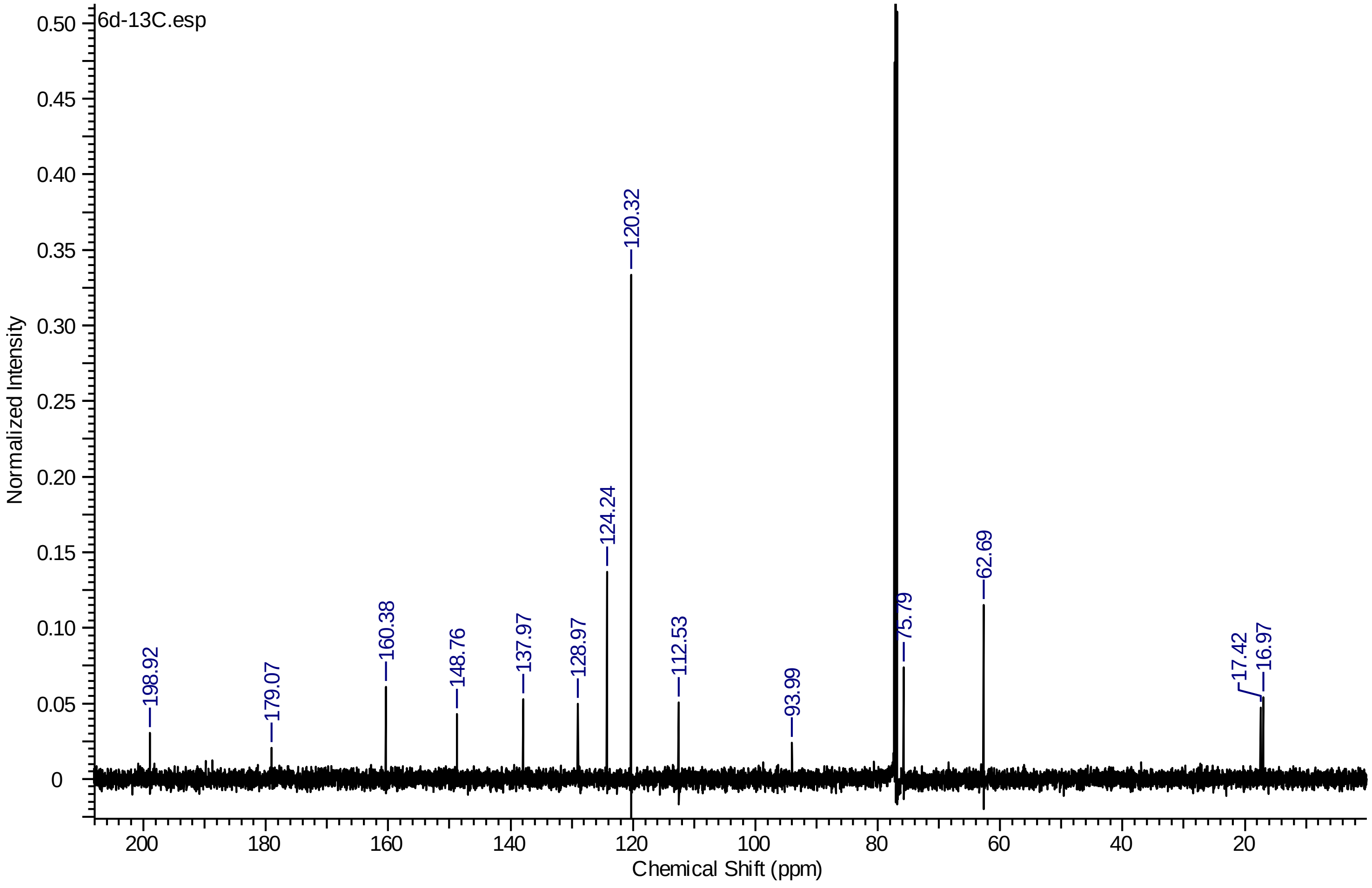
6d-13C.esp
198.92
179.07
160.38
148.76
137.97
128.97
124.24
120.32
112.53
93.99
75.79
62.69
17.42
16.97
Normalized Intensity
Chemical Shift (ppm)

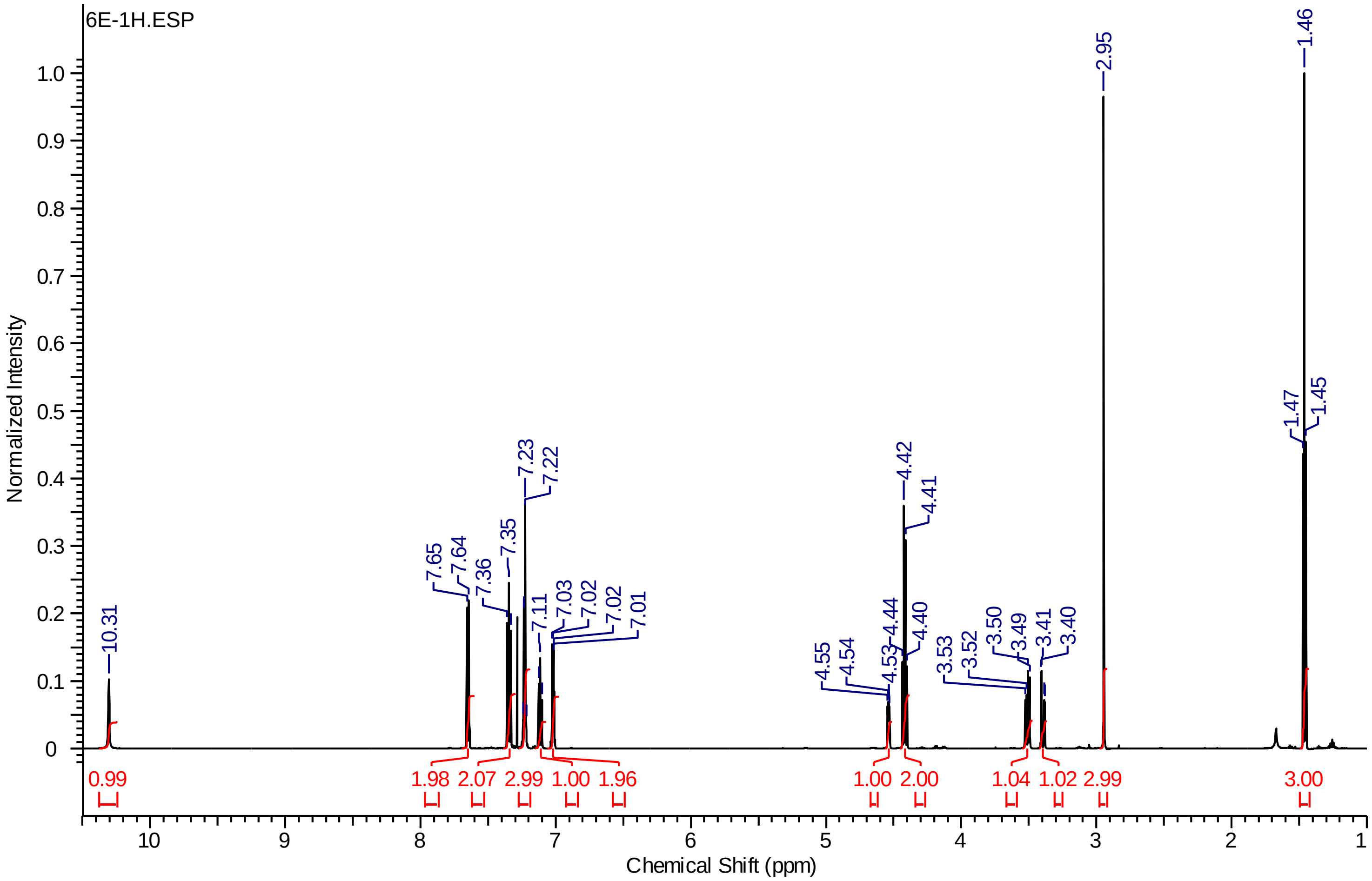
6E-1H.ESP
Normalized Intensity
Chemical Shift (ppm)
10.31
7.65
7.64
7.36
7.35
7.23
7.22
7.11
7.03
7.02
7.02
7.01
4.55
4.54
4.53
4.44
4.42
4.41
4.40
3.53
3.52
3.50
3.49
3.41
3.40
2.95
1.47
1.46
1.45
0.99
1.98
2.07
2.99
1.00
1.96
1.00
2.00
1.04
1.02
2.99
3.00

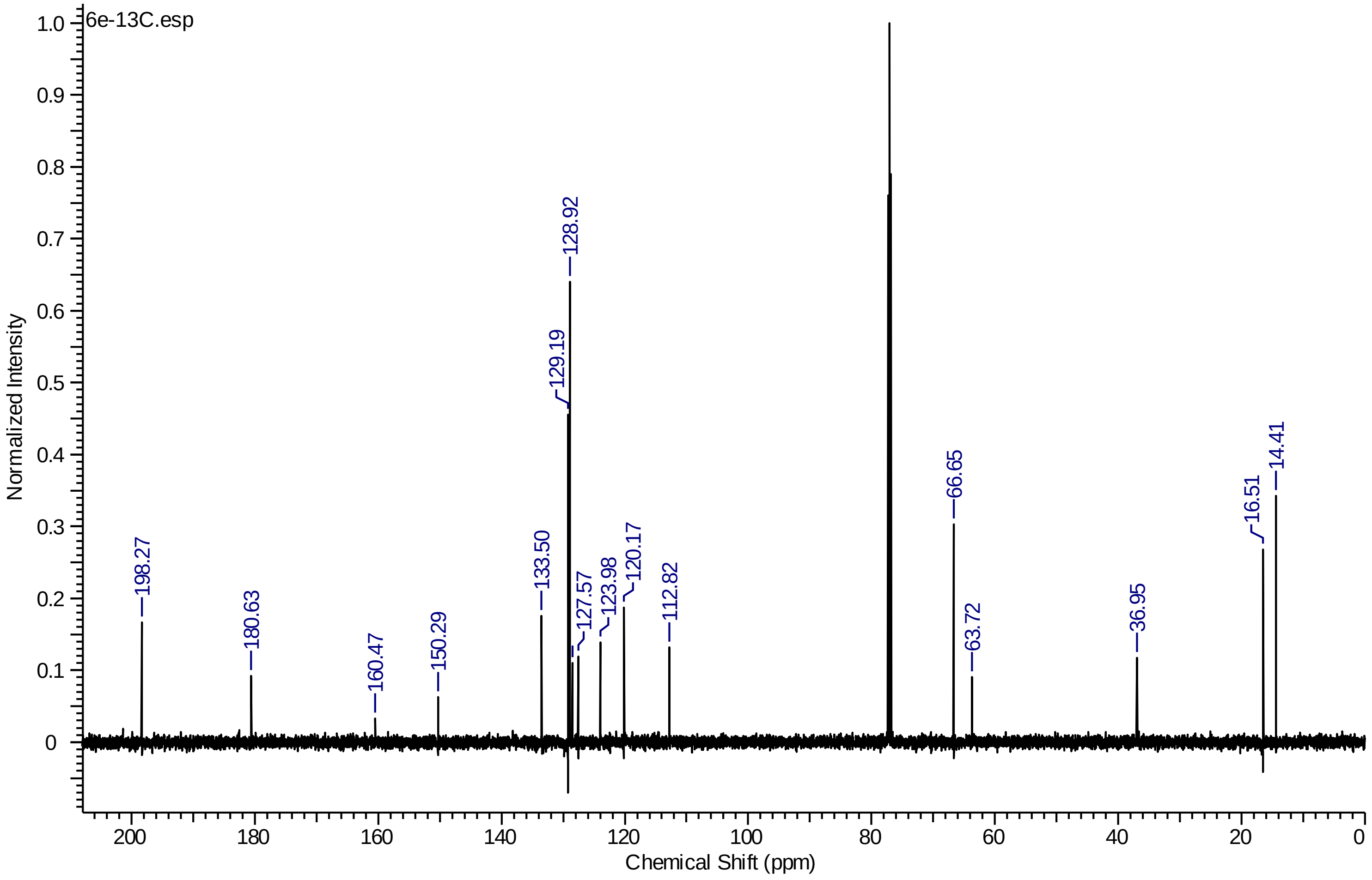
6e-13C.esp
Normalized Intensity
Chemical Shift (ppm)
198.27
180.63
160.47
150.29
133.50
129.19
128.92
127.57
123.98
120.17
112.82
66.65
63.72
36.95
16.51
14.41

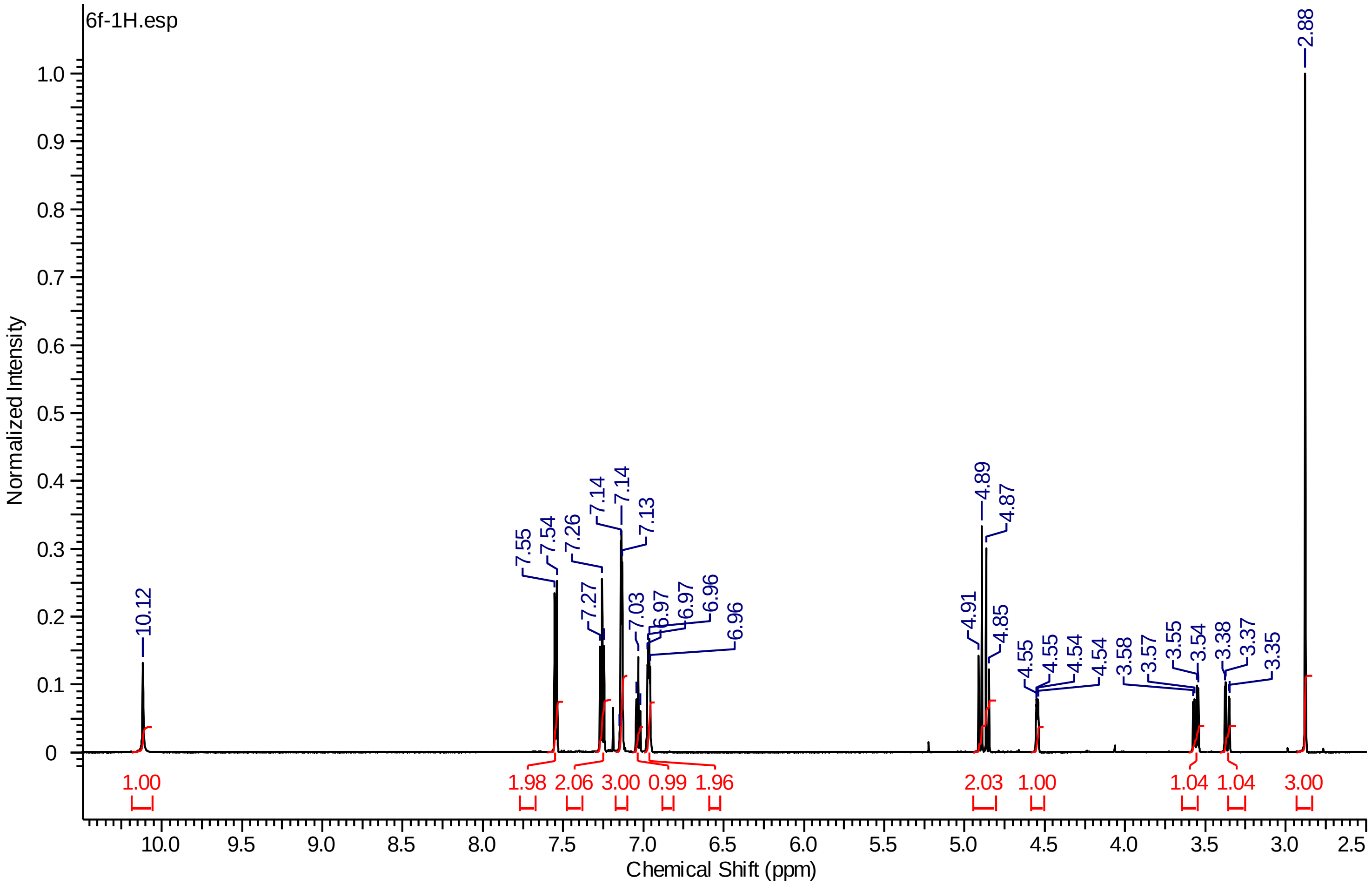
6f-1H.esp
Normalized Intensity
Chemical Shift (ppm)
10.12
7.55
7.54
7.26
7.27
7.14
7.14
7.13
7.03
6.97
6.97
6.96
6.96
4.91
4.89
4.87
4.85
4.55
4.55
4.54
4.54
3.58
3.57
3.55
3.54
3.38
3.37
3.35
2.88
1.00
1.98
2.06
3.00
0.99
1.96
2.03
1.00
1.04
1.04
3.00

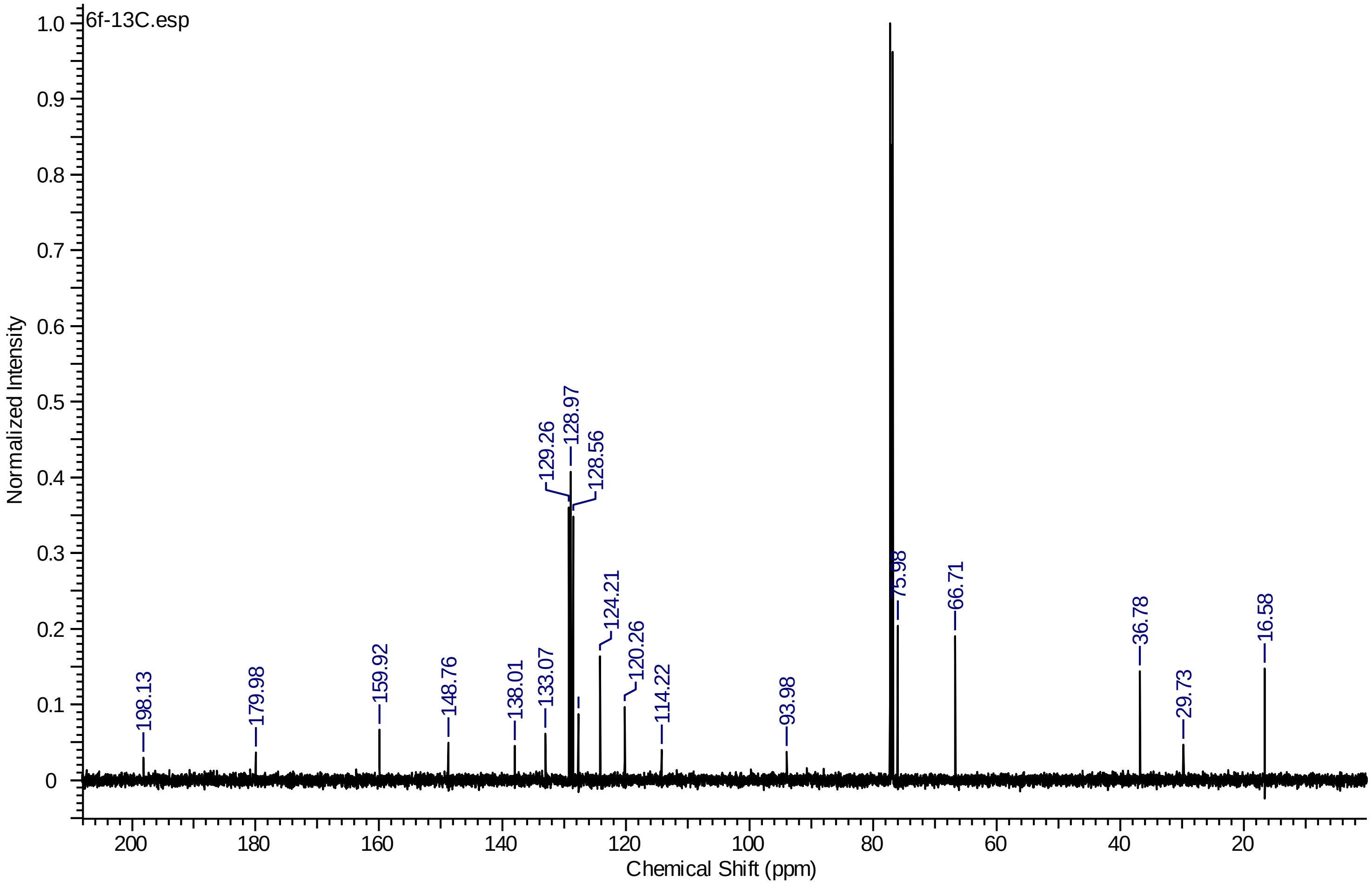
6f-13C.esp
Normalized Intensity
Chemical Shift (ppm)
198.13
179.98
159.92
148.76
138.01
133.07
129.26
128.97
128.56
124.21
120.26
114.22
93.98
75.98
66.71
36.78
29.73
16.58

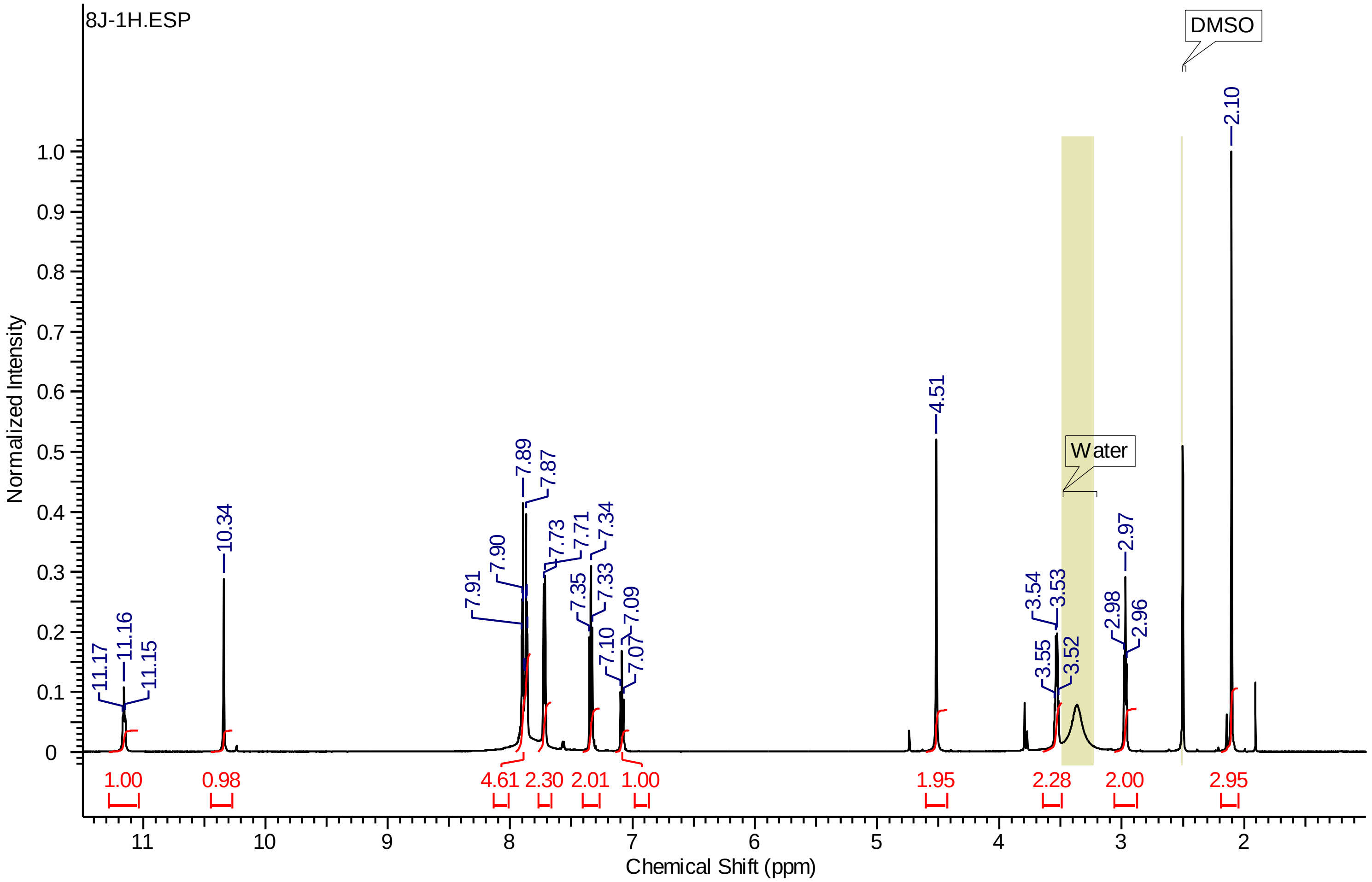
8J-1H.ESP
DMSO
Water
Normalized Intensity
Chemical Shift (ppm)
11.17
11.16
11.15
10.34
7.91
7.90
7.89
7.87
7.73
7.71
7.35
7.34
7.33
7.10
7.09
7.07
4.51
3.55
3.54
3.53
3.52
2.98
2.97
2.96
2.10
1.00
0.98
4.61
2.30
2.01
1.00
1.95
2.28
2.00
2.95

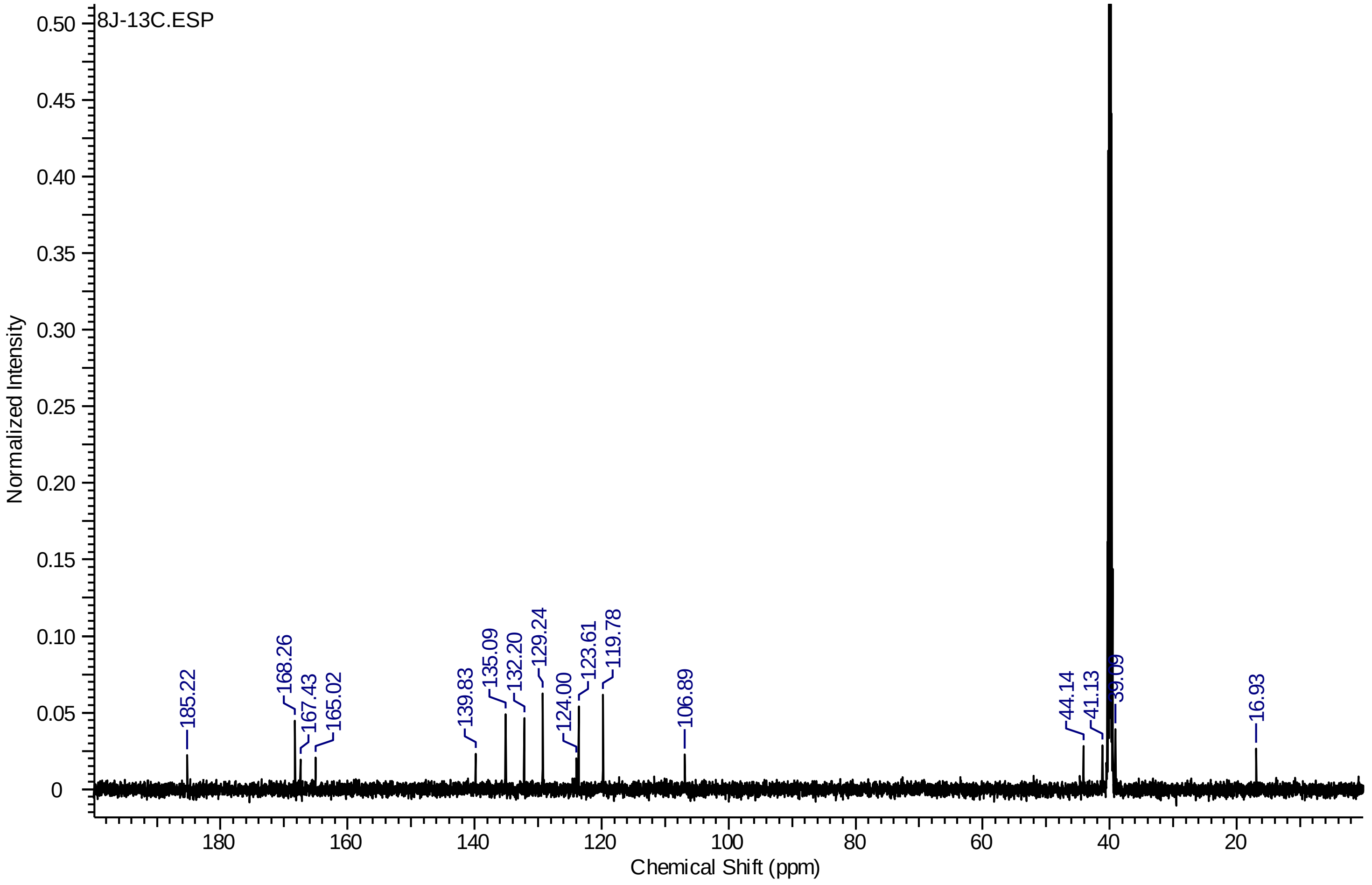
8J-13C.ESP
Normalized Intensity
Chemical Shift (ppm)
185.22
168.26
167.43
165.02
139.83
135.09
132.20
129.24
124.00
123.61
119.78
106.89
44.14
41.13
39.09
16.93

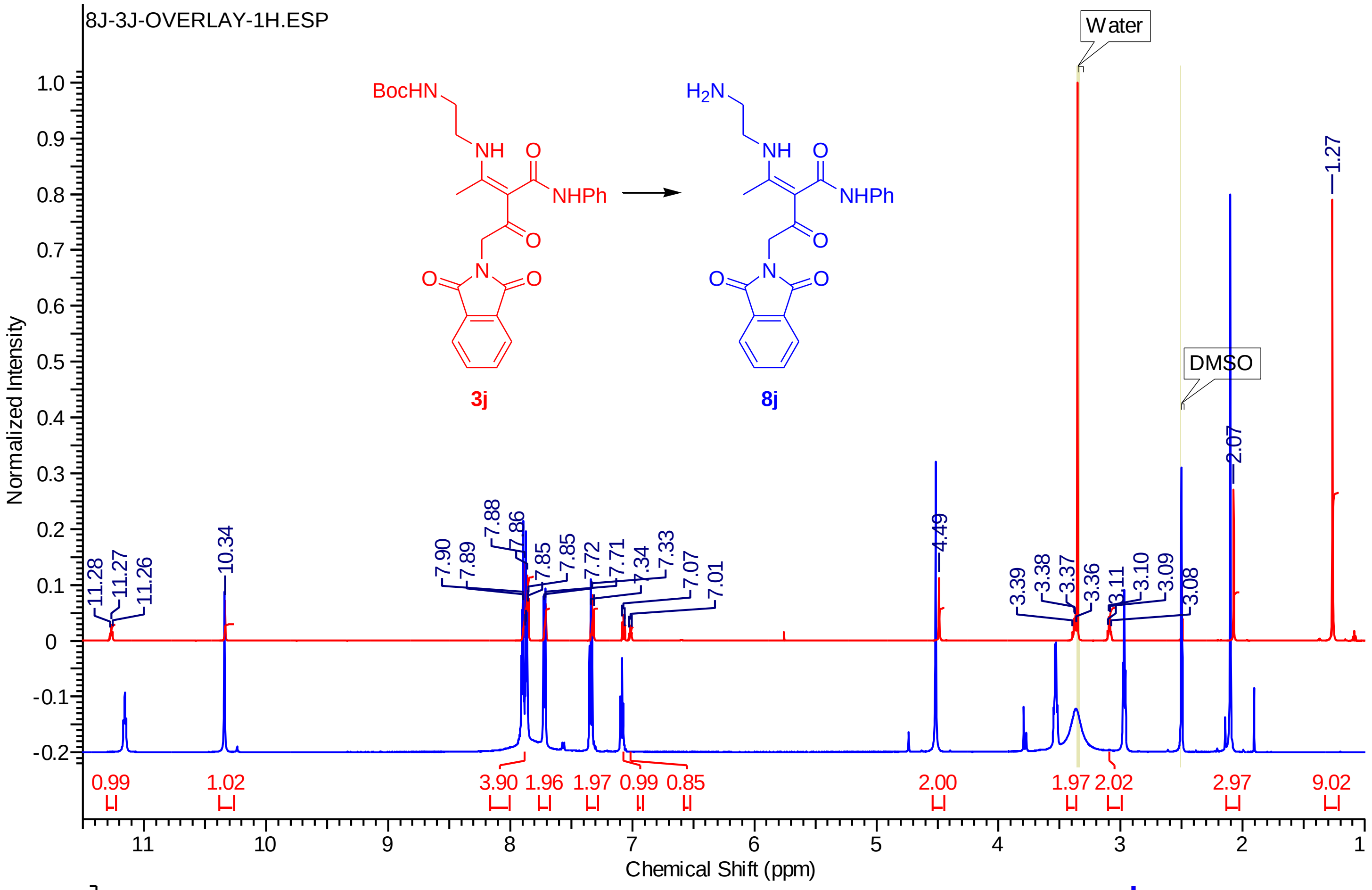
8J-3J-OVERLAY-1H.ESP
BocHN
NH
O
NHPh
O
O
N
O
3j
H2N
NH
O
NHPh
O
O
N
O
8j
Water
DMSO
11.28
11.27
11.26
10.34
7.90
7.89
7.88
7.86
7.85
7.85
7.72
7.71
7.34
7.33
7.07
7.01
4.49
3.39
3.38
3.37
3.36
3.11
3.10
3.09
3.08
2.07
1.27
0.99
1.02
3.90
1.96
1.97
0.99
0.85
2.00
1.97
2.02
2.97
9.02
Normalized Intensity
Chemical Shift (ppm)

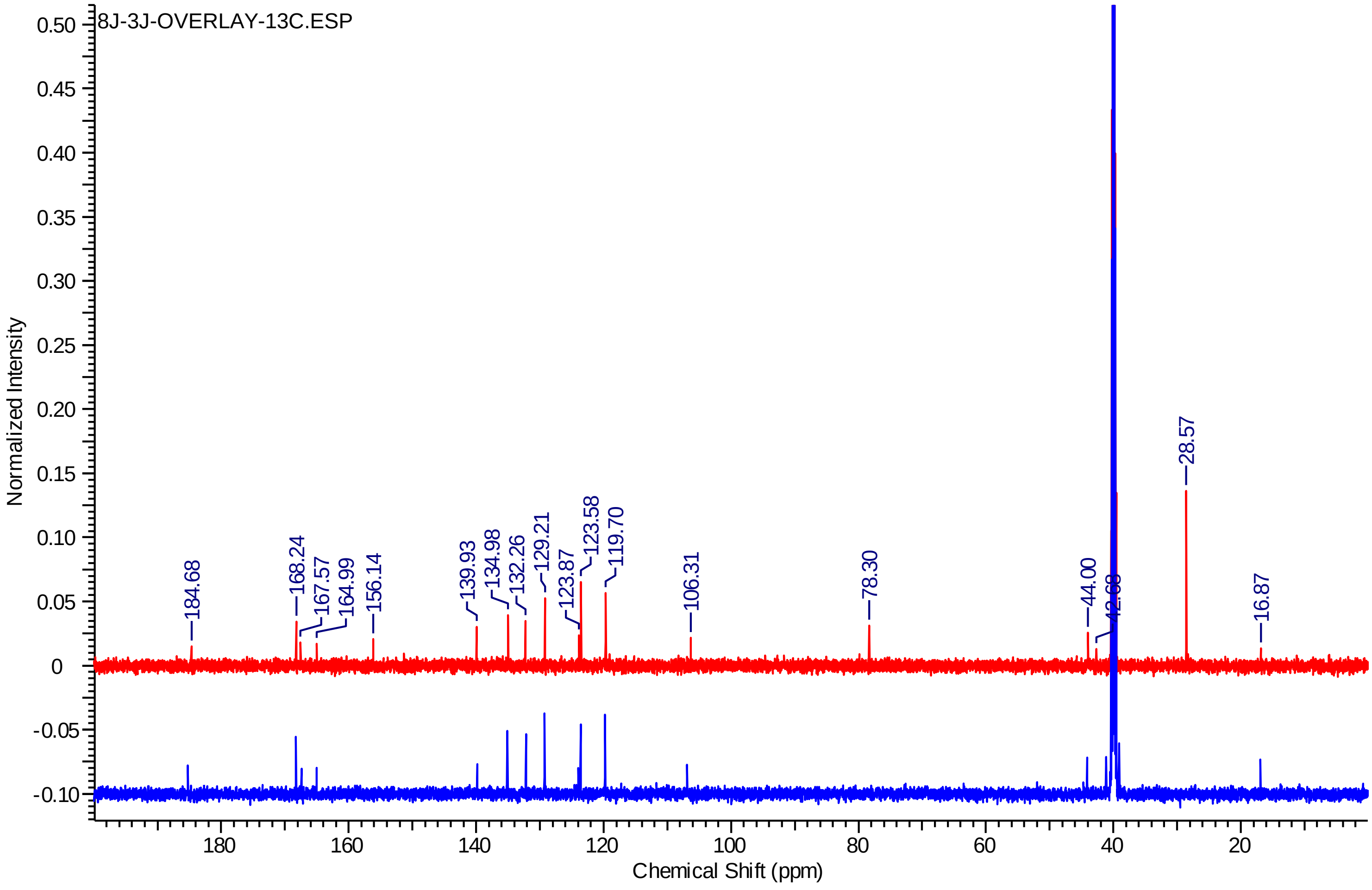
8J-3J-OVERLAY-13C.ESP
184.68
168.24
167.57
164.99
156.14
139.93
134.98
132.26
129.21
123.87
123.58
119.70
106.31
78.30
44.00
42.68
28.57
16.87
Normalized Intensity
Chemical Shift (ppm)

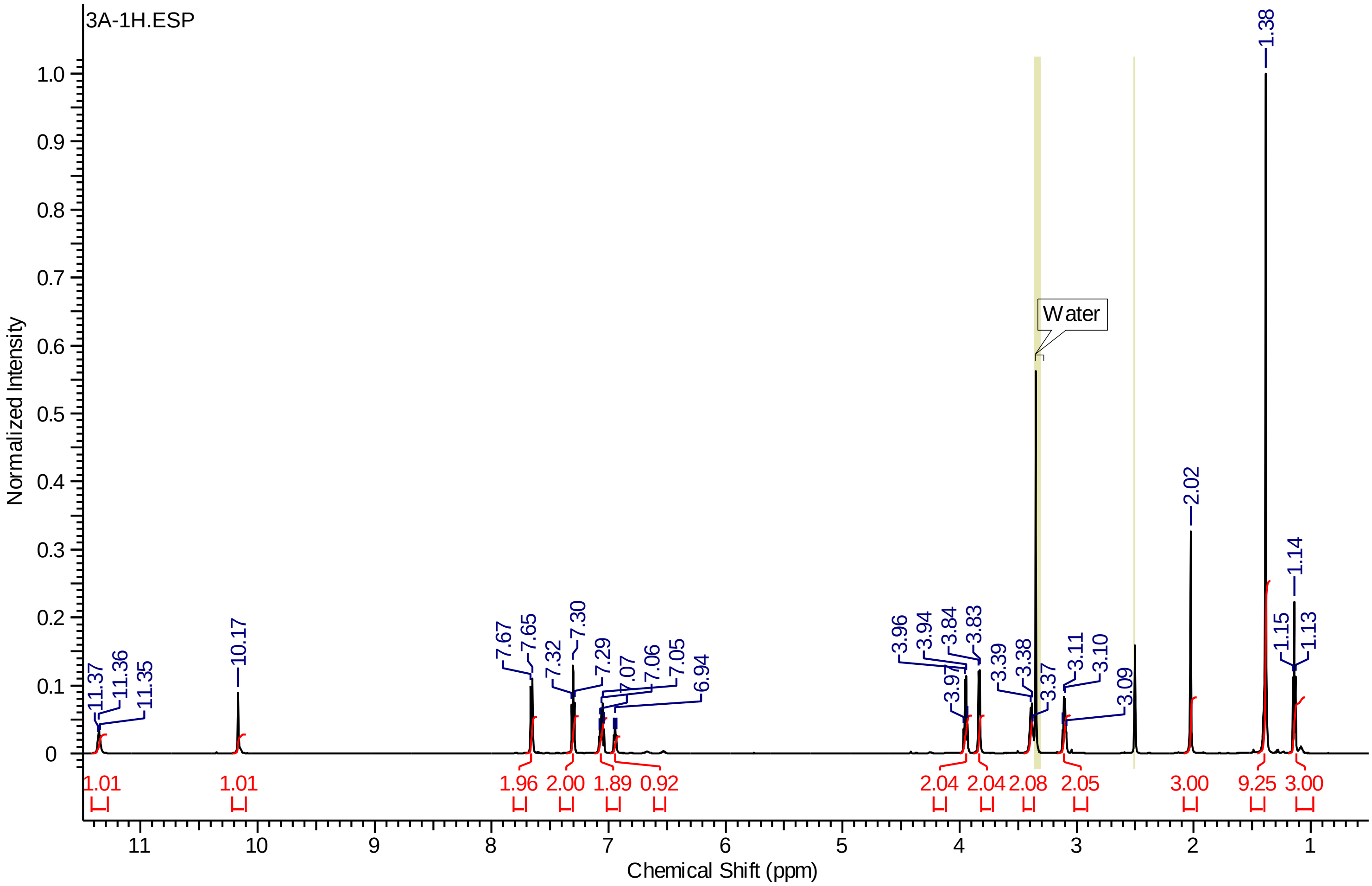
3A-1H.ESP
Water
Normalized Intensity
Chemical Shift (ppm)
11.37
11.36
11.35
10.17
7.67
7.65
7.32
7.30
7.29
7.07
7.06
7.05
6.94
3.96
3.94
3.84
3.83
3.97
3.39
3.38
3.37
3.11
3.10
3.09
2.02
1.38
1.15
1.14
1.13
1.01
1.01
1.96
2.00
1.89
0.92
2.04
2.04
2.08
2.05
3.00
9.25
3.00

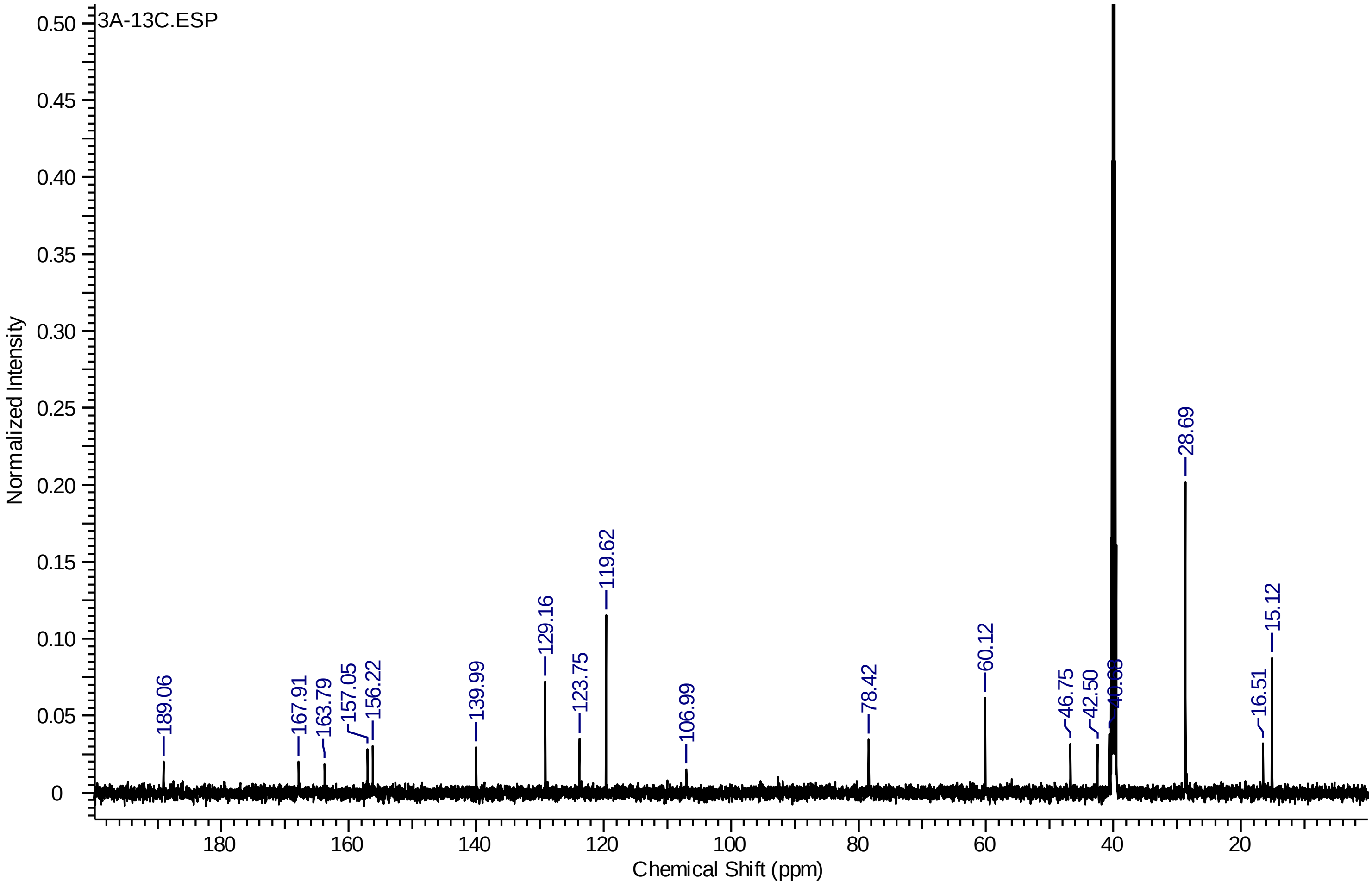
3A-13C.ESP
Normalized Intensity
Chemical Shift (ppm)
189.06
167.91
163.79
157.05
156.22
139.99
129.16
123.75
119.62
106.99
78.42
60.12
46.75
42.50
28.69
16.51
15.12

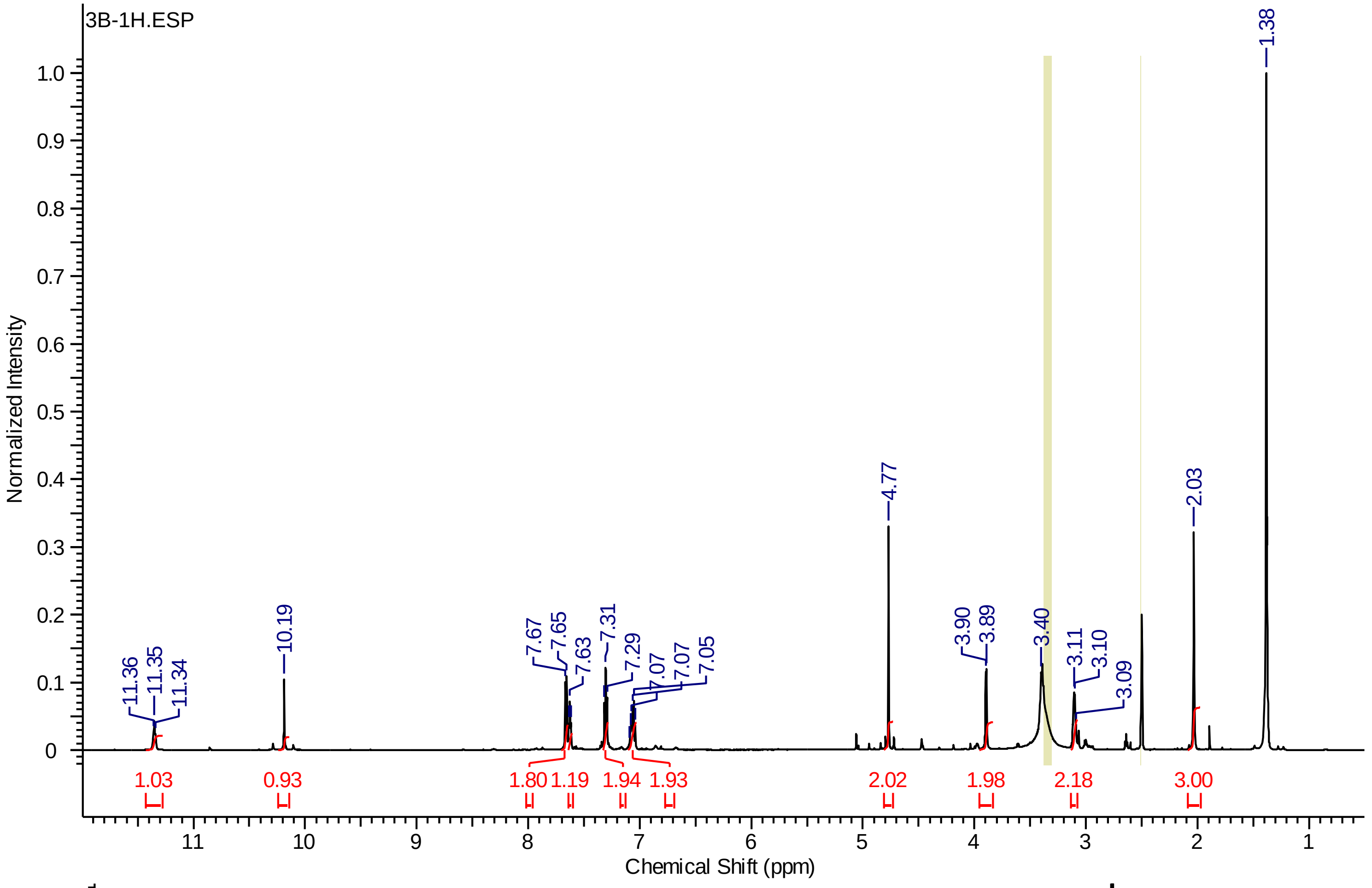
3B-1H.ESP
Normalized Intensity
Chemical Shift (ppm)
11.36
11.35
11.34
10.19
7.67
7.65
7.63
7.31
7.29
7.07
7.07
7.05
4.77
3.90
3.89
3.40
3.11
3.10
3.09
2.03
1.38
1.03
0.93
1.80
1.19
1.94
1.93
2.02
1.98
2.18
3.00

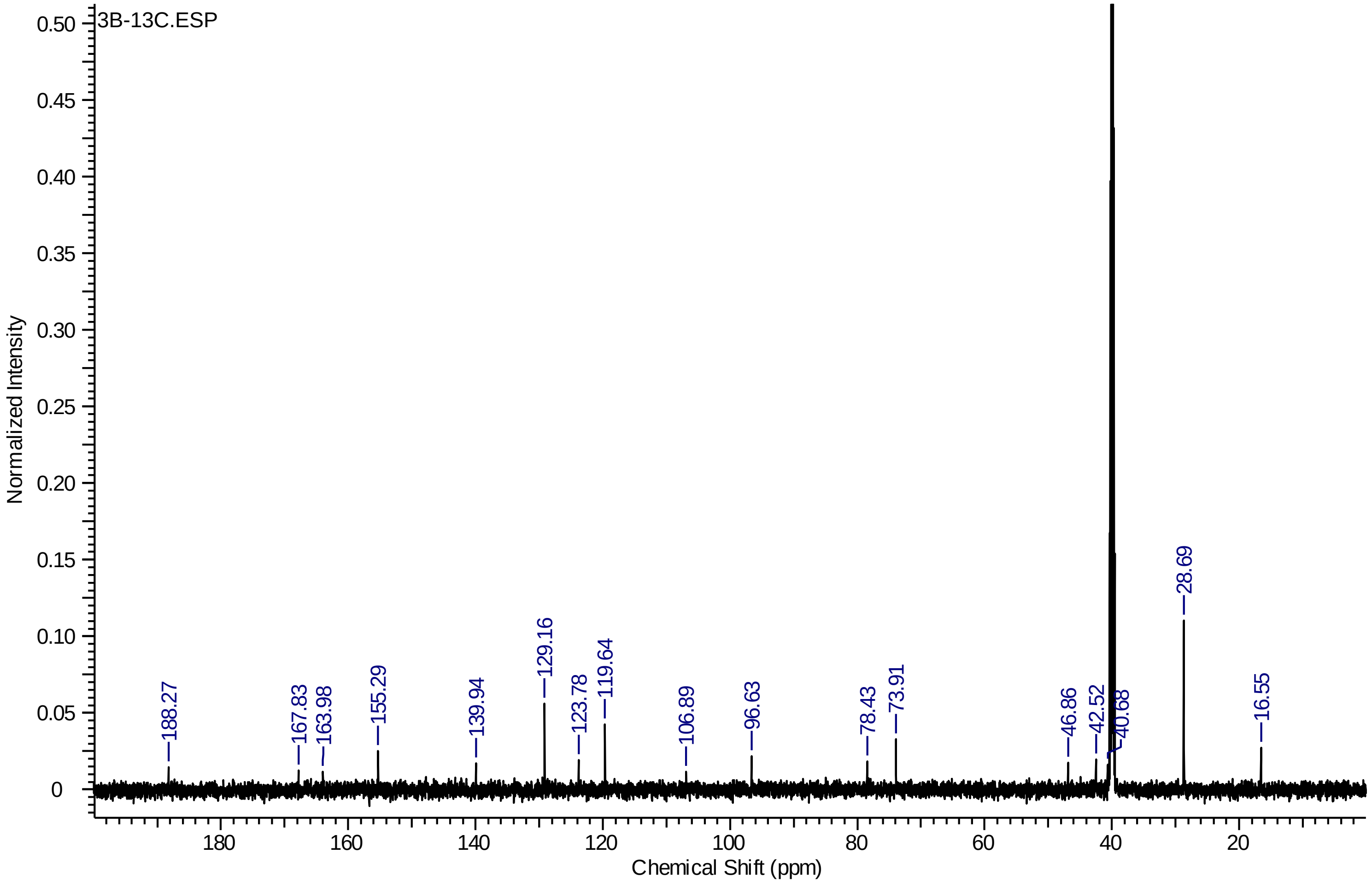
3B-13C.ESP
Normalized Intensity
Chemical Shift (ppm)
188.27
167.83
163.98
155.29
139.94
129.16
123.78
119.64
106.89
96.63
78.43
73.91
46.86
42.52
40.68
28.69
16.55

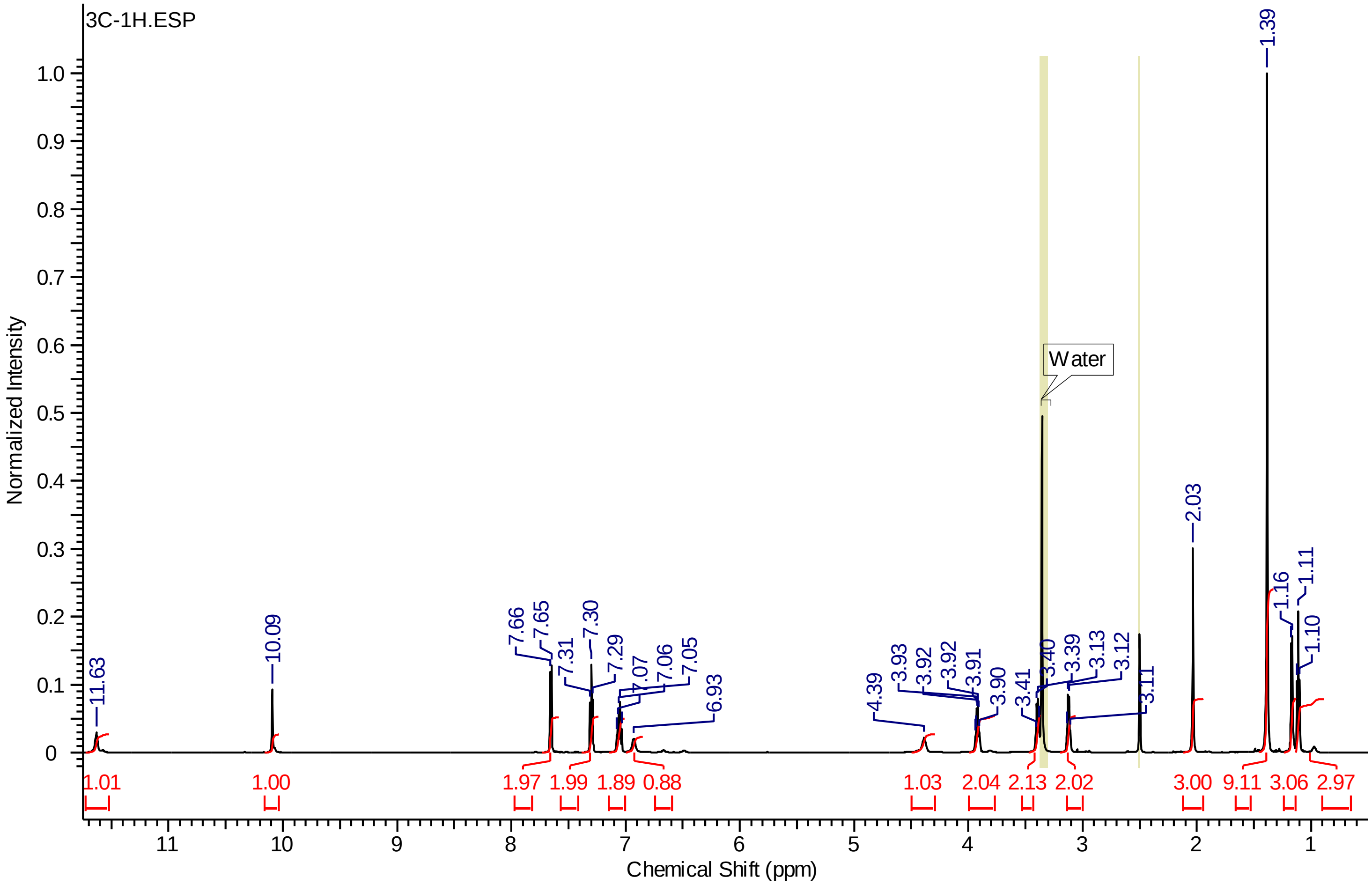
3C-1H.ESP
Normalized Intensity
Chemical Shift (ppm)
Water
11.63
10.09
7.66
7.65
7.31
7.30
7.29
7.07
7.06
7.05
6.93
4.39
3.93
3.92
3.92
3.91
3.90
3.41
3.40
3.39
3.13
3.12
3.11
2.03
1.39
1.16
1.11
1.10
1.01
1.00
1.97
1.99
1.89
0.88
1.03
2.04
2.13
2.02
3.00
9.11
3.06
2.97

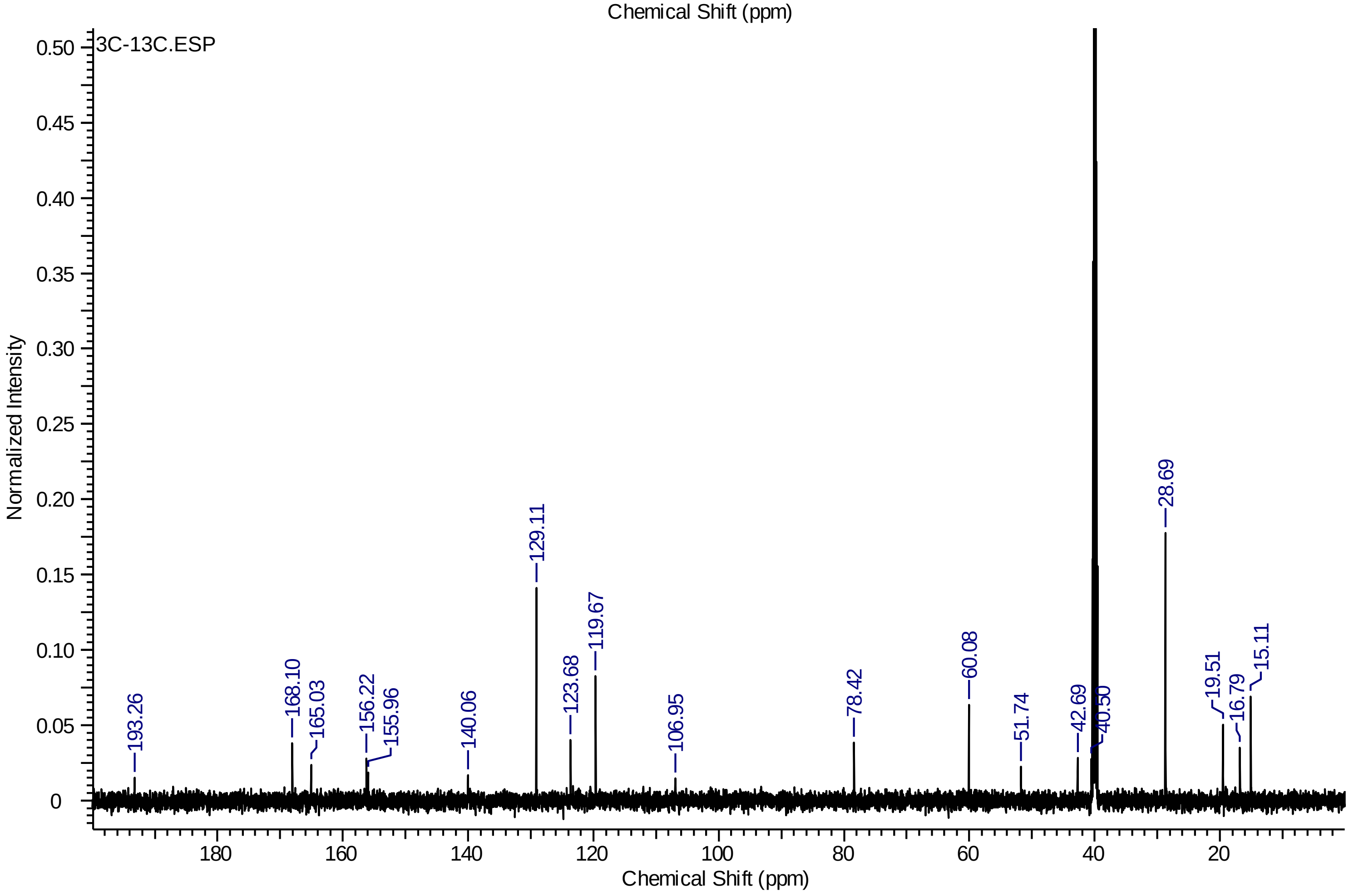
3C-13C.ESP
Normalized Intensity
Chemical Shift (ppm)
193.26
168.10
165.03
156.22
155.96
140.06
129.11
123.68
119.67
106.95
78.42
60.08
51.74
42.69
40.50
28.69
19.51
16.79
15.11

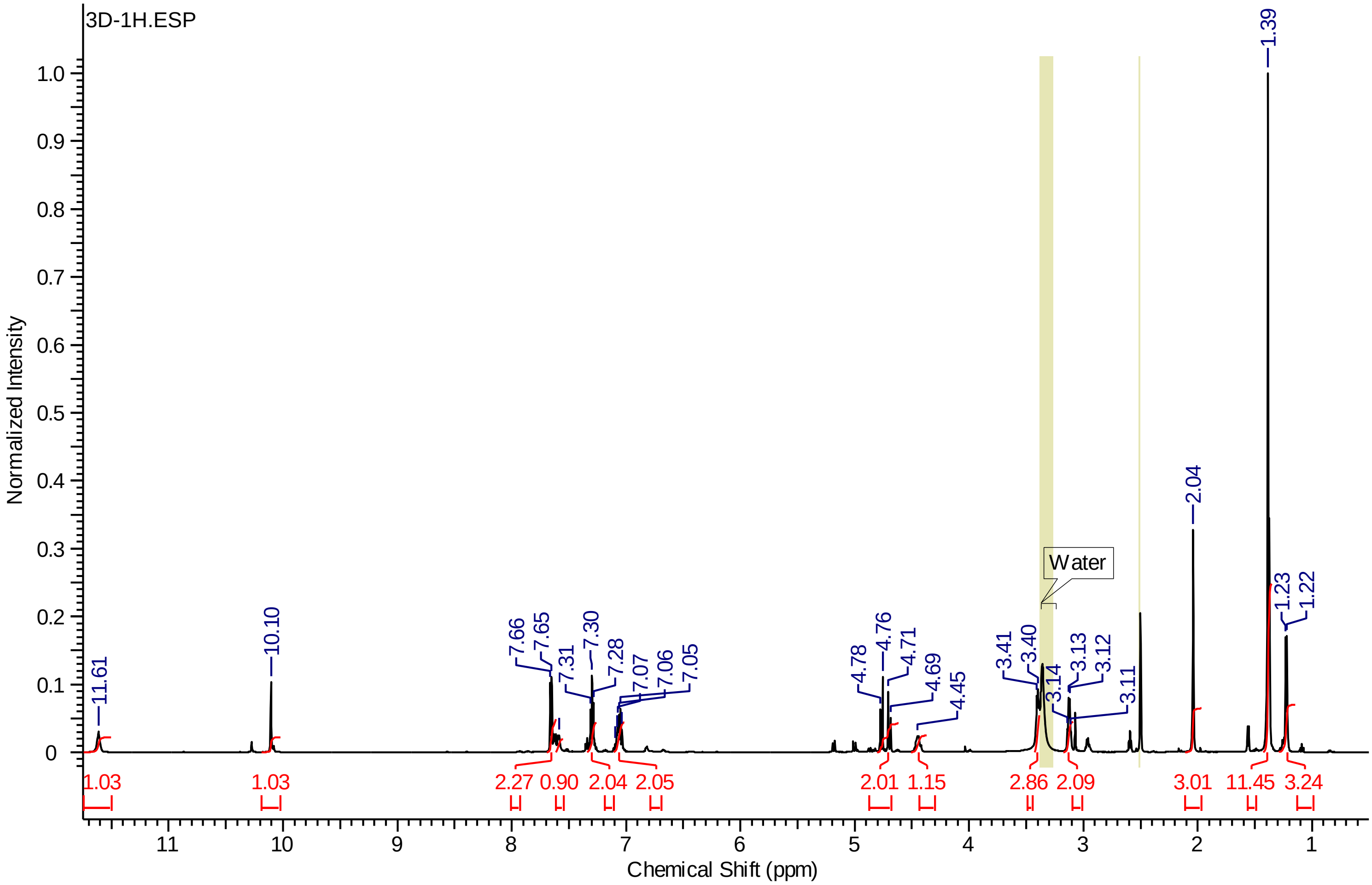
3D-1H.ESP
Normalized Intensity
Chemical Shift (ppm)
Water
11.61
10.10
7.66
7.65
7.31
7.30
7.28
7.07
7.06
7.05
4.78
4.76
4.71
4.69
4.45
3.41
3.40
3.14
3.13
3.12
3.11
2.04
1.39
1.23
1.22
1.03
1.03
2.27
0.90
2.04
2.05
2.01
1.15
2.86
2.09
3.01
11.45
3.24

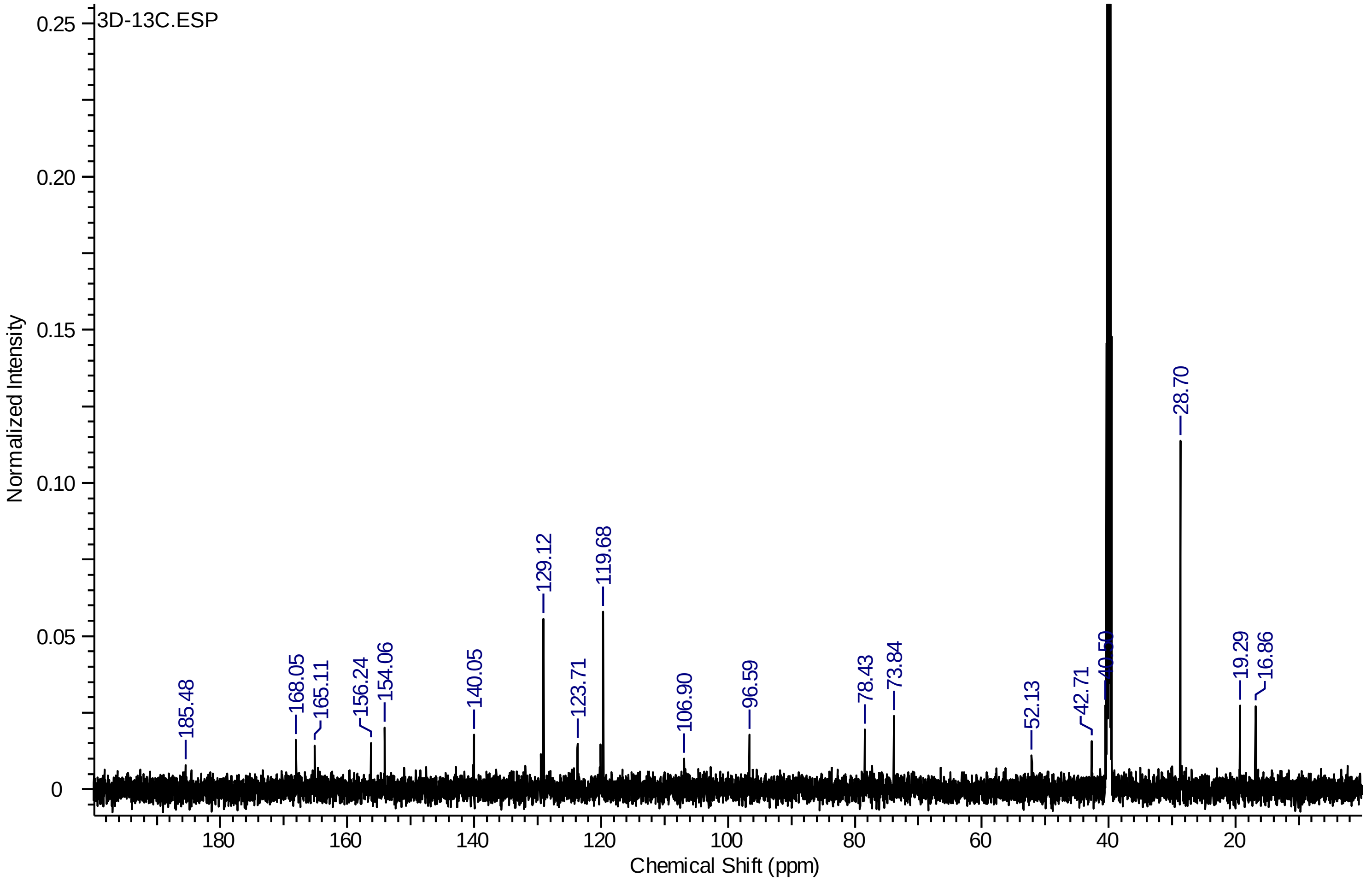
3D-13C.ESP
Normalized Intensity
Chemical Shift (ppm)
185.48
168.05
165.11
156.24
154.06
140.05
129.12
123.71
119.68
106.90
96.59
78.43
73.84
52.13
42.71
40.50
28.70
19.29
16.86

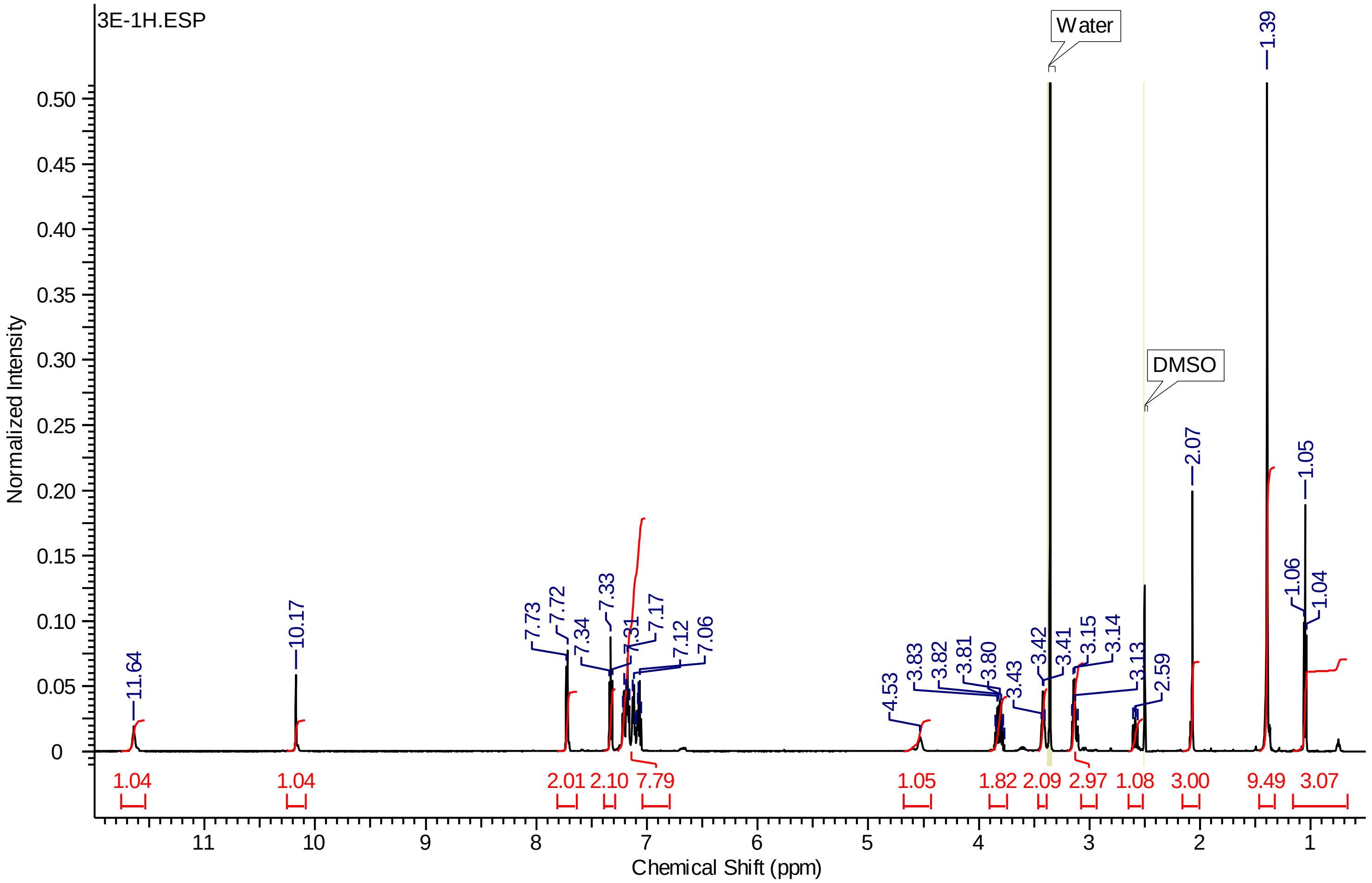
3E-1H.ESP
Water
DMSO
11.64
10.17
7.73
7.72
7.34
7.33
7.31
7.17
7.12
7.06
4.53
3.83
3.82
3.81
3.80
3.43
3.42
3.41
3.15
3.14
3.13
2.59
2.07
1.39
1.06
1.05
1.04
1.04
1.04
2.01
2.10
7.79
1.05
1.82
2.09
2.97
1.08
3.00
9.49
3.07
Normalized Intensity
Chemical Shift (ppm)

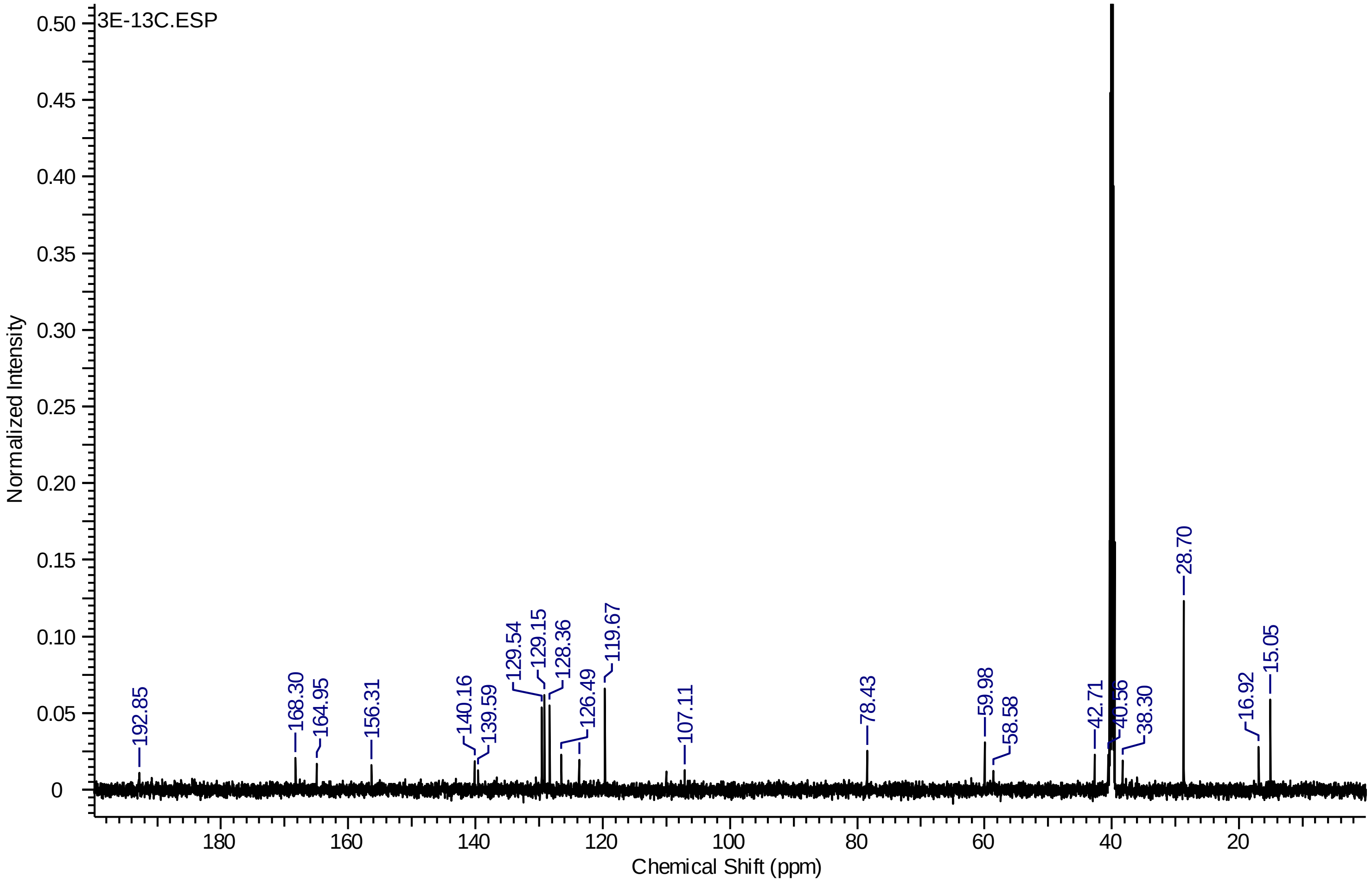
3E-13C.ESP
192.85
168.30
164.95
156.31
140.16
139.59
129.54
129.15
128.36
126.49
119.67
107.11
78.43
59.98
58.58
42.71
40.56
38.30
28.70
16.92
15.05
Normalized Intensity
Chemical Shift (ppm)

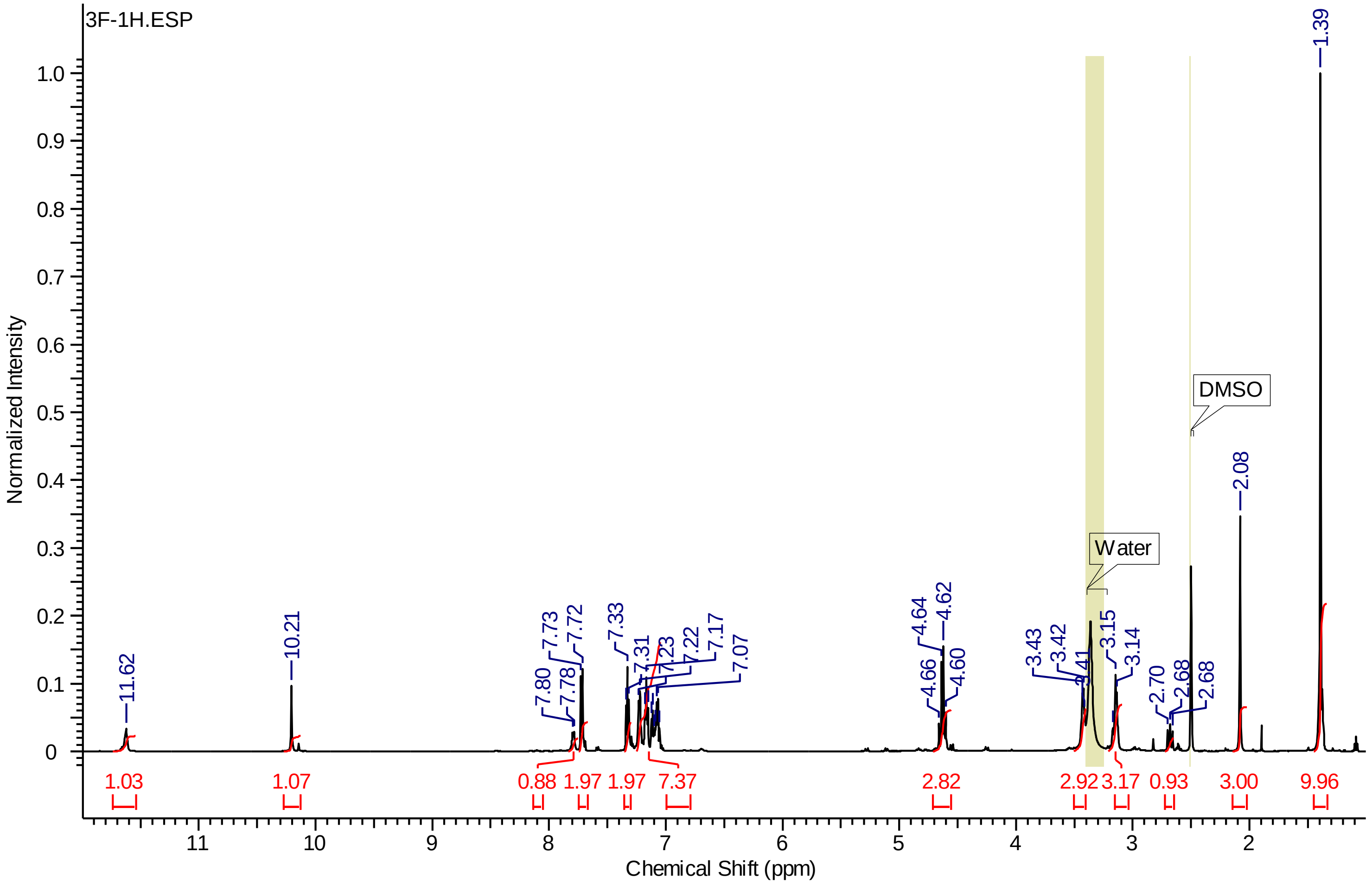
3F-1H.ESP
Normalized Intensity
Chemical Shift (ppm)
Water
DMSO
11.62
10.21
7.80
7.78
7.73
7.72
7.33
7.31
7.23
7.22
7.17
7.07
4.66
4.64
4.62
4.60
3.43
3.42
3.41
3.15
3.14
2.70
2.68
2.68
2.08
1.39
1.03
1.07
0.88
1.97
1.97
7.37
2.82
2.92
3.17
0.93
3.00
9.96

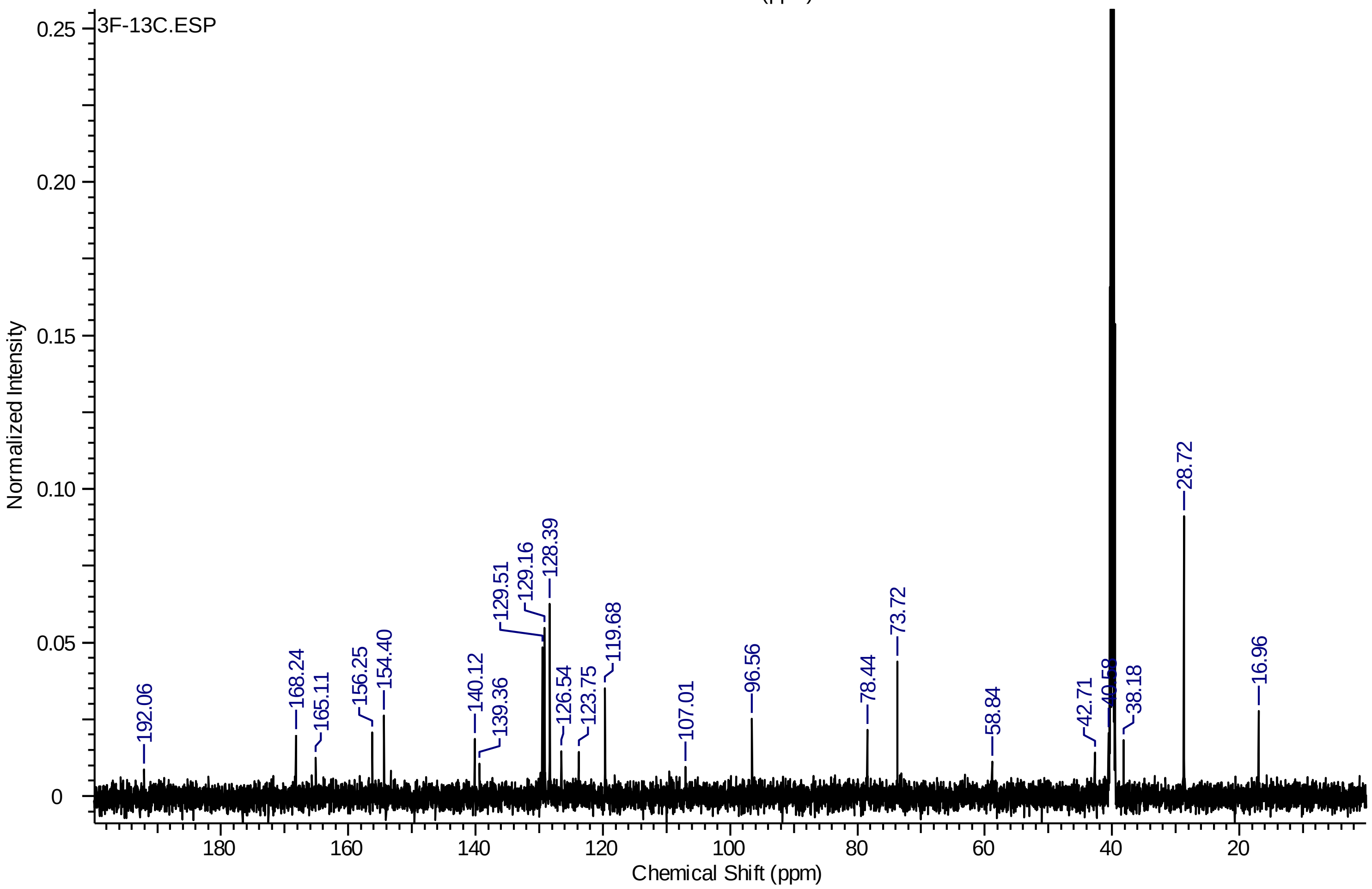
3F-13C.ESP
Normalized Intensity
Chemical Shift (ppm)
192.06
168.24
165.11
156.25
154.40
140.12
139.36
129.51
129.16
128.39
126.54
123.75
119.68
107.01
96.56
78.44
73.72
58.84
42.71
40.58
38.18
28.72
16.96

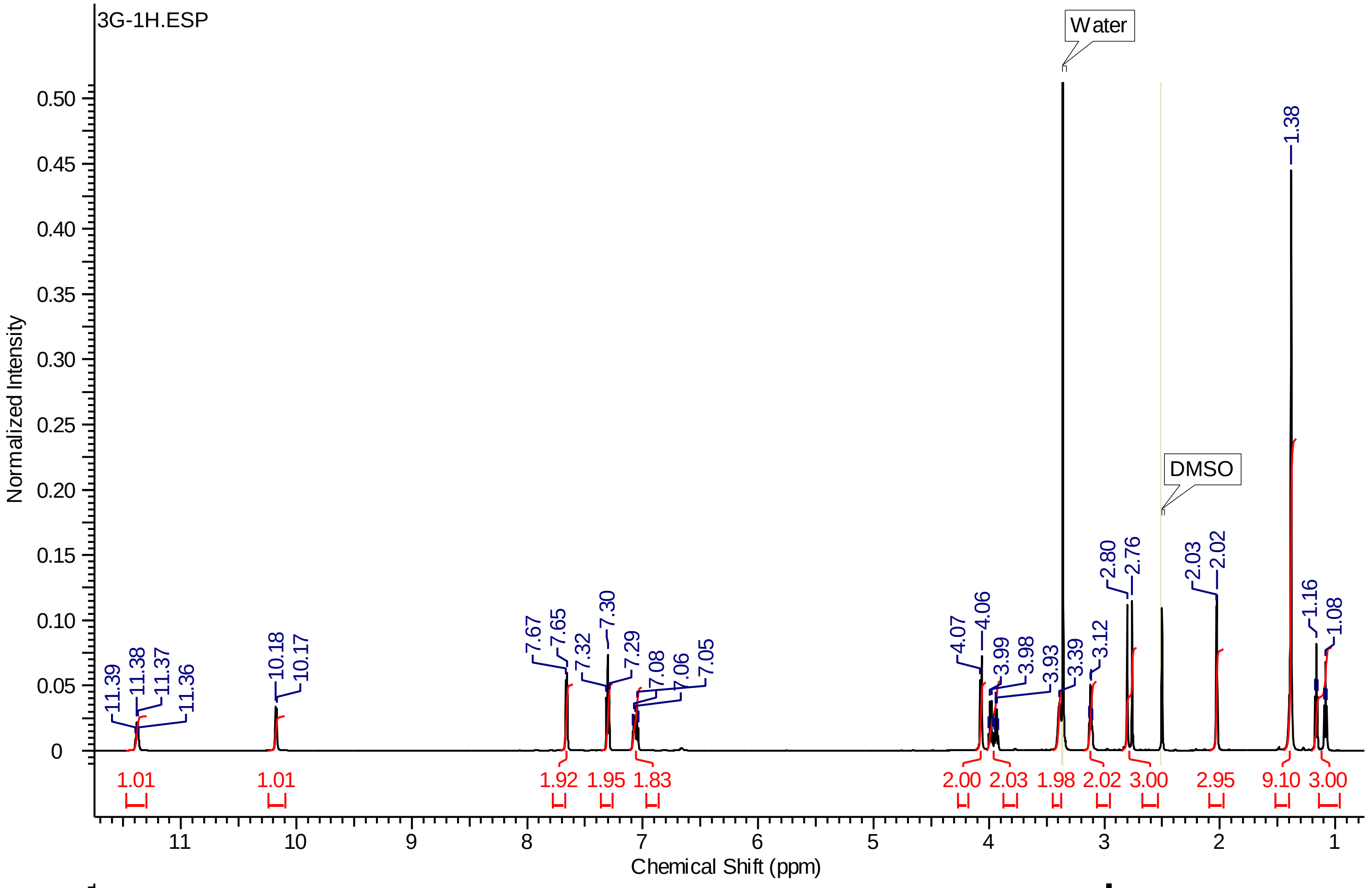
3G-1H.ESP
Water
DMSO
Normalized Intensity
Chemical Shift (ppm)
11.39
11.38
11.37
11.36
10.18
10.17
7.67
7.65
7.32
7.30
7.29
7.08
7.06
7.05
4.07
4.06
3.99
3.98
3.93
3.39
3.12
2.80
2.76
2.03
2.02
1.38
1.16
1.08
1.01
1.01
1.92
1.95
1.83
2.00
2.03
1.98
2.02
3.00
2.95
9.10
3.00

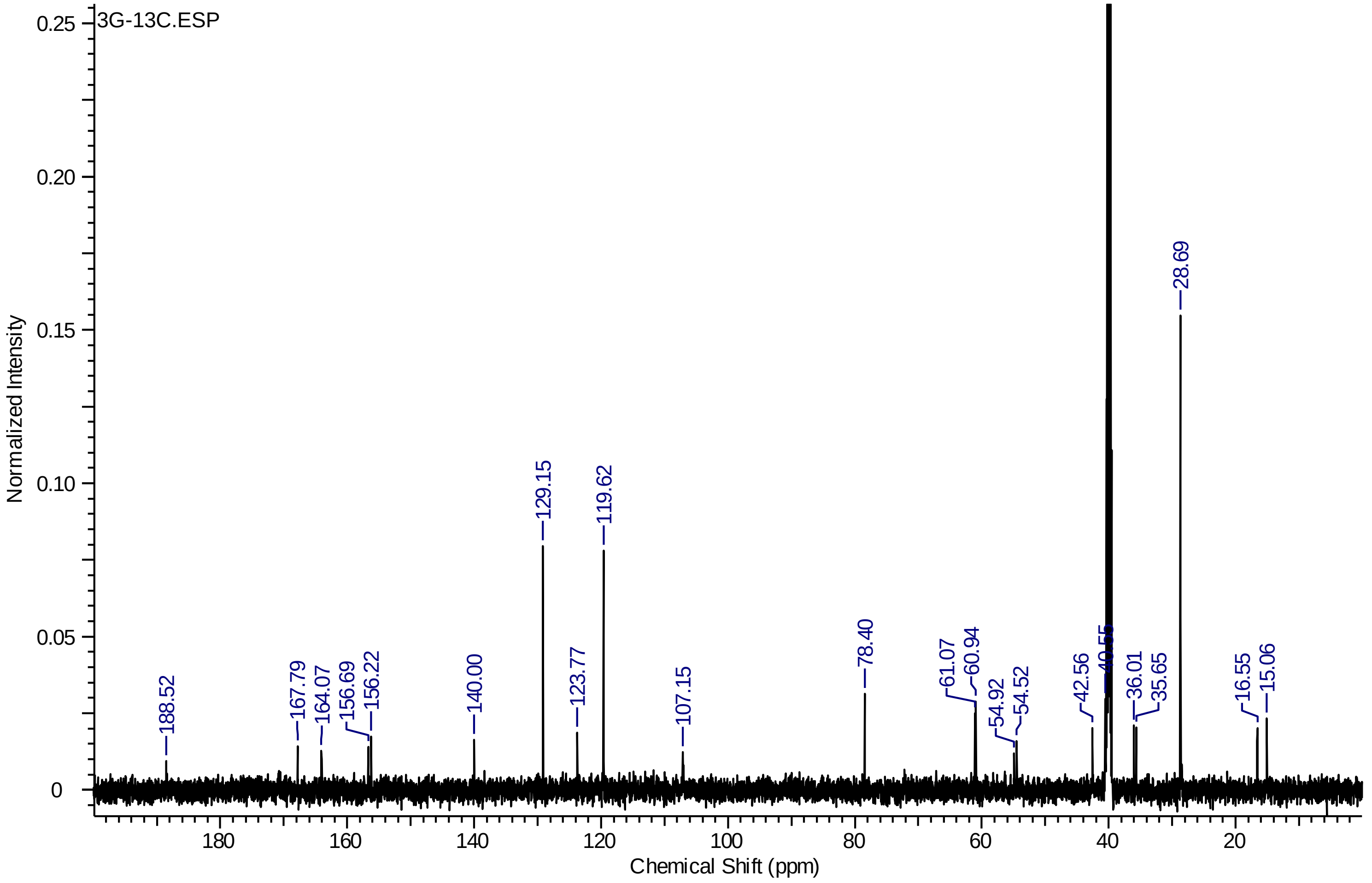
3G-13C.ESP
Normalized Intensity
Chemical Shift (ppm)
188.52
167.79
164.07
156.69
156.22
140.00
129.15
123.77
119.62
107.15
78.40
61.07
60.94
54.92
54.52
42.56
40.55
36.01
35.65
28.69
16.55
15.06

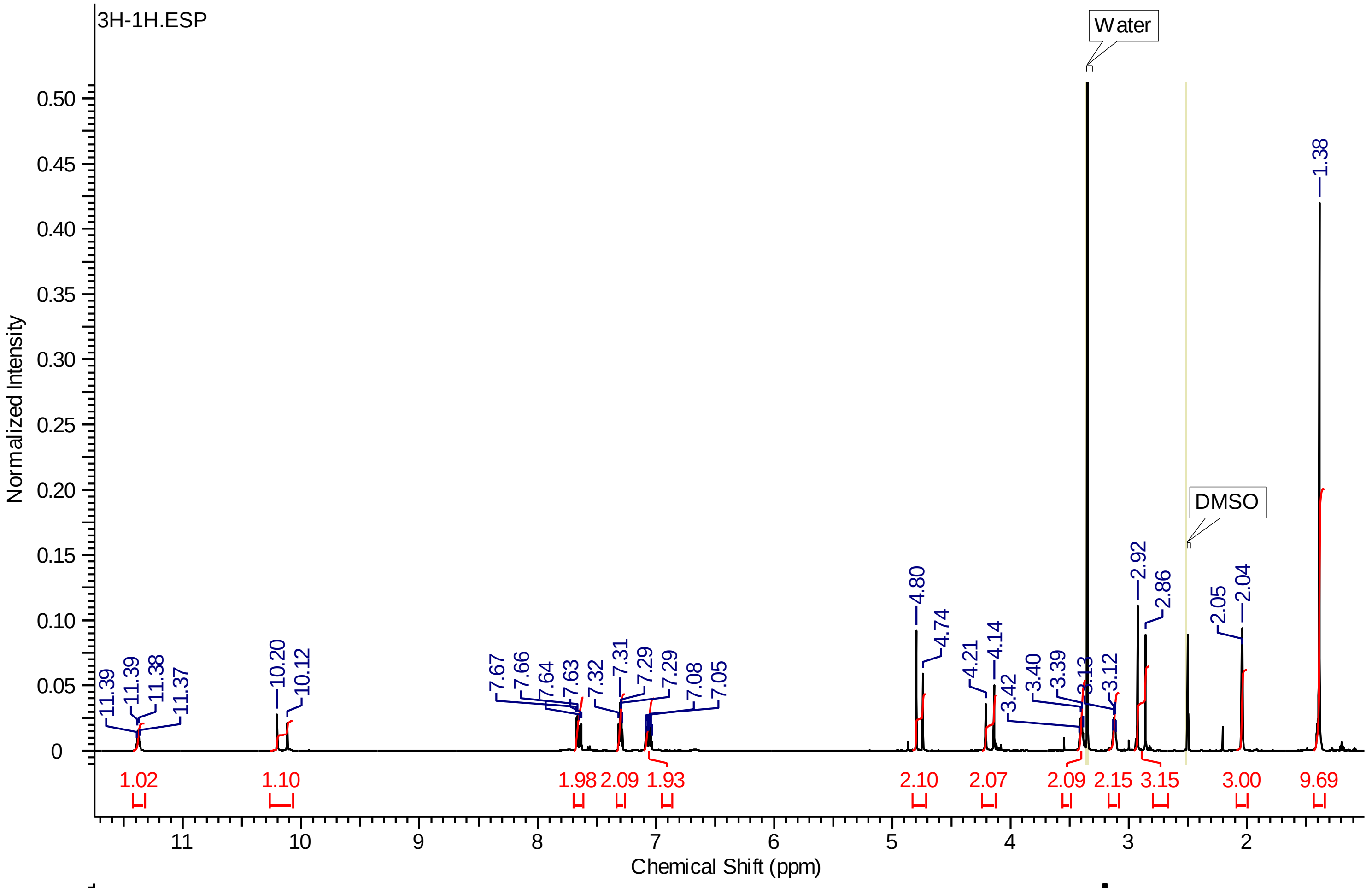
3H-1H.ESP
Water
DMSO
Normalized Intensity
Chemical Shift (ppm)
11.39 11.39 11.38 11.37 10.20 10.12 7.67 7.66 7.64 7.63 7.32 7.31 7.29 7.29 7.08 7.05 4.80 4.74 4.21 4.14 3.42 3.40 3.39 3.13 3.12 2.92 2.86 2.05 2.04 1.38
1.02 1.10 1.98 2.09 1.93 2.10 2.07 2.09 2.15 3.15 3.00 9.69

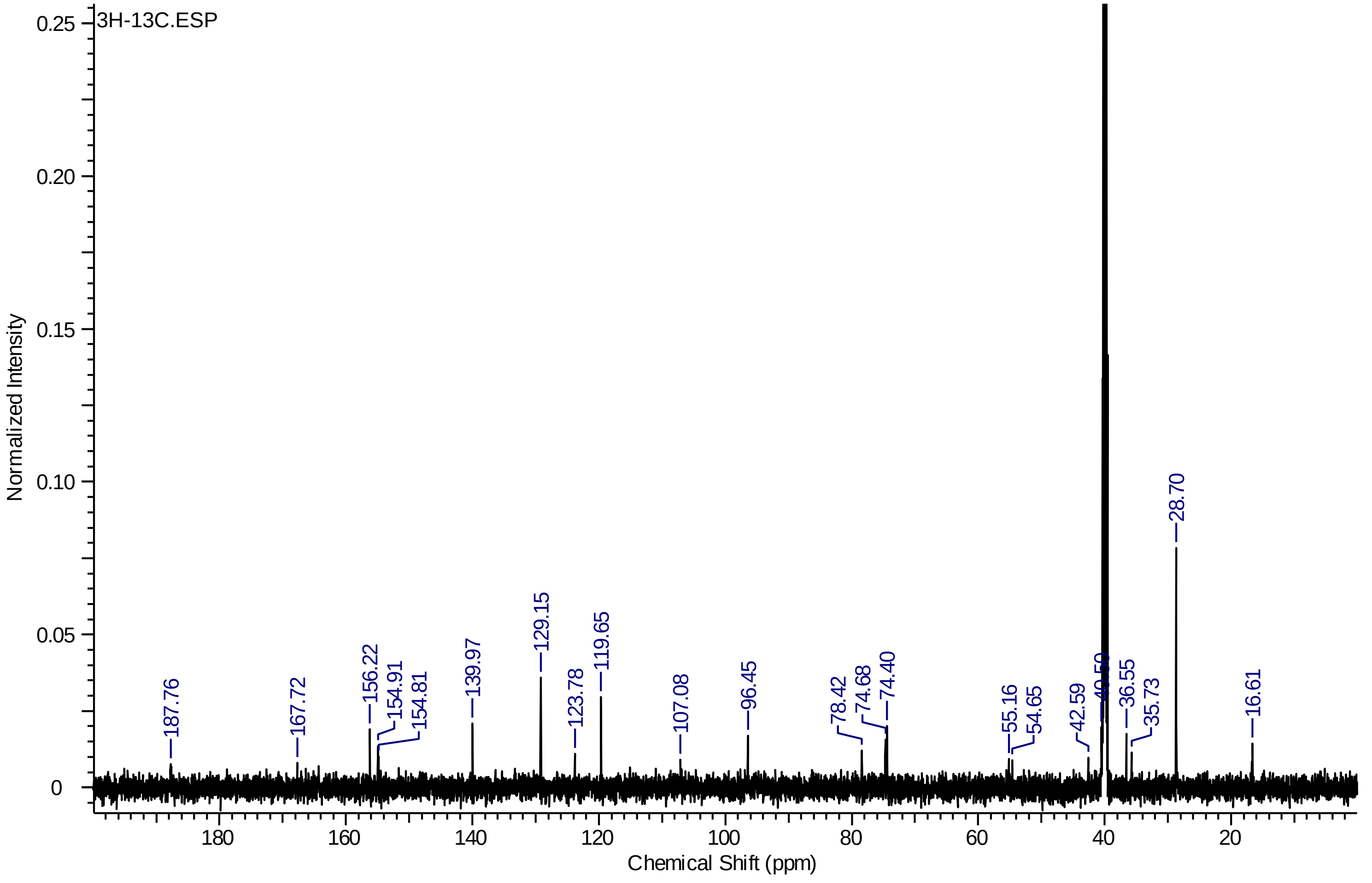
3H-13C.ESP
Normalized Intensity
Chemical Shift (ppm)
187.76 167.72 156.22 154.91 154.81 139.97 129.15 123.78 119.65 107.08 96.45 78.42 74.68 74.40 55.16 54.65 42.59 40.50 36.55 35.73 28.70 16.61

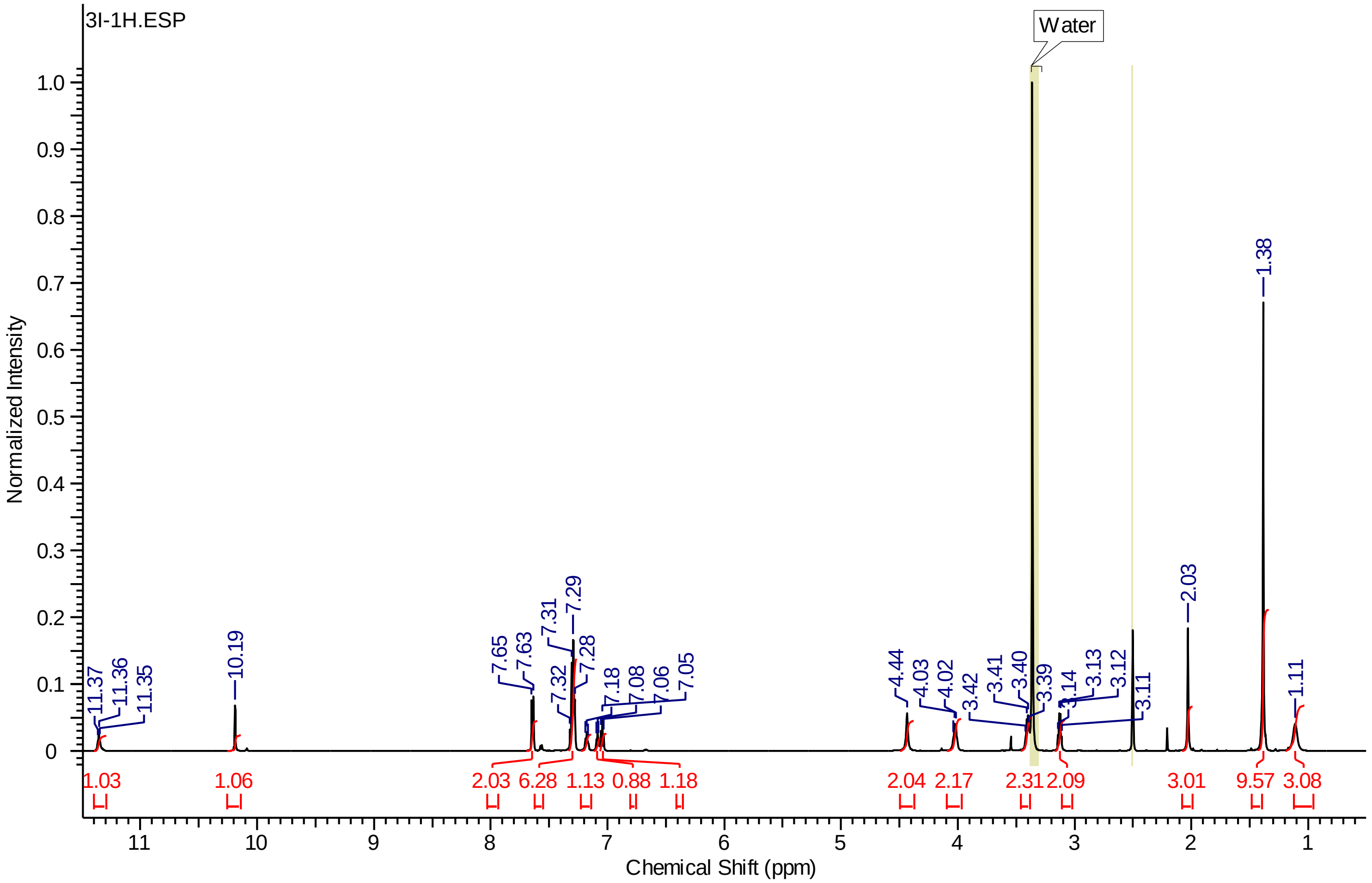
3I-1H.ESP
Water
Normalized Intensity
Chemical Shift (ppm)
11.37 11.36 11.35 10.19 7.65 7.63 7.32 7.31 7.29 7.28 7.18 7.08 7.06 7.05 4.44 4.03 4.02 3.42 3.41 3.40 3.39 3.14 3.13 3.12 3.11 2.03 1.38 1.11
1.03 1.06 2.03 6.28 1.13 0.88 1.18 2.04 2.17 2.31 2.09 3.01 9.57 3.08

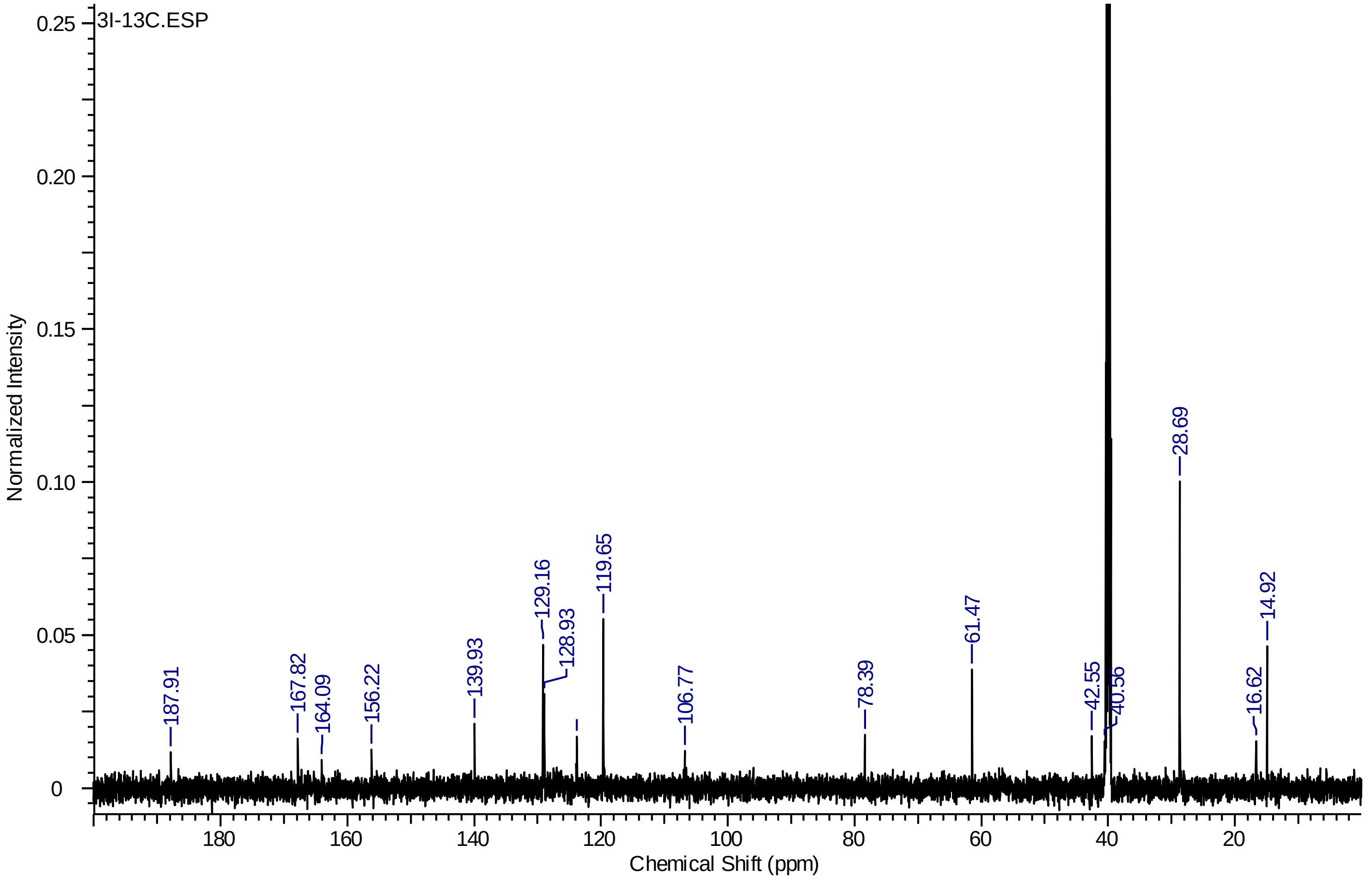
3I-13C.ESP
Normalized Intensity
Chemical Shift (ppm)
187.91 167.82 164.09 156.22 139.93 129.16 128.93 119.65 106.77 78.39 61.47 42.55 40.56 28.69 16.62 14.92

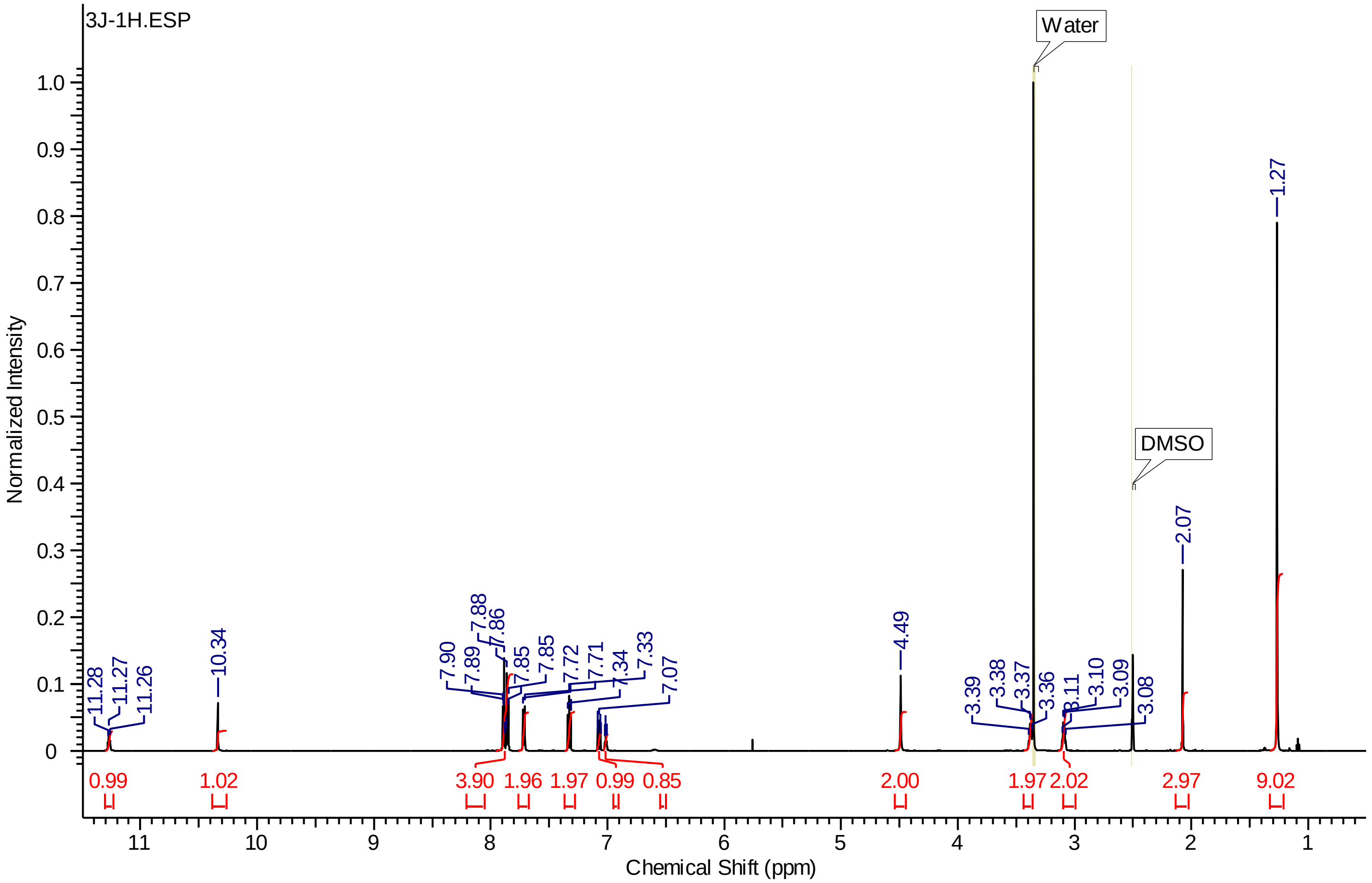
3J-1H.ESP
Water
DMSO
Normalized Intensity
Chemical Shift (ppm)
11.28
11.27
11.26
10.34
7.90
7.89
7.88
7.86
7.85
7.85
7.72
7.71
7.34
7.33
7.07
4.49
3.39
3.38
3.37
3.36
3.11
3.10
3.09
3.08
2.07
1.27
0.99
1.02
3.90
1.96
1.97
0.99
0.85
2.00
1.97
2.02
2.97
9.02

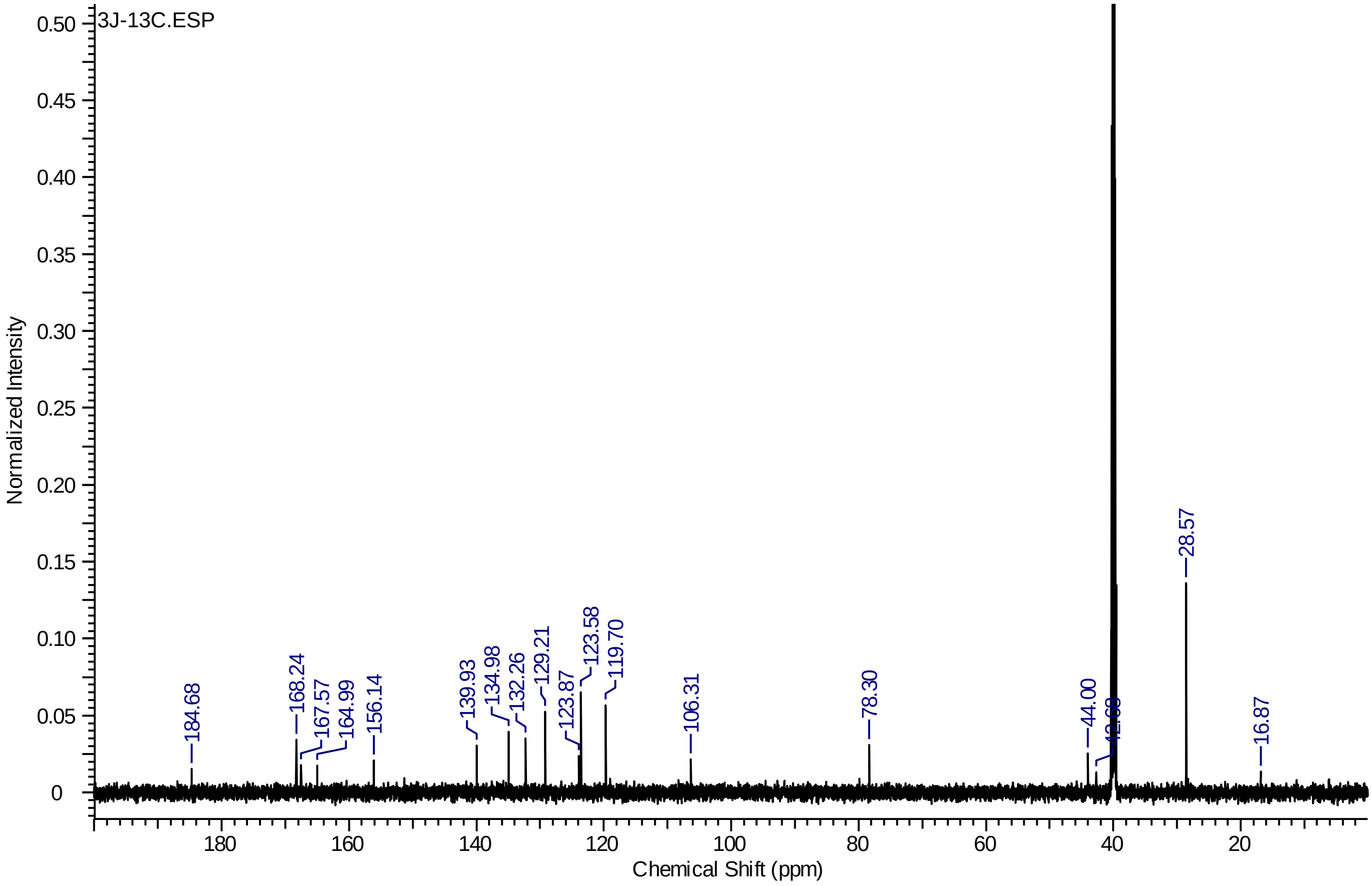
3J-13C.ESP
Normalized Intensity
Chemical Shift (ppm)
184.68
168.24
167.57
164.99
156.14
139.93
134.98
132.26
129.21
123.87
123.58
119.70
106.31
78.30
44.00
16.87
28.57

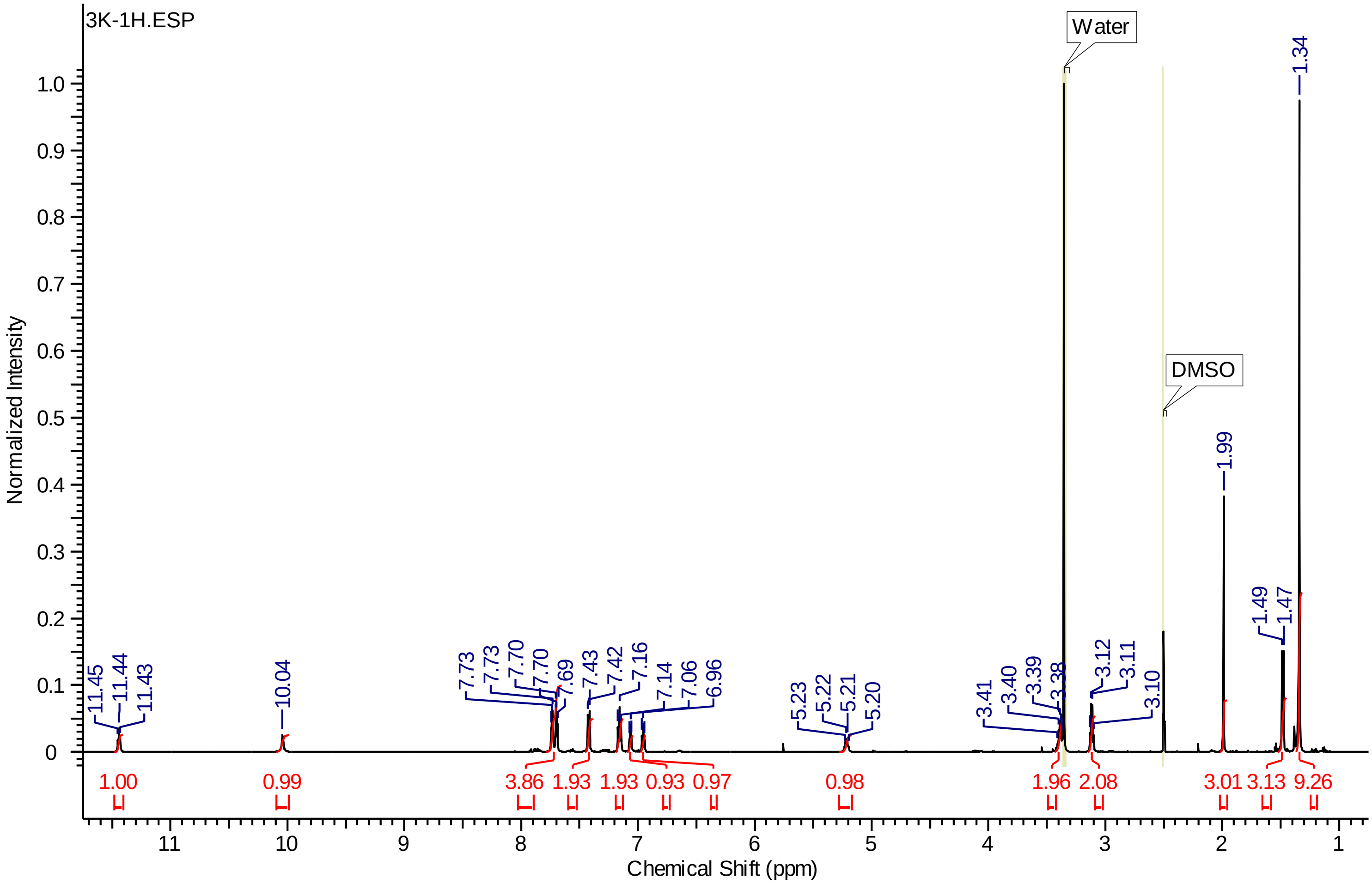
3K-1H.ESP
Water
DMSO
Normalized Intensity
Chemical Shift (ppm)
11.45
11.44
11.43
10.04
7.73
7.73
7.70
7.70
7.69
7.43
7.42
7.16
7.14
7.06
6.96
5.23
5.22
5.21
5.20
3.41
3.40
3.39
3.38
3.12
3.11
3.10
1.99
1.49
1.47
1.34
1.00
0.99
3.86
1.93
1.93
0.93
0.97
0.98
1.96
2.08
3.01
3.13
9.26

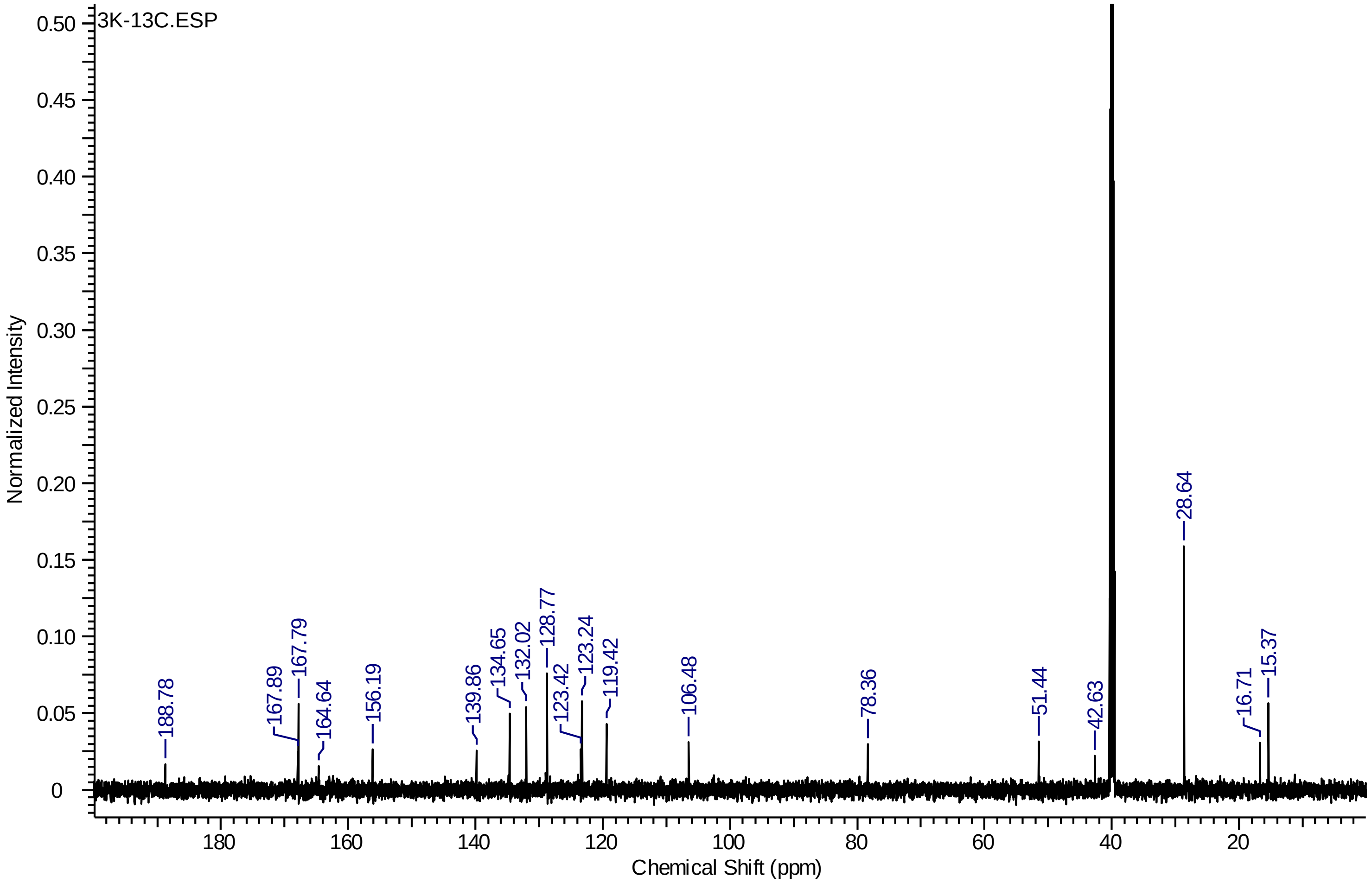
3K-13C.ESP
Normalized Intensity
Chemical Shift (ppm)
188.78
167.89
167.79
164.64
156.19
139.86
134.65
132.02
128.77
123.42
123.24
119.42
106.48
78.36
51.44
42.63
28.64
16.71
15.37

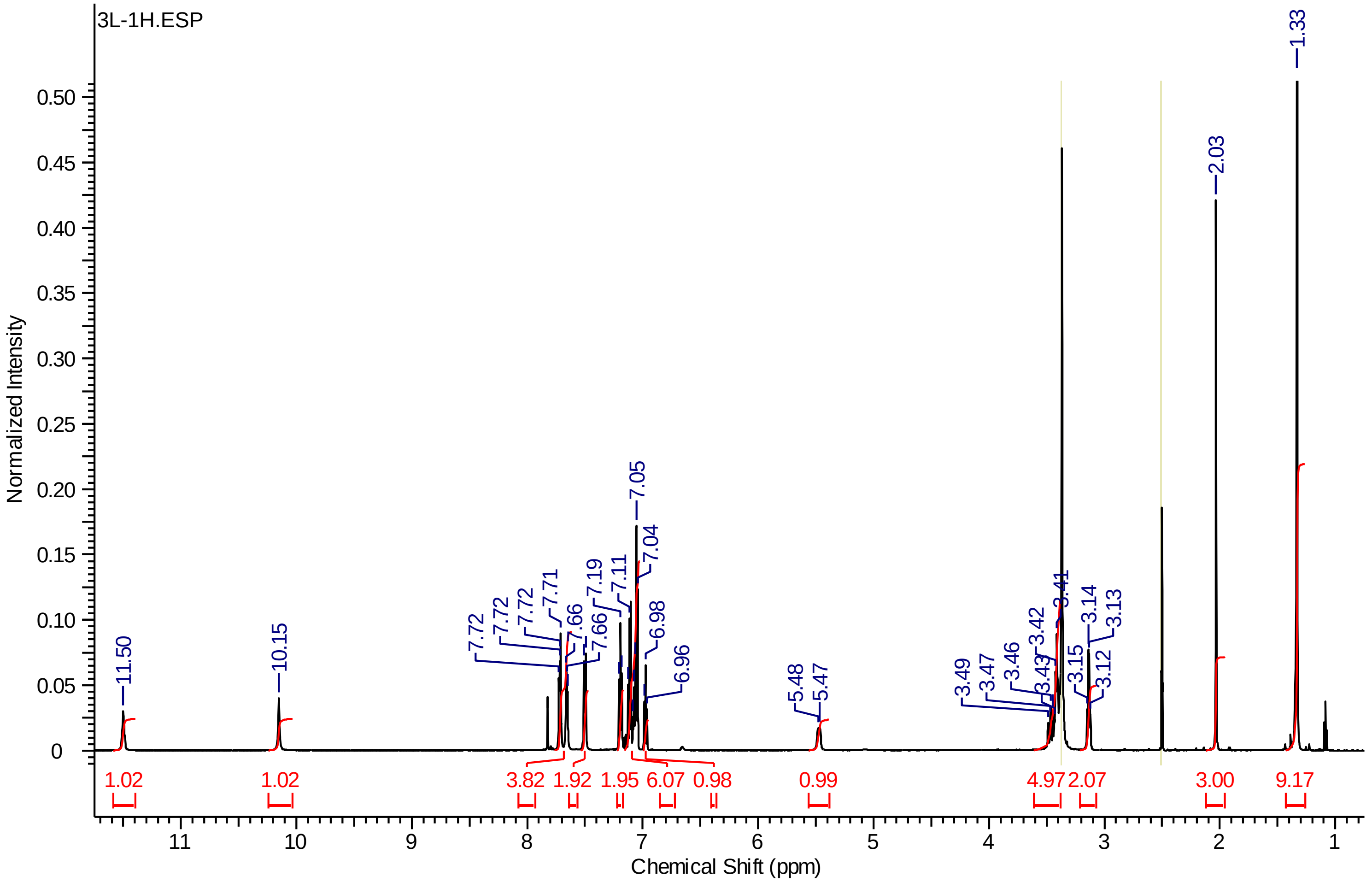
3L-1H.ESP
Normalized Intensity
Chemical Shift (ppm)
11.50
10.15
7.72
7.72
7.72
7.71
7.66
7.66
7.19
7.11
7.05
7.04
6.98
6.96
5.48
5.47
3.49
3.47
3.46
3.43
3.42
3.41
3.15
3.14
3.13
3.12
2.03
1.33
1.02
1.02
3.82
1.92
1.95
6.07
0.98
0.99
4.97
2.07
3.00
9.17

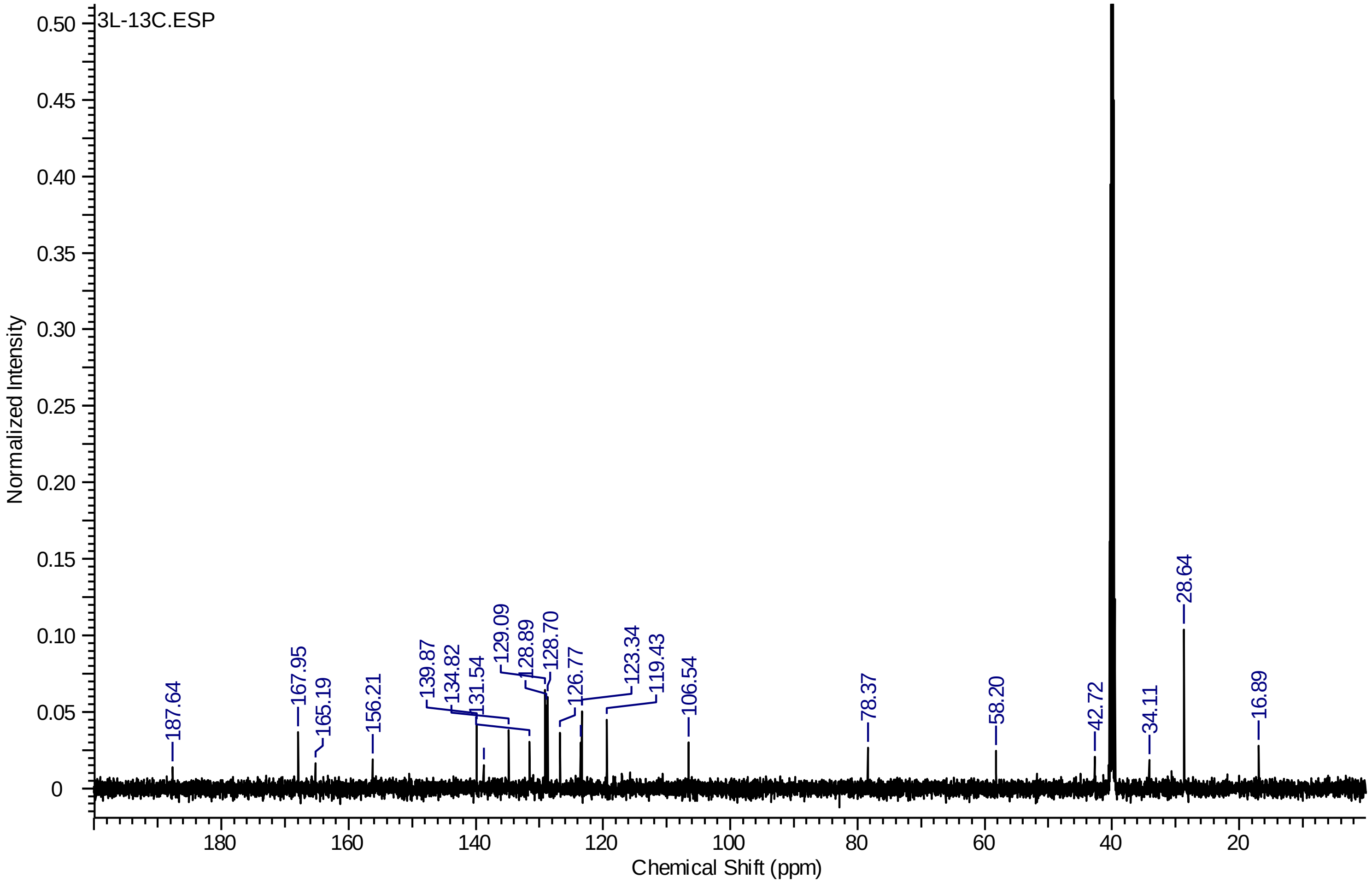
3L-13C.ESP
Normalized Intensity
Chemical Shift (ppm)
187.64
167.95
165.19
156.21
139.87
134.82
131.54
129.09
128.89
128.70
126.77
123.34
119.43
106.54
78.37
58.20
42.72
34.11
28.64
16.89

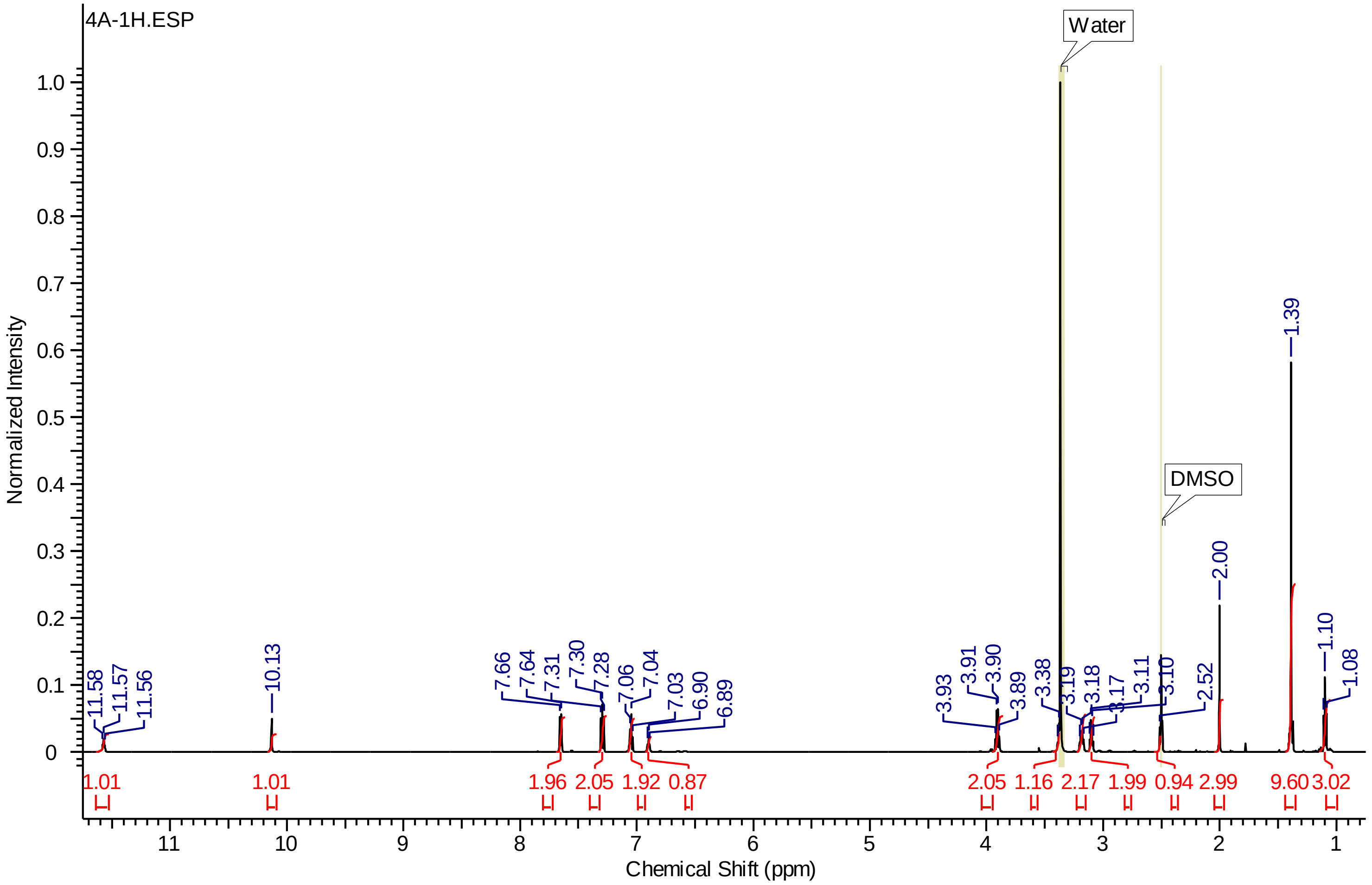
4A-1H.ESP
Water
DMSO
Normalized Intensity
Chemical Shift (ppm)
11.58 11.57 11.56 10.13 7.66 7.64 7.31 7.30 7.28 7.06 7.04 7.03 6.90 6.89 3.93 3.91 3.90 3.89 3.38 3.19 3.18 3.17 3.11 3.10 2.52 2.00 1.39 1.10 1.08
1.01 1.01 1.96 2.05 1.92 0.87 2.05 1.16 2.17 1.99 0.94 2.99 9.60 3.02

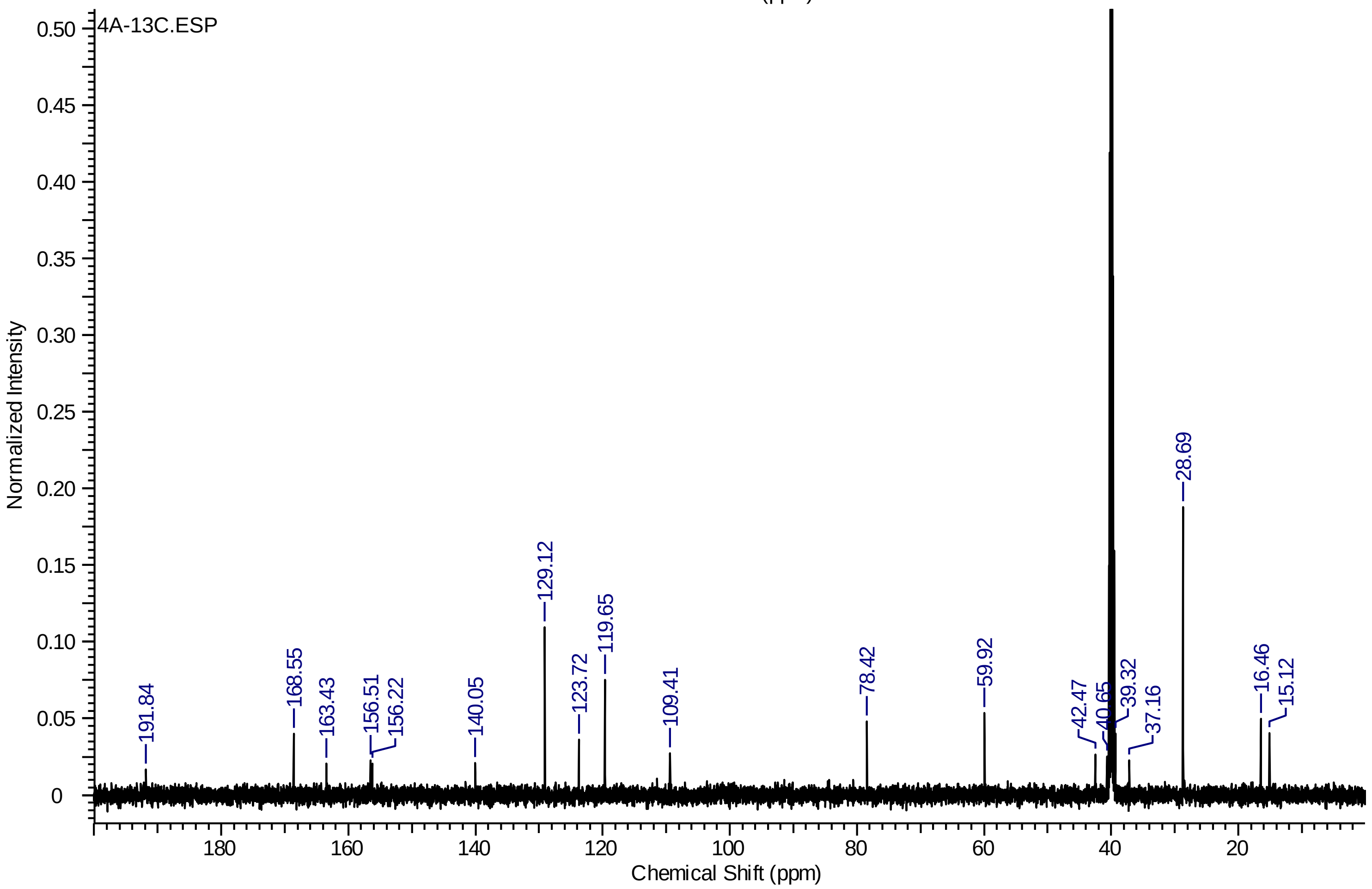
4A-13C.ESP
Normalized Intensity
Chemical Shift (ppm)
191.84 168.55 163.43 156.51 156.22 140.05 129.12 123.72 119.65 109.41 78.42 59.92 42.47 40.65 39.32 37.16 28.69 16.46 15.12

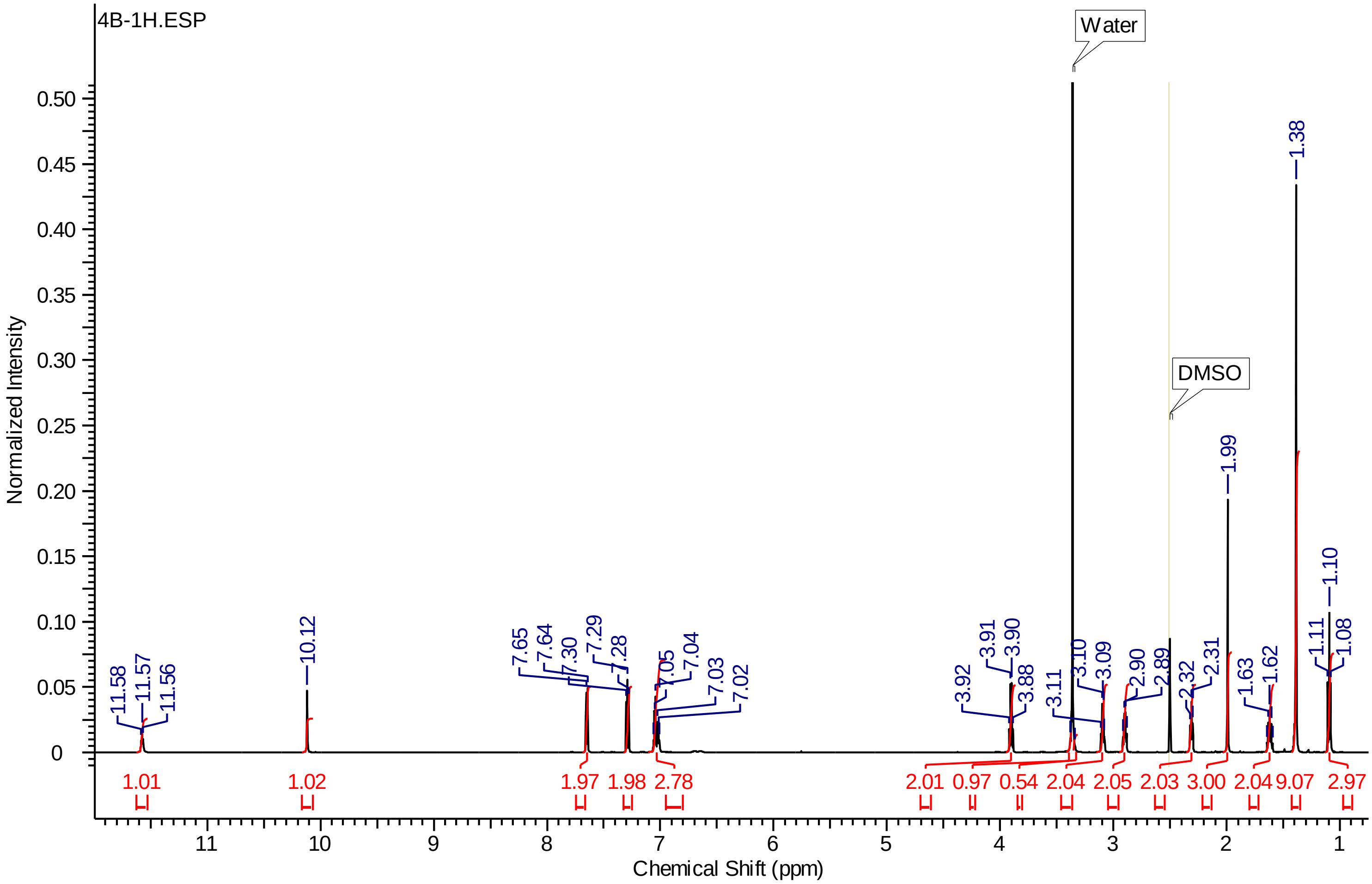
4B-1H.ESP
Water
DMSO
Normalized Intensity
Chemical Shift (ppm)
11.58 11.57 11.56 10.12 7.65 7.64 7.30 7.29 7.28 7.05 7.04 7.03 7.02 3.92 3.91 3.90 3.88 3.11 3.10 3.09 2.90 2.89 2.32 2.31 1.99 1.63 1.62 1.38 1.11 1.10 1.08
1.01 1.02 1.97 1.98 2.78 2.01 0.97 0.54 2.04 2.05 2.03 3.00 2.04 9.07 2.97

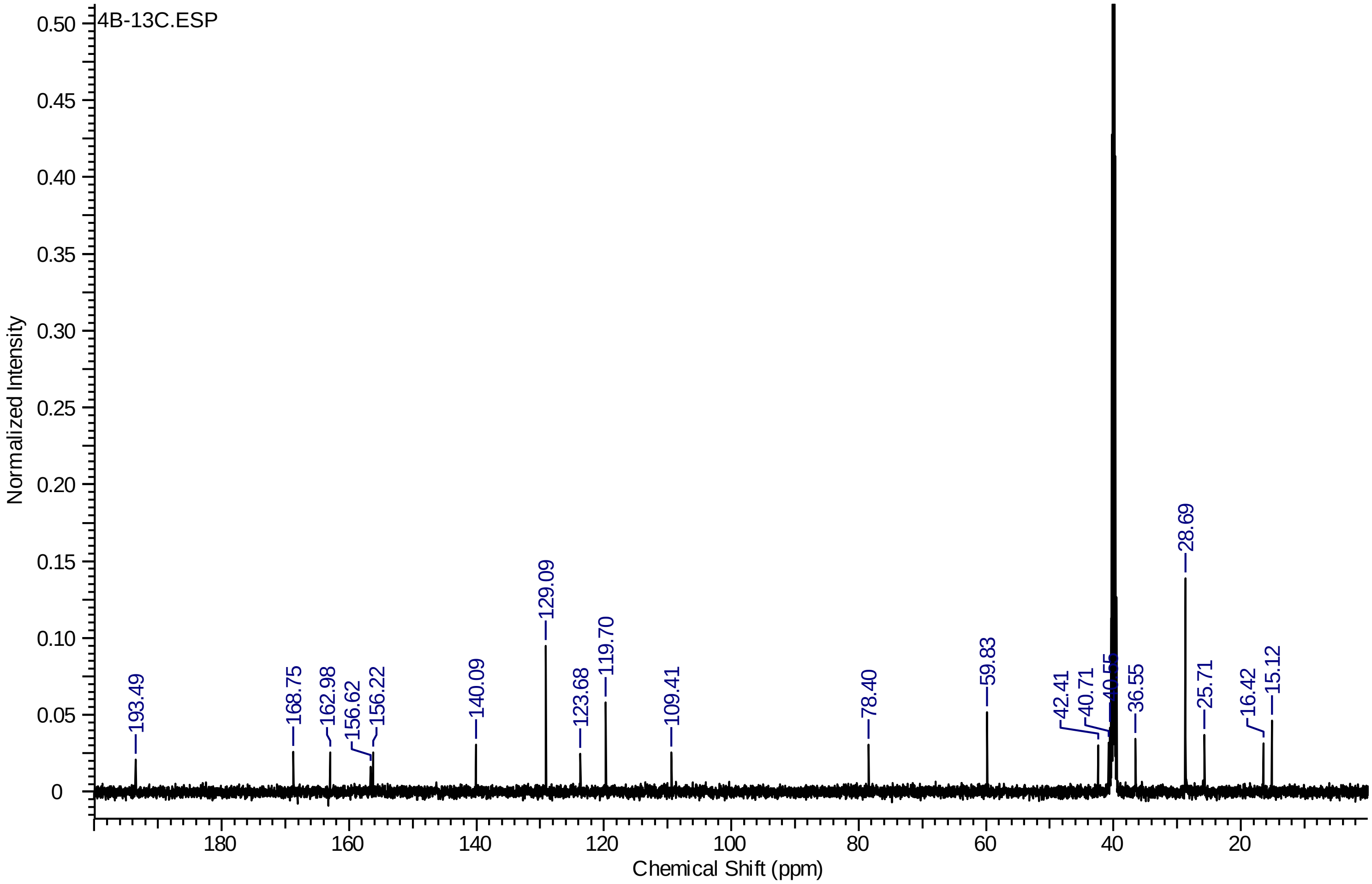
4B-13C.ESP
Normalized Intensity
Chemical Shift (ppm)
193.49 168.75 162.98 156.62 156.22 140.09 129.09 123.68 119.70 109.41 78.40 59.83 42.41 40.71 40.55 36.55 28.69 25.71 16.42 15.12

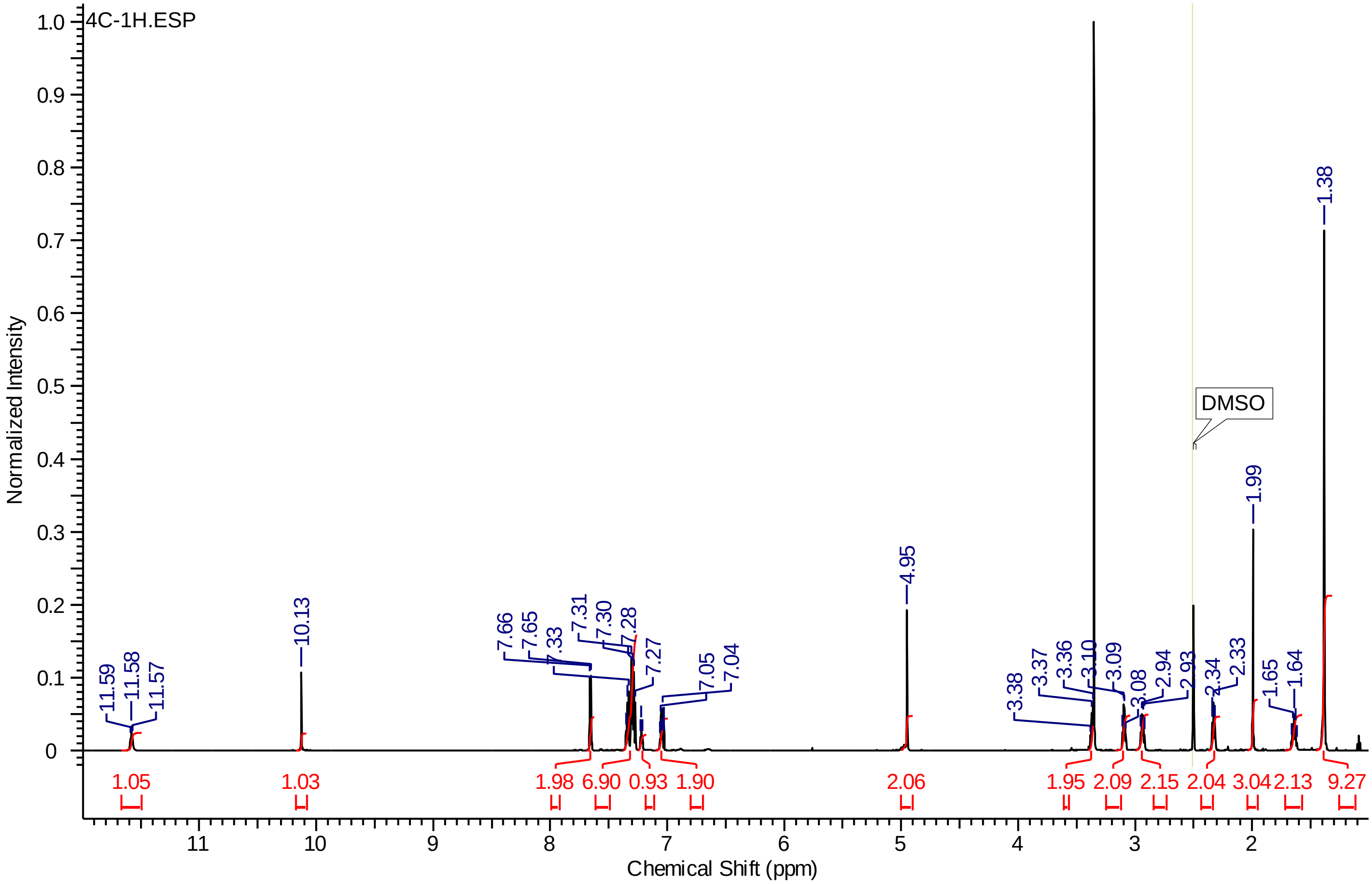
4C-1H.ESP
DMSO
Normalized Intensity
Chemical Shift (ppm)
11.59 11.58 11.57 10.13 7.66 7.65 7.33 7.31 7.30 7.28 7.27 7.05 7.04 4.95 3.38 3.37 3.36 3.10 3.09 3.08 2.94 2.93 2.34 2.33 1.99 1.65 1.64 1.38
1.05 1.03 1.98 6.90 0.93 1.90 2.06 1.95 2.09 2.15 2.04 3.04 2.13 9.27

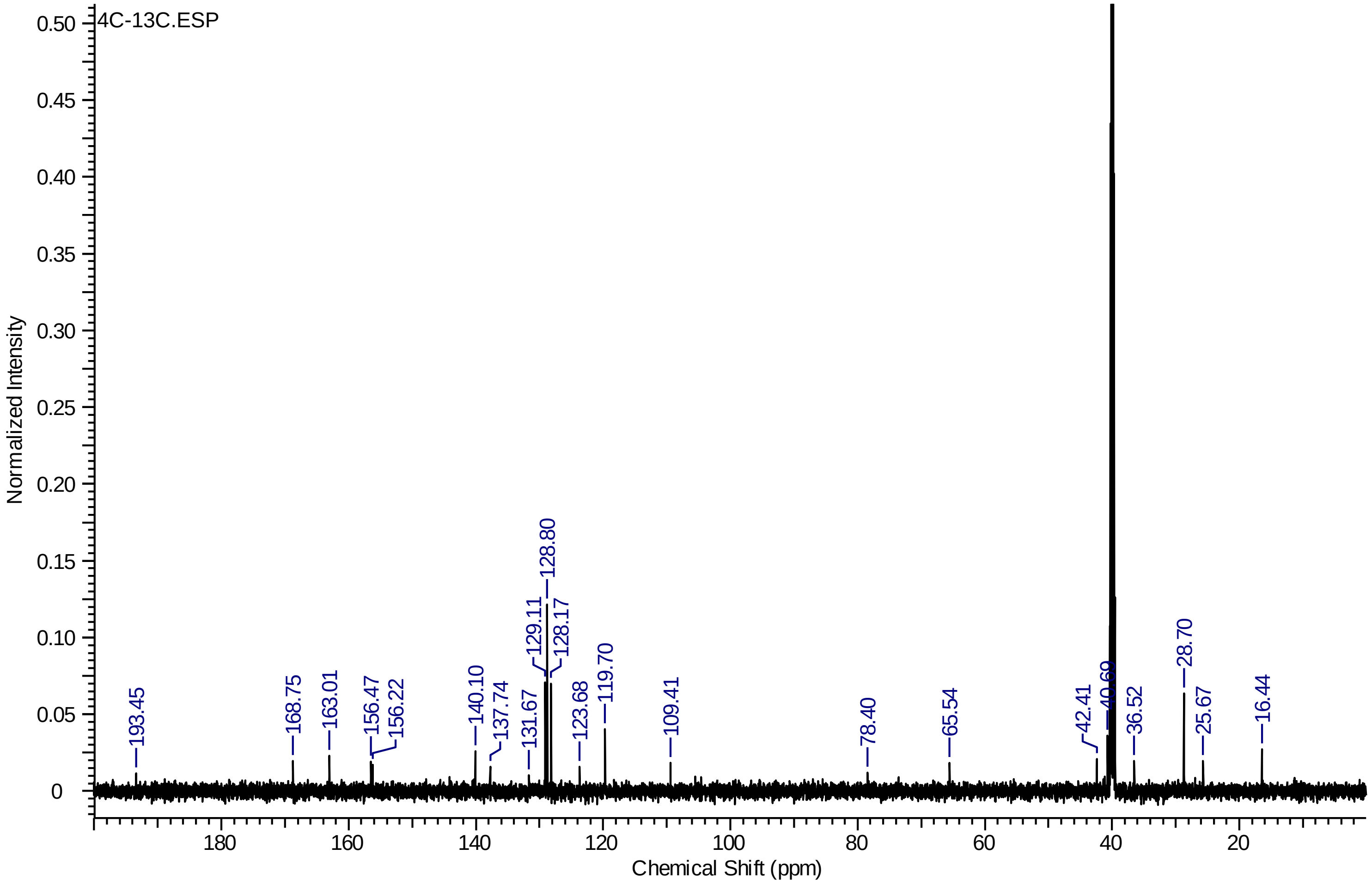
4C-13C.ESP
Normalized Intensity
Chemical Shift (ppm)
193.45 168.75 163.01 156.47 156.22 140.10 137.74 131.67 129.11 128.80 128.17 123.68 119.70 109.41 78.40 65.54 42.41 40.69 36.52 28.70 25.67 16.44

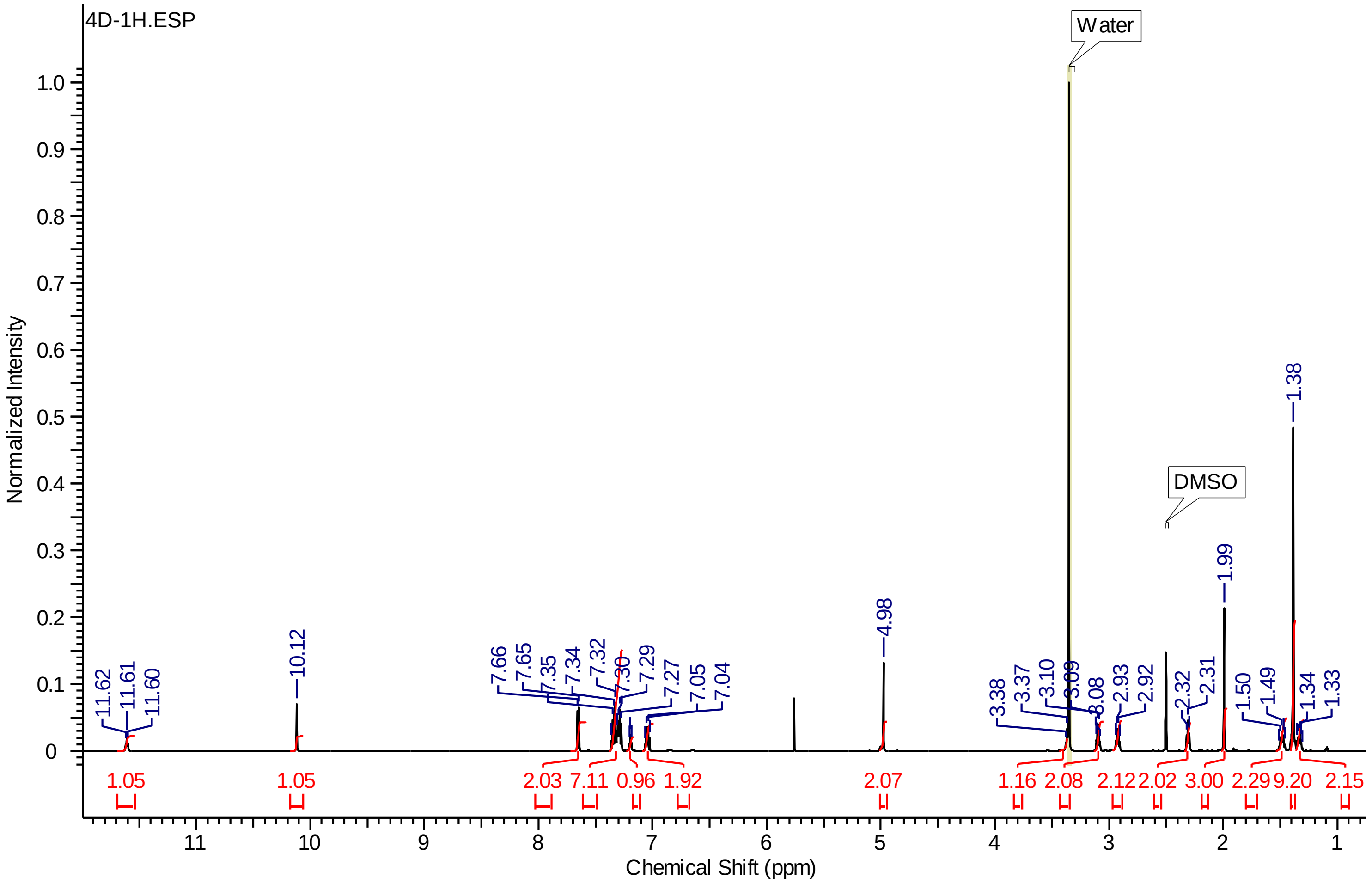
4D-1H.ESP
Water
DMSO
Normalized Intensity
Chemical Shift (ppm)

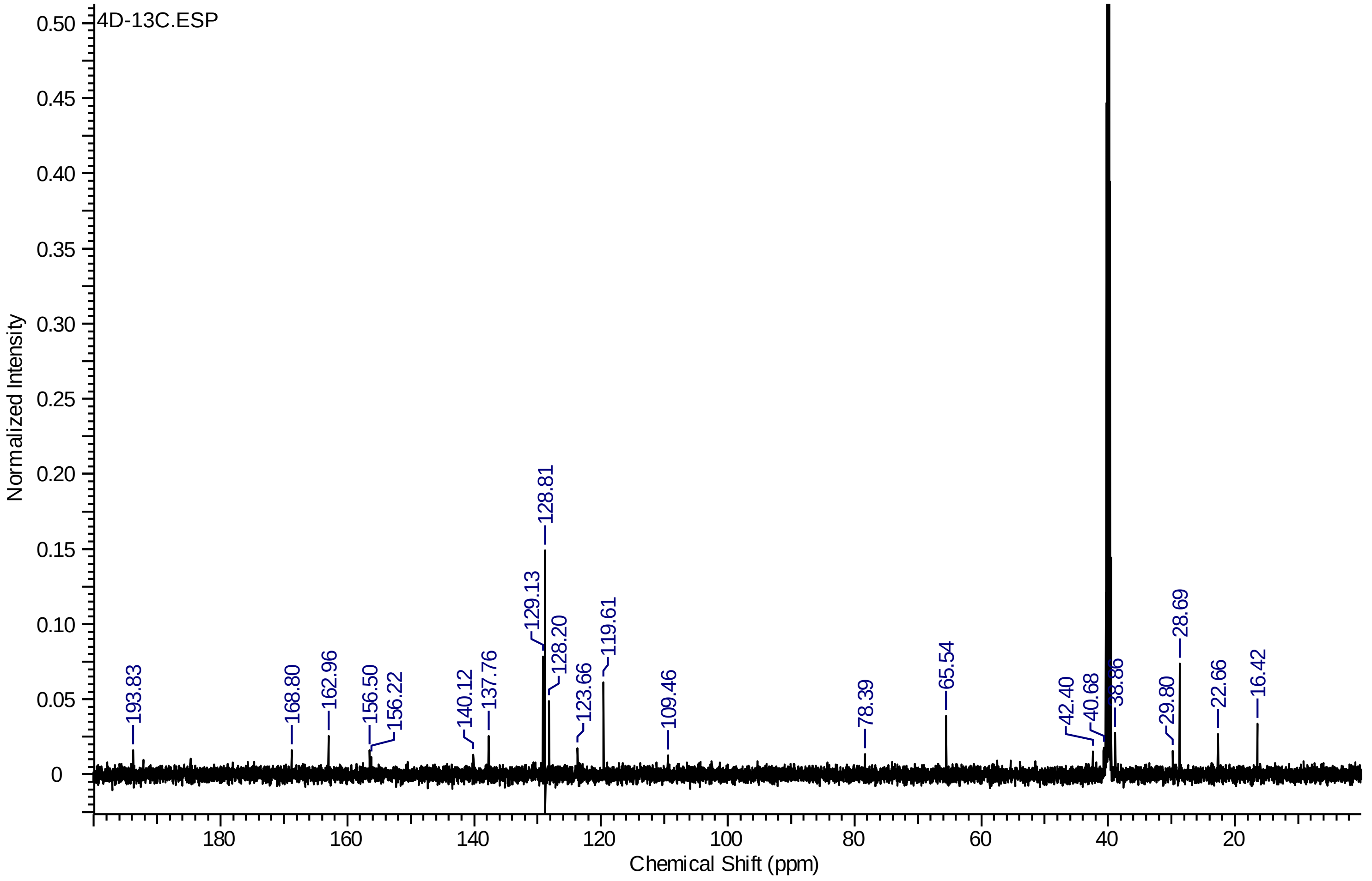
4D-13C.ESP
Normalized Intensity
Chemical Shift (ppm)

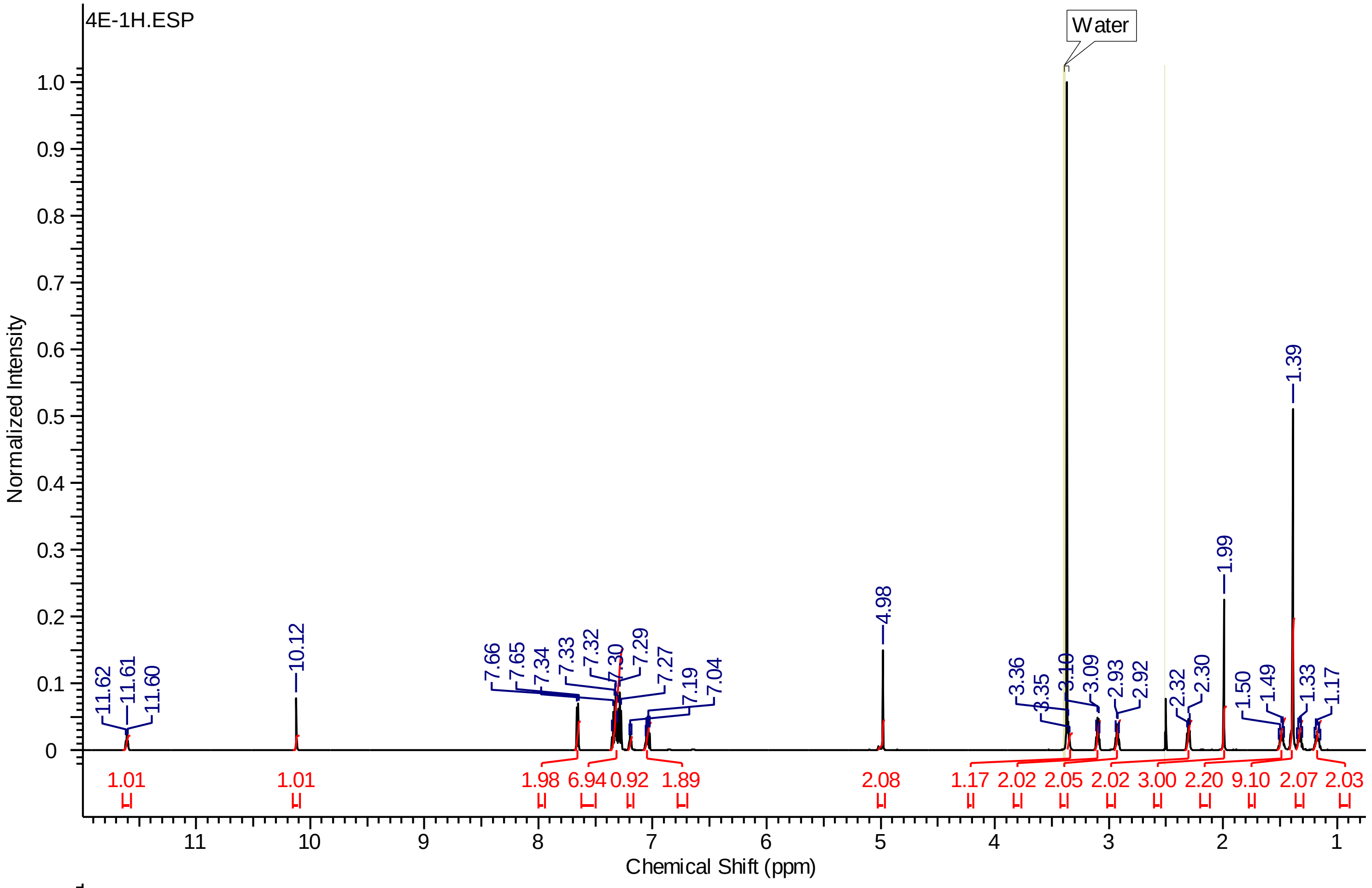
4E-1H.ESP
Water
Normalized Intensity
Chemical Shift (ppm)

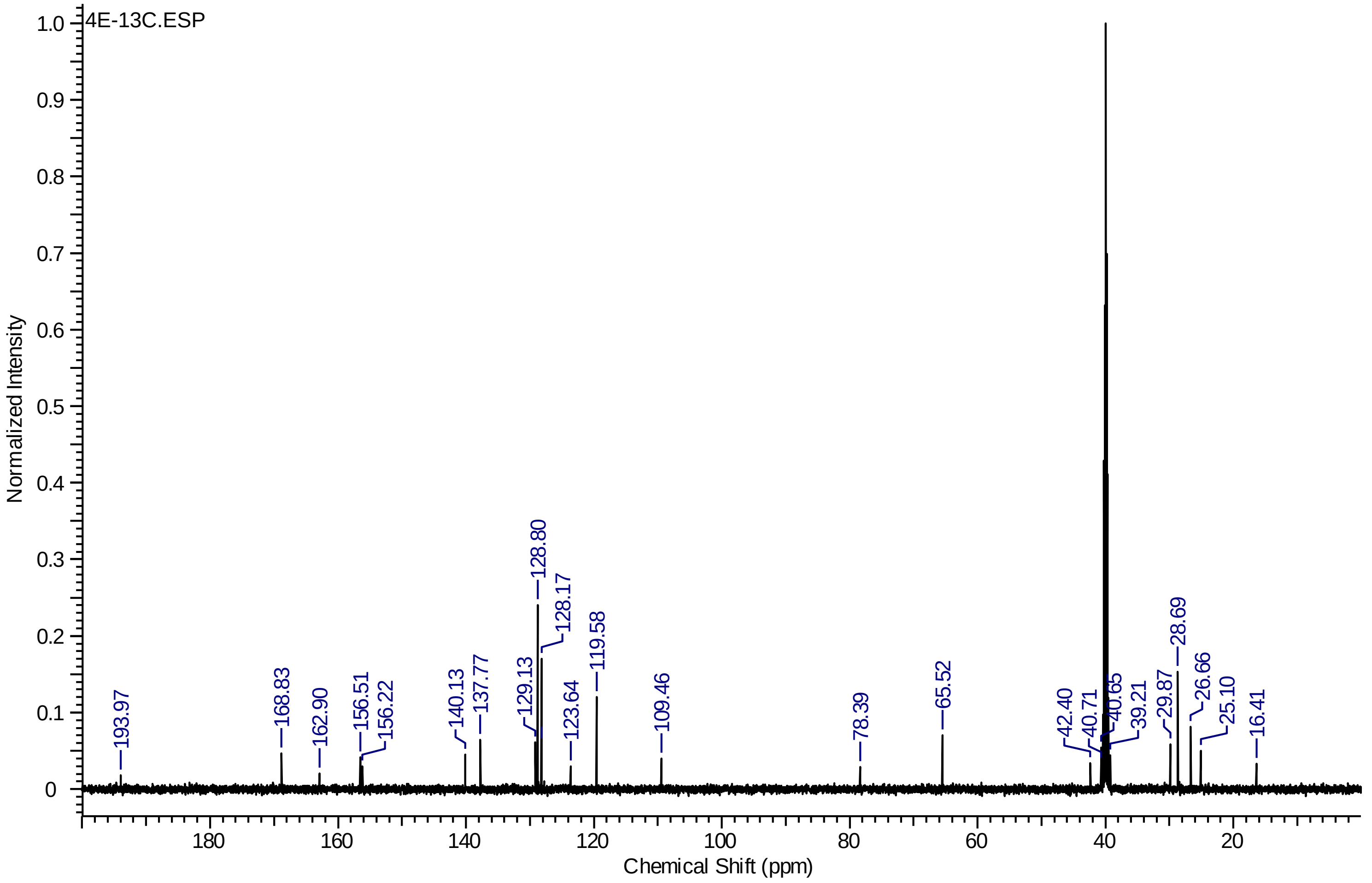
4E-13C.ESP
Normalized Intensity
Chemical Shift (ppm)

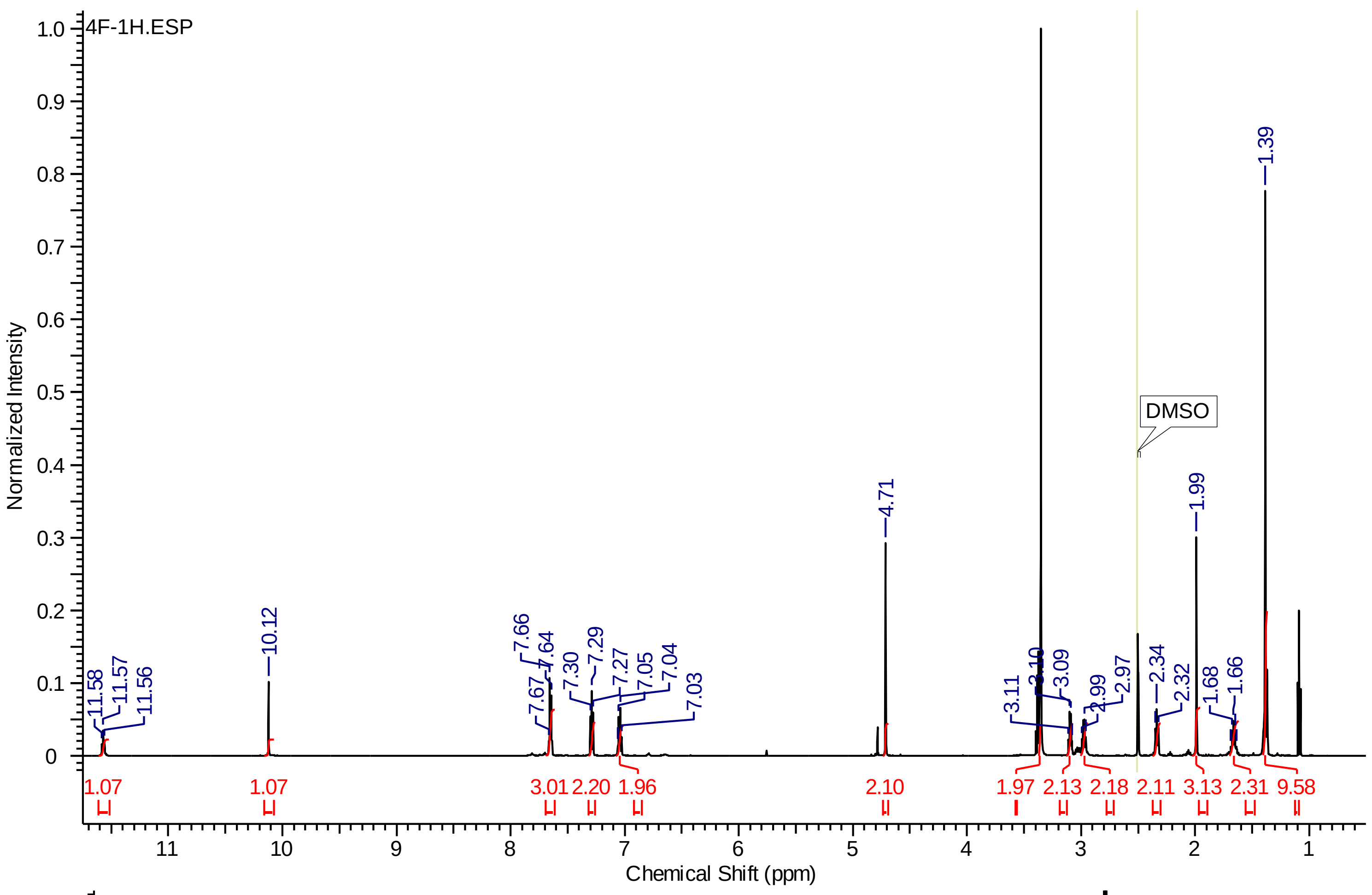

4F-1H.ESP
Normalized Intensity
Chemical Shift (ppm)
DMSO
11.58
11.57
11.56
10.12
7.67
7.66
7.64
7.30
7.29
7.27
7.05
7.04
7.03
4.71
3.11
3.10
3.09
2.99
2.97
2.34
2.32
1.99
1.68
1.66
1.39
1.07
1.07
3.01
2.20
1.96
2.10
1.97
2.13
2.18
2.11
3.13
2.31
9.58

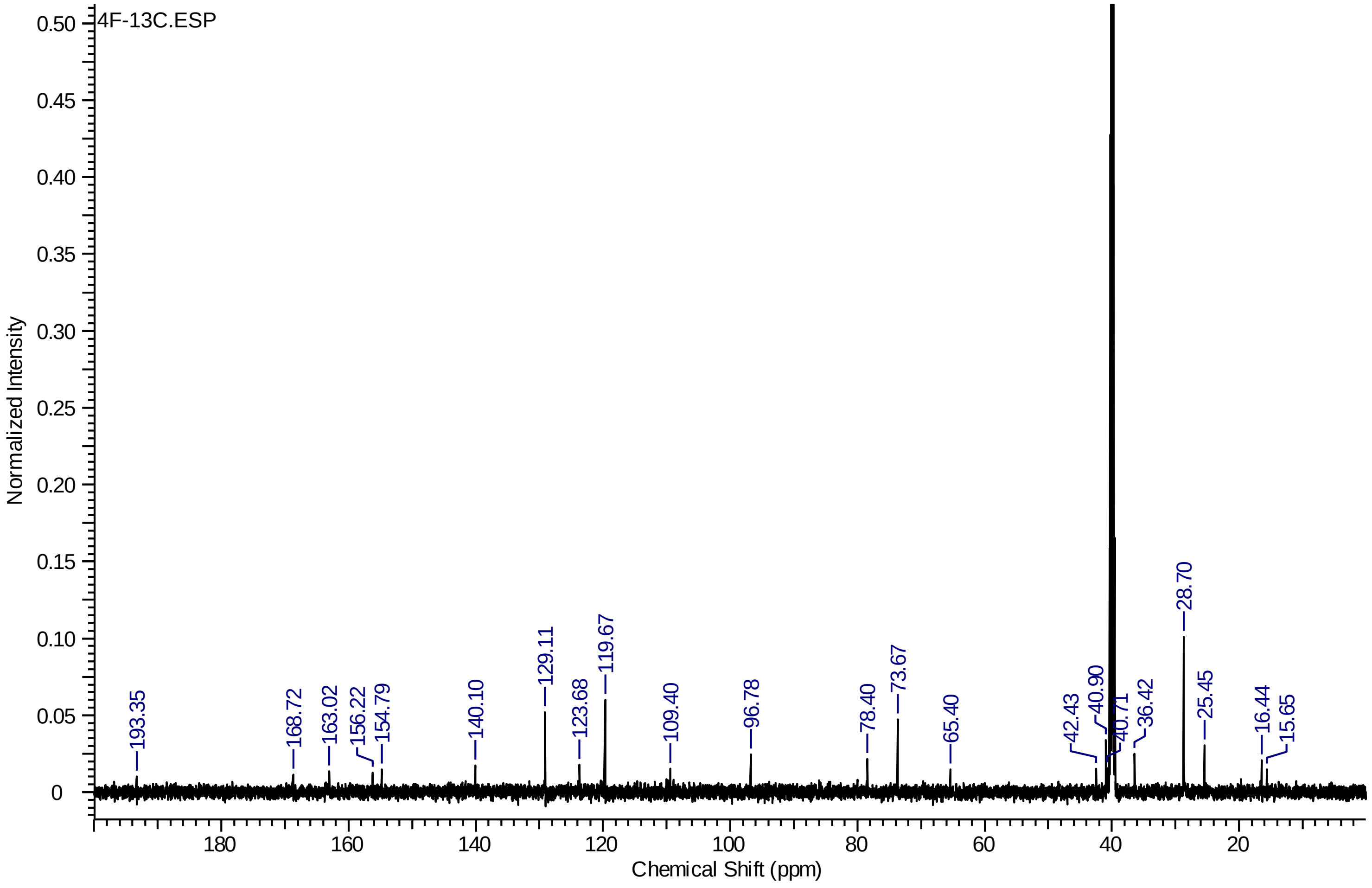

4F-13C.ESP
Normalized Intensity
Chemical Shift (ppm)
193.35
168.72
163.02
156.22
154.79
140.10
129.11
123.68
119.67
109.40
96.78
78.40
73.67
65.40
42.43
40.90
40.71
36.42
28.70
25.45
16.44
15.65

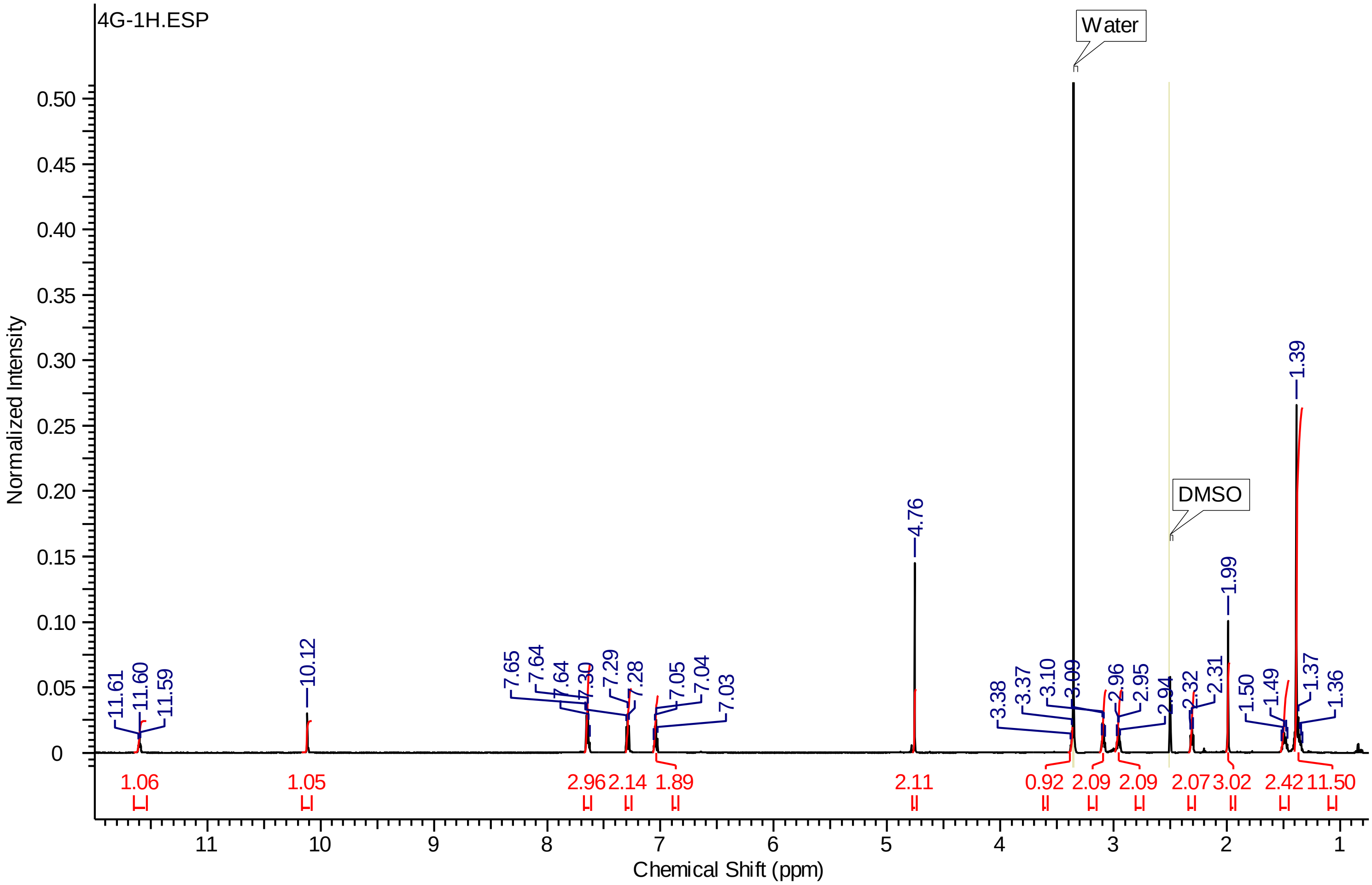
4G-1H.ESP
Water
DMSO
Normalized Intensity
Chemical Shift (ppm)
11.61
11.60
11.59
10.12
7.65
7.64
7.64
7.30
7.29
7.28
7.05
7.04
7.03
4.76
3.38
3.37
3.10
3.09
2.96
2.95
2.94
2.32
2.31
1.99
1.50
1.49
1.39
1.37
1.36
1.06
1.05
2.96
2.14
1.89
2.11
0.92
2.09
2.09
2.07
3.02
2.42
11.50

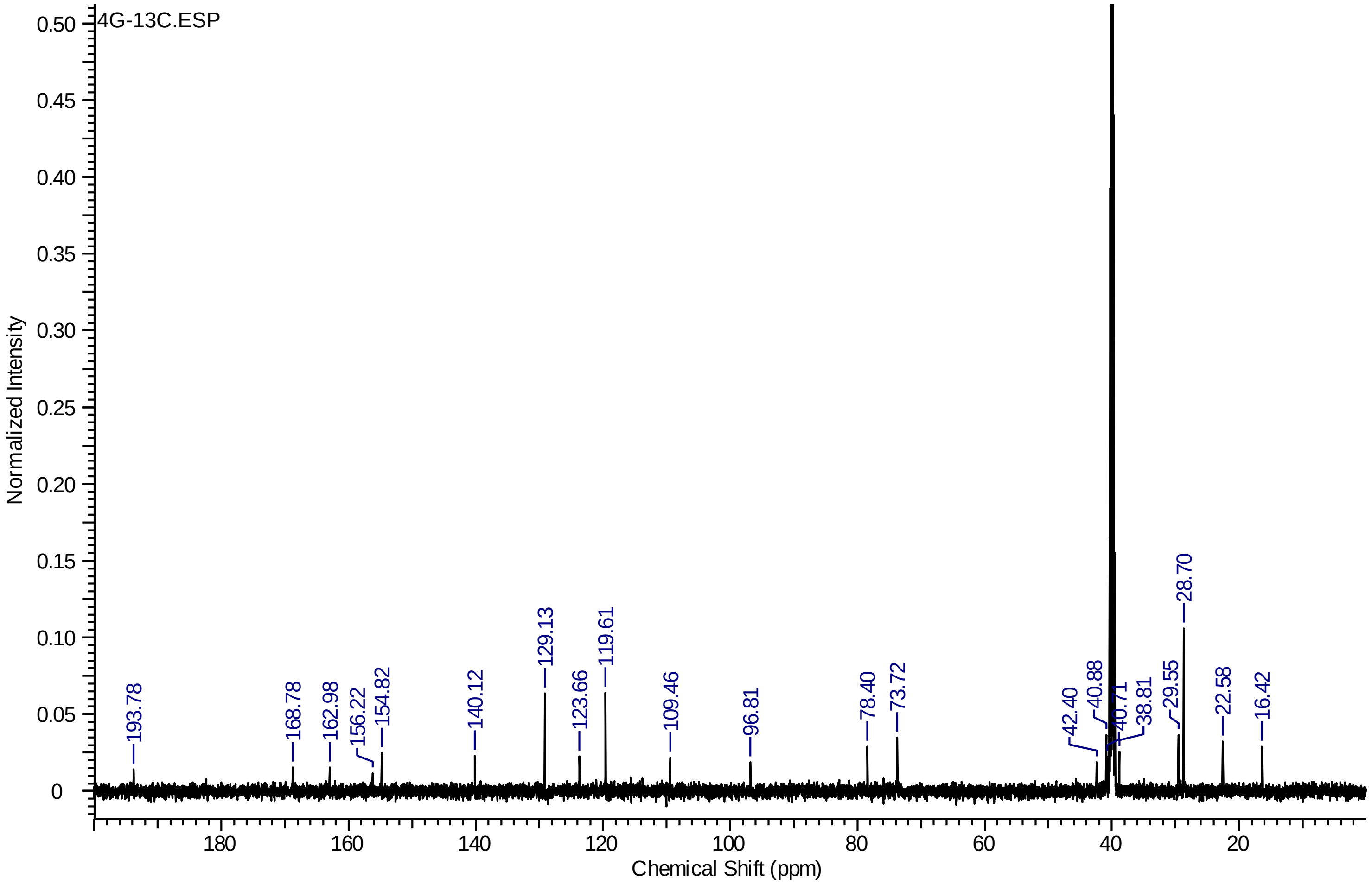
4G-13C.ESP
Normalized Intensity
Chemical Shift (ppm)
193.78
168.78
162.98
156.22
154.82
140.12
129.13
123.66
119.61
109.46
96.81
78.40
73.72
42.40
40.88
40.71
38.81
29.55
28.70
22.58
16.42

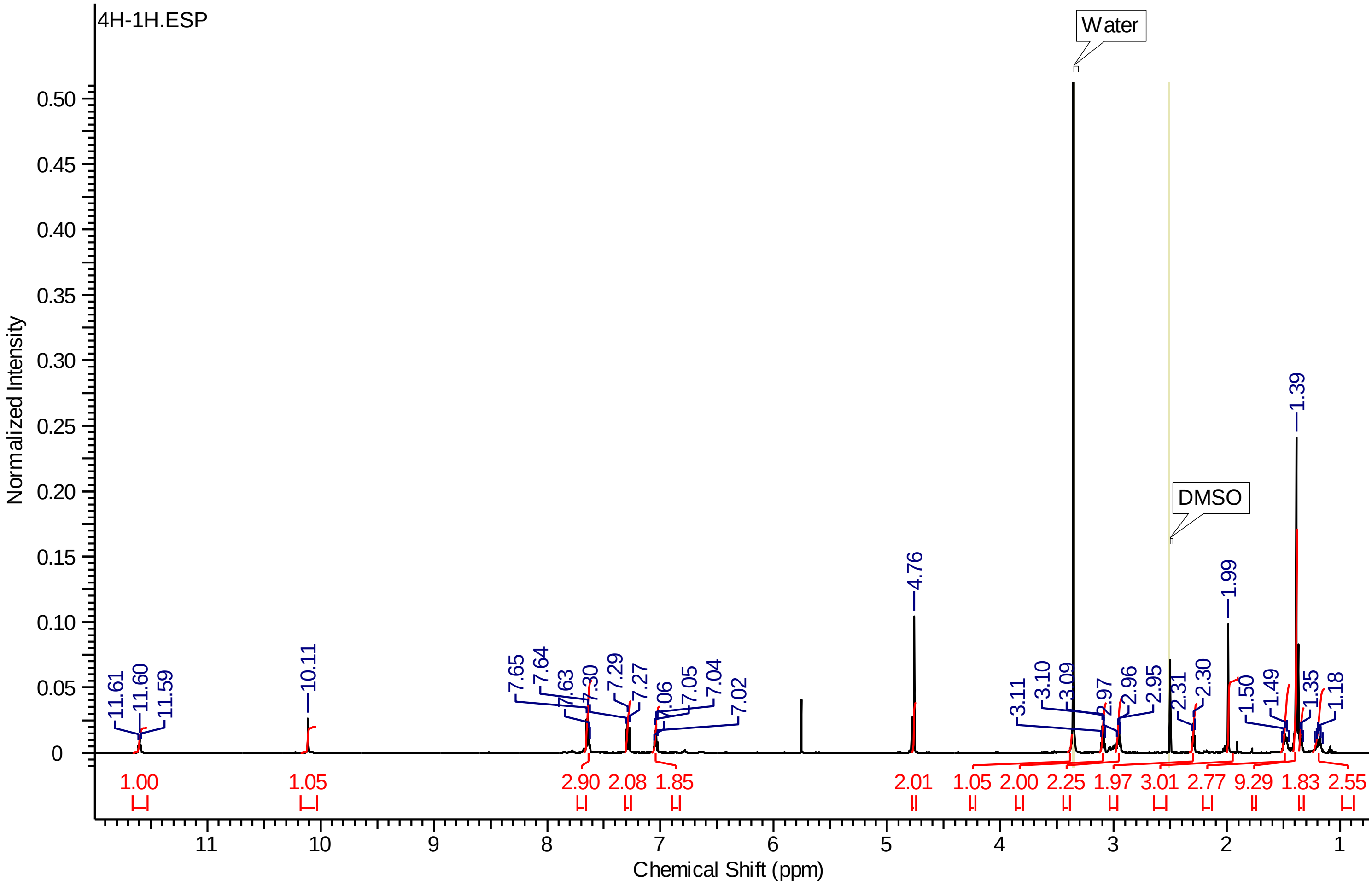
4H-1H.ESP
Water
DMSO
Normalized Intensity
Chemical Shift (ppm)
11.61 11.60 11.59 10.11 7.65 7.64 7.63 7.30 7.29 7.27 7.06 7.05 7.04 7.02 4.76 3.11 3.10 3.09 2.97 2.96 2.95 2.31 2.30 1.99 1.50 1.49 1.39 1.35 1.18
1.00 1.05 2.90 2.08 1.85 2.01 1.05 2.00 2.25 1.97 3.01 2.77 9.29 1.83 2.55

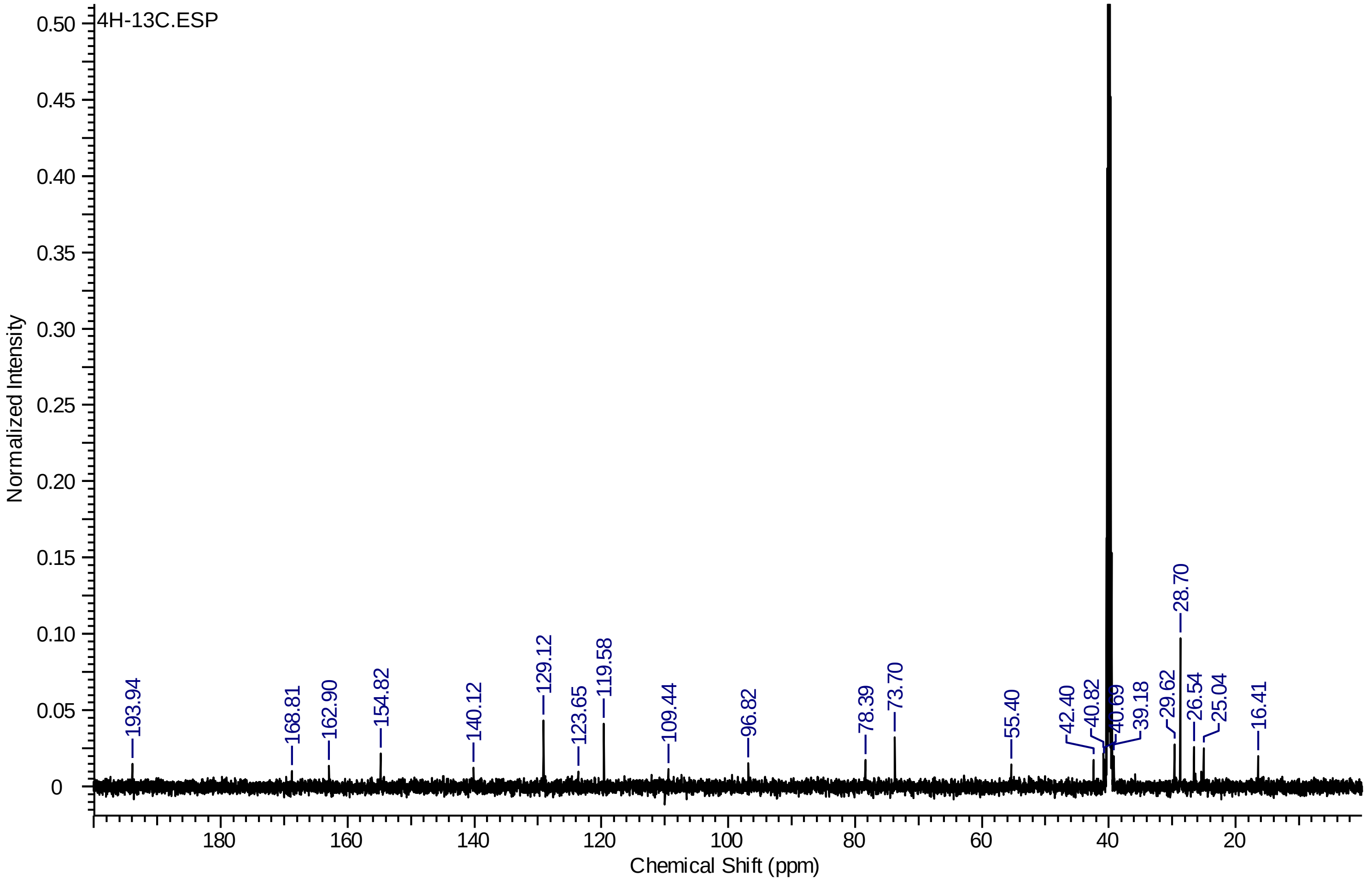
4H-13C.ESP
Normalized Intensity
Chemical Shift (ppm)
193.94 168.81 162.90 154.82 140.12 129.12 123.65 119.58 109.44 96.82 78.39 73.70 55.40 42.40 40.82 40.69 39.18 29.62 28.70 26.54 25.04 16.41

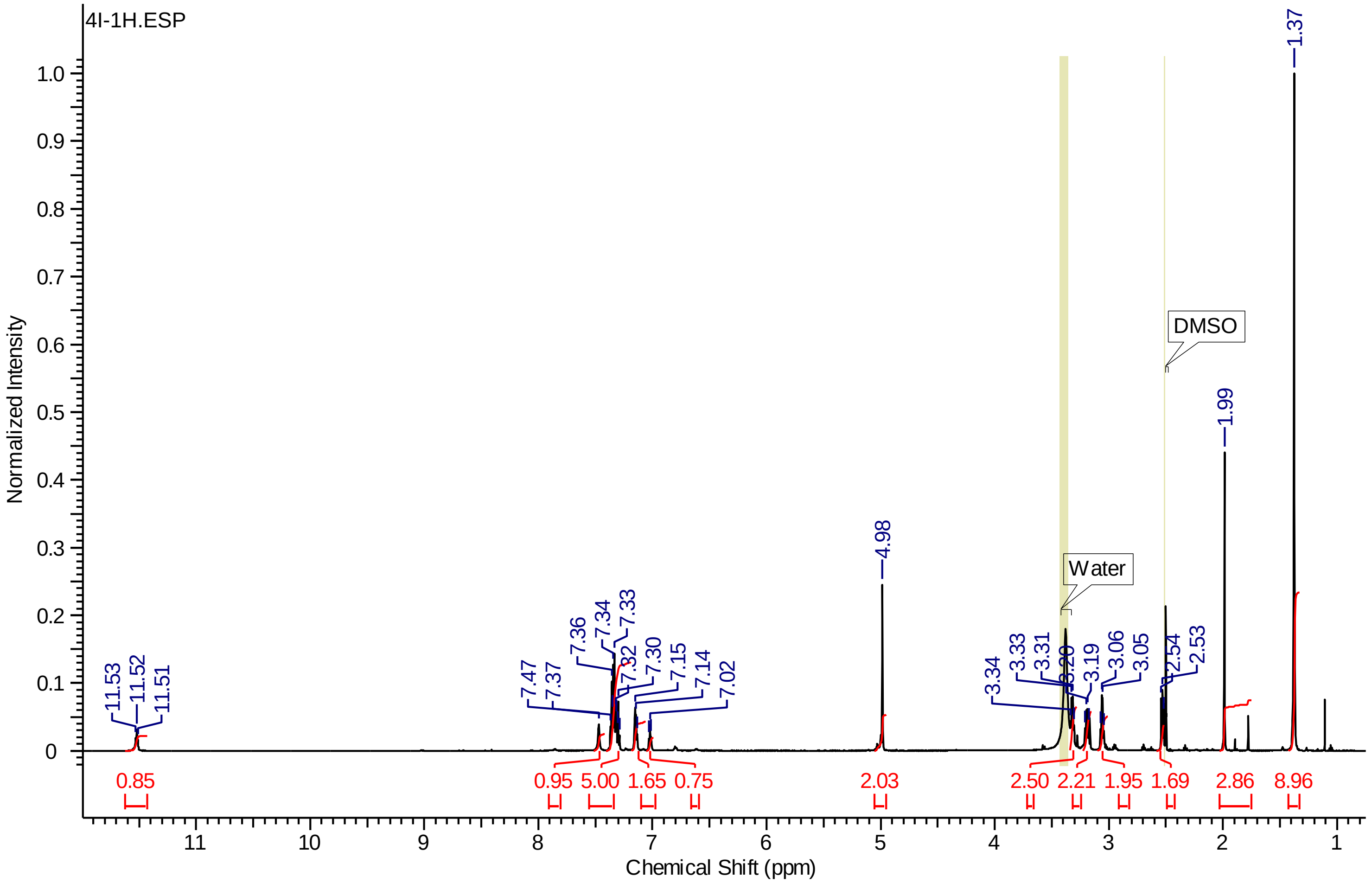
4I-1H.ESP
Normalized Intensity
Chemical Shift (ppm)
Water
DMSO
11.53
11.52
11.51
7.47
7.37
7.36
7.34
7.33
7.32
7.30
7.15
7.14
7.02
4.98
3.34
3.33
3.31
3.20
3.19
3.06
3.05
2.54
2.53
1.99
1.37
0.85
0.95
5.00
1.65
0.75
2.03
2.50
2.21
1.95
1.69
2.86
8.96

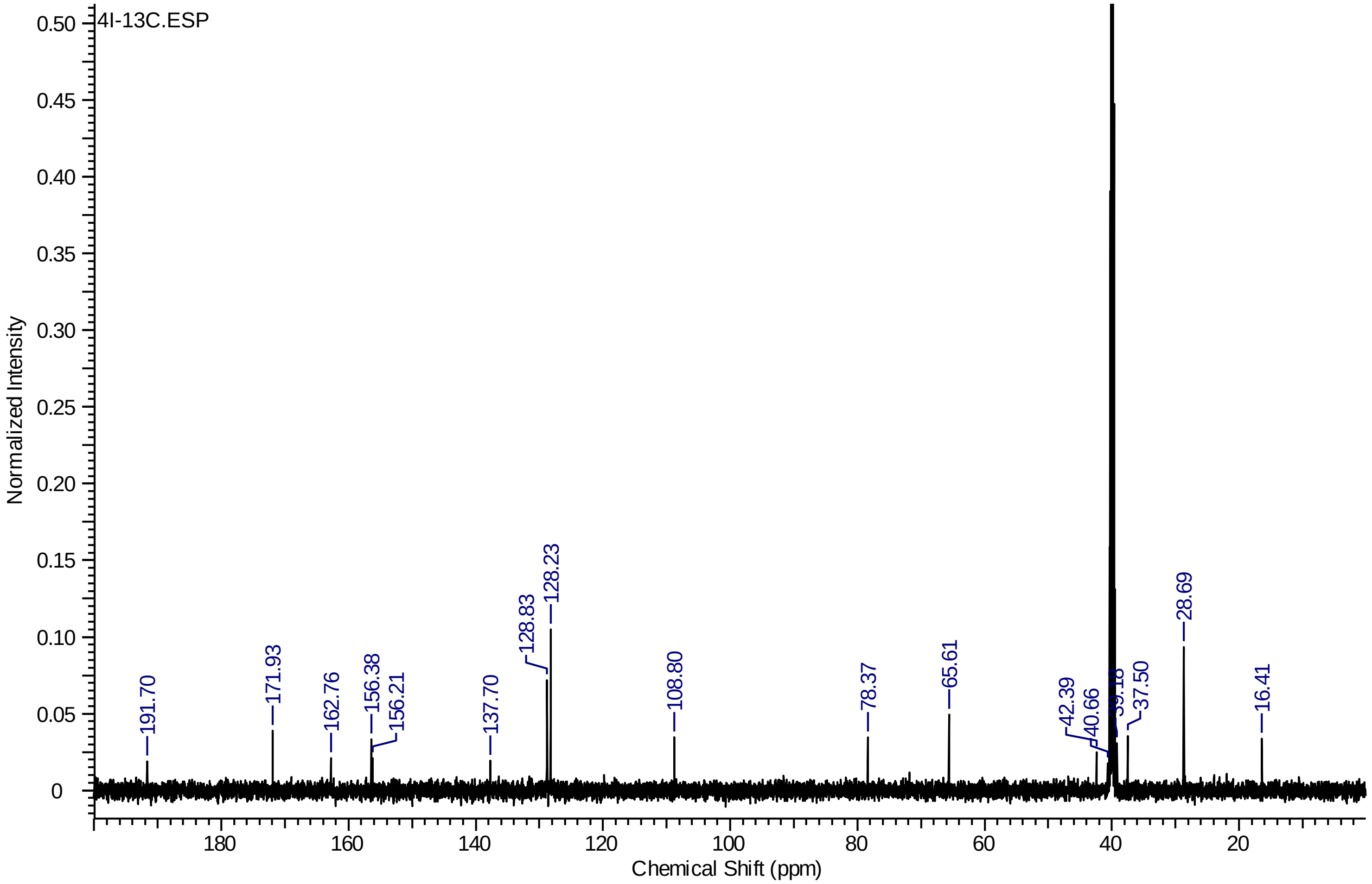
4I-13C.ESP
Normalized Intensity
Chemical Shift (ppm)
191.70
171.93
162.76
156.38
156.21
137.70
128.83
128.23
108.80
78.37
65.61
42.39
40.66
39.18
37.50
28.69
16.41

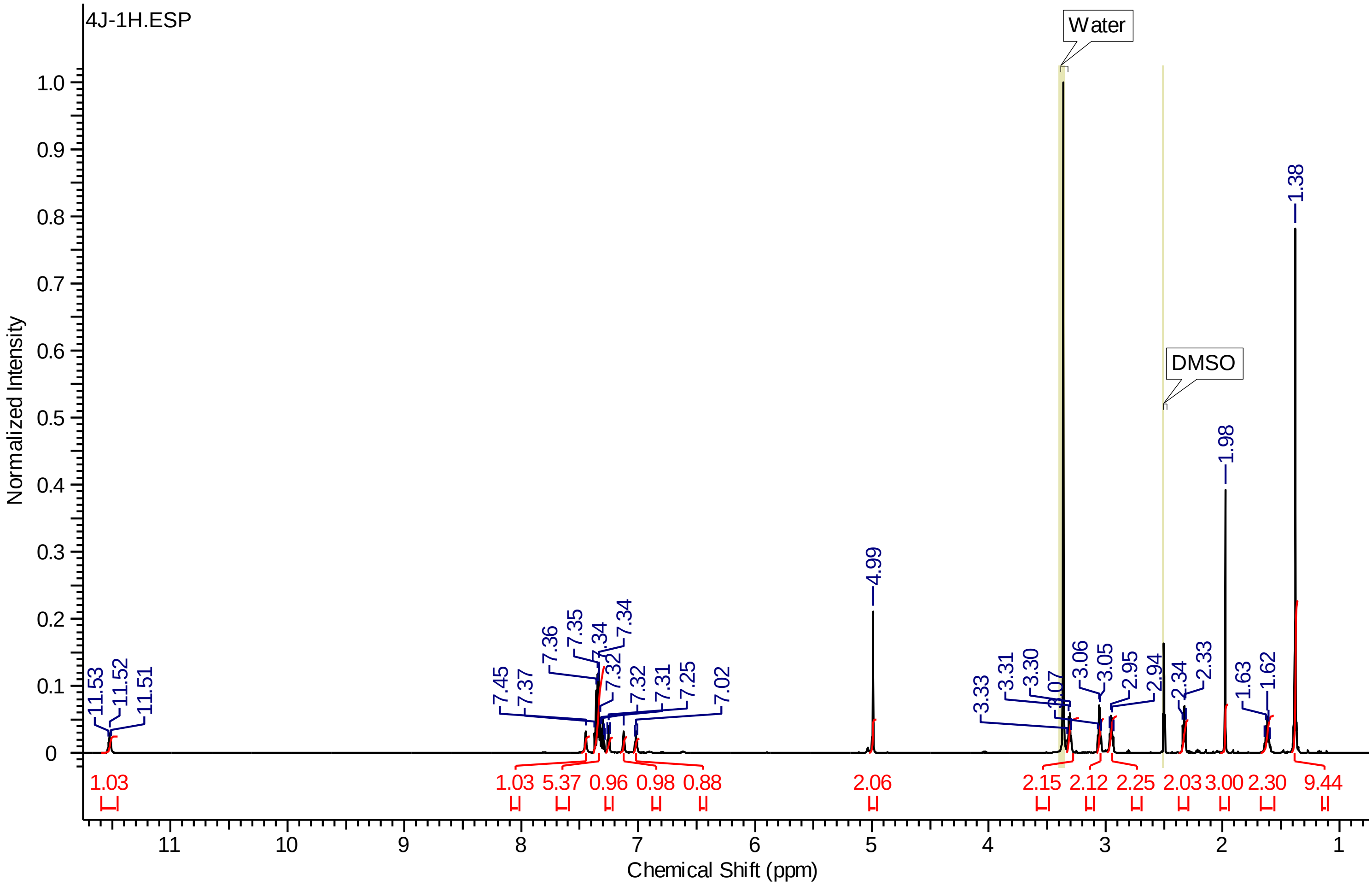
4J-1H.ESP
Water
DMSO
Normalized Intensity
Chemical Shift (ppm)
11.53
11.52
11.51
7.45
7.37
7.36
7.35
7.34
7.34
7.32
7.32
7.31
7.25
7.02
4.99
3.33
3.31
3.30
3.07
3.06
3.05
2.95
2.94
2.34
2.33
1.98
1.63
1.62
1.38
1.03
1.03
5.37
0.96
0.98
0.88
2.06
2.15
2.12
2.25
2.03
3.00
2.30
9.44

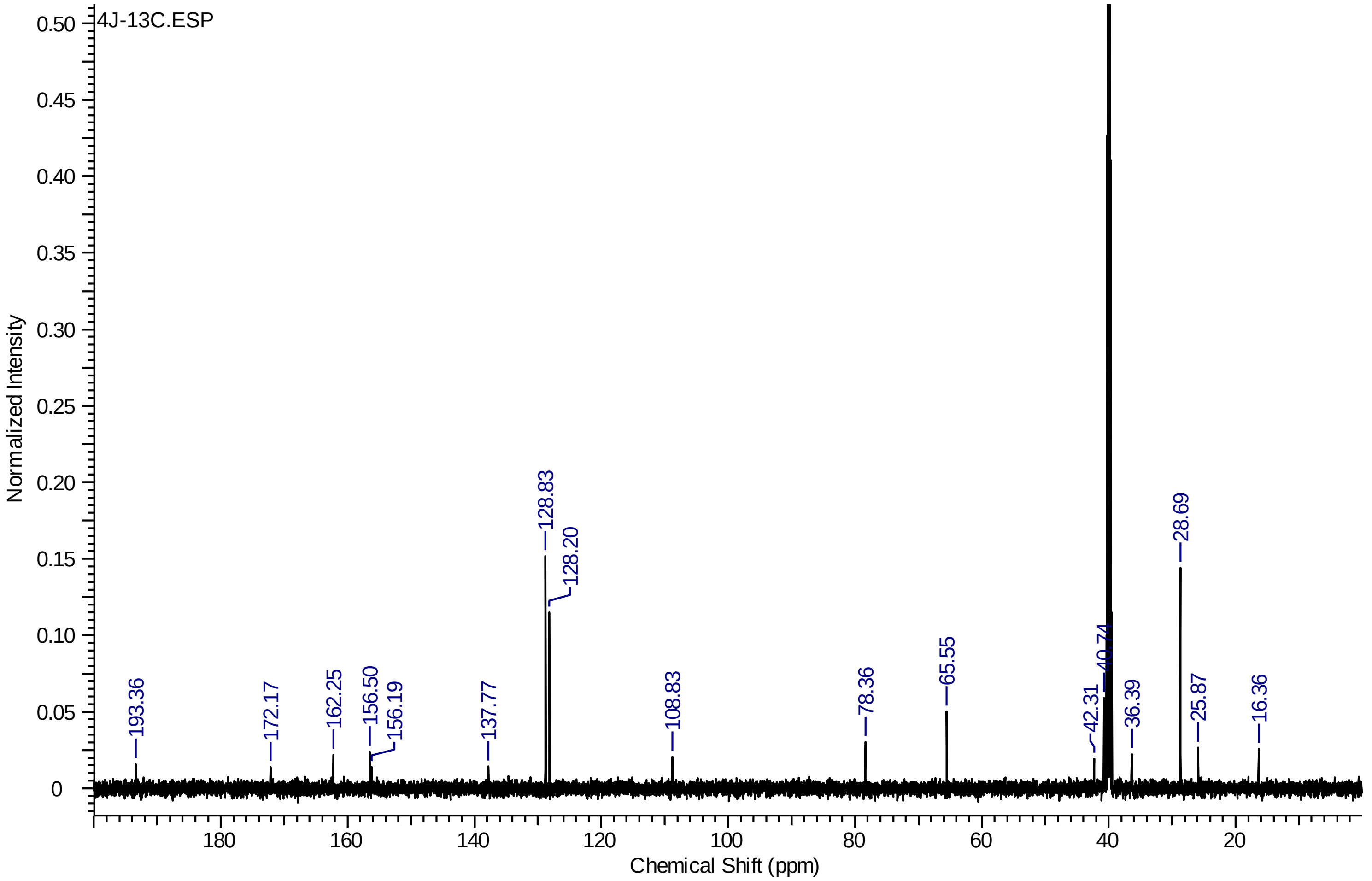
4J-13C.ESP
Normalized Intensity
Chemical Shift (ppm)
193.36
172.17
162.25
156.50
156.19
137.77
128.83
128.20
108.83
78.36
65.55
42.31
40.74
36.39
28.69
25.87
16.36

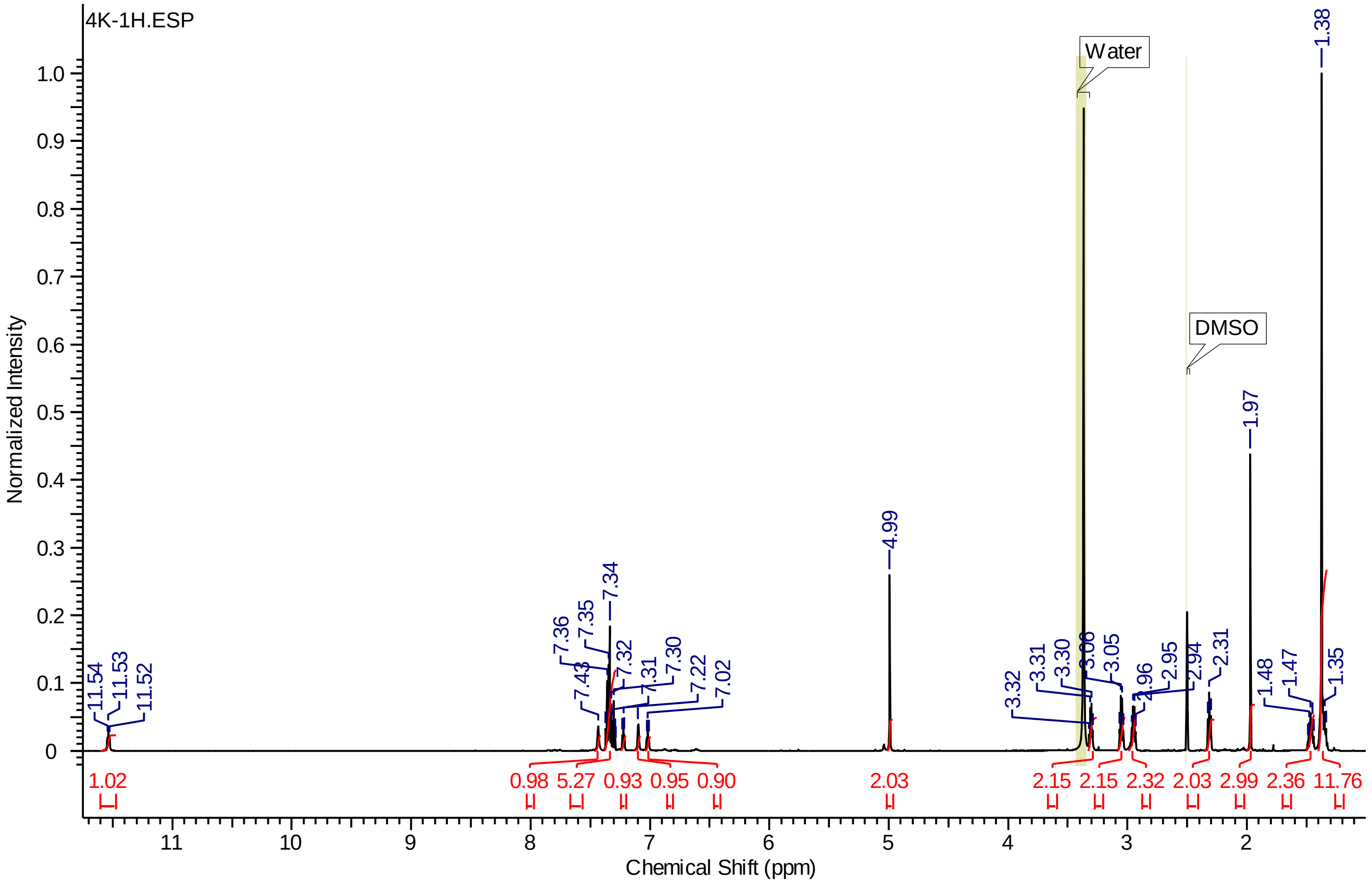
4K-1H.ESP
Water
DMSO
Normalized Intensity
Chemical Shift (ppm)
11.54 11.53 11.52
7.43 7.36 7.35 7.34 7.32 7.31 7.30 7.22 7.02
4.99
3.32 3.31 3.30 3.06 3.05 2.96 2.95 2.94 2.31
1.97
1.48 1.47 1.38 1.35
1.02 0.98 5.27 0.93 0.95 0.90 2.03 2.15 2.15 2.32 2.03 2.99 2.36 11.76

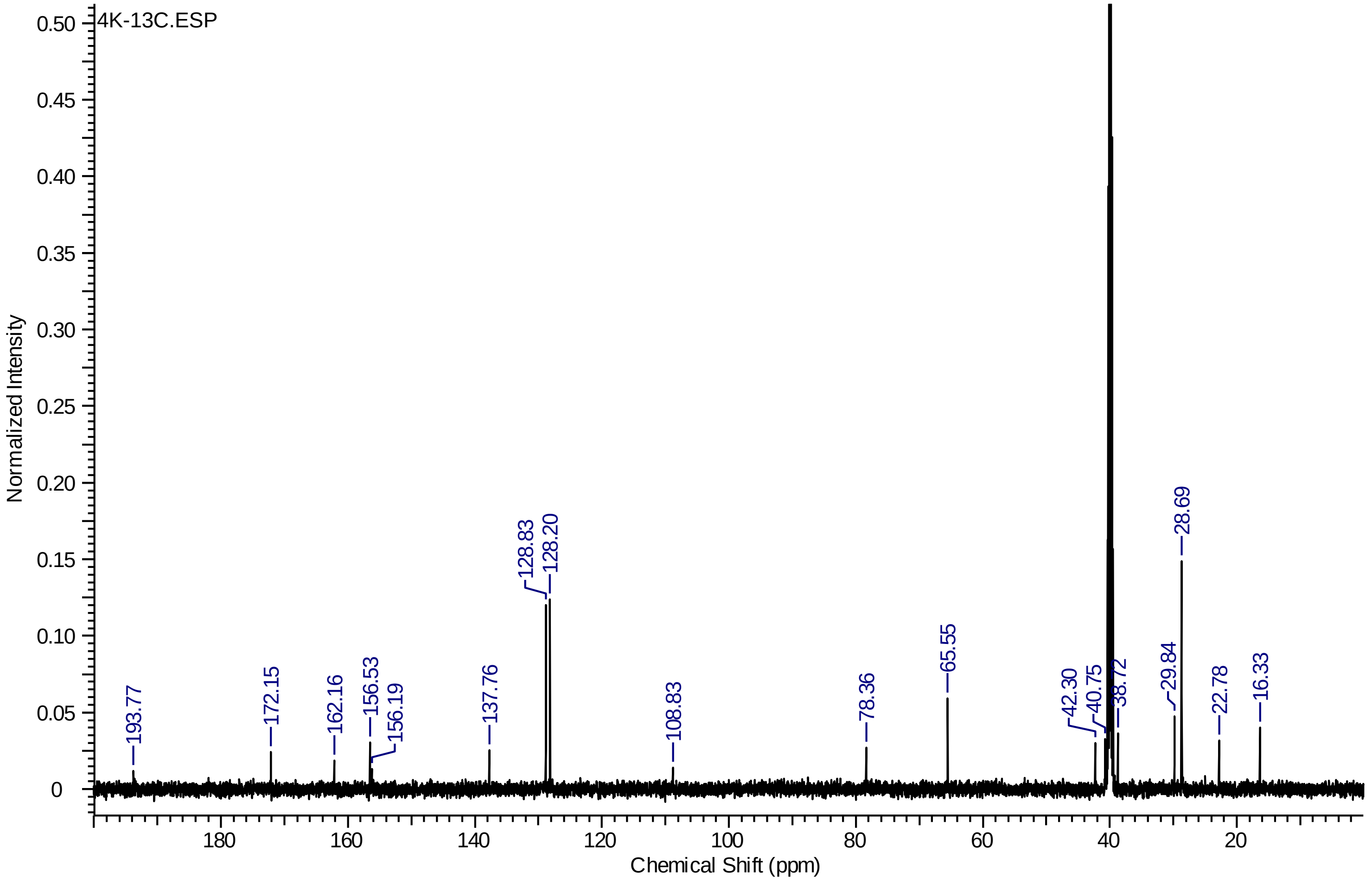
4K-13C.ESP
Normalized Intensity
Chemical Shift (ppm)
193.77 172.15 162.16 156.53 156.19 137.76 128.83 128.20 108.83 78.36 65.55 42.30 40.75 38.72 29.84 28.69 22.78 16.33

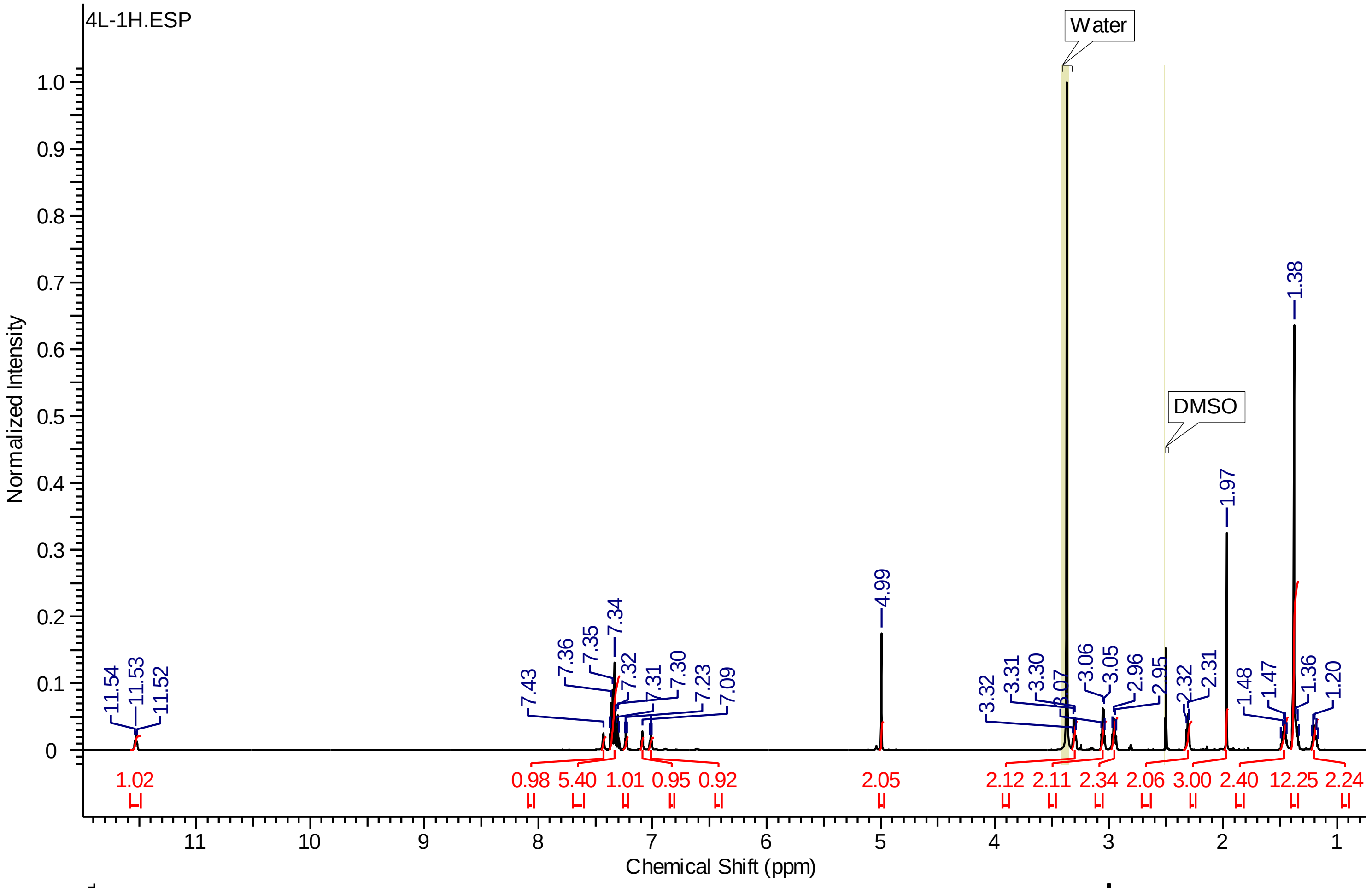
4L-1H.ESP
Water
DMSO
Normalized Intensity
Chemical Shift (ppm)
11.54
11.53
11.52
7.43
7.36
7.35
7.34
7.32
7.31
7.30
7.23
7.09
4.99
3.32
3.31
3.30
3.07
3.06
3.05
2.96
2.95
2.32
2.31
1.97
1.48
1.47
1.38
1.36
1.20
1.02
0.98
5.40
1.01
0.95
0.92
2.05
2.12
2.11
2.34
2.06
3.00
2.40
12.25
2.24

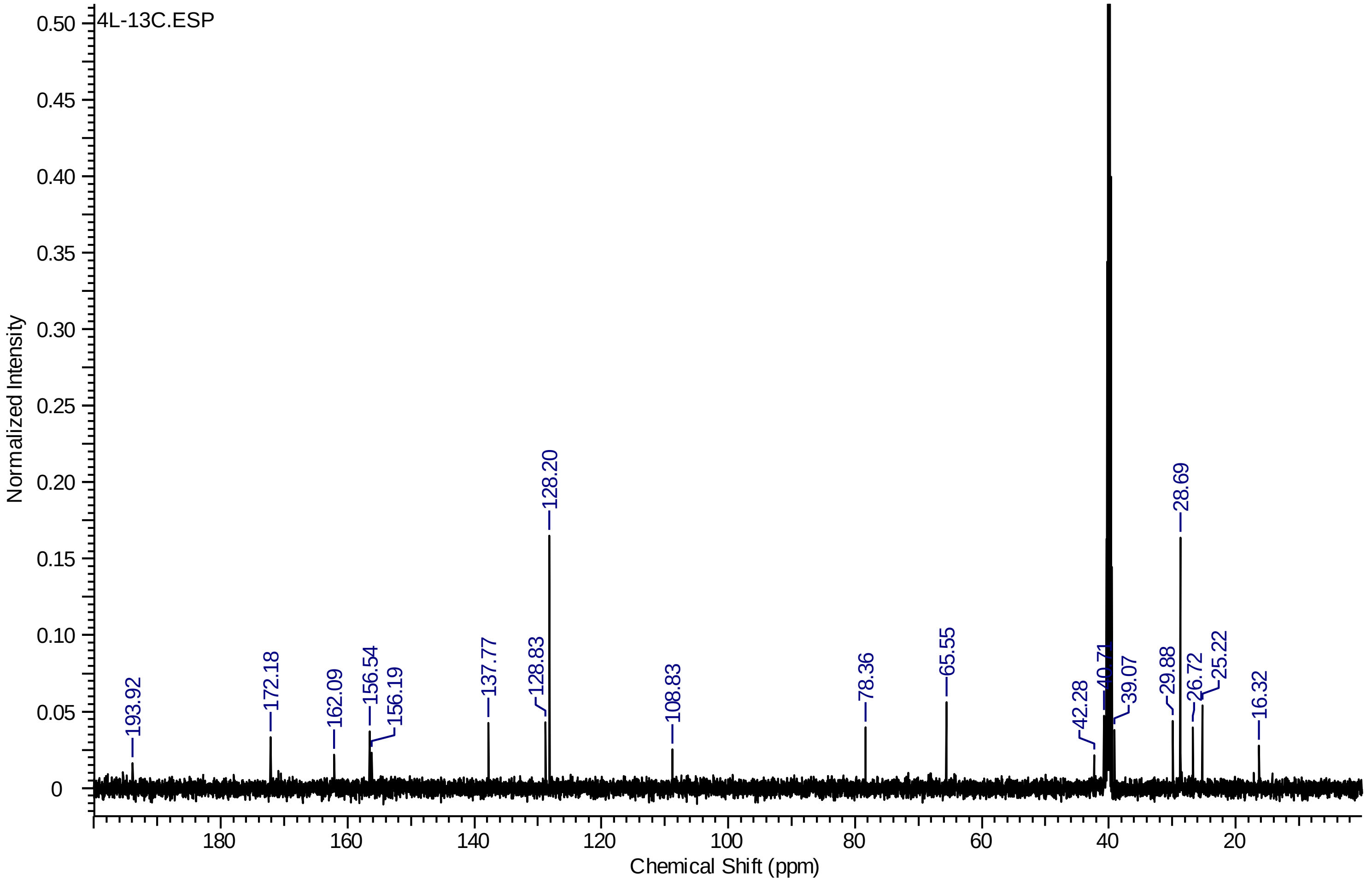
4L-13C.ESP
Normalized Intensity
Chemical Shift (ppm)
193.92
172.18
162.09
156.54
156.19
137.77
128.83
128.20
108.83
78.36
65.55
42.28
40.71
39.07
29.88
28.69
26.72
25.22
16.32

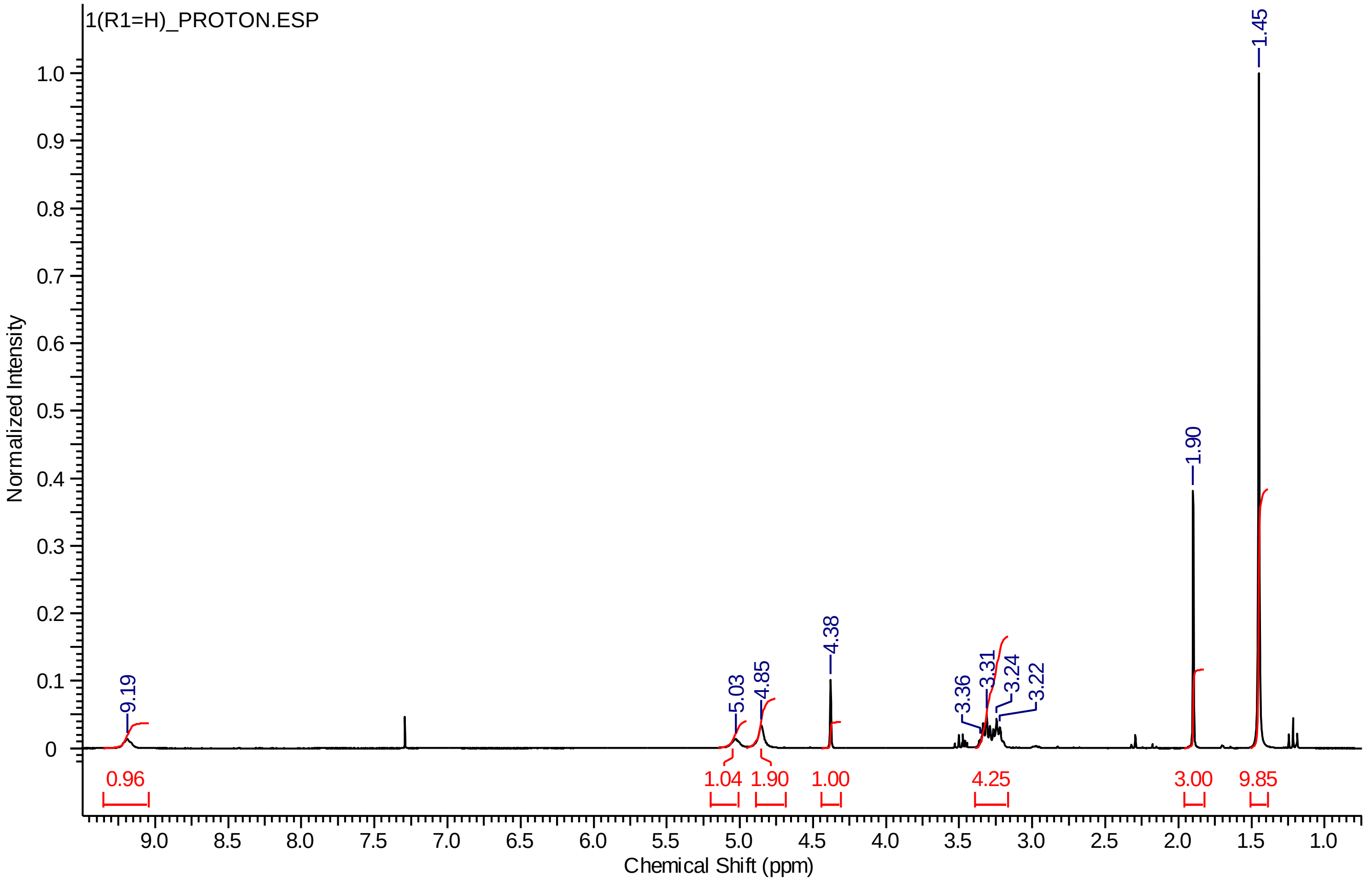
1(R1=H)_PROTON.ESP
Normalized Intensity
Chemical Shift (ppm)
9.19
5.03
4.85
4.38
3.36
3.31
3.24
3.22
1.90
1.45
0.96
1.04
1.90
1.00
4.25
3.00
9.85

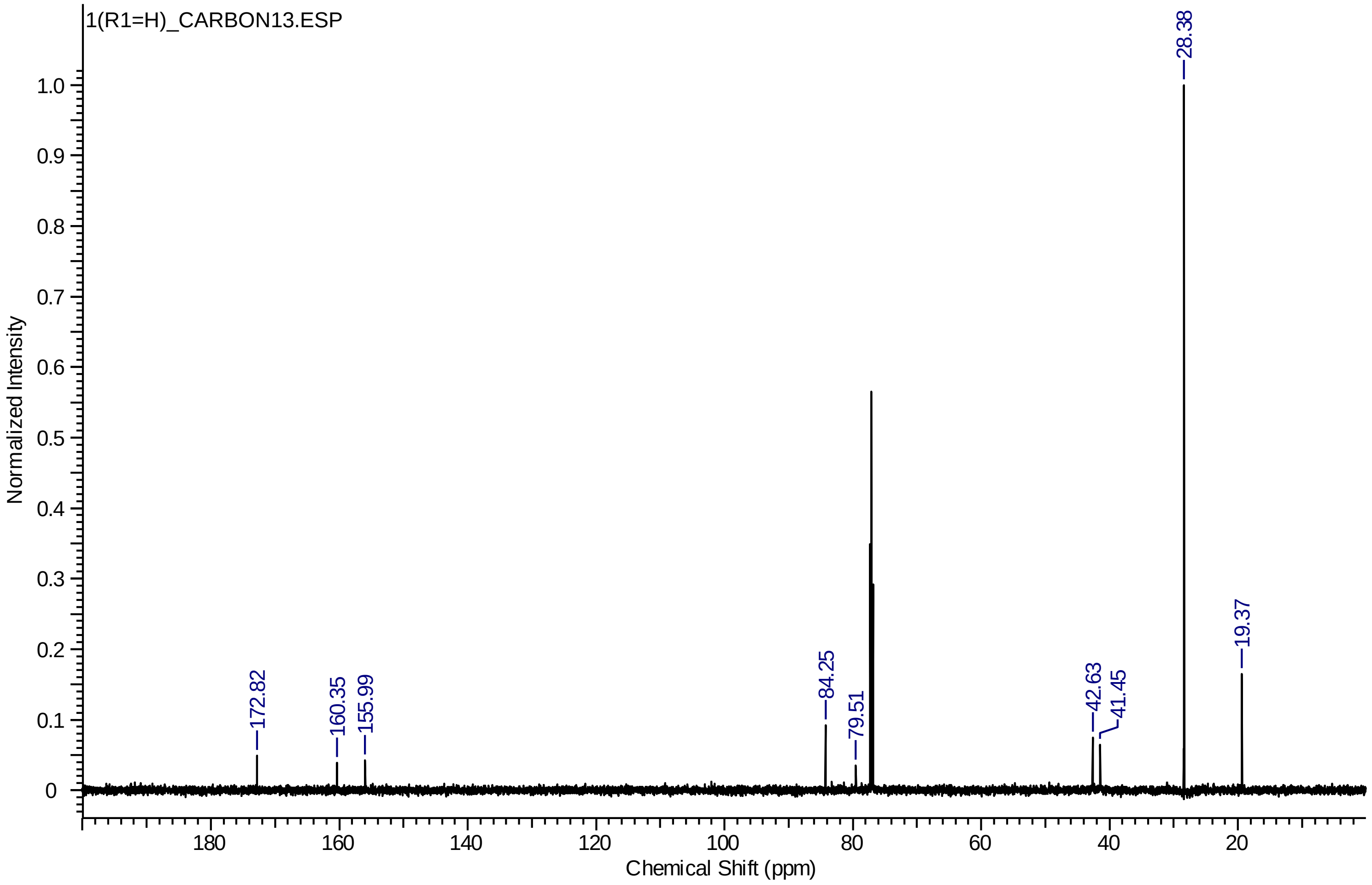
1(R1=H)_CARBON13.ESP
Normalized Intensity
Chemical Shift (ppm)
172.82
160.35
155.99
84.25
79.51
42.63
41.45
28.38
19.37

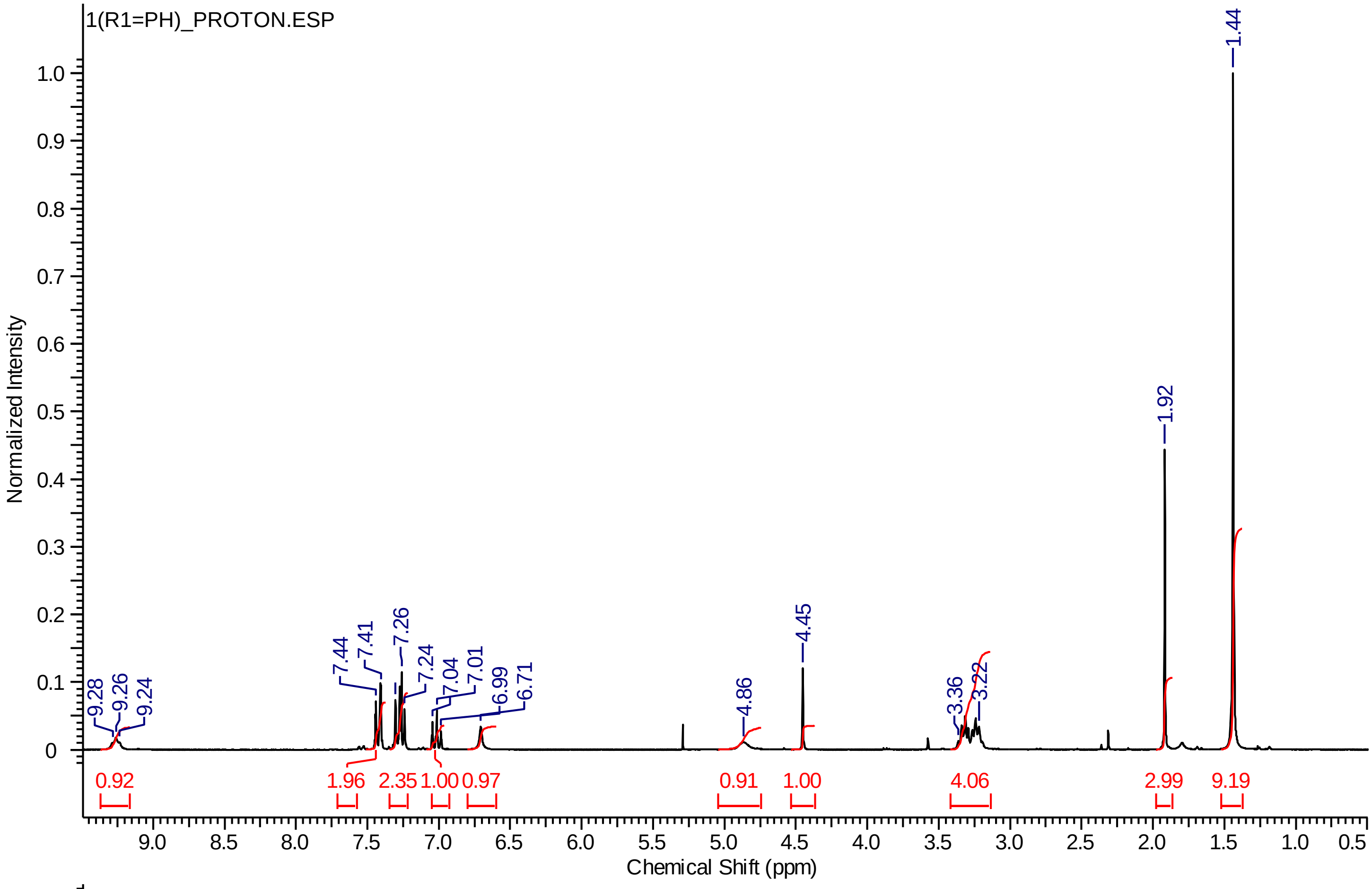
1(R1=PH)_PROTON.ESP
Normalized Intensity
Chemical Shift (ppm)
9.28
9.26
9.24
7.44
7.41
7.26
7.24
7.04
7.01
6.99
6.71
4.86
4.45
3.36
3.22
1.92
1.44
0.92
1.96
2.35
1.00
0.97
0.91
1.00
4.06
2.99
9.19

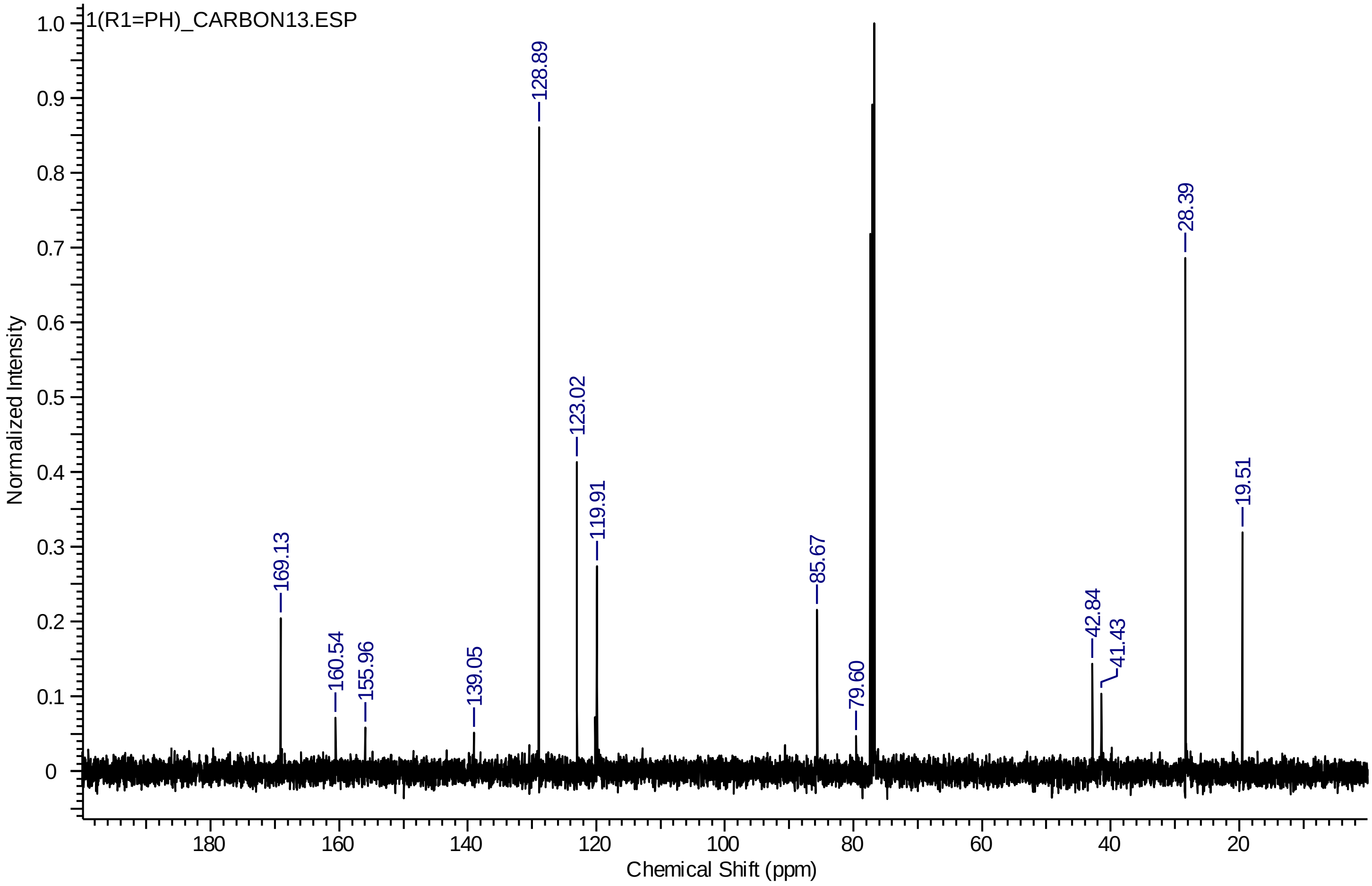
1(R1=PH)_CARBON13.ESP
Normalized Intensity
Chemical Shift (ppm)
169.13
160.54
155.96
139.05
128.89
123.02
119.91
85.67
79.60
42.84
41.43
28.39
19.51
